# The Landscape of Host Transcriptional Response Programs Commonly Perturbed by Bacterial Pathogens: Towards Host-Oriented Broad-Spectrum Drug Targets

**DOI:** 10.1371/journal.pone.0058553

**Published:** 2013-03-13

**Authors:** Yared H. Kidane, Christopher Lawrence, T. M. Murali

**Affiliations:** 1 Genetics, Bioinformatics, and Computational Biology PhD Program, Virginia Tech, Blacksburg, Virginia, United States of America; 2 Virginia Bioinformatics Institute, Virginia Tech, Blacksburg, Virginia, United States of America; 3 Department of Biology, Virginia Tech, Blacksburg, Virginia, United States of America; 4 Department of Computer Science, Virginia Tech, Blacksburg, Virginia, United States of America; 5 ICTAS Center for Systems Biology of Engineered Tissues, Virginia Tech, Blacksburg, Virginia, United States of America; Kansas State University, United States Of America

## Abstract

**Background:**

The emergence of drug-resistant pathogen strains and new infectious agents pose major challenges to public health. A promising approach to combat these problems is to target the host’s genes or proteins, especially to discover targets that are effective against multiple pathogens, i.e., host-oriented broad-spectrum (HOBS) drug targets. An important first step in the discovery of such drug targets is the identification of host responses that are commonly perturbed by multiple pathogens.

**Results:**

In this paper, we present a methodology to identify common host responses elicited by multiple pathogens. First, we identified host responses perturbed by each pathogen using a gene set enrichment analysis of publicly available genome-wide transcriptional datasets. Then, we used biclustering to identify groups of host pathways and biological processes that were perturbed only by a subset of the analyzed pathogens. Finally, we tested the enrichment of each bicluster in human genes that are known drug targets, on the basis of which we elicited putative HOBS targets for specific groups of bacterial pathogens. We identified 84 up-regulated and three down-regulated statistically significant biclusters. Each bicluster contained a group of pathogens that commonly dysregulated a group of biological processes. We validated our approach by checking whether these biclusters correspond to known hallmarks of bacterial infection. Indeed, these biclusters contained biological process such as inflammation, activation of dendritic cells, pro- and anti- apoptotic responses and other innate immune responses. Next, we identified biclusters containing pathogens that infected the same tissue. After a literature-based analysis of the drug targets contained in these biclusters, we suggested new uses of the drugs Anakinra, Etanercept, and Infliximab for gastrointestinal pathogens *Yersinia enterocolitica*, *Helicobacter pylori* kx2 strain, and enterohemorrhagic *Escherichia coli* and the drug Simvastatin for hematopoietic pathogen *Ehrlichia chaffeensis*.

**Conclusions:**

Using a combination of automated analysis of host-response gene expression data and manual study of the literature, we have been able to suggest host-oriented treatments for specific bacterial infections. The analyses and suggestions made in this study may be utilized to generate concrete hypothesis on which gene sets to probe further in the quest for HOBS drug targets for bacterial infections. All our results are available at the following supplementary website: http://bioinformatics.cs.vt.edu/ murali/supplements/2013-kidane-plos-one

## Introduction

Infectious diseases are the second leading cause of death worldwide, next to cardiovascular diseases [1]. Bacterial infections such as tuberculosis and food- and water- borne infections from *Salmonella enterica* and *Escherichia coli* still present many challenges to biomedical researchers. Foremost among these challenges is that infectious agents rapidly mutate and become resistant to drugs [2]. The conventional approach of targeting pathogen proteins has accelerated the spread of resistance, resulting in the re-emergence of once-contained infectious diseases, such as those caused by multidrug-resistant strains of *Mycobacterium tuberculosis, Staphylococcus aureus*, and *Salmonella enterica* [3]. In an effort to combat the issue of drug resistance, anti-infective drug discovery is shifting to a new approach that targets the host instead of pathogens [3], [4]. “Host-oriented” drug discovery focuses on manipulating or subverting biological processes in the host that pathogens utilize [5]. Another problem facing the treatment of infectious diseases is the increasing number of pathogenic agents [6]. Furthermore, new pathogens are appearing regularly, e.g., the pandemic swine flu H1N1 virus recognized in 2009. The expanding range of infectious agents coupled with the high cost associated with drug discovery have made it economically infeasible and practically impossible to tackle each pathogen individually [6], [7]. These factors have necessitated treatment regimens that are effective against a wide variety of infectious agents.

These factors have encouraged efforts in host-oriented broad-spectrum (HOBS) drug discovery, i.e., finding targets in the host that can simultaneously cure multiple infections [3], [8]. Examples of HOBS drugs currently available in the market include Statins and Isoprinosine. Statins are used in the treatment of *Leishmania, Staphylococcus aureus*, and HIV infections [9]–[11]. Statins lower the cholesterol level in human body. They are effective against pathogens that utilize cholesterol in binding and internalization to the host cell. Isoprinosine, which stimulates the proliferation of T-cells, is used in the treatment of *Herpes simplex*, *Hepatitis*, and *Epstein-Barr* virus infections [12].

A first and important step in HOBS drug discovery is the development of computational tools to discover common physiological processes and cellular pathways that different pathogens utilize to infect, proliferate, and spread in the host. We hypothesized that comprehensive molecular datasets of host responses to diverse varieties of pathogens might form a powerful resource to discover such pathways. Transcriptional datasets that correspond to different infectious diseases, cell/tissue types, and organisms are the most abundantly available. Meta-analysis of transcriptional datasets have been performed for a wide range of diseases. For instance, Rhodes *et al.* [13] analyzed 40 cancer related microarray datasets to identify common signatures of cancer. English and Butte [14] integrated 49 obesity-related genome-wide experiments obtained from human, mouse, rat, and worm to predict new genes that may be associated with obesity. Magalhaes *et al.* [15] performed meta-analysis of 27 age-related gene expression profile datasets from human, mouse, and rat to reveal several common signatures of aging. Jenner *et al.* [16] used hierarchical clustering of gene expression profiles of 77 pathogens in order to find genes that exhibited similar expression profiles across several disease types.

Recent approaches have taken meta-analysis of DNA microarray datasets one step further by incorporating drug targets into the analysis and inferring new uses for existing drugs on the basis of disease similarities. The premise underlying these approaches is that diseases with a high degree of transcriptional similarity might be treated with similar drugs [17]. Hu *et al.* [18] discovered disease-disease links by using correlation-based methods and gene set enrichment analysis to measure the similarities between gene expression profiles of diseases. They also integrated gene expression profiles that pertain to responses of cell lines to drugs derived from the Connectivity Map [19] to create a drug-disease network where clusters of drugs and diseases suggested shared drug mechanisms and molecular disease pathology. Suthram *et al.* [20] performed integrative analysis of 54 disease-related mRNA expression datasets. They measured the perturbation of predefined protein functional modules using the mean normalized transcriptional activity of each module's component genes in the disease's transcriptional profile. Furthermore, they identified known drug targets in the modules that were perturbed by multiple disease types, which they proposed as pluripotent/broad-spectrum drug targets .

The goal of our work is similar to that of Jenner *et al.* , Hu *et al.* , and Suthram *et al.* : to discover transcriptional responses common to many diseases, specifically those caused by bacterial pathogens, and to discover existing drug targets within those transcriptional signatures. The previous authors have used global correlation measures to detect disease associations, which may obscure relationships that exist over only a subset of the diseases or genes. In contrast, we use a combination of gene set level enrichment and biclustering. As we demonstrate in this work, this approach enables us to group sets of host genes that are dysregulated only by a subset of the pathogens, facilitating the capture of pathway-specific relationships among groups of pathogens.

## Results

**Figure 1:**
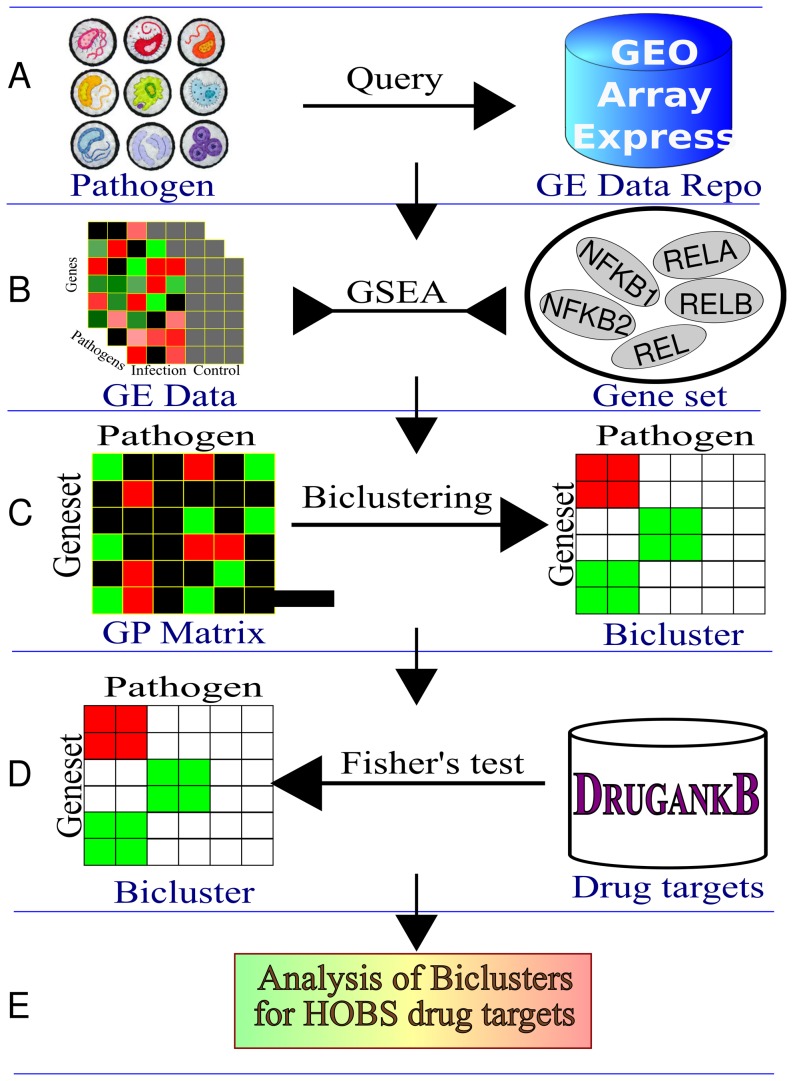
Overview of our system. Overview of our computational system to compute host-oriented broad-spectrum drug targets. (A) Obtaining relevant collection of taxonomic names for human bacterial pathogens. Querying the GEO metadatabase in search of relevant transcriptional datasets. (B) Gene Set Enrichment Analysis of the transcriptional datasets collected in Step A. (C) Identification of pathogen-gene set biclusters and estimation of statistical significance of biclusters (D) Testing bicluster enrichment for known drug targets. (E) Literature analysis of putative HOBS drug targets contained in biclusters.

We start with an overview of the method (Figure 1). We obtained genome-wide transcriptional data sets of host responses after infection by bacterial pathogens from the NCBI's Gene Expression Omnibus (GEO) (Figure 1A). After data filtering (see Methods), we retained 29 gene expression profiling studies which represent 213 host samples and 38 bacterial pathogens or pathogen strains. We sub-divided the datasets into four major kinds of infection: gastrointestinal, oral cavity, hematopoietic, and respiratory. A complete description of these datasets and their GEO accession numbers is provided in Table S1.

Since these datasets were generated by different research groups with different objectives in mind, they tended to be very diverse, e.g., in the microarray platform used, the infected host, and the tissue or cell type from which the gene expression measurements were taken. Such variations made the direct comparison of the datasets difficult. To alleviate this problem, we computed gene sets perturbed by each pathogen using Gene Set Enrichment Analysis (GSEA) (Figure 1B), thereby enabling comparison across pathogens at the level of perturbed gene sets. We recorded all pathogens and the gene sets they perturbed in a matrix. Next, we biclustered this matrix in order to identify all subsets of the gene sets that were co-perturbed across a subset of the pathogens (Figure 1C). We assessed the statistical significance of the biclusters by comparing their sizes to biclusters found in randomized matrices. This process yielded 84 up-regulated and three down-regulated significant biclusters at a 0.05 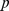-value cutoff, after adjusting for multiple-hypothesis testing [21] (Tables S2 and S3). In this paper, we focus our discussion on up-regulated biclusters as (a) they are far greater in number than down-regulated biclusters and (b) up-regulated genes and pathways may be controlled, in general, by drugs that prevent function of their targets. We used Fisher's exact test to estimate the enrichment of a bicluster in known drug targets (Figure 1D). We acknowledge that even a bicluster with a single drug target may be worthy of study. We computed bicluster enrichment in drug targets in order to prioritize biclusters for examination since we had a large number of biclusters. Finally, we searched the literature for biologically meaningful connections among the gene sets, pathogens, and drug targets in a bicluster in order to find support for the hypothesis that modulating the activity of the drug targets may control the infection caused by the pathogens (Figure 1E).

We have organized the results from our study into two major sections. First, we asked if the biclusters we computed could reveal well-known immunological responses in the host to bacterial infection. To this end, we identified host gene sets that were contained in those biclusters that were also perturbed by many pathogens. Our analysis revealed that biological functions pertaining to the up-regulation of inflammatory gene sets, Lipopolysaccharide (LPS)-inducible gene sets, innate immunity response, induction and inhibition of apoptosis, and maturation of dendritic cells are host responses that are triggered by most of the bacterial pathogens. Rediscovering well known host responses to infection established the validity of our approach in detecting common host signatures. Second, we analyzed the biclusters for putative HOBS targets. Out of the 84 significantly up-regulated biclusters, 47 of them were enriched in known drug targets at the 0.05 significance level (Table S2). We identified seven biclusters where all the pathogens contained in each of these biclusters infected a single tissue or organ in the human body. For instance, in bicluster 38, we found four gastrointestinal pathogens, namely, *Yersinia enterocolitica* wap and p60 strains, *Helicobacter pylori* kx2 strain, and Enterohemorrhagic *Escherichia coli*. From this bicluster, we suggested the potential use of chronic inflammation suppressors such as Anakinra, Etanercept and Infliximab in treating infection caused by these four pathogens.

### Gene Sets Perturbed in Response to Bacterial Infection

There are several stages and outcomes that are hallmarks of generalized infection. On one hand, pathogens try to enter, multiply, and spread in the host, causing disease. On the other hand, hosts attempt to defend the attack from pathogens using processes conferring innate and adaptive immunity, leading to the elimination of pathogens. There are different strategies that are utilized by pathogens and by hosts to achieve these objectives. Among other things, pathogens induce or inhibit apoptosis, import their genetic material into the host, and replicate their genome [22], [23]. Hosts utilize various arms of the immune system such as inflammation, response to stimulus, maturation of dendritic cells and activation of various components of the innate immunity to lessen pathogenicity.

The 84 statistically significant up-regulated biclusters contained 1,364 distinct gene sets and 34 pathogens. To determine if our biclusters capture the hallmarks of infection mentioned above, we asked which gene sets belonged to the largest number of biclusters. Upon ranking the gene sets in decreasing order of number of biclusters they were perturbed in, we observed that the number of biclusters that a gene set was contained in had a high positive correlation (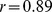, 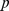-value 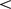 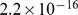) with the number of pathogens that perturb the gene set (Figure S1). Table 1 shows the top ten gene sets in this ranked list. Then, for each gene set, we assigned Gene Ontology (GO) biological processes for intuitive interpretation (Table 2) using the procedure described in Methods. We now proceed to discuss these highly-ranked gene sets and correlate them to well-known hallmarks of infection.

**Table 1:** Gene sets perturbed in many pathogens.

Gene Set	# Pathogens	# Biclusters
Zhang Response to IKK Inhibitor and TNF up	33	83
Seki Inflammatory Response LPS up	33	83
Dirmeier LMP1 Response Early	32	76
Dauer STAT3 Targets up	31	75
Hinata NFKB Targets Keratinocyte up	31	74
Tian TNF Signaling via NFKB	32	73
Lindstedt Dendritic Cell Maturation B	30	67
Uzonyi Response to Leukotriene and Thrombin	31	63
Netpath IL 4 Pathway Down	30	59
Mahadevan Response to MP470 up	30	53

For each gene set, the table shows the number of pathogens that perturb it and the number of biclusters it appears in.

**Table 2:** Mapping of Gene Sets to GO Biological Processes.

Gene Set	GO Enriched Processes (Top Three)	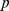 -value
Zhang Response to IKK Inhibitor and TNF up	Inflammatory Response	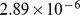
	Response to Wounding	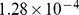
	Defense Response	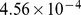
Seki Inflammatory Response LPS up	Locomotory Behavior	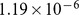
	Response to External Stimulus	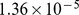
	Defense Response	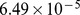
Dirmeier LMP1 Response Early	Apoptosis GO	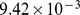
	Programmed Cell Death	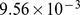
	Viral Genome Replication	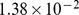
Dauer STAT3 Targets up	Cyclic Nucleotide Metabolic Process	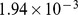
	Protein Import into Nucleus Translocation	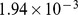
	DNA Damage Response Signal Transduction Resulting in Induction of Apoptosis	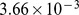
Hinata NFKB Targets Keratinocyte up	Response to Wounding	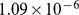
	Inflammatory Response	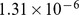
	Response to Stress	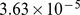
Tian TNF Signaling via NFKB	Defense Response	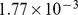
	Regulation of I KAPPAB Kinase NF KAPPAB Cascade	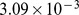
	Response to Wounding	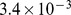
Lindstedt Dendritic Cell Maturation B	Apoptosis GO	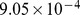
	Programmed Cell Death	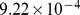
	Cell Development	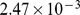
Uzonyi Response to Leukotriene and Thrombin	Heart Development	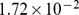
	Inflammatory Response	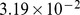
	Regulation of Transcription	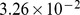
Netpath IL 4 Pathway Down	Activation of Innate Immune Response	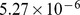
	Pattern Recognition Receptor Signaling Pathway	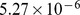
	Toll-like Receptor Signaling Pathway	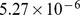
Mahadevan Response to MP470 up	Locomotory Behavior	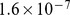
	Defense Response	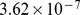
	Inflammatory Response	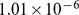

The table shows top three GO biological processes that have the highest overlap with each of the ten most frequently perturbed gene sets (in Table 1). The 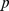-value indicates the statistical significance of the overlap, based on Fisher's exact test.

#### Inflammatory Response

Inflammation is one of the immediate reactions by the host against pathogenic infections. Of the top ten gene sets, four gene sets have a high overlap with genes annotated with GO's inflammatory response process (GO:0006954; “Zhang Response to IKK Inhibitor and TNF up”, “Uzonyi Response to Leukotriene and Thrombin”, “Hinata NFKB Targets Keratinocyte up”, and “Mahadevan Response to MP470 up”). For each of these gene sets, we describe the experiment that generated it. We note that these experiments were conducted in diverse tissues and were not directly related to pathogen infection. Nevertheless, by examining the connection between each of these gene sets and inflammation, we demonstrate that inflammation is a non-specific response triggered by many of the pathogens irrespective of the type of cell being infected. The gene set “Zhang Response to IKK Inhibitor and TNF up” is perturbed in 83 biclusters spanning 33 different bacterial pathogens. This gene set contains 219 genes that are up-regulated in BxPC3 pancreatic cancer cells after treatment with tumor necrosis factor (TNF)-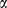, a pro-inflammatory cytokine [24]. This gene set consists of genes encoding for pro-inflammatory mediators such as IL1A, IL1B, TNFSF10 and a number of other chemokines including CCL20, CCL5, CXCL1, CXCL10, CXCL11, CXCL16, CXCL2, and CXCL3. The next set in the list is “Hinata NFKB Targets Keratinocyte up”, which was perturbed by 31 pathogens and appeared in 74 biclusters. This gene set contains 71 genes that were up-regulated in primary keratinocyte cells after transduction with NF-kappa B [25]. The majority of the genes in this gene set are cytokines and growth factor genes including chemokines ( CCL20, CCL5, CXCL10, CXCL11, CXCL3, CXCL6); interleukins ( IL15, IL1B, IL1RN, IL6, IL8); and growth factor genes (TNC, VEGFA, ESM1, MP2). The “Uzonyi Response to Leukotriene and Thrombin” gene set is perturbed by the same number of pathogens as “Hinata NFKB Targets Keratinocyte up”. It contains 37 genes that were up-regulated in Human Umbilical Vein Endothelial Cells (HUVEC) after stimulation with leukotriene LTD4, a leukocyte produced at sites of inflammation [26]. The fourth gene set is “Mahadevan Response to MP470 up”, which is perturbed by 30 pathogens and appeared in 53 biclusters. This gene set contains 19 genes that were up-regulated in gastrointestinal stromal tumor cell-line after treatment with protein-kinase inhibitor drug (MP470) [27]. This gene set also contains chemokines and proinflammatory cytokines such as CCL5, CXCL1, CXCL10, CXCL3, CXCL5, CXCL6, IL8, and IL6.

#### Activation of Innate Immunity

In addition to inflammation, innate immunity also involves the activation of anatomical barriers, mechanical removal of antigens, pattern-recognition receptors, complement pathways, and phagocytosis. The “Netpath IL 4 Pathway down” gene set (which contains 90 genes that are supposed to be transcriptionally down-regulated by the activation of IL4 pathway) is among the top ten most perturbed gene sets. It is perturbed by 30 pathogens and is implicated in 59 biclusters. This gene set has a high overlap with three GO biological process namely “Activation of Innate Immune Response”, “Pattern Recognition Receptor Signaling Pathway”, and “Toll-like Receptor Signaling Pathway”. The perturbation of this gene set indicated that in addition to inflammation, other components of the innate immunity process are also perturbed by multiple bacterial pathogens.

#### Maturation of Dendritic Cells

Dendritic cells have the ability to develop from immature antigen-capturing cells to more specialized antigen-presenting cells. The maturation of dendritic cells is a very important aspect of the host response to bacterial infection. This step indicates the stimulation of various cytokines, chemokines, and other co-stimulatory molecules that are necessary for the onset of adaptive immunity [28]. A number of factors drive the maturation of dendritic cells including the type of antigen (e.g., lipopolysaccharide) and the presence of inflammatory cytokines (e.g., IL-1 and TNF-alpha). In our study, we found that the “Lindstedt Dendritic Cell Maturation A” gene set was perturbed by 30 pathogens and implicated in 67 biclusters. This gene set contains 54 genes that were up-regulated in a transcriptional study involving stimulation of human monocyte-derived dendritic cells with inflammatory stimuli, consisting of tumor necrosis factor (TNF)-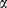 and IL-1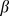 [29].

#### Induction and Inhibition of Apoptosis

Induction and inhibition of apoptosis are important mechanisms of bacterial pathogenesis [22]. The “Dirmeier LMP1 Response Early” gene set, which has a high overlap with GO's “Apoptosis” (GO:0006915) and “Programmed Cell Death” (GO:0012501) biological processes is the second most highly perturbed gene set across the significant biclusters. It is perturbed by 32 pathogens spanning 76 biclusters. This gene set contains 54 genes that are dysregulated in B lymphocyte cells after induction of LMP1, an oncogene. This gene set contains both pro- and antiapoptotic genes whose balance permitted survival of B lymphocyte cells [30]. Perturbation of the “Dirmeier LMP1 Response Early” gene set by most of the pathogens we analyzed indicated that genes with opposing activities involved in cell survival were up-regulated during bacterial infection. This gene set contains tumor suppressors (KLF6, TNFAIP3), oncogenes (BIRC3, CXCR7, HERPUD1, HSP90AB1, LCP1, MYC, NFKB2), cell differentiation markers (CD69, CD83, ICAM1, SLAMF1), and growth markers (LTA, NPPB, TNFSF9).

#### Response to Lipopolysaccharide Stimulation

The host responds in a variety of ways against internal or external stimuli. An example of an external stimulus is a lipopolysaccharide (LPS). LPS is a molecule found on the outer membrane of Gram-negative bacteria. It triggers the expression of a number of signaling molecules, pro-inflammatory cytokines, and antibacterial genes when interacting with the Toll-like receptor of the host cell [31]. The “Seki Inflammatory Response LPS up” gene set [32], [33] contains genes that were up regulated in hepatic stellate cells of the mouse after stimulation with bacterial LPS. This gene set is up-regulated in as many as 83 biclusters (similar to “Zhang Response to IKK Inhibitor and TNF up” gene set) indicating that, genes related to LPS stimulation are predominantly perturbed across a significant number of Gram-negative pathogens. Previous studies have shown that LPS and Gram-negative bacteria such as *Salmonella* elicit identical patterns of gene regulation in macrophages [34], [35].

**Figure 2:**
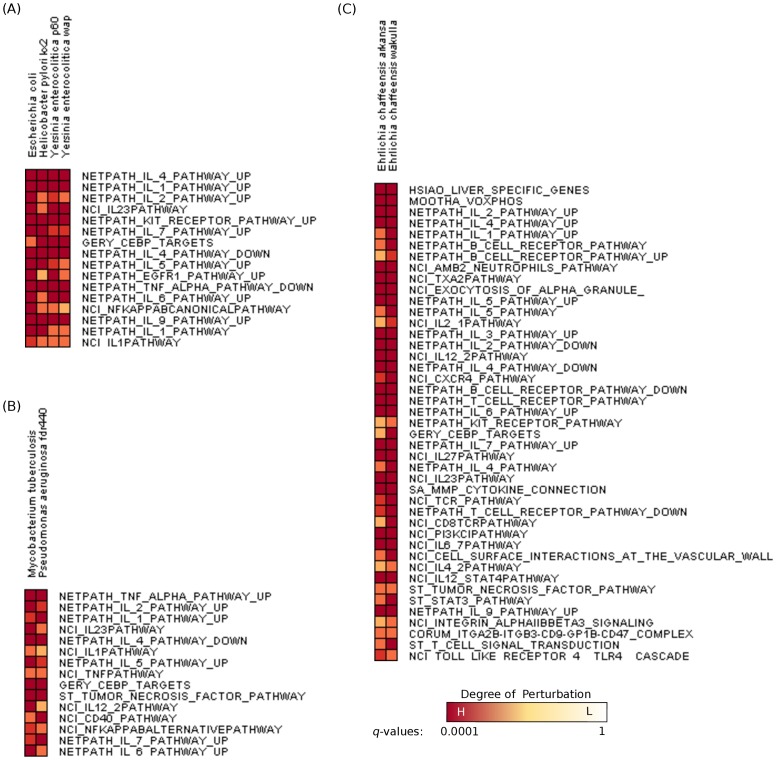
Pathogens that perturb the “Seki Inflammatory Response LPS up” gene set. Pathogens that perturb the “Seki Inflammatory Response LPS up” gene set. The second column contains the 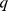-values as well as a color indicating the magnitude of the 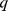-value. Figure 3 contains the legend mapping 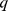-values to colors. All pathogens up-regulate this gene set, except *Streptococcus gordonii*, which down-regulates it.

We expected this gene set would be perturbed only by Gram-negative bacteria, as LPS is a characteristic of these bacteria [31]. However, we observed that 30% of the pathogens that up-regulated this gene set were Gram-positive. Figure 2 shows 20 distinct pathogens (without counting strains of the same pathogen) that up-regulated the “Seki Inflammatory Response LPS up” gene set. Six of these pathogens are Gram-positive, namely *Streptococcus pneumoniae*, *Listeria monocytogenes*, *Bifidobacterium bifidum*, *Streptococcus pyogenes*, *Lactobacillus acidophilus*, and *Bacillus anthracis*. We noted that this gene set has a significant overlap with genes annotated with the biological process “Response to External Stimulus” (GO:0009605). This biological process represents the cells's response to external stimuli. Of the 83 genes annotated to this GO term, 14 genes also belong to “Seki Inflammatory Response LPS up” gene set (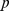-value 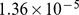). This high degree of overlap suggests that many genes that respond to LPS may belong to a broader class of genes that are perturbed by any external stimulus, including a pathogenic bacterium. This possibility may explain our finding that many Gram-positive bacteria perturb the gene set “Seki Inflammatory Response LPS up”.

### Putative HOBS Drug Targets

We now turn our attention to discovering potential HOBS drug targets in our biclusters. To this end, we further filtered the 84 significant biclusters based on the type of infection caused by the pathogens they contained. Table 3 shows biclusters that contained pathogens that cause an infection in a single type of tissue. We identified seven such biclusters: five gastrointestinal, one respiratory, and one hematopoietic. We selected the most statistically significant bicluster from each category for discussion in this paper.

**Table 3:** Biclusters divided by kind of infection.

Pathogens	Bicluster 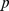 -value	# Gene Sets	# Targets	Target Enrich. ( 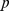 -value)
**Gastrointestinal**				
*Yersinia Enterocolitica* wap and p60 strains, *Helicobacter Pylori*, and *Escherichia Coli*	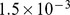	227	18	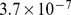
*Yersinia Enterocolitica*, *Lactobacillus Acidophilus*, *Listeria Monocytogenes*, and *Helicobacter Pylori*	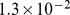	173	11	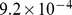
*Yersinia Enterocolitica* and *Helicobacter Pylori*	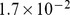	272	21	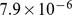
*Yersinia Enterocolitica*, *Listeria Monocytogenes*, and *Bifidobacterium Bifidum*	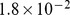	269	17	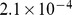
*Yersinia Enterocolitica*, *Bifidobacterium Bifidum*, *Streptococcus Pyogenes*, and *Helicobacter Pylori*	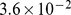	101	6	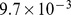
**Respiratory**				
*Pseudomonas Aeruginosa*, and *Mycobacterium Tuberculosis*	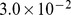	245	16	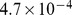
**Hematopoietic**				
*Ehrlichia Chaffeensis*; Strains: arkansa and wakulla	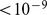	979	186	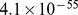

The table shows the biclusters that contained pathogens that cause an infection in a single type of tissue. The columns from left to right are: (i) list of pathogens contained in a bicluster, (ii) a 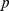-value indicating the statistical significance of the bicluster, (iii) the number of gene sets in the bicluster, (iv) the number of known human drug target genes/proteins in the bicluster, and (v) 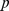-value indicating the enrichment of the bicluster in know human drug-target genes/proteins.

#### Gastrointestinal Pathogens

**Figure 3:**
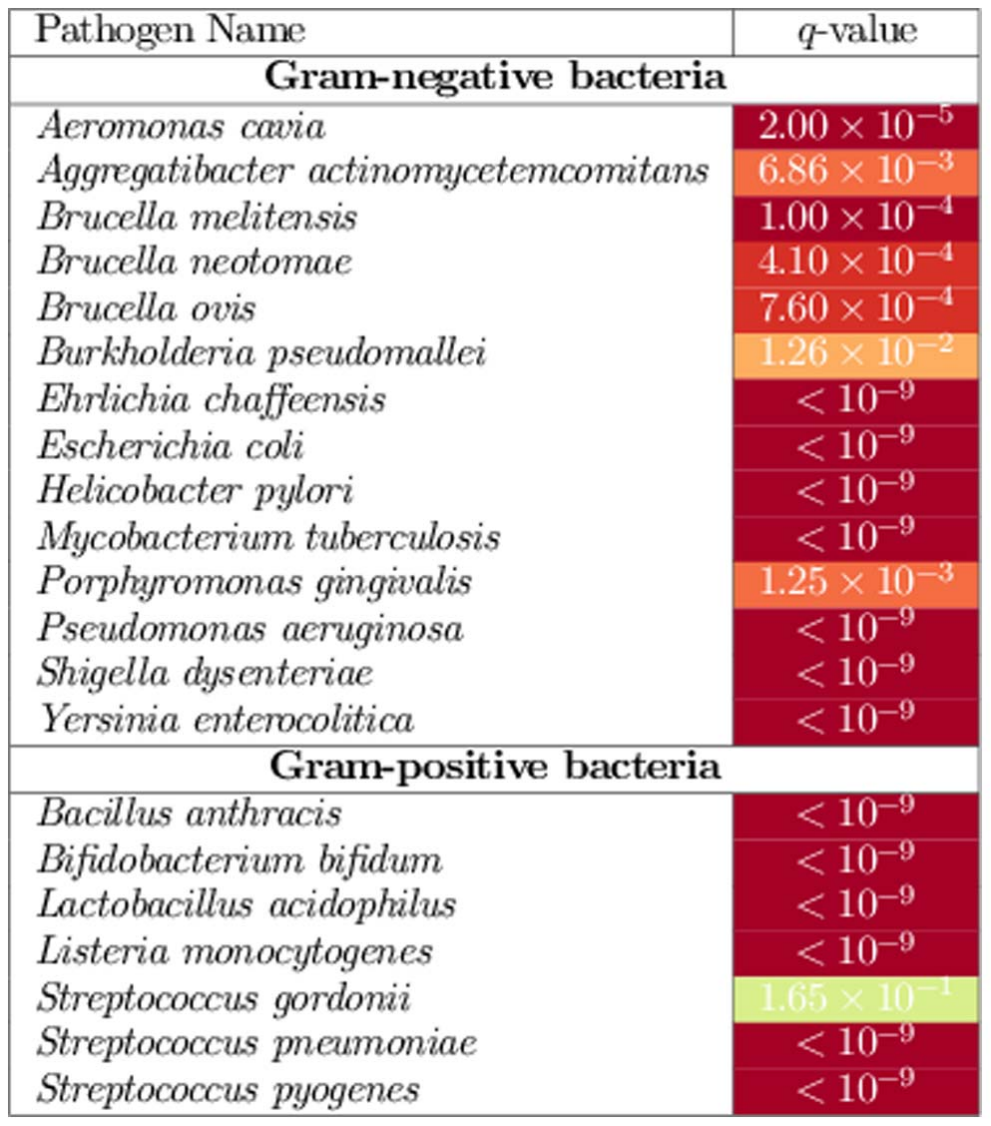
Dendrogram of hierarchical clustering of gene sets for three tissue-specific biclusters. Dendrogram of hierarchical clustering of gene sets for three tissue-specific biclusters. (A) *Yersinia enterocolitica* wap and p60 strains, *Helicobacter pylori* kx2 strain, and enterohemorrhagic *Escherichia coli*. (B) *Pseudomonas aeruginosa* and *Mycobacterium tuberculosis*. (C) *E.chaffeensis* Arkansa and Wakulla strains. The figure only shows gene sets that contain one or more known human drug targets.

Bicluster 38 consisting of the Gram-negative pathogens *Yersinia enterocolitica* wap and p60 strains, *Helicobacter pylori* kx2 strain, and enterohemorrhagic *Escherichia coli* is the bicluster most enriched with gastrointestinal pathogens (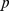-value 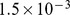). *Yersinia enterocolitica* causes a broad range of gastrointestinal syndromes ranging from acute diarrhea, terminal ileitis, mesenteric lymphadenitis, and pseudoappendicitis [36]. *Helicobacter pylori* kx2 strain is responsible for causing gastric adenocarcinoma [37]. Enterohemorrhagic *Escherichia coli* causes diarrhea or hemorrhagic colitis in humans [38]. The four pathogens jointly up-regulate 227 gene sets (Figure 3A shows the gene sets in this bicluster that contain drug targets). There are 18 known drug targets in this bicluster (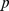-value 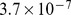). Below we will discuss the drug targets IL1R1 and TNF, which are both primary pro-inflammatory cytokines.

Interleukin-1 type 1 receptor (IL-1R1) is a target molecule for the drug Anakinra (DrugBank ID DB00026). Anakinra is designed to treat rheumatoid arthritis by competitively binding to IL-1R1 thereby inhibiting the action of elevated levels of the pro-inflammatory cytokine IL-1. Previous studies have shown that *Yersinia enterocolitica*, *Helicobacter pylori* kx2 strain, and Enterohemorrhagic *Escherichia coli* induce chronic inflammation [37], [39], [40]. These observations suggest the potential use of drugs that suppress elevated levels of IL-1, such as Anakinra, in the treatment of gastrointestinal infections caused by these four pathogens. Another pro-inflammatory molecule produced by cells infected with bacteria is TNF-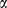, which can cause TNF-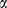-induced apoptosis. TNF-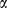 has been implicated as a target molecule for a number of FDA-approved drugs. Etanercept (DrugBank ID: DB00005) and Infliximab (DrugBank ID: DB00065) are TNF-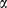 blockers. Anti-TNF therapies have shown to be effective in the treatment of Crohn's disease and ulcerative colitis, which are both disease of the gastrointestinal tract that are characterized by inflammation [41], [42]. Although we did not find supporting evidence on the use of these drugs in the treatment of infections caused by *Yersinia enterocolitica*, *Helicobacter pylori* kx2 strain, and Enterohemorrhagic *Escherichia coli*, the potential use of TNF-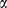 blockers such as Etanercept and Infliximab in the treatment of infection caused by these four pathogens may be worth investigating.

#### Respiratory Pathogens

Bicluster 72 is enriched with respiratory pathogens (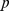-value 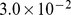). It contains the pathogens *Pseudomonas aeruginosa* and *Mycobacterium tuberculosis*. *Pseudomonas aeruginosa* causes major infections in immunocompromised patients. It is also a leading cause of hospital-acquired infections such as pneumonia [43]. *Mycobacterium tuberculosis* is a causative agent of tuberculosis. The two pathogens jointly perturb 245 gene sets including the IL-12 and IL-23 pathways (Figure 3B shows the gene sets in this bicluster that contain drug targets). The role of IL-12 induction in the treatment of *M. tuberculosis* has been reported in previous studies. For instance, Lowrie *et al.* have shown that up-regulation of IL-12 suppressed proliferation of *M.tuberculosis* in mice [44]. They further suggested the inclusion of this cytokine in tuberculosis vaccines. IL-12 plays a significant role in the host response against *P.aeruginosa*. It is an important molecule in the generation of IFN-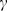 and TNF-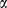, which are essential to promote bacterial clearance. Up-regulation of IL-12 by the host cell is a common strategy used by the host to fight infections caused by these two pathogens. Boosting the level of this molecule when needed, e.g., in immunocompromised patients, might be a viable strategy to treat infection caused by *Pseudomonas aeruginosa* and *Mycobacterium tuberculosis*. Studies suggest that *Pseudomonas aeruginosa* up-regulates IL-23 thereby creating airway inflammation in the host. Dubin *et al.* [45] suggested the suppression of IL-23 as a potential avenue for immunotherapy to infection with this pathogen. Another study indicated that IL-23 is not required by the host to control *Mycobacterium tuberculosis* infection [46] indicating that the down-regulation of IL-23 may not disrupt the host defense mechanism during *M.tuberculosis* infection. Therefore, we suggest that down-regulating IL-23 might be a common strategy to treat infection caused by *Pseudomonas aeruginosa* and *Mycobacterium tuberculosis*.

#### Hematopoietic Pathogens

Bicluster 0 contains two *E.chaffeensis* species, Arkansa and Wakulla. Infection with *Ehrlichia chaffeensis* causes ehrlichiosis, which is characterized by an influenza-like illness, elevation of transaminase levels and sepsis [47]. These two strains commonly up-regulated as many as 979 gene sets, which is not surprising considering the fact that they are different strains of the same bacterial pathogen. However, what is interesting is that the *E.chaffeensis* Liberty strain, which is a part of our study, is not part of this bicluster. This result indicates *E.chaffeensis* Arkansa and *E.chaffeensis* wakulla elicit similar host responses that are different from those perturbed by the Liberty strain. Considering the similarity in the host transcriptional responses, it is tempting to speculate that a common treatment regimen may exist for infection caused by the strains Arkansa and Wakulla.

Among the commonly up-regulated gene sets, “Hsiao Liver Specific Genes” contains the highest number of known drug targets (Figure 3C shows the gene sets in this bicluster that contain drug targets). There are 49 known drug-target proteins in this gene set alone. The “Hsiao Liver Specific Genes” gene set determined by Hsiao *et al.* [48] contains 255 genes that are selectively expressed in the human liver in a gene expression profiling study that involved 59 human samples of 19 different tissue types. The genes in “Hsiao Liver Specific Genes” genes are annotated with liver-specific function including blood coagulation (GO:0007596) and homeostasis (GO:0007599). The up-regulation of the “Hsiao Liver Specific Genes” gene sets by by Wakulla and Arkansas (but not by Liberty) might indicate that *E.chaffeensis* Liberty is inactive in the liver.

The liver is an important organ in cholesterol synthesis, regulation, and export to the other cells. The “Hsiao Liver Specific Genes” gene set contains the protein F2, coagulation factor II (thrombin), which is linked to the cholesterol lowering drug Simvastatin (DrugBank ID: DB00641). Simvastatin reduces total and LDL-cholesterol as well as plasma triglycerides and apolipoprotein B. Previous studies have indicated that *E.chaffeensis* requires cholesterol for survival and growth. However, *E.chaffeensis* does not have the genes for synthesizing cholesterol. Instead, it depends on the host cell to acquire this molecule [49]. In another study, treatment of *E.chaffeensis* with cholesterol extraction reagent methyl-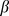-cyclodextrin hampred the ability of this pathogen to infect leukocytes [50]. With this observation in mind, we reasoned that cholesterol lowering drugs such as Simvastatin can be used in the treatment of *E.chaffeensis* infection.

### Known Anti-infective Drug-targets in Biclusters

In the previous section, we attempted to predict HOBS drug targets for three biclusters where the pathogens contained in each bicluster are known to infect similar organs of the human host. In this section, we ranked all statistically significant biclusters based on the number of known anti-infective drug targets that they contain. Identification of such biclusters may be useful to predict other HOBS drug targets in the same bicluster.

To this end, we used the Anatomical Therapeutic Chemical (ATC) Classification from DrugBank and categorized drug targets that are found in statistically significant biclusters as anti-infective or non-anti-infective targets (Table S4). Out of 479 drug targets that are contained in these biclusters, 73 of them are known to be targeted by one or more anti-infective drugs. A functional enrichment analysis of these drug-target genes using DAVID [51] revealed that “response to wounding” (GO:0009611), “inflammatory response” (GO:0006954), “defense response” (GO:0006952), and the KEGG complement and coagulation cascades pathway are among the top five highly enriched biological processes (Table S5). These results shed light on which biological processes in the host are commonly targeted by existing anti-infective drugs.

Bicluster 0 and Bicluster 72 that we discussed in the previous section are the two biclusters that contain the highest number of anti-infective drug targets. Bicluster 0 that contains two strains of *Ehrlichia Chaffeensis*, arkansa and wakulla, has 58 anti-infective drug targets. Bicluster 72 that contains two respiratory pathogens *Pseudomonas aeruginosa* and *Mycobacterium tuberculosis*, has 12 anti-infective targets. It appears that biclusters that had the highest number of anti-infective targets also contained pathogens that are related to one another. This result provided support to our approach of focusing on biclusters that contained pathogens infecting similar organs/tissue of the host.

## Conclusions

In this paper, we have presented a computational approach to identify potential host-oriented broad-spectrum drug targets. Gene set enrichment and biclustering were key ingredients of our method. We combined these two techniques to compute subsets of pathogens that commonly up- or down- regulated sets of biological pathways, gene sets, or protein complexes. We applied this approach on a compendium of gene expression data that represented 38 bacterial pathogens and pathogen strains, from which we identified 84 up-regulated and three down-regulated statistically significant biclusters. Using this approach we were successful in detecting common host responses that are hallmarks of bacterial infections.

Motivated by the premise that diseases that have high degree of transcriptional similarity may be treated with similar drugs [17], we integrated drug target information into our analysis to predict HOBS targets for bacterial infections. Focusing on biclusters that contained pathogens that infected same tissue, we predicted new uses of the drugs Anakinra, Etanercept, and Infliximab for gastrointestinal pathogens *Yersinia enterocolitica*, *Helicobacter pylori* kx2 strain, and enterohemorrhagic *Escherichia coli* and the drug Simvastatin for hematopoietic pathogen *Ehrlichia chaffeensis*.

Broadly, the approach we presented in this paper falls in the realm of integrative DNA microarray data analysis. It can be viewed as an alternative approach to the existing methods developed to discover transcriptional responses common to many diseases [16], [18], [20]. Unlike previous approaches, our method leverages biclustering to detect pathway-specific relationships only among subsets of pathogens.

Our computational approach depends on the identification and targeting of genes whose expression is modulated during host-pathogen interactions. A potential concern with this approach is that it may not distinguish between beneficial host responses and those that may worsen the pathogenecity of the microbe. Dysregulation of a particular biological pathway may not have the same effect on the host under all kinds of infections. For instance, inflammation is often an important host defensive mechanism that may be harmful if uncontrolled.

We computed biclusters that contained groups of biological pathways that are commonly dysregulated by group of pathogens. We acknowledged that a pathway may not be appropriate to target by HOBS drugs simply because a group of pathogens dysregulated that pathway. Accordingly, we used biclustering as a filtering step that would provide potential candidates for HOBS drug targets. In our analysis, we subjected each commonly dysregulated pathway to additional examination, wherein we studied the literature on these pathways and the genes they contained in the context of the pathogens that perturbed them. We used this additional manual step in order to prevent us from proposing an intervention mechanism that would inadvertently block beneficial host responses.

Another difficulty that may arise with our approach is that the number of pathways in a bicluster can sometimes be overwhelming for subsequent analysis. A rational extension to our work is to design methods to prioritize non-redundant biclusters and biological processes based on the similarity of their perturbation. Recent techniques for functional enrichment [52] may be appropriate for this task.

The perturbation of a group of gene sets by a group of pathogens indicates by itself that there might be some underlying similarities in the mechanisms used by the pathogens to infect the host. Therefore, we would ideally like to examine each statistically significant bicluster regardless of whether it contains a drug target or not. The large number of biclusters we computed precluded this detailed analysis. Hence, we chose the strategy of prioritizing biclusters based on drug-target enrichment. The other statistically significant biclusters presented in our supplementary results may also be worthy of further study in the future.

In this study, we analyzed host response data from bacterial infections. In the future, we plan to apply the approach developed here to fungal and viral data sets as well. The results from our studies and related approaches [20] may serve as powerful resources for researchers engaged in host-oriented broad-spectrum drug target discovery.

## Methods

### Gene Expression Datasets

We retrieved 808 distinct taxonomic names of bacterial pathogens from the American Biological Safety Association database of human pathogens. We downloaded the GEO meta database [53] that contains metadata associated with the NCBI's Gene Expression Omnibus (GEO) [54] samples, platforms, and datasets. Next, we queried the meta database using the taxonomic names as keywords. We obtained gene expression datasets for 105 of the 808 bacterial pathogens. Next, we pruned the datasets using the following criteria: (i) We removed time-course data to avoid complications that could arise due to temporal variation of cellular responses to the various pathogens. (ii) We excluded datasets that have less than six samples (infected and healthy samples combined) so that our datasets conform to the recommended sample size for conducting *t*-tests. (iii) We considered DNA microarray data collected from three hosts, namely, *Homo sapiens*, *Mus musclus*, and *Rattus norvegicus*. (iv) We considered experiments that involved the comparison of normal and infected samples. After this process, we retained 29 GEO datasets for subsequent analysis. Details on these datasets are given in Table S1.

### Gene Set Compendium

We built comprehensive functional annotation data sets encompassing biological pathways and functionally associated genes. We integrated data from four sources:

- 1.National Cancer Institute-Nature Pathway Interaction Database (NCI-PID): The NCI-PID contains a collection of curated and peer-reviewed pathways of molecular signaling, regulatory events, and cellular processes [55].
- 2.NetPath: The NetPath database contains cancer and immune signaling pathways, such as the T- and B- cell receptor signaling pathways [56].
- 3.CORUM: The CORUM database houses protein complexes mainly from human, rat, and mouse. A protein complex contains multiple gene products annotated by the same function or localization e.g., respiratory chain protein complex mitochondrial [57].
- 4.The Molecular Signature Database (MsigDB): MsigDB contains genes that are biologically related. This relatedness can be defined by participation in the same biological pathway, chromosomal location, or response to some treatment as evidenced by high-throughput experiments such as gene expression profiling. MsigDB houses four categories of gene sets namely, positional gene sets, curated gene sets, motif gene sets, and computational gene sets. In our analyses we used only curated gene sets.

We collected 449 curated pathways from NCI-PID, 20 curated pathways from the NetPath database, 1,765 protein complexes from the CORUM database, and 3,272 curated gene sets from MsigDB.

### Drugs and Drug Targets Data

We collected 1,652 human drug target proteins from DrugBank [58]. These drug targets were linked to 6,796 therapeutically-validated and experimental drugs.

### Computation of Gene Sets Perturbed in the Host by a Pathogen

We downloaded the raw gene expression profiles (CEL files) from the NCBI's Gene Expression Omnibus (GEO) [54] for the 29 GEO accessions identified above. We normalized the datasets with the Microarray Analysis Suite (MAS5) [59] using the ExpressionFileCreator Module of the GenePattern genomic analysis platform [60]. We ran Gene Set Enrichment Analysis (GSEA) [61] on each gene expression dataset using the compendium of gene sets collected above. We collected the resulting *q*-values (False Discovery Rate or FDR values) into a matrix that indicates the significance of perturbation of each gene set by each pathogen. A *q*-value is the expected probability that GSEA's assessment that a pathogen perturbs a gene set represents a false positive finding. We use a cutoff of 0.2 on *q*-value, which implies that four out of five gene sets that we consider to be perturbed by a pathogen are likely to be true discoveries. As we describe below, we further reduce the possibility of false discoveries in three steps: (i) we compute pathogen-gene set biclusters, (ii) we estimate the statistical significance of each bicluster, and (iii) we compute the enrichment of biclusters in known drug targets. A bicluster associates multiple pathogens with multiple gene sets. Therefore, each gene set in a bicluster is perturbed by more than one pathogen, decreasing the possibility that the perturbation of this gene set is a random occurrence. Furthermore, by estimating the statistical significance of each bicluster, we discard biclusters (and the pathogen-gene set associations that they represent) that could have arisen by random chance. Finally, we filter-out biclusters that are not significantly enriched in known drug targets. This process enabled us to focus on drug-target enriched, non-random, pathogen-gene set associations.

### Biclustering the *q*-value Matrix

Then, we created two binary matrices representing up-regulated and down-regulated biclusters, respectively. In each matrix, each row corresponded to a gene set and each column to a pathogen. An entry in one of these matrices had a value of 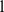 if and only if the GSEA *q*-value for that gene set-pathogen pair was at least 0.2. We applied the BiMax algorithm [62] implemented in the BicAT biclustering analysis toolbox [63] on these matrices to obtain two sets of biclusters, one for up-regulated gene sets and another for down-regulated gene sets.

### Computing the Statistical Significance of Biclusters

We generated 10,000 randomized binary matrices using the swap randomization algorithm [64]. Given a binary matrix 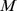 with values 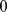 and 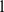, the swap randomization algorithm creates a random matrix 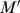 such that each row (respectively, column) of 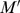 has the same number of 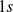 as the corresponding row (respectively, column) of 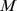. The algorithm achieves this goal through a series of steps that swap row-column pairs. We used our own Perl implementation of this algorithm. We computed biclusters in each of these matrices. We built two sets of distributions reflecting the number of pathogens and the number of genes sets in random biclusters. First, for every integer 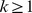, we recorded the number of biclusters that contained 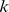 pathogens and at least 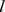 gene sets, for different values of 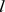. Next, we repeated this process for each integer 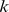, considering the number of gene sets in a bicluster. Now, given a bicluster in the original data containing 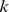 pathogens and 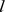 gene sets, we computed two 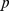-values. One 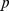-value was the fraction of random biclusters that contained 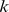 pathogens and at least 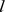 gene sets. The second 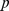-value was the fraction of random biclusters that contained 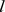 gene sets and at least 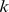 pathogens. These 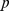-values indicate the probability of observing a bicluster that contains at least a certain number of pathogens or gene sets in the original dataset by chance. We adjusted the 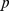-values for multiple hypothesis testing using the method of Benjamini-Hochberg [21]. Finally, we chose the greater of the two 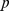-values as a 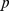-value for each bicluster. We further considered only biclusters with 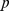-value of at most 0.05.

### Computation of Bicluster Enrichment

We computed the enrichment of each bicluster in various attributes such as the number of known drug targets, host type (human, mouse, and rat), infected cell type (epithelial, dendritic, and macrophage), Gram stain of the pathogen (positive and negative), and infection kind (gastrointestinal, respiratory, oral cavity, and hematopoietic). We used Fisher's exact test for testing the significance of enrichment of a bicluster in each of these attributes.

### Translating Gene Identifiers

Different data sources use different naming schemes for identifying genes . For instance, the molecular signature database uses HUGO symbols while DrugBank uses UniProt namespaces. We used HUGO gene symbols as the common gene identifier in our study. We used the Synergizer service for translating gene/protein's identifiers from other namespaces to HUGO [65].

### Assigning Gene Ontology Biological Processes to a Gene Set

Some of the gene set names in the MsigBD are not self-explanatory, affecting intuitive interpretation of results. In order alleviate this problem, we considered the Gene Ontology biological processes that have the highest overlaps with each respective gene set. To this end, we used the pre-computed overlap/hypergeometric 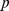-values between a gene set and GO processes that are provided on the MsigDB website. For the “Netpath IL 4 Pathway Down” gene set, we obtained the corresponding GO biological processes using GOrilla [66].

## Supporting Information

Figure S1 **Scatter plot of number of pathogens vs. biclusters.** Plot indicates that number of pathogens perturbing a gene set are positively correlated with the number of biclusters a particular gene set appeared in. Supporting information can also be accessed from our supplementary website: http://bioinformatics.cs.vt.edu/ murali/supplements/2013-kidane-plos-one.
(PDF)

Table S1 **Details of DNA microarray dataset used in the study.** It contains GEO accession numbers, microarray platform used, infected host, and tissue or cell type from which the gene expression measurements were taken.
(HTML)

Table S2
**Up-regulated biclusters.** It contains detail information on all up-regulated biclusters. This include: bicluster ID, list of pathogens and gene sets in bicluster, 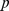-values indicating statistical significance of bicluster and enrichment of these biclusters in various attributes such as drug targets and host type.(HTML)

Table S3
**Down-regulated biclusters.** It contains detail information on all down-regulated biclusters. This include: bicluster ID, list of pathogens and gene sets in bicluster, 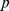-values indicating statistical significance of bicluster and enrichment of these biclusters in various attributes such as drug targets and host type.(HTML)

Table S4 **Known anti-infective targets in biclusters.** It contains bicluster ID, list of all drug targets, and anti-infective targets in bicluster.
(XLS)

Table S5
**Functional annotations of anti-infective targets.** It contains 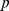-values indicating enrichment of anti-infective drug targets in GO biological processes.(XLS)
