# Supplementary material for: The Landscape of Host Transcriptional Response Programs Commonly Perturbed by Bacterial Pathogens: Towards Host-Oriented Broad-Spectrum Drug Targets
Source: PLoS One. 2013 Mar 13;8(3):e58553. doi: 10.1371/journal.pone.0058553 (PMC3596304; doi:10.1371/journal.pone.0058553)
Supplement: Figure S1 — Scatter plot of number of pathogens vs. biclusters. Plot indicates that number of pathogens perturbing a gene set are positively correlated with the number of biclusters a particular gene set appeared in. Supporting information can also be accessed from our supplementary website: http://bioinformatics.cs.vt.edu/ murali/supplements/2013-kidane-plos-one. (PDF) [file pone.0058553.s001.pdf]

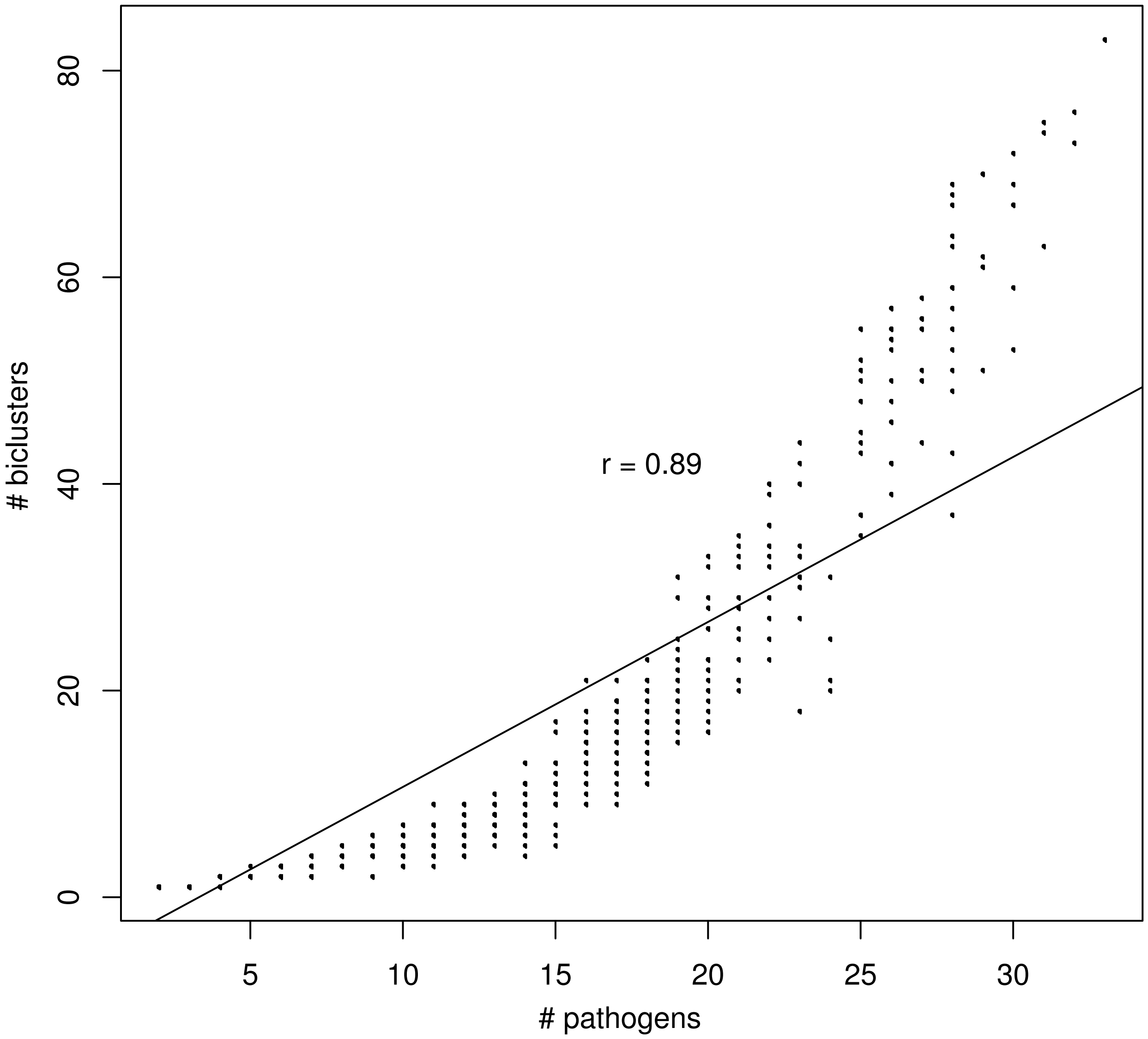

Figure S1. Correlation between number of pathogens that perturb gene set and the number of biclusters that contain the gene set. Plot indicates that number of pathogens perturbing a gene set are positively correlated with the number of biclusters a particular gene set appeared in.
