# Supplementary material for: The Landscape of Host Transcriptional Response Programs Commonly Perturbed by Bacterial Pathogens: Towards Host-Oriented Broad-Spectrum Drug Targets
Source: PLoS One. 2013 Mar 13;8(3):e58553. doi: 10.1371/journal.pone.0058553 (PMC3596304; doi:10.1371/journal.pone.0058553)
Supplement: Table S1 — Details of DNA microarray dataset used in the study. It contains GEO accession numbers, microarray platform used, infected host, and tissue or cell type from which the gene expression measurements were taken. (HTML) [file pone.0058553.s002.html]

**Details of DNA Microarray Datasets Used in the Study** 

|  |  |  |  |  |  |  |
| --- | --- | --- | --- | --- | --- | --- |
| **S/N** | **GEO Acc #** | **Pathogen Name** | **Gram Stain** | **Platform** | **Organism** | **Target Cell/Tissue** |
| 1 | GSE6765 | *Aeromonas caviae (epa72)* | Negative | GPL1261 | *Mus musculus* | Neonatal mouse small intestinal tissue |
| 2 | GSE9723 | *Aggregatibacter actinomycetemcomitans* | Negative | GPL96 | *Homo sapiens* | Human immortalized gingival keratinocyte |
| 3 | GSE2600 | *Anaplasma phagocytophilum* | Negative | GPL570 | *Homo sapiens* | Promyelocytic cells (NB4) |
| 4 | GSE14390 | *Bacillus anthracis (7702)* | Positive | GPL570 | *Homo sapiens* | Human alveolar macrophage |
| 5 | GSE14686 | *Bacteroides thetaiotaomicron* | Negative | GPL1261 | *Mus musculus* | Proximal colon of mouse |
| 6 | GSE20302 | *Bifidobacterium bifidum* | Positive | GPL1261 | *Mus musculus* | Bone marrow-derived dendritic cells (BMDD) |
| 7 | GSE8385 | *Brucella melitensis* | Negative | GPL81 | *Mus musculus* | Macrophage cell lines (RAW 264.7) |
| 8 | GSE8385 | *Brucella neotomae* | Negative | GPL81 | *Mus musculus* | Macrophage cell lines (RAW 264.7) |
| 9 | GSE8385 | *Brucella ovis* | Negative | GPL81 | *Mus musculus* | Macrophage cell lines (RAW 264.7) |
| 10 | GSE7577 | *Burkholderia pseudomallei* | Negative | GPL96 | *Homo sapiens* | Human monocytic macrophage ell lines (THP-1) |
| 11 | GSE6688 | *Chlamydia pneumoniae* | Negative | GPL339 | *Mus musculus* | Lung |
| 12 | GSE8966 | *Ehrlichia chaffeensis (arkansas, wakulla, liberty)* | Negative | GPL1261 | *Mus musculus* | Liver |
| 13 | GSE19315 | *Escherichia coli* | Negative | GPL570 | *Homo sapiens* | Human monocytic macrophage ell lines (THP-1) cells |
| 14 | GSE14686 | *Eubacterium rectale* | Positive | GPL1261 | *Mus musculus* | Proximal colon of mouse |
| 15 | GSE6927 | *Fusobacterium nucleatum* | Negative | GPL96 | *Homo sapiens* | Human immortalized gingival keratinocytes cells (HIGC) |
| 16 | GSE10262 | *Helicobacter pylori (kx1, kx2)* | Negative | GPL1261 | *Mus musculus* | Gastric epithelial progenitor and non-progenitor cells |
| 17 | GSE581 | *Helicobacter pylori* | Negative | GPL193 | *Homo sapiens* | Gastric biopsies |
| 18 | GSE20302 | *Lactobacillus acidophilus (ncfm)* | Positive | GPL1261 | *Mus musculus* | Bone marrow-derived dendritic cells (BMDD) |
| 19 | GSE9946 | *Listeria monocytogenes* | Positive | GPL96 | *Homo sapiens* | Monocyte-derived dendritic cells |
| 20 | GSE17477 | *Mycobacterium tuberculosis* | none | GPL571 | *Homo sapiens* | Human monocytic macrophage ell lines (THP-1) |
| 21 | GSE9723 | *Porphyromonas gingivalis* | Negative | GPL96 | *Homo sapiens* | Human immortalized gingival keratinocytes cells(HIGC) |
| 22 | GSE1469 | *Pseudomonas aeruginosa (pak)* | Negative | GPL91 | *Homo sapiens* | Lung pneumocytes cell line (A549) |
| 23 | GSE923 | *Pseudomonas aeruginosa (fdr1234, fdr875,fdr1, fdr440)* | Negative | GPL96 | *Homo sapiens* | Calu-3 human lung epithelial cells |
| 24 | GSE19315 | *Shigella dysenteriae* | Negative | GPL570 | *Homo sapiens* | Human monocytic macrophage ell lines (THP-1) cells |
| 25 | GSE6802 | *Staphylococcus aureus* | Positive | GPL571 | *Homo sapiens* | Bronchial epithelial cells beas-2b |
| 26 | GSE6927 | *Streptococcus gordonii* | Positive | GPL96 | *Homo sapiens* | Human immortalized gingival keratinocytes cells (HIGC) |
| 27 | GSE8527 | *Streptococcus pneumoniae (d39, g54, tigr4)* | Positive | GPL570 | *Homo sapiens* | Pharyngeal epithelial cell lines (Detroit 562) |
| 28 | GSE11494 | *Streptococcus pyogenes (90-226)* | Positive | GPL1261 | *Mus musculus* | Nasal-associated lymphoid tissue (NALT) |
| 29 | GSE2973 | *Yersinia enterocolitica (wap, p60)* | Negative | GPL339 | *Mus musculus* | Bone marrow-derived macrophages (BMDM) BALB/C and C57BL/6 |
