# Supplementary material for: The Landscape of Host Transcriptional Response Programs Commonly Perturbed by Bacterial Pathogens: Towards Host-Oriented Broad-Spectrum Drug Targets
Source: PLoS One. 2013 Mar 13;8(3):e58553. doi: 10.1371/journal.pone.0058553 (PMC3596304; doi:10.1371/journal.pone.0058553)
Supplement: Table S2 — Up-regulated biclusters. It contains detail information on all up-regulated biclusters. This include: bicluster ID, list of pathogens and gene sets in bicluster, -values indicating statistical significance of bicluster and enrichment of these biclusters in various attributes such as drug targets and host type. (HTML) [file pone.0058553.s003.html]

**Summary of Up-regulated Biclusters and their Enrichment in Various Attributes** 

*Click on the "Bicluster ID" to view details. Statistically significant biclusters are highlighted in green*

|  |  |  |  |  |  |  |  |  |  |  |  |  |  |  |  |  |  |
| --- | --- | --- | --- | --- | --- | --- | --- | --- | --- | --- | --- | --- | --- | --- | --- | --- | --- |
|  | | | | | | | | | | | | | | | | | |
| **Bicluster ID** | **# Pathogens** | **# Genesets** | **List of Pathogens** | **Bicluster p-value** | **# Targets** | **Target Enrich. (p-value)** | ***Mus musclus Enrich. (p-value)*** | ***Homo sapiens Enrich. (p-value)*** | **Grm. Pos. Enrich. (p-value)** | **Grm. Neg. Enrich. (p-value)** | **Epithelial Enrich. (p-value)** | **Dendritic Enrich. (p-value)** | **Macrophage Enrch. (p-value)** | **GI Enrich. (p-value)** | **Resp. Enrich. (p-value)** | **Oral Cavity Enrich. (p-value)** | **Hemato. Enrich. (p-value)** |
| 0 | 2 | 979 | *Ehrlichia chaffeensis arkansa Ehrlichia chaffeensis wakulla* | < 5.41E-09 | 186 | < 3.71E-07 | 1.00E+00 | 2.44E-01 | 1.00E+00 | 5.05E-01 | 1.00E+00 | 1.00E+00 | 1.00E+00 | 1.00E+00 | 1.00E+00 | 1.00E+00 | 4.18E-02 |
| 2 | 9 | 205 | *Yersinia enterocolitica wap bl6 Yersinia enterocolitica wap bc Yersinia enterocolitica p60 bl6 Yersinia enterocolitica p60 bc Listeria monocytogenes Ehrlichia chaffeensis arkansa Ehrlichia chaffeensis wakulla Mycobacterium tuberculosis Escherichia coli* | < 5.41E-09 | 5 | 7.15E-02 | 9.35E-01 | 2.27E-01 | 9.55E-01 | 4.91E-01 | 1.00E+00 | 5.25E-01 | 9.22E-03 | 3.83E-02 | 9.55E-01 | 1.00E+00 | 6.33E-01 |
| 4 | 8 | 162 | *Yersinia enterocolitica wap bc Helicobacter pylori kx1 mgep Pseudomonas aeruginosa Ehrlichia chaffeensis wakulla Pseudomonas aeruginosa fdr440 Mycobacterium tuberculosis Helicobacter pylori kx2 mgep Escherichia coli* | < 5.41E-09 | 5 | 2.60E-02 | 6.52E-01 | 6.52E-01 | 1.00E+00 | 2.57E-01 | 6.64E-01 | 1.00E+00 | 4.11E-01 | 2.94E-01 | 3.45E-01 | 1.00E+00 | 8.82E-01 |
| 6 | 2 | 644 | *Listeria monocytogenes Mycobacterium tuberculosis* | 1.00E+000 | 63 | < 3.71E-07 | 2.44E-01 | 1.00E+00 | 4.60E-01 | 1.00E+00 | 1.00E+00 | 1.39E-01 | 4.95E-01 | 5.92E-01 | 4.60E-01 | 1.00E+00 | 1.00E+00 |
| 8 | 2 | 621 | *Yersinia enterocolitica wap bc Yersinia enterocolitica p60 bc* | 1.00E+000 | 71 | < 3.71E-07 | 1.00E+00 | 2.44E-01 | 1.00E+00 | 5.05E-01 | 1.00E+00 | 1.00E+00 | 7.67E-02 | 1.22E-01 | 1.00E+00 | 1.00E+00 | 1.00E+00 |
| 10 | 4 | 305 | *Ehrlichia chaffeensis wakulla Lactobacillus acidophilus Bifidobacterium bifidum Escherichia coli* | 7.43E-002 | 16 | 3.40E-04 | 9.47E-01 | 3.03E-01 | 2.77E-01 | 9.37E-01 | 1.00E+00 | 2.02E-02 | 7.55E-01 | 8.43E-01 | 1.00E+00 | 1.00E+00 | 6.34E-01 |
| 12 | 8 | 145 | *Yersinia enterocolitica wap bc Helicobacter pylori kx1 mgep Yersinia enterocolitica p60 bl6 Yersinia enterocolitica p60 bc Listeria monocytogenes streptococcus pneumoniae tigr4 Helicobacter pylori kx2 mgep Shigella dysenteriae* | < 5.41E-09 | 4 | 1.06E-01 | 8.81E-01 | 3.48E-01 | 6.88E-01 | 5.89E-01 | 6.64E-01 | 4.79E-01 | 1.46E-01 | 1.53E-03 | 9.33E-01 | 1.00E+00 | 1.00E+00 |
| 14 | 2 | 575 | *Ehrlichia chaffeensis wakulla streptococcus pneumoniae g54* | 1.00E+000 | 53 | < 3.71E-07 | 7.56E-01 | 7.56E-01 | 4.60E-01 | 9.23E-01 | 6.23E-01 | 1.00E+00 | 1.00E+00 | 1.00E+00 | 4.60E-01 | 1.00E+00 | 3.87E-01 |
| 16 | 3 | 369 | *Ehrlichia chaffeensis wakulla Mycobacterium tuberculosis Ehrlichia chaffeensis liberty* | 1.00E+000 | 27 | < 3.71E-07 | 8.84E-01 | 5.00E-01 | 1.00E+00 | 8.08E-01 | 1.00E+00 | 1.00E+00 | 6.46E-01 | 1.00E+00 | 6.08E-01 | 1.00E+00 | 1.11E-01 |
| 18 | 7 | 155 | *Yersinia enterocolitica wap bl6 Yersinia enterocolitica wap bc Yersinia enterocolitica p60 bl6 Yersinia enterocolitica p60 bc Lactobacillus acidophilus Bifidobacterium bifidum Pseudomonas aeruginosa* | < 5.41E-09 | 9 | 1.63E-03 | 9.96E-01 | 4.66E-02 | 6.02E-01 | 6.88E-01 | 1.00E+00 | 6.71E-02 | 8.83E-02 | 1.92E-01 | 9.03E-01 | 1.00E+00 | 1.00E+00 |
| 20 | 3 | 355 | *streptococcus pneumoniae g54 Mycobacterium tuberculosis Escherichia coli* | 1.00E+000 | 13 | 8.90E-04 | 1.16E-01 | 1.00E+00 | 6.08E-01 | 9.81E-01 | 7.74E-01 | 1.00E+00 | 1.92E-01 | 7.45E-01 | 1.63E-01 | 1.00E+00 | 1.00E+00 |
| 22 | 2 | 524 | *Yersinia enterocolitica p60 bl6 Ehrlichia chaffeensis wakulla* | 1.00E+000 | 82 | < 3.71E-07 | 1.00E+00 | 2.44E-01 | 1.00E+00 | 5.05E-01 | 1.00E+00 | 1.00E+00 | 4.95E-01 | 5.92E-01 | 1.00E+00 | 1.00E+00 | 3.87E-01 |
| 24 | 3 | 340 | *Pseudomonas aeruginosa fdr440 Listeria monocytogenes Escherichia coli* | 1.00E+000 | 21 | 4.37E-03 | 1.16E-01 | 1.00E+00 | 6.08E-01 | 8.08E-01 | 7.74E-01 | 2.04E-01 | 6.46E-01 | 2.87E-01 | 6.08E-01 | 1.00E+00 | 1.00E+00 |
| 26 | 2 | 503 | *Helicobacter pylori kx1 mgep Helicobacter pylori kx2 mgep* | 1.00E+000 | 82 | < 3.71E-07 | 1.00E+00 | 2.44E-01 | 1.00E+00 | 5.05E-01 | 1.39E-01 | 1.00E+00 | 1.00E+00 | 1.22E-01 | 1.00E+00 | 1.00E+00 | 1.00E+00 |
| 28 | 3 | 332 | *Yersinia enterocolitica wap bc streptococcus pneumoniae g54 Listeria monocytogenes* | 1.40E-003 | 13 | 8.00E-05 | 5.00E-01 | 8.84E-01 | 1.63E-01 | 9.81E-01 | 7.74E-01 | 2.04E-01 | 6.46E-01 | 2.87E-01 | 6.08E-01 | 1.00E+00 | 1.00E+00 |
| 30 | 7 | 141 | *Yersinia enterocolitica wap bc Ehrlichia chaffeensis arkansa Yersinia enterocolitica p60 bc Ehrlichia chaffeensis wakulla Lactobacillus acidophilus streptococcus pyogenes Ehrlichia chaffeensis liberty* | 1.40E-004 | 6 | 7.19E-02 | 1.00E+00 | 4.31E-03 | 6.02E-01 | 6.88E-01 | 1.00E+00 | 4.30E-01 | 6.60E-01 | 8.03E-01 | 1.00E+00 | 1.00E+00 | 1.55E-01 |
| 32 | 7 | 137 | *Yersinia enterocolitica wap bc Ehrlichia chaffeensis arkansa Ehrlichia chaffeensis wakulla streptococcus pneumoniae g54 Mycobacterium tuberculosis streptococcus pneumoniae tigr4 Shigella dysenteriae* | 3.63E-004 |  | 1.00E+00 | 5.00E-01 | 7.95E-01 | 6.02E-01 | 9.12E-01 | 8.39E-01 | 1.00E+00 | 3.12E-01 | 8.03E-01 | 2.56E-01 | 1.00E+00 | 4.72E-01 |
| 34 | 3 | 314 | *Yersinia enterocolitica p60 bl6 Yersinia enterocolitica p60 bc streptococcus pneumoniae g54* | 6.43E-003 | 44 | < 3.71E-07 | 8.84E-01 | 5.00E-01 | 6.08E-01 | 8.08E-01 | 7.74E-01 | 1.00E+00 | 1.92E-01 | 2.87E-01 | 6.08E-01 | 1.00E+00 | 1.00E+00 |
| 36 | 3 | 306 | *Ehrlichia chaffeensis arkansa Bifidobacterium bifidum Mycobacterium tuberculosis* | 7.76E-003 | 19 | 1.00E-05 | 8.84E-01 | 5.00E-01 | 6.08E-01 | 9.81E-01 | 1.00E+00 | 2.04E-01 | 6.46E-01 | 1.00E+00 | 6.08E-01 | 1.00E+00 | 5.25E-01 |
| 38 | 4 | 227 | *Yersinia enterocolitica wap bl6 Yersinia enterocolitica p60 bl6 Helicobacter pylori kx2 mgep Escherichia coli* | 1.50E-003 | 18 | 3.71E-07 | 9.47E-01 | 3.03E-01 | 1.00E+00 | 2.45E-01 | 8.66E-01 | 1.00E+00 | 6.34E-02 | 1.22E-02 | 1.00E+00 | 1.00E+00 | 1.00E+00 |
| 40 | 4 | 222 | *Yersinia enterocolitica p60 bc Ehrlichia chaffeensis wakulla Shigella dysenteriae Escherichia coli* | 1.87E-003 | 13 | 1.10E-04 | 6.97E-01 | 6.97E-01 | 1.00E+00 | 2.45E-01 | 1.00E+00 | 1.00E+00 | 6.34E-02 | 1.22E-01 | 1.00E+00 | 1.00E+00 | 6.34E-01 |
| 42 | 3 | 295 | *Yersinia enterocolitica wap bc Yersinia enterocolitica p60 bl6 Pseudomonas aeruginosa fdr440* | 9.90E-003 | 24 | < 3.71E-07 | 8.84E-01 | 5.00E-01 | 1.00E+00 | 3.54E-01 | 7.74E-01 | 1.00E+00 | 1.92E-01 | 2.87E-01 | 6.08E-01 | 1.00E+00 | 1.00E+00 |
| 44 | 8 | 110 | *Yersinia enterocolitica wap bl6 Helicobacter pylori kx1 mgep Yersinia enterocolitica p60 bl6 Listeria monocytogenes Bifidobacterium bifidum Ehrlichia chaffeensis liberty Ehrlichia chaffeensis wakulla Pseudomonas aeruginosa fdr440* | 6.04E-005 |  | 1.00E+00 | 9.78E-01 | 1.19E-01 | 6.88E-01 | 5.89E-01 | 8.98E-01 | 8.78E-02 | 7.43E-01 | 2.94E-01 | 9.33E-01 | 1.00E+00 | 5.57E-01 |
| 46 | 4 | 219 | *Ehrlichia chaffeensis arkansa Ehrlichia chaffeensis wakulla Listeria monocytogenes Pseudomonas aeruginosa* | 2.13E-003 | 13 | 6.10E-04 | 6.97E-01 | 6.97E-01 | 7.19E-01 | 6.80E-01 | 1.00E+00 | 2.65E-01 | 1.00E+00 | 8.43E-01 | 7.19E-01 | 1.00E+00 | 1.96E-01 |
| 48 | 13 | 64 | *Yersinia enterocolitica wap bc Yersinia enterocolitica p60 bc Lactobacillus acidophilus Listeria monocytogenes Bifidobacterium bifidum Brucella ovis Ehrlichia chaffeensis arkansa Ehrlichia chaffeensis wakulla Pseudomonas aeruginosa fdr440 Brucella melitensis Mycobacterium tuberculosis Brucella neotomae Escherichia coli* | < 5.41E-09 | 2 | 6.40E-02 | 9.78E-01 | 9.05E-02 | 7.49E-01 | 7.23E-01 | 1.00E+00 | 2.49E-02 | 2.14E-02 | 7.85E-01 | 9.31E-01 | 1.00E+00 | 8.43E-02 |
| 50 | 3 | 272 | *Yersinia enterocolitica wap bl6 Yersinia enterocolitica wap bc Helicobacter pylori kx1 mgep* | 1.65E-002 | 21 | 7.93E-06 | 1.00E+00 | 1.16E-01 | 1.00E+00 | 3.54E-01 | 7.74E-01 | 1.00E+00 | 1.92E-01 | 3.96E-02 | 1.00E+00 | 1.00E+00 | 1.00E+00 |
| 52 | 2 | 404 | *Pseudomonas aeruginosa Escherichia coli* | 6.67E-002 | 44 | < 3.71E-07 | 2.44E-01 | 1.00E+00 | 1.00E+00 | 5.05E-01 | 1.00E+00 | 1.00E+00 | 4.95E-01 | 5.92E-01 | 4.60E-01 | 1.00E+00 | 1.00E+00 |
| 54 | 3 | 269 | *Helicobacter pylori kx1 mgep Mycobacterium tuberculosis Shigella dysenteriae* | 1.78E-002 | 18 | 7.00E-04 | 5.00E-01 | 8.84E-01 | 1.00E+00 | 8.08E-01 | 7.74E-01 | 1.00E+00 | 1.92E-01 | 2.87E-01 | 6.08E-01 | 1.00E+00 | 1.00E+00 |
| 56 | 3 | 269 | *Yersinia enterocolitica p60 bc Listeria monocytogenes Bifidobacterium bifidum* | 1.78E-002 | 17 | 2.10E-04 | 8.84E-01 | 5.00E-01 | 1.63E-01 | 9.81E-01 | 1.00E+00 | 1.03E-02 | 6.46E-01 | 2.87E-01 | 1.00E+00 | 1.00E+00 | 1.00E+00 |
| 58 | 7 | 115 | *Yersinia enterocolitica wap bl6 Yersinia enterocolitica p60 bc Pseudomonas aeruginosa fdr440 streptococcus pneumoniae g54 Listeria monocytogenes Mycobacterium tuberculosis Helicobacter pylori kx2 mgep* | 1.20E-005 | 3 | 2.68E-02 | 5.00E-01 | 7.95E-01 | 6.02E-01 | 9.12E-01 | 5.46E-01 | 4.30E-01 | 3.12E-01 | 1.92E-01 | 2.56E-01 | 1.00E+00 | 1.00E+00 |
| 60 | 3 | 265 | *Helicobacter pylori kx1 mgep Ehrlichia chaffeensis arkansa Escherichia coli* | 1.97E-002 | 21 | < 3.71E-07 | 8.84E-01 | 5.00E-01 | 1.00E+00 | 3.54E-01 | 7.74E-01 | 1.00E+00 | 6.46E-01 | 2.87E-01 | 1.00E+00 | 1.00E+00 | 5.25E-01 |
| 62 | 3 | 263 | *streptococcus pyogenes Mycobacterium tuberculosis Escherichia coli* | 2.07E-002 | 33 | < 3.71E-07 | 5.00E-01 | 8.84E-01 | 6.08E-01 | 9.81E-01 | 1.00E+00 | 1.00E+00 | 1.92E-01 | 7.45E-01 | 6.08E-01 | 1.00E+00 | 1.00E+00 |
| 64 | 3 | 255 | *Ehrlichia chaffeensis arkansa Yersinia enterocolitica p60 bc Pseudomonas aeruginosa fdr440* | 2.48E-002 | 11 | 2.02E-03 | 8.84E-01 | 5.00E-01 | 1.00E+00 | 3.54E-01 | 7.74E-01 | 1.00E+00 | 6.46E-01 | 7.45E-01 | 6.08E-01 | 1.00E+00 | 5.25E-01 |
| 66 | 7 | 109 | *Yersinia enterocolitica wap bl6 Ehrlichia chaffeensis arkansa Yersinia enterocolitica p60 bl6 Yersinia enterocolitica p60 bc Bifidobacterium bifidum Aeromonas cavia Escherichia coli* | 4.48E-005 | 1 | 8.25E-01 | 9.96E-01 | 4.66E-02 | 9.03E-01 | 3.40E-01 | 1.00E+00 | 4.30E-01 | 8.83E-02 | 4.43E-02 | 1.00E+00 | 1.00E+00 | 8.42E-01 |
| 68 | 8 | 94 | *Yersinia enterocolitica wap bl6 Helicobacter pylori kx1 mgep Yersinia enterocolitica p60 bc Lactobacillus acidophilus Ehrlichia chaffeensis arkansa Ehrlichia chaffeensis wakulla Bacillus anthracis Helicobacter pylori kx2 mgep* | 1.02E-006 | 3 | 1.18E-01 | 9.98E-01 | 2.24E-02 | 6.88E-01 | 5.89E-01 | 8.98E-01 | 4.79E-01 | 4.11E-01 | 2.94E-01 | 1.00E+00 | 1.00E+00 | 2.19E-01 |
| 70 | 8 | 92 | *Yersinia enterocolitica wap bl6 Yersinia enterocolitica wap bc Listeria monocytogenes Pseudomonas aeruginosa Bacillus anthracis Mycobacterium tuberculosis Escherichia coli Shigella dysenteriae* | 2.71E-006 | 4 | 2.20E-02 | 1.19E-01 | 9.78E-01 | 6.88E-01 | 8.54E-01 | 1.00E+00 | 4.79E-01 | 3.61E-03 | 9.08E-02 | 6.88E-01 | 1.00E+00 | 8.82E-01 |
| 72 | 2 | 245 | *Pseudomonas aeruginosa fdr440 Mycobacterium tuberculosis* | 3.03E-002 | 16 | 4.70E-04 | 5.00E-01 | 8.84E-01 | 6.08E-01 | 9.81E-01 | 7.74E-01 | 2.04E-01 | 6.46E-01 | 1.00E+00 | 1.63E-01 | 1.00E+00 | 1.00E+00 |
| 74 | 8 | 89 | *Yersinia enterocolitica wap bc Helicobacter pylori kx1 mgep Lactobacillus acidophilus Bifidobacterium bifidum Pseudomonas aeruginosa fdr440 streptococcus pneumoniae g54 Escherichia coli Shigella dysenteriae* | 1.74E-005 | 2 | 1.20E-01 | 6.52E-01 | 6.52E-01 | 3.45E-01 | 8.54E-01 | 6.64E-01 | 8.78E-02 | 4.11E-01 | 2.94E-01 | 6.88E-01 | 1.00E+00 | 1.00E+00 |
| 76 | 5 | 142 | *Ehrlichia chaffeensis arkansa Ehrlichia chaffeensis wakulla Listeria monocytogenes Mycobacterium tuberculosis Aeromonas cavia* | 3.26E-003 | 7 | 3.89E-02 | 8.28E-01 | 5.00E-01 | 8.00E-01 | 8.69E-01 | 1.00E+00 | 3.23E-01 | 8.32E-01 | 5.96E-01 | 8.00E-01 | 1.00E+00 | 2.88E-01 |
| 78 | 3 | 235 | *streptococcus pneumoniae g54 Helicobacter pylori kx2 mgep Shigella dysenteriae* | 3.69E-002 | 7 | 8.63E-01 | 5.00E-01 | 8.84E-01 | 6.08E-01 | 8.08E-01 | 3.21E-01 | 1.00E+00 | 6.46E-01 | 2.87E-01 | 6.08E-01 | 1.00E+00 | 1.00E+00 |
| 80 | 2 | 350 | *Escherichia coli Ehrlichia chaffeensis liberty* | 1.12E-001 | 30 | < 3.71E-07 | 7.56E-01 | 7.56E-01 | 1.00E+00 | 5.05E-01 | 1.00E+00 | 1.00E+00 | 4.95E-01 | 5.92E-01 | 1.00E+00 | 1.00E+00 | 3.87E-01 |
| 82 | 3 | 233 | *Yersinia enterocolitica p60 bl6 Mycobacterium tuberculosis streptococcus pneumoniae tigr4* | 3.84E-002 | 6 | 2.40E-01 | 5.00E-01 | 8.84E-01 | 6.08E-01 | 9.81E-01 | 7.74E-01 | 1.00E+00 | 1.92E-01 | 7.45E-01 | 1.63E-01 | 1.00E+00 | 1.00E+00 |
| 84 | 3 | 231 | *Helicobacter pylori kx1 mgep Ehrlichia chaffeensis wakulla streptococcus pneumoniae tigr4* | 3.99E-002 | 6 | 1.21E-01 | 8.84E-01 | 5.00E-01 | 6.08E-01 | 8.08E-01 | 3.21E-01 | 1.00E+00 | 1.00E+00 | 7.45E-01 | 6.08E-01 | 1.00E+00 | 5.25E-01 |
| 86 | 7 | 99 | *Yersinia enterocolitica wap bc Helicobacter pylori kx1 mgep Yersinia enterocolitica p60 bl6 Yersinia enterocolitica p60 bc Mycobacterium tuberculosis Helicobacter pylori kx1 npgec Escherichia coli* | 2.20E-004 | 7 | 1.08E-03 | 9.53E-01 | 2.05E-01 | 1.00E+00 | 3.40E-01 | 8.39E-01 | 1.00E+00 | 1.38E-02 | 5.25E-03 | 9.03E-01 | 1.00E+00 | 1.00E+00 |
| 88 | 9 | 77 | *Yersinia enterocolitica wap bl6 Yersinia enterocolitica wap bc Yersinia enterocolitica p60 bl6 Yersinia enterocolitica p60 bc Porphyromonas gingivalis Ehrlichia chaffeensis liberty Ehrlichia chaffeensis arkansa Ehrlichia chaffeensis wakulla Escherichia coli* | 3.48E-007 | 1 | 4.23E-01 | 9.90E-01 | 6.50E-02 | 1.00E+00 | 3.21E-02 | 9.93E-01 | 1.00E+00 | 5.79E-02 | 1.56E-01 | 1.00E+00 | 6.34E-01 | 2.88E-01 |
| 90 | 4 | 173 | *Yersinia enterocolitica wap bc Lactobacillus acidophilus Listeria monocytogenes Helicobacter pylori kx2 mgep* | 1.33E-002 | 11 | 9.20E-04 | 9.47E-01 | 3.03E-01 | 2.77E-01 | 9.37E-01 | 8.66E-01 | 2.02E-02 | 7.55E-01 | 1.22E-01 | 1.00E+00 | 1.00E+00 | 1.00E+00 |
| 92 | 3 | 226 | *Yersinia enterocolitica wap bc Pseudomonas aeruginosa streptococcus pneumoniae tigr4* | 4.42E-002 | 11 | 4.00E-03 | 5.00E-01 | 8.84E-01 | 6.08E-01 | 8.08E-01 | 7.74E-01 | 1.00E+00 | 6.46E-01 | 7.45E-01 | 1.63E-01 | 1.00E+00 | 1.00E+00 |
| 94 | 3 | 223 | *Ehrlichia chaffeensis arkansa streptococcus pneumoniae g54 Ehrlichia chaffeensis liberty* | 4.70E-002 | 12 | 1.80E-04 | 8.84E-01 | 5.00E-01 | 6.08E-01 | 8.08E-01 | 7.74E-01 | 1.00E+00 | 1.00E+00 | 1.00E+00 | 6.08E-01 | 1.00E+00 | 1.11E-01 |
| 96 | 7 | 93 | *Yersinia enterocolitica p60 bl6 Yersinia enterocolitica p60 bc Ehrlichia chaffeensis wakulla Pseudomonas aeruginosa fdr440 streptococcus pyogenes Pseudomonas aeruginosa Escherichia coli* | 5.40E-004 | 5 | 5.20E-03 | 7.95E-01 | 5.00E-01 | 9.03E-01 | 3.40E-01 | 9.76E-01 | 1.00E+00 | 3.12E-01 | 4.88E-01 | 6.02E-01 | 1.00E+00 | 8.42E-01 |
| 98 | 6 | 107 | *Helicobacter pylori kx1 mgep Burkholderia pseudomallei Listeria monocytogenes Mycobacterium tuberculosis Helicobacter pylori kx2 mgep Escherichia coli* | 2.45E-003 | 2 | 1.83E-01 | 3.31E-01 | 9.08E-01 | 8.60E-01 | 7.84E-01 | 7.55E-01 | 3.78E-01 | 2.16E-01 | 1.08E-01 | 8.60E-01 | 1.00E+00 | 7.89E-01 |
| 100 | 3 | 213 | *Pseudomonas aeruginosa fdr440 Pseudomonas aeruginosa Shigella dysenteriae* | 5.67E-002 | 16 | 2.95E-03 | 1.16E-01 | 1.00E+00 | 1.00E+00 | 3.54E-01 | 7.74E-01 | 1.00E+00 | 6.46E-01 | 7.45E-01 | 1.63E-01 | 1.00E+00 | 1.00E+00 |
| 102 | 7 | 90 | *Yersinia enterocolitica wap bc Burkholderia pseudomallei Yersinia enterocolitica p60 bl6 Yersinia enterocolitica p60 bc Ehrlichia chaffeensis wakulla Pseudomonas aeruginosa Helicobacter pylori kx2 mgep* | 8.34E-004 | 3 | 2.68E-02 | 9.53E-01 | 2.05E-01 | 1.00E+00 | 7.55E-02 | 9.76E-01 | 1.00E+00 | 8.83E-02 | 1.92E-01 | 9.03E-01 | 1.00E+00 | 4.72E-01 |
| 104 | 2 | 314 | *Brucella melitensis Brucella neotomae* | 1.62E-001 | 50 | 2.00E-05 | 1.00E+00 | 2.44E-01 | 1.00E+00 | 5.05E-01 | 1.00E+00 | 1.00E+00 | 7.67E-02 | 1.00E+00 | 1.00E+00 | 1.00E+00 | 4.18E-02 |
| 106 | 12 | 52 | *Yersinia enterocolitica wap bc Helicobacter pylori kx1 mgep Yersinia enterocolitica p60 bl6 Listeria monocytogenes Ehrlichia chaffeensis liberty Ehrlichia chaffeensis arkansa Ehrlichia chaffeensis wakulla streptococcus pneumoniae g54 streptococcus pyogenes Mycobacterium tuberculosis Helicobacter pylori kx2 mgep Escherichia coli* | 2.09E-006 |  | 1.00E+00 | 9.57E-01 | 1.53E-01 | 6.82E-01 | 7.93E-01 | 9.30E-01 | 6.46E-01 | 4.69E-01 | 1.93E-01 | 9.03E-01 | 1.00E+00 | 5.09E-01 |
| 108 | 3 | 207 | *Ehrlichia chaffeensis arkansa Yersinia enterocolitica p60 bl6 Shigella dysenteriae* | 6.33E-002 | 12 | 8.40E-04 | 8.84E-01 | 5.00E-01 | 1.00E+00 | 3.54E-01 | 1.00E+00 | 1.00E+00 | 1.92E-01 | 2.87E-01 | 1.00E+00 | 1.00E+00 | 5.25E-01 |
| 110 | 7 | 87 | *Pseudomonas aeruginosa fdr440 Mycobacterium tuberculosis Helicobacter pylori kx2 mgep Brucella neotomae streptococcus pneumoniae tigr4 Shigella dysenteriae Escherichia coli* | 1.27E-003 | 2 | 3.17E-01 | 2.05E-01 | 9.53E-01 | 9.03E-01 | 6.88E-01 | 5.46E-01 | 1.00E+00 | 8.83E-02 | 4.88E-01 | 2.56E-01 | 1.00E+00 | 8.42E-01 |
| 112 | 2 | 301 | *streptococcus pneumoniae g54 Bifidobacterium bifidum* | 1.74E-001 | 23 | 4.00E-05 | 7.56E-01 | 7.56E-01 | 6.39E-02 | 1.00E+00 | 6.23E-01 | 1.39E-01 | 1.00E+00 | 1.00E+00 | 4.60E-01 | 1.00E+00 | 1.00E+00 |
| 114 | 7 | 86 | *Ehrlichia chaffeensis wakulla Lactobacillus acidophilus Bifidobacterium bifidum Pseudomonas aeruginosa Mycobacterium tuberculosis Helicobacter pylori kx2 mgep Helicobacter pylori kx1 npgec* | 1.46E-003 | 4 | 2.86E-02 | 9.53E-01 | 2.05E-01 | 6.02E-01 | 9.12E-01 | 8.39E-01 | 6.71E-02 | 9.25E-01 | 8.03E-01 | 6.02E-01 | 1.00E+00 | 8.42E-01 |
| 116 | 3 | 196 | *Yersinia enterocolitica p60 bl6 Yersinia enterocolitica p60 bc Brucella neotomae* | 7.80E-002 | 24 | < 3.71E-07 | 1.00E+00 | 1.16E-01 | 1.00E+00 | 3.54E-01 | 1.00E+00 | 1.00E+00 | 1.92E-02 | 2.87E-01 | 1.00E+00 | 1.00E+00 | 5.25E-01 |
| 118 | 12 | 49 | *Yersinia enterocolitica wap bl6 Yersinia enterocolitica wap bc Yersinia enterocolitica p60 bl6 Yersinia enterocolitica p60 bc Lactobacillus acidophilus Listeria monocytogenes Bifidobacterium bifidum Helicobacter pylori kx1 npgec Aggregatibacter actinomycetemcomitans Ehrlichia chaffeensis arkansa Ehrlichia chaffeensis wakulla Mycobacterium tuberculosis* | 1.29E-005 | 1 | 3.67E-01 | 9.93E-01 | 4.28E-02 | 6.82E-01 | 7.93E-01 | 9.88E-01 | 1.92E-02 | 2.07E-01 | 1.93E-01 | 9.87E-01 | 7.55E-01 | 8.10E-01 |
| 120 | 3 | 195 | *Lactobacillus acidophilus Bifidobacterium bifidum Brucella neotomae* | 7.95E-002 | 23 | 6.10E-04 | 1.00E+00 | 1.16E-01 | 1.63E-01 | 9.81E-01 | 1.00E+00 | 1.03E-02 | 6.46E-01 | 1.00E+00 | 1.00E+00 | 1.00E+00 | 5.25E-01 |
| 122 | 4 | 146 | *Yersinia enterocolitica wap bl6 Yersinia enterocolitica p60 bc Brucella melitensis Escherichia coli* | 3.57E-002 | 13 | 4.00E-05 | 9.47E-01 | 3.03E-01 | 1.00E+00 | 2.45E-01 | 1.00E+00 | 1.00E+00 | 4.42E-03 | 1.22E-01 | 1.00E+00 | 1.00E+00 | 6.34E-01 |
| 124 | 3 | 192 | *Yersinia enterocolitica wap bl6 Listeria monocytogenes streptococcus pneumoniae tigr4* | 8.43E-002 | 8 | 1.54E-02 | 5.00E-01 | 8.84E-01 | 1.63E-01 | 9.81E-01 | 7.74E-01 | 2.04E-01 | 6.46E-01 | 2.87E-01 | 6.08E-01 | 1.00E+00 | 1.00E+00 |
| 126 | 8 | 72 | *Helicobacter pylori kx1 mgep Pseudomonas aeruginosa Brucella ovis Bacillus anthracis Brucella melitensis Pseudomonas aeruginosa fdr440 Helicobacter pylori kx2 mgep Escherichia coli* | 7.21E-004 | 6 | 4.40E-04 | 6.52E-01 | 6.52E-01 | 9.33E-01 | 2.57E-01 | 6.64E-01 | 1.00E+00 | 1.46E-01 | 6.05E-01 | 6.88E-01 | 1.00E+00 | 2.19E-01 |
| 128 | 2 | 288 | *Helicobacter pylori kx1 mgep Lactobacillus acidophilus* | 1.89E-001 | 26 | 2.78E-03 | 1.00E+00 | 2.44E-01 | 4.60E-01 | 9.23E-01 | 6.23E-01 | 1.39E-01 | 1.00E+00 | 5.92E-01 | 1.00E+00 | 1.00E+00 | 1.00E+00 |
| 130 | 5 | 115 | *Yersinia enterocolitica wap bl6 Yersinia enterocolitica p60 bl6 Ehrlichia chaffeensis wakulla Mycobacterium tuberculosis Brucella ovis* | 1.69E-002 | 9 | 5.00E-05 | 9.76E-01 | 1.72E-01 | 1.00E+00 | 5.54E-01 | 1.00E+00 | 1.00E+00 | 1.84E-02 | 5.96E-01 | 8.00E-01 | 1.00E+00 | 2.88E-01 |
| 132 | 3 | 188 | *streptococcus pneumoniae g54 Listeria monocytogenes Brucella neotomae* | 9.08E-002 | 9 | 1.08E-02 | 5.00E-01 | 8.84E-01 | 1.63E-01 | 9.81E-01 | 7.74E-01 | 2.04E-01 | 6.46E-01 | 7.45E-01 | 6.08E-01 | 1.00E+00 | 5.25E-01 |
| 134 | 12 | 47 | *Yersinia enterocolitica wap bc Yersinia enterocolitica p60 bl6 Yersinia enterocolitica p60 bc Lactobacillus acidophilus Listeria monocytogenes Bifidobacterium bifidum Ehrlichia chaffeensis liberty Ehrlichia chaffeensis arkansa Ehrlichia chaffeensis wakulla Mycobacterium tuberculosis Escherichia coli Helicobacter pylori* | 5.55E-008 | 1 | 1.67E-01 | 9.57E-01 | 1.53E-01 | 6.82E-01 | 7.93E-01 | 1.00E+00 | 1.92E-02 | 2.07E-01 | 1.93E-01 | 9.87E-01 | 1.00E+00 | 5.09E-01 |
| 136 | 4 | 141 | *Yersinia enterocolitica wap bl6 Ehrlichia chaffeensis wakulla streptococcus pneumoniae g54 Pseudomonas aeruginosa* | 4.24E-002 | 4 | 7.97E-03 | 6.97E-01 | 6.97E-01 | 7.19E-01 | 6.80E-01 | 8.66E-01 | 1.00E+00 | 7.55E-01 | 8.43E-01 | 2.77E-01 | 1.00E+00 | 6.34E-01 |
| 138 | 3 | 187 | *Pseudomonas aeruginosa fdr440 streptococcus pneumoniae g54 streptococcus pneumoniae tigr4* | 9.26E-002 | 5 | 9.27E-02 | 1.16E-01 | 1.00E+00 | 1.63E-01 | 9.81E-01 | 4.88E-02 | 1.00E+00 | 1.00E+00 | 1.00E+00 | 1.44E-02 | 1.00E+00 | 1.00E+00 |
| 140 | 3 | 185 | *Ehrlichia chaffeensis arkansa Ehrlichia chaffeensis wakulla Brucella melitensis* | 9.61E-002 | 25 | < 3.71E-07 | 1.00E+00 | 1.16E-01 | 1.00E+00 | 3.54E-01 | 1.00E+00 | 1.00E+00 | 6.46E-01 | 1.00E+00 | 1.00E+00 | 1.00E+00 | 7.32E-03 |
| 142 | 3 | 184 | *Helicobacter pylori kx1 mgep Yersinia enterocolitica p60 bc Ehrlichia chaffeensis liberty* | 9.78E-002 | 10 | 1.88E-03 | 1.00E+00 | 1.16E-01 | 1.00E+00 | 3.54E-01 | 7.74E-01 | 1.00E+00 | 6.46E-01 | 2.87E-01 | 1.00E+00 | 1.00E+00 | 5.25E-01 |
| 144 | 4 | 138 | *Yersinia enterocolitica wap bc Ehrlichia chaffeensis wakulla Bacillus anthracis Bifidobacterium bifidum* | 4.69E-002 | 11 | 1.30E-04 | 9.47E-01 | 3.03E-01 | 2.77E-01 | 9.37E-01 | 1.00E+00 | 2.65E-01 | 3.20E-01 | 8.43E-01 | 1.00E+00 | 1.00E+00 | 1.96E-01 |
| 146 | 8 | 69 | *Yersinia enterocolitica wap bc Helicobacter pylori kx1 mgep Lactobacillus acidophilus Bifidobacterium bifidum Aeromonas cavia Ehrlichia chaffeensis wakulla Pseudomonas aeruginosa fdr440 Escherichia coli* | 1.28E-003 | 1 | 4.23E-01 | 9.78E-01 | 1.19E-01 | 6.88E-01 | 5.89E-01 | 8.98E-01 | 8.78E-02 | 7.43E-01 | 2.94E-01 | 9.33E-01 | 1.00E+00 | 8.82E-01 |
| 148 | 3 | 180 | *Yersinia enterocolitica p60 bl6 Listeria monocytogenes streptococcus pyogenes* | 1.05E-001 | 15 | 1.00E-05 | 8.84E-01 | 5.00E-01 | 1.63E-01 | 9.81E-01 | 1.00E+00 | 2.04E-01 | 6.46E-01 | 2.87E-01 | 1.00E+00 | 1.00E+00 | 1.00E+00 |
| 150 | 3 | 179 | *Ehrlichia chaffeensis arkansa Helicobacter pylori kx2 mgep streptococcus pneumoniae tigr4* | 1.07E-001 | 5 | 1.72E-01 | 8.84E-01 | 5.00E-01 | 6.08E-01 | 8.08E-01 | 3.21E-01 | 1.00E+00 | 1.00E+00 | 7.45E-01 | 6.08E-01 | 1.00E+00 | 5.25E-01 |
| 152 | 4 | 133 | *Yersinia enterocolitica p60 bl6 Lactobacillus acidophilus Bacillus anthracis Escherichia coli* | 5.53E-002 | 11 | 3.70E-04 | 6.97E-01 | 6.97E-01 | 2.77E-01 | 9.37E-01 | 1.00E+00 | 2.65E-01 | 6.34E-02 | 4.51E-01 | 1.00E+00 | 1.00E+00 | 6.34E-01 |
| 154 | 7 | 75 | *Yersinia enterocolitica wap bc Ehrlichia chaffeensis arkansa Ehrlichia chaffeensis wakulla Pseudomonas aeruginosa Brucella neotomae Shigella dysenteriae Ehrlichia chaffeensis liberty* | 6.24E-003 | 5 | 8.80E-04 | 9.53E-01 | 2.05E-01 | 1.00E+00 | 7.55E-02 | 1.00E+00 | 1.00E+00 | 3.12E-01 | 8.03E-01 | 9.03E-01 | 1.00E+00 | 2.81E-02 |
| 156 | 10 | 51 | *Yersinia enterocolitica wap bc Helicobacter pylori kx1 mgep Yersinia enterocolitica p60 bl6 Listeria monocytogenes Bifidobacterium bifidum Pseudomonas aeruginosa Porphyromonas gingivalis streptococcus pneumoniae tigr4 Helicobacter pylori kx2 mgep Escherichia coli* | 3.27E-004 | 2 | 2.16E-02 | 6.41E-01 | 6.41E-01 | 5.24E-01 | 7.04E-01 | 5.85E-01 | 1.36E-01 | 6.00E-01 | 7.41E-02 | 8.19E-01 | 6.79E-01 | 1.00E+00 |
| 158 | 3 | 169 | *streptococcus pyogenes Aeromonas cavia Ehrlichia chaffeensis liberty* | 1.28E-001 | 25 | < 3.71E-07 | 1.00E+00 | 1.16E-01 | 6.08E-01 | 8.08E-01 | 1.00E+00 | 1.00E+00 | 1.00E+00 | 7.45E-01 | 1.00E+00 | 1.00E+00 | 5.25E-01 |
| 160 | 5 | 101 | *Yersinia enterocolitica wap bl6 Yersinia enterocolitica wap bc Bifidobacterium bifidum streptococcus pyogenes Helicobacter pylori kx2 mgep* | 3.64E-002 | 6 | 9.73E-03 | 1.00E+00 | 2.39E-02 | 3.93E-01 | 8.69E-01 | 9.23E-01 | 3.23E-01 | 4.46E-01 | 2.35E-01 | 1.00E+00 | 1.00E+00 | 1.00E+00 |
| 162 | 3 | 165 | *Listeria monocytogenes Brucella ovis Escherichia coli* | 1.36E-001 | 19 | 1.00E-05 | 5.00E-01 | 8.84E-01 | 6.08E-01 | 8.08E-01 | 1.00E+00 | 2.04E-01 | 1.92E-01 | 2.87E-01 | 1.00E+00 | 1.00E+00 | 5.25E-01 |
| 164 | 5 | 97 | *Yersinia enterocolitica wap bl6 Yersinia enterocolitica wap bc Ehrlichia chaffeensis arkansa Burkholderia pseudomallei Mycobacterium tuberculosis* | 4.47E-002 | 3 | 5.03E-02 | 8.28E-01 | 5.00E-01 | 1.00E+00 | 5.54E-01 | 1.00E+00 | 1.00E+00 | 1.84E-02 | 5.96E-01 | 8.00E-01 | 1.00E+00 | 2.88E-01 |
| 166 | 4 | 121 | *Yersinia enterocolitica wap bl6 Ehrlichia chaffeensis wakulla Helicobacter pylori kx1 npgec Ehrlichia chaffeensis liberty* | 8.02E-002 | 6 | 2.23E-02 | 1.00E+00 | 5.35E-02 | 1.00E+00 | 2.45E-01 | 8.66E-01 | 1.00E+00 | 7.55E-01 | 4.51E-01 | 1.00E+00 | 1.00E+00 | 1.96E-01 |
| 168 | 3 | 160 | *Yersinia enterocolitica p60 bc Bacillus anthracis Mycobacterium tuberculosis* | 1.48E-001 | 20 | < 3.71E-07 | 5.00E-01 | 8.84E-01 | 6.08E-01 | 9.81E-01 | 1.00E+00 | 1.00E+00 | 1.92E-02 | 7.45E-01 | 6.08E-01 | 1.00E+00 | 5.25E-01 |
| 170 | 6 | 80 | *Ehrlichia chaffeensis wakulla Bacillus anthracis streptococcus pneumoniae g54 Listeria monocytogenes streptococcus pneumoniae tigr4 Escherichia coli* | 2.61E-002 | 1 | 3.07E-01 | 9.18E-02 | 9.90E-01 | 3.21E-02 | 9.95E-01 | 7.55E-01 | 3.78E-01 | 5.61E-01 | 7.13E-01 | 5.03E-01 | 1.00E+00 | 3.82E-01 |
| 172 | 15 | 32 | *Yersinia enterocolitica wap bl6 Yersinia enterocolitica wap bc Helicobacter pylori kx2 npgec Helicobacter pylori kx1 mgep Yersinia enterocolitica p60 bl6 Yersinia enterocolitica p60 bc Listeria monocytogenes Pseudomonas aeruginosa streptococcus pneumoniae tigr4 Helicobacter pylori kx1 npgec Ehrlichia chaffeensis wakulla Pseudomonas aeruginosa fdr440 streptococcus pneumoniae g54 Helicobacter pylori kx2 mgep Shigella dysenteriae* | 1.72E-006 |  | 1.00E+00 | 9.01E-01 | 2.60E-01 | 8.52E-01 | 2.92E-01 | 3.00E-01 | 7.45E-01 | 4.34E-01 | 2.71E-03 | 6.16E-01 | 1.00E+00 | 9.89E-01 |
| 174 | 2 | 237 | *Helicobacter pylori kx1 mgep Brucella neotomae* | 2.76E-001 | 19 | 1.40E-03 | 1.00E+00 | 2.44E-01 | 1.00E+00 | 5.05E-01 | 6.23E-01 | 1.00E+00 | 4.95E-01 | 5.92E-01 | 1.00E+00 | 1.00E+00 | 3.87E-01 |
| 176 | 4 | 118 | *Helicobacter pylori kx1 mgep Brucella melitensis Bifidobacterium bifidum Mycobacterium tuberculosis* | 8.77E-002 | 11 | 1.70E-04 | 9.47E-01 | 3.03E-01 | 7.19E-01 | 9.37E-01 | 8.66E-01 | 2.65E-01 | 3.20E-01 | 8.43E-01 | 7.19E-01 | 1.00E+00 | 6.34E-01 |
| 178 | 7 | 67 | *Yersinia enterocolitica wap bc Yersinia enterocolitica p60 bl6 Yersinia enterocolitica p60 bc Brucella melitensis streptococcus pneumoniae g54 Mycobacterium tuberculosis Shigella dysenteriae* | 1.62E-002 | 2 | 9.05E-02 | 7.95E-01 | 5.00E-01 | 9.03E-01 | 6.88E-01 | 9.76E-01 | 1.00E+00 | 1.06E-03 | 1.92E-01 | 6.02E-01 | 1.00E+00 | 8.42E-01 |
| 180 | 7 | 67 | *Ehrlichia chaffeensis arkansa Ehrlichia chaffeensis wakulla Lactobacillus acidophilus Pseudomonas aeruginosa fdr440 Listeria monocytogenes streptococcus pyogenes Shigella dysenteriae* | 1.62E-002 | 3 | 1.18E-01 | 7.95E-01 | 5.00E-01 | 2.56E-01 | 9.12E-01 | 9.76E-01 | 6.71E-02 | 9.25E-01 | 8.03E-01 | 9.03E-01 | 1.00E+00 | 4.72E-01 |
| 182 | 5 | 93 | *Ehrlichia chaffeensis arkansa streptococcus pyogenes Mycobacterium tuberculosis Brucella ovis Brucella neotomae* | 5.46E-002 | 9 | 1.00E-05 | 9.76E-01 | 1.72E-01 | 8.00E-01 | 8.69E-01 | 1.00E+00 | 1.00E+00 | 1.31E-01 | 1.00E+00 | 8.00E-01 | 1.00E+00 | 5.72E-02 |
| 184 | 3 | 154 | *Bifidobacterium bifidum streptococcus pneumoniae tigr4 Shigella dysenteriae* | 1.61E-001 | 5 | 2.53E-01 | 5.00E-01 | 8.84E-01 | 1.63E-01 | 9.81E-01 | 7.74E-01 | 2.04E-01 | 6.46E-01 | 7.45E-01 | 6.08E-01 | 1.00E+00 | 1.00E+00 |
| 186 | 7 | 66 | *Yersinia enterocolitica wap bl6 Burkholderia pseudomallei Yersinia enterocolitica p60 bl6 Lactobacillus acidophilus streptococcus pneumoniae g54 Listeria monocytogenes Bifidobacterium bifidum* | 1.82E-002 |  | 1.00E+00 | 7.95E-01 | 5.00E-01 | 6.35E-02 | 9.86E-01 | 9.76E-01 | 3.05E-03 | 3.12E-01 | 4.88E-01 | 9.03E-01 | 1.00E+00 | 8.42E-01 |
| 188 | 11 | 42 | *Yersinia enterocolitica wap bc Helicobacter pylori kx1 mgep Yersinia enterocolitica p60 bc Pseudomonas aeruginosa streptococcus pneumoniae d39 Ehrlichia chaffeensis arkansa Ehrlichia chaffeensis wakulla Mycobacterium tuberculosis Helicobacter pylori kx2 mgep Escherichia coli Shigella dysenteriae* | 4.71E-004 |  | 1.00E+00 | 7.58E-01 | 5.00E-01 | 9.80E-01 | 3.18E-01 | 8.91E-01 | 1.00E+00 | 1.46E-01 | 1.26E-01 | 6.07E-01 | 1.00E+00 | 7.60E-01 |
| 190 | 4 | 115 | *Ehrlichia chaffeensis arkansa Bacillus anthracis Listeria monocytogenes Ehrlichia chaffeensis liberty* | 9.56E-002 | 7 | 2.19E-02 | 6.97E-01 | 6.97E-01 | 2.77E-01 | 9.37E-01 | 1.00E+00 | 2.65E-01 | 7.55E-01 | 8.43E-01 | 1.00E+00 | 1.00E+00 | 2.59E-02 |
| 192 | 3 | 152 | *Pseudomonas aeruginosa fdr440 Helicobacter pylori kx2 mgep Ehrlichia chaffeensis liberty* | 1.66E-001 | 8 | 5.49E-02 | 8.84E-01 | 5.00E-01 | 1.00E+00 | 3.54E-01 | 3.21E-01 | 1.00E+00 | 1.00E+00 | 7.45E-01 | 6.08E-01 | 1.00E+00 | 5.25E-01 |
| 194 | 9 | 50 | *Yersinia enterocolitica wap bl6 Yersinia enterocolitica wap bc Yersinia enterocolitica p60 bl6 Yersinia enterocolitica p60 bc Lactobacillus acidophilus Brucella ovis Aeromonas cavia Helicobacter pylori kx1 npgec streptococcus pyogenes* | 5.50E-003 | 1 | 1.67E-01 | 1.00E+00 | 6.60E-04 | 7.60E-01 | 4.91E-01 | 9.93E-01 | 5.25E-01 | 5.79E-02 | 3.83E-02 | 1.00E+00 | 1.00E+00 | 9.14E-01 |
| 196 | 6 | 74 | *Yersinia enterocolitica wap bl6 Yersinia enterocolitica wap bc Yersinia enterocolitica p60 bc Pseudomonas aeruginosa fdr440 Brucella ovis streptococcus pneumoniae tigr4* | 4.11E-002 | 3 | 5.03E-02 | 9.08E-01 | 3.31E-01 | 8.60E-01 | 4.39E-01 | 7.55E-01 | 1.00E+00 | 4.58E-02 | 3.61E-01 | 5.03E-01 | 1.00E+00 | 7.89E-01 |
| 198 | 6 | 73 | *Yersinia enterocolitica wap bl6 Yersinia enterocolitica p60 bc Lactobacillus acidophilus Bifidobacterium bifidum Porphyromonas gingivalis Mycobacterium tuberculosis* | 4.42E-002 | 6 | 2.60E-04 | 9.08E-01 | 3.31E-01 | 5.03E-01 | 9.54E-01 | 9.56E-01 | 4.88E-02 | 2.16E-01 | 7.13E-01 | 8.60E-01 | 4.74E-01 | 1.00E+00 |
| 200 | 4 | 109 | *Yersinia enterocolitica p60 bl6 Pseudomonas aeruginosa fdr440 Bifidobacterium bifidum Helicobacter pylori kx1 npgec* | 1.13E-001 | 6 | 2.36E-03 | 9.47E-01 | 3.03E-01 | 7.19E-01 | 6.80E-01 | 4.95E-01 | 2.65E-01 | 7.55E-01 | 4.51E-01 | 7.19E-01 | 1.00E+00 | 1.00E+00 |
| 202 | 8 | 54 | *Helicobacter pylori kx1 mgep Lactobacillus acidophilus Listeria monocytogenes Pseudomonas aeruginosa Aeromonas cavia Mycobacterium tuberculosis streptococcus pyogenes Helicobacter pylori kx2 mgep* | 1.65E-002 |  | 1.00E+00 | 8.81E-01 | 3.48E-01 | 3.45E-01 | 9.69E-01 | 8.98E-01 | 8.78E-02 | 9.50E-01 | 2.94E-01 | 6.88E-01 | 1.00E+00 | 1.00E+00 |
| 204 | 3 | 141 | *Ehrlichia chaffeensis wakulla streptococcus pneumoniae g54 streptococcus pyogenes* | 1.94E-001 | 10 | 2.00E-05 | 8.84E-01 | 5.00E-01 | 1.63E-01 | 9.81E-01 | 7.74E-01 | 1.00E+00 | 1.00E+00 | 1.00E+00 | 6.08E-01 | 1.00E+00 | 5.25E-01 |
| 206 | 7 | 60 | *Yersinia enterocolitica wap bc Yersinia enterocolitica p60 bc Bacillus anthracis Listeria monocytogenes streptococcus pyogenes Brucella neotomae Escherichia coli* | 3.47E-002 | 5 | 2.39E-03 | 7.95E-01 | 5.00E-01 | 2.56E-01 | 9.12E-01 | 1.00E+00 | 4.30E-01 | 1.38E-02 | 1.92E-01 | 1.00E+00 | 1.00E+00 | 4.72E-01 |
| 208 | 3 | 140 | *Ehrlichia chaffeensis arkansa Ehrlichia chaffeensis wakulla Helicobacter pylori* | 1.96E-001 | 17 | < 3.71E-07 | 8.84E-01 | 5.00E-01 | 1.00E+00 | 3.54E-01 | 1.00E+00 | 1.00E+00 | 1.00E+00 | 7.45E-01 | 1.00E+00 | 1.00E+00 | 1.11E-01 |
| 210 | 8 | 52 | *Helicobacter pylori kx1 mgep Ehrlichia chaffeensis arkansa Burkholderia pseudomallei Ehrlichia chaffeensis wakulla Lactobacillus acidophilus Pseudomonas aeruginosa fdr440 Brucella neotomae Escherichia coli* | 2.21E-002 | 2 | 4.07E-02 | 8.81E-01 | 3.48E-01 | 9.33E-01 | 2.57E-01 | 8.98E-01 | 4.79E-01 | 4.11E-01 | 8.68E-01 | 9.33E-01 | 1.00E+00 | 4.99E-02 |
| 212 | 7 | 59 | *Yersinia enterocolitica wap bc Yersinia enterocolitica p60 bc streptococcus pneumoniae g54 Listeria monocytogenes Bifidobacterium bifidum Helicobacter pylori kx1 npgec Ehrlichia chaffeensis liberty* | 3.84E-002 |  | 1.00E+00 | 9.53E-01 | 2.05E-01 | 2.56E-01 | 9.12E-01 | 8.39E-01 | 6.71E-02 | 6.60E-01 | 1.92E-01 | 9.03E-01 | 1.00E+00 | 8.42E-01 |
| 214 | 12 | 34 | *Yersinia enterocolitica wap bl6 Yersinia enterocolitica wap bc Helicobacter pylori kx1 mgep Yersinia enterocolitica p60 bl6 Yersinia enterocolitica p60 bc Ehrlichia chaffeensis wakulla Pseudomonas aeruginosa fdr1 Pseudomonas aeruginosa fdr440 Mycobacterium tuberculosis Helicobacter pylori kx2 mgep Escherichia coli Shigella dysenteriae* | 7.31E-004 |  | 1.00E+00 | 8.47E-01 | 3.67E-01 | 1.00E+00 | 6.71E-02 | 7.72E-01 | 1.00E+00 | 1.15E-02 | 1.16E-02 | 6.82E-01 | 1.00E+00 | 9.68E-01 |
| 216 | 7 | 58 | *Helicobacter pylori kx1 mgep Ehrlichia chaffeensis arkansa Ehrlichia chaffeensis wakulla streptococcus pneumoniae g54 Brucella ovis Helicobacter pylori kx2 mgep Shigella dysenteriae* | 4.25E-002 |  | 1.00E+00 | 9.53E-01 | 2.05E-01 | 9.03E-01 | 3.40E-01 | 5.46E-01 | 1.00E+00 | 6.60E-01 | 4.88E-01 | 9.03E-01 | 1.00E+00 | 1.55E-01 |
| 218 | 6 | 67 | *Ehrlichia chaffeensis arkansa Yersinia enterocolitica p60 bl6 Ehrlichia chaffeensis wakulla Helicobacter pylori kx2 mgep Aeromonas cavia Ehrlichia chaffeensis liberty* | 6.71E-002 | 4 | 1.63E-02 | 1.00E+00 | 1.03E-02 | 1.00E+00 | 1.13E-01 | 9.56E-01 | 1.00E+00 | 8.87E-01 | 3.61E-01 | 1.00E+00 | 1.00E+00 | 1.01E-01 |
| 220 | 2 | 200 | *Lactobacillus acidophilus Brucella melitensis* | 3.42E-001 | 23 | 1.96E-02 | 1.00E+00 | 2.44E-01 | 4.60E-01 | 9.23E-01 | 1.00E+00 | 1.39E-01 | 4.95E-01 | 1.00E+00 | 1.00E+00 | 1.00E+00 | 3.87E-01 |
| 222 | 8 | 49 | *Yersinia enterocolitica wap bc Burkholderia pseudomallei Pseudomonas aeruginosa streptococcus pneumoniae tigr4 Brucella melitensis Pseudomonas aeruginosa fdr440 Escherichia coli Shigella dysenteriae* | 3.38E-002 |  | 1.00E+00 | 1.19E-01 | 9.78E-01 | 9.33E-01 | 2.57E-01 | 8.98E-01 | 1.00E+00 | 3.09E-02 | 6.05E-01 | 3.45E-01 | 1.00E+00 | 5.57E-01 |
| 224 | 10 | 39 | *Yersinia enterocolitica wap bl6 Yersinia enterocolitica wap bc Yersinia enterocolitica p60 bl6 Yersinia enterocolitica p60 bc Listeria monocytogenes Aeromonas cavia streptococcus pneumoniae tigr4 Ehrlichia chaffeensis wakulla Mycobacterium tuberculosis Escherichia coli* | 1.17E-002 | 1 | 3.07E-01 | 8.61E-01 | 3.59E-01 | 8.19E-01 | 7.04E-01 | 9.96E-01 | 5.68E-01 | 1.96E-02 | 1.44E-02 | 8.19E-01 | 1.00E+00 | 9.37E-01 |
| 226 | 4 | 97 | *Listeria monocytogenes Pseudomonas aeruginosa Helicobacter pylori kx1 npgec Escherichia coli* | 1.55E-001 | 6 | 7.63E-03 | 3.03E-01 | 9.47E-01 | 7.19E-01 | 6.80E-01 | 8.66E-01 | 2.65E-01 | 7.55E-01 | 1.22E-01 | 7.19E-01 | 1.00E+00 | 1.00E+00 |
| 228 | 7 | 55 | *streptococcus pneumoniae g54 Bifidobacterium bifidum Pseudomonas aeruginosa Mycobacterium tuberculosis Brucella ovis Brucella neotomae Escherichia coli* | 5.70E-002 | 3 | 1.09E-02 | 5.00E-01 | 7.95E-01 | 6.02E-01 | 9.12E-01 | 9.76E-01 | 4.30E-01 | 8.83E-02 | 9.67E-01 | 2.56E-01 | 1.00E+00 | 4.72E-01 |
| 230 | 6 | 64 | *Ehrlichia chaffeensis arkansa Pseudomonas aeruginosa fdr440 Listeria monocytogenes Porphyromonas gingivalis Mycobacterium tuberculosis Escherichia coli* | 8.19E-002 | 3 | 5.84E-03 | 9.18E-02 | 9.90E-01 | 8.60E-01 | 7.84E-01 | 7.55E-01 | 3.78E-01 | 5.61E-01 | 7.13E-01 | 5.03E-01 | 4.74E-01 | 7.89E-01 |
| 232 | 2 | 191 | *streptococcus pneumoniae tigr4 Ehrlichia chaffeensis liberty* | 3.58E-001 | 7 | 5.97E-01 | 7.56E-01 | 7.56E-01 | 4.60E-01 | 9.23E-01 | 6.23E-01 | 1.00E+00 | 1.00E+00 | 1.00E+00 | 4.60E-01 | 1.00E+00 | 3.87E-01 |
| 234 | 5 | 76 | *Yersinia enterocolitica p60 bl6 Brucella melitensis Listeria monocytogenes Brucella neotomae streptococcus pneumoniae tigr4* | 1.17E-001 | 3 | 8.09E-02 | 8.28E-01 | 5.00E-01 | 3.93E-01 | 8.69E-01 | 9.23E-01 | 3.23E-01 | 1.31E-01 | 5.96E-01 | 8.00E-01 | 1.00E+00 | 2.88E-01 |
| 236 | 8 | 47 | *Lactobacillus acidophilus Bifidobacterium bifidum Aeromonas cavia Ehrlichia chaffeensis arkansa streptococcus pneumoniae g54 Mycobacterium tuberculosis streptococcus pyogenes Escherichia coli* | 4.42E-002 | 1 | 3.07E-01 | 8.81E-01 | 3.48E-01 | 1.07E-01 | 9.96E-01 | 9.87E-01 | 8.78E-02 | 7.43E-01 | 8.68E-01 | 6.88E-01 | 1.00E+00 | 8.82E-01 |
| 238 | 5 | 75 | *Ehrlichia chaffeensis arkansa Brucella melitensis streptococcus pneumoniae g54 Listeria monocytogenes streptococcus pyogenes* | 1.22E-001 | 1 | 3.07E-01 | 8.28E-01 | 5.00E-01 | 1.03E-01 | 9.82E-01 | 9.23E-01 | 3.23E-01 | 8.32E-01 | 9.05E-01 | 8.00E-01 | 1.00E+00 | 2.88E-01 |
| 240 | 8 | 46 | *Helicobacter pylori kx1 mgep Ehrlichia chaffeensis wakulla Bacillus anthracis streptococcus pyogenes Helicobacter pylori kx2 mgep Shigella dysenteriae Helicobacter pylori kx1 npgec Escherichia coli* | 5.04E-002 | 3 | 6.48E-02 | 8.81E-01 | 3.48E-01 | 6.88E-01 | 5.89E-01 | 6.64E-01 | 1.00E+00 | 4.11E-01 | 9.08E-02 | 1.00E+00 | 1.00E+00 | 5.57E-01 |
| 242 | 8 | 46 | *Burkholderia pseudomallei Yersinia enterocolitica p60 bl6 Yersinia enterocolitica p60 bc Porphyromonas gingivalis streptococcus pneumoniae tigr4 Ehrlichia chaffeensis wakulla Mycobacterium tuberculosis Shigella dysenteriae* | 5.04E-002 |  | 1.00E+00 | 3.48E-01 | 8.81E-01 | 9.33E-01 | 5.89E-01 | 8.98E-01 | 1.00E+00 | 3.09E-02 | 6.05E-01 | 6.88E-01 | 5.86E-01 | 5.57E-01 |
| 244 | 6 | 61 | *Ehrlichia chaffeensis wakulla Brucella melitensis Bifidobacterium bifidum streptococcus pyogenes Brucella neotomae Ehrlichia chaffeensis liberty* | 9.92E-002 | 4 | 3.64E-02 | 1.00E+00 | 1.03E-02 | 5.03E-01 | 7.84E-01 | 1.00E+00 | 3.78E-01 | 5.61E-01 | 1.00E+00 | 1.00E+00 | 1.00E+00 | 1.35E-02 |
| 246 | 19 | 19 | *Helicobacter pylori kx1 mgep Yersinia enterocolitica p60 bl6 Bifidobacterium bifidum Pseudomonas aeruginosa Brucella ovis Aggregatibacter actinomycetemcomitans Ehrlichia chaffeensis arkansa Ehrlichia chaffeensis wakulla Pseudomonas aeruginosa fdr440 Brucella melitensis streptococcus pyogenes Helicobacter pylori kx2 mgep Yersinia enterocolitica wap bl6 Yersinia enterocolitica wap bc Yersinia enterocolitica p60 bc Lactobacillus acidophilus Ehrlichia chaffeensis liberty Escherichia coli Shigella dysenteriae* | 5.41E-009 |  | 1.00E+00 | 9.99E-01 | 6.09E-03 | 9.62E-01 | 9.18E-02 | 9.92E-01 | 4.27E-01 | 7.76E-02 | 3.22E-01 | 9.95E-01 | 9.21E-01 | 3.71E-01 |
| 248 | 2 | 180 | *Helicobacter pylori kx1 mgep streptococcus pyogenes* | 3.78E-001 | 25 | < 3.71E-07 | 1.00E+00 | 2.44E-01 | 4.60E-01 | 9.23E-01 | 6.23E-01 | 1.00E+00 | 1.00E+00 | 5.92E-01 | 1.00E+00 | 1.00E+00 | 1.00E+00 |
| 250 | 4 | 89 | *streptococcus pyogenes Pseudomonas aeruginosa Mycobacterium tuberculosis Shigella dysenteriae* | 1.88E-001 | 8 | 2.72E-03 | 3.03E-01 | 9.47E-01 | 7.19E-01 | 9.37E-01 | 1.00E+00 | 1.00E+00 | 3.20E-01 | 8.43E-01 | 2.77E-01 | 1.00E+00 | 1.00E+00 |
| 252 | 5 | 70 | *Bacillus anthracis Pseudomonas aeruginosa fdr440 streptococcus pneumoniae g54 Bifidobacterium bifidum Helicobacter pylori kx2 mgep* | 1.50E-001 | 3 | 3.76E-02 | 5.00E-01 | 8.28E-01 | 1.03E-01 | 9.82E-01 | 2.75E-01 | 3.23E-01 | 8.32E-01 | 9.05E-01 | 3.93E-01 | 1.00E+00 | 7.21E-01 |
| 254 | 12 | 29 | *Yersinia enterocolitica wap bl6 Yersinia enterocolitica wap bc Helicobacter pylori kx2 npgec Yersinia enterocolitica p60 bl6 Yersinia enterocolitica p60 bc Lactobacillus acidophilus Bifidobacterium bifidum Ehrlichia chaffeensis liberty Ehrlichia chaffeensis arkansa Ehrlichia chaffeensis wakulla Bacillus anthracis Escherichia coli* | 6.04E-003 | 1 | 3.07E-01 | 9.99E-01 | 7.39E-03 | 6.82E-01 | 5.31E-01 | 9.99E-01 | 1.92E-01 | 6.11E-02 | 1.93E-01 | 1.00E+00 | 1.00E+00 | 2.16E-01 |
| 256 | 2 | 173 | *Yersinia enterocolitica wap bc Aeromonas cavia* | 3.90E-001 | 25 | 3.20E-04 | 1.00E+00 | 2.44E-01 | 1.00E+00 | 5.05E-01 | 1.00E+00 | 1.00E+00 | 4.95E-01 | 1.22E-01 | 1.00E+00 | 1.00E+00 | 1.00E+00 |
| 258 | 8 | 43 | *Yersinia enterocolitica wap bl6 Helicobacter pylori kx1 mgep Yersinia enterocolitica p60 bc Ehrlichia chaffeensis wakulla Pseudomonas aeruginosa fdr440 Helicobacter pylori kx2 mgep Escherichia coli Helicobacter pylori* | 7.36E-002 | 1 | 3.07E-01 | 8.81E-01 | 3.48E-01 | 1.00E+00 | 4.96E-02 | 6.64E-01 | 1.00E+00 | 4.11E-01 | 1.64E-02 | 9.33E-01 | 1.00E+00 | 8.82E-01 |
| 260 | 8 | 43 | *Yersinia enterocolitica wap bl6 Yersinia enterocolitica wap bc Yersinia enterocolitica p60 bl6 Listeria monocytogenes streptococcus pneumoniae tigr4 streptococcus pneumoniae d39 streptococcus pneumoniae g54 Mycobacterium tuberculosis* | 7.36E-002 | 2 | 2.83E-01 | 3.48E-01 | 8.81E-01 | 1.07E-01 | 9.96E-01 | 6.64E-01 | 4.79E-01 | 1.46E-01 | 2.94E-01 | 1.07E-01 | 1.00E+00 | 1.00E+00 |
| 262 | 3 | 114 | *Ehrlichia chaffeensis arkansa streptococcus pyogenes Helicobacter pylori kx1 npgec* | 2.74E-001 | 9 | 9.26E-03 | 1.00E+00 | 1.16E-01 | 6.08E-01 | 8.08E-01 | 7.74E-01 | 1.00E+00 | 1.00E+00 | 7.45E-01 | 1.00E+00 | 1.00E+00 | 5.25E-01 |
| 264 | 5 | 66 | *Pseudomonas aeruginosa fdr440 Bifidobacterium bifidum streptococcus pyogenes streptococcus pneumoniae tigr4 Escherichia coli* | 1.76E-001 | 2 | 2.16E-01 | 5.00E-01 | 8.28E-01 | 1.03E-01 | 9.82E-01 | 6.41E-01 | 3.23E-01 | 8.32E-01 | 9.05E-01 | 3.93E-01 | 1.00E+00 | 1.00E+00 |
| 266 | 6 | 55 | *Helicobacter pylori kx1 mgep Ehrlichia chaffeensis wakulla Lactobacillus acidophilus Porphyromonas gingivalis Helicobacter pylori kx2 mgep Ehrlichia chaffeensis liberty* | 1.44E-001 | 1 | 2.40E-01 | 9.90E-01 | 9.18E-02 | 8.60E-01 | 4.39E-01 | 4.13E-01 | 3.78E-01 | 1.00E+00 | 7.13E-01 | 1.00E+00 | 4.74E-01 | 3.82E-01 |
| 268 | 8 | 41 | *Yersinia enterocolitica wap bl6 Yersinia enterocolitica p60 bl6 Yersinia enterocolitica p60 bc Lactobacillus acidophilus streptococcus pneumoniae g54 streptococcus pyogenes Pseudomonas aeruginosa streptococcus pneumoniae tigr4* | 9.37E-002 | 1 | 3.67E-01 | 8.81E-01 | 3.48E-01 | 1.07E-01 | 9.69E-01 | 8.98E-01 | 4.79E-01 | 4.11E-01 | 6.05E-01 | 3.45E-01 | 1.00E+00 | 1.00E+00 |
| 270 | 2 | 163 | *Yersinia enterocolitica p60 bl6 Porphyromonas gingivalis* | 4.07E-001 | 14 | 6.40E-04 | 7.56E-01 | 7.56E-01 | 1.00E+00 | 5.05E-01 | 6.23E-01 | 1.00E+00 | 4.95E-01 | 5.92E-01 | 1.00E+00 | 1.84E-01 | 1.00E+00 |
| 272 | 4 | 81 | *Burkholderia pseudomallei Ehrlichia chaffeensis wakulla Listeria monocytogenes Ehrlichia chaffeensis liberty* | 2.29E-001 | 2 | 2.49E-01 | 6.97E-01 | 6.97E-01 | 7.19E-01 | 6.80E-01 | 1.00E+00 | 2.65E-01 | 7.55E-01 | 8.43E-01 | 1.00E+00 | 1.00E+00 | 2.59E-02 |
| 274 | 6 | 54 | *Yersinia enterocolitica wap bc Ehrlichia chaffeensis arkansa Ehrlichia chaffeensis wakulla Bifidobacterium bifidum streptococcus pyogenes Porphyromonas gingivalis* | 1.53E-001 | 5 | 8.80E-04 | 9.90E-01 | 9.18E-02 | 5.03E-01 | 7.84E-01 | 9.56E-01 | 3.78E-01 | 8.87E-01 | 9.44E-01 | 1.00E+00 | 4.74E-01 | 3.82E-01 |
| 276 | 5 | 64 | *Yersinia enterocolitica wap bl6 Yersinia enterocolitica wap bc Bacillus anthracis Listeria monocytogenes Porphyromonas gingivalis* | 1.91E-001 | 4 | 3.06E-03 | 5.00E-01 | 8.28E-01 | 3.93E-01 | 8.69E-01 | 9.23E-01 | 3.23E-01 | 1.31E-01 | 2.35E-01 | 1.00E+00 | 4.10E-01 | 7.21E-01 |
| 278 | 10 | 32 | *Yersinia enterocolitica wap bl6 Yersinia enterocolitica wap bc Yersinia enterocolitica p60 bl6 Lactobacillus acidophilus Ehrlichia chaffeensis arkansa Ehrlichia chaffeensis wakulla Bacillus anthracis Brucella melitensis streptococcus pyogenes Helicobacter pylori* | 5.76E-002 |  | 1.00E+00 | 9.96E-01 | 3.35E-02 | 5.24E-01 | 7.04E-01 | 1.00E+00 | 5.68E-01 | 9.63E-02 | 5.13E-01 | 1.00E+00 | 1.00E+00 | 1.18E-01 |
| 280 | 6 | 53 | *Helicobacter pylori kx2 npgec Helicobacter pylori kx1 mgep Pseudomonas aeruginosa Mycobacterium tuberculosis Helicobacter pylori kx2 mgep Escherichia coli* | 1.63E-001 | 6 | 4.44E-03 | 6.69E-01 | 6.69E-01 | 1.00E+00 | 4.39E-01 | 4.13E-01 | 1.00E+00 | 5.61E-01 | 1.08E-01 | 5.03E-01 | 1.00E+00 | 1.00E+00 |
| 282 | 4 | 79 | *Lactobacillus acidophilus Pseudomonas aeruginosa Brucella ovis Ehrlichia chaffeensis liberty* | 2.40E-001 | 5 | 3.60E-03 | 9.47E-01 | 3.03E-01 | 7.19E-01 | 6.80E-01 | 1.00E+00 | 2.65E-01 | 7.55E-01 | 1.00E+00 | 7.19E-01 | 1.00E+00 | 1.96E-01 |
| 284 | 7 | 45 | *Lactobacillus acidophilus Pseudomonas aeruginosa fdr440 Listeria monocytogenes streptococcus pyogenes Mycobacterium tuberculosis Escherichia coli Aggregatibacter actinomycetemcomitans* | 1.40E-001 | 3 | 1.09E-02 | 2.05E-01 | 9.53E-01 | 2.56E-01 | 9.86E-01 | 8.39E-01 | 6.71E-02 | 6.60E-01 | 8.03E-01 | 6.02E-01 | 5.32E-01 | 1.00E+00 |
| 286 | 8 | 39 | *Yersinia enterocolitica wap bc Helicobacter pylori kx1 mgep Burkholderia pseudomallei Yersinia enterocolitica p60 bl6 Listeria monocytogenes Pseudomonas aeruginosa streptococcus pyogenes Escherichia coli* | 1.19E-001 | 1 | 2.40E-01 | 6.52E-01 | 6.52E-01 | 6.88E-01 | 5.89E-01 | 9.87E-01 | 4.79E-01 | 1.46E-01 | 9.08E-02 | 9.33E-01 | 1.00E+00 | 8.82E-01 |
| 288 | 5 | 62 | *Yersinia enterocolitica wap bc Ehrlichia chaffeensis arkansa streptococcus pneumoniae g54 Porphyromonas gingivalis streptococcus pneumoniae tigr4* | 2.08E-001 |  | 1.00E+00 | 5.00E-01 | 8.28E-01 | 3.93E-01 | 8.69E-01 | 2.75E-01 | 1.00E+00 | 8.32E-01 | 9.05E-01 | 3.93E-01 | 4.10E-01 | 7.21E-01 |
| 290 | 14 | 22 | *Yersinia enterocolitica wap bl6 Yersinia enterocolitica wap bc Helicobacter pylori kx1 mgep Yersinia enterocolitica p60 bl6 Yersinia enterocolitica p60 bc Lactobacillus acidophilus Bifidobacterium bifidum Brucella ovis streptococcus pneumoniae d39 Brucella melitensis Pseudomonas aeruginosa fdr440 Brucella neotomae Helicobacter pylori kx2 mgep Escherichia coli* | 1.58E-003 |  | 1.00E+00 | 9.99E-01 | 1.01E-02 | 8.05E-01 | 3.65E-01 | 8.93E-01 | 2.54E-01 | 6.19E-03 | 1.53E-01 | 9.52E-01 | 1.00E+00 | 6.45E-01 |
| 292 | 6 | 51 | *Ehrlichia chaffeensis arkansa Brucella melitensis Listeria monocytogenes Brucella neotomae Aeromonas cavia Escherichia coli* | 1.84E-001 | 2 | 1.20E-01 | 9.08E-01 | 3.31E-01 | 8.60E-01 | 4.39E-01 | 1.00E+00 | 3.78E-01 | 2.16E-01 | 3.61E-01 | 1.00E+00 | 1.00E+00 | 1.01E-01 |
| 294 | 3 | 102 | *Burkholderia pseudomallei Yersinia enterocolitica p60 bc Pseudomonas aeruginosa fdr440* | 3.22E-001 | 3 | 6.48E-02 | 5.00E-01 | 8.84E-01 | 1.00E+00 | 3.54E-01 | 7.74E-01 | 1.00E+00 | 1.92E-01 | 7.45E-01 | 6.08E-01 | 1.00E+00 | 5.25E-01 |
| 296 | 2 | 152 | *Bacillus anthracis Shigella dysenteriae* | 4.32E-001 | 30 | < 3.71E-07 | 2.44E-01 | 1.00E+00 | 4.60E-01 | 9.23E-01 | 1.00E+00 | 1.00E+00 | 7.67E-02 | 5.92E-01 | 1.00E+00 | 1.00E+00 | 3.87E-01 |
| 298 | 6 | 50 | *Burkholderia pseudomallei Ehrlichia chaffeensis wakulla Lactobacillus acidophilus Brucella melitensis Bifidobacterium bifidum Brucella ovis* | 1.95E-001 | 1 | 2.40E-01 | 9.90E-01 | 9.18E-02 | 5.03E-01 | 7.84E-01 | 1.00E+00 | 4.88E-02 | 2.16E-01 | 1.00E+00 | 1.00E+00 | 1.00E+00 | 1.35E-02 |
| 300 | 3 | 99 | *Yersinia enterocolitica wap bc Shigella dysenteriae Helicobacter pylori kx1 npgec* | 3.35E-001 | 8 | 1.79E-02 | 8.84E-01 | 5.00E-01 | 1.00E+00 | 3.54E-01 | 7.74E-01 | 1.00E+00 | 1.92E-01 | 3.96E-02 | 1.00E+00 | 1.00E+00 | 1.00E+00 |
| 302 | 3 | 98 | *Mycobacterium tuberculosis Brucella neotomae Helicobacter pylori kx1 npgec* | 3.40E-001 | 10 | 2.00E-04 | 8.84E-01 | 5.00E-01 | 1.00E+00 | 8.08E-01 | 7.74E-01 | 1.00E+00 | 1.92E-01 | 7.45E-01 | 6.08E-01 | 1.00E+00 | 5.25E-01 |
| 304 | 7 | 42 | *Yersinia enterocolitica wap bc Ehrlichia chaffeensis arkansa Ehrlichia chaffeensis wakulla streptococcus pyogenes Aeromonas cavia Ehrlichia chaffeensis liberty Aggregatibacter actinomycetemcomitans* | 1.81E-001 | 1 | 4.23E-01 | 9.96E-01 | 4.66E-02 | 9.03E-01 | 3.40E-01 | 9.76E-01 | 1.00E+00 | 9.25E-01 | 8.03E-01 | 1.00E+00 | 5.32E-01 | 1.55E-01 |
| 306 | 10 | 29 | *Yersinia enterocolitica wap bc Helicobacter pylori kx1 mgep Yersinia enterocolitica p60 bc Pseudomonas aeruginosa Aeromonas cavia Pseudomonas aeruginosa fdr440 streptococcus pneumoniae g54 Helicobacter pylori kx2 mgep Escherichia coli Shigella dysenteriae* | 1.04E-001 |  | 1.00E+00 | 6.41E-01 | 6.41E-01 | 9.70E-01 | 1.37E-01 | 5.85E-01 | 1.00E+00 | 2.96E-01 | 1.44E-02 | 5.24E-01 | 1.00E+00 | 1.00E+00 |
| 308 | 9 | 32 | *Yersinia enterocolitica wap bc Helicobacter pylori kx1 mgep Yersinia enterocolitica p60 bc Listeria monocytogenes Pseudomonas aeruginosa streptococcus pneumoniae tigr4 Aggregatibacter actinomycetemcomitans Mycobacterium tuberculosis Helicobacter pylori kx2 mgep* | 1.38E-001 | 1 | 3.07E-01 | 5.00E-01 | 7.73E-01 | 7.60E-01 | 7.84E-01 | 4.71E-01 | 5.25E-01 | 5.09E-01 | 1.56E-01 | 4.36E-01 | 6.34E-01 | 1.00E+00 |
| 310 | 3 | 95 | *streptococcus pneumoniae g54 Listeria monocytogenes Fusobacterium nucleatum* | 3.55E-001 | 2 | 9.70E-01 | 1.16E-01 | 1.00E+00 | 1.63E-01 | 9.81E-01 | 3.21E-01 | 2.04E-01 | 1.00E+00 | 7.45E-01 | 6.08E-01 | 2.65E-01 | 1.00E+00 |
| 312 | 3 | 94 | *Brucella melitensis Brucella ovis Shigella dysenteriae* | 3.60E-001 | 17 | 1.80E-04 | 8.84E-01 | 5.00E-01 | 1.00E+00 | 3.54E-01 | 1.00E+00 | 1.00E+00 | 1.92E-02 | 7.45E-01 | 1.00E+00 | 1.00E+00 | 1.11E-01 |
| 314 | 7 | 40 | *Helicobacter pylori kx2 npgec Ehrlichia chaffeensis arkansa streptococcus pneumoniae g54 Listeria monocytogenes Bifidobacterium bifidum Mycobacterium tuberculosis Brucella neotomae* | 2.14E-001 | 2 | 4.07E-02 | 7.95E-01 | 5.00E-01 | 2.56E-01 | 9.86E-01 | 8.39E-01 | 6.71E-02 | 6.60E-01 | 8.03E-01 | 6.02E-01 | 1.00E+00 | 4.72E-01 |
| 316 | 8 | 35 | *Listeria monocytogenes Pseudomonas aeruginosa Aeromonas cavia Ehrlichia chaffeensis liberty Ehrlichia chaffeensis arkansa Ehrlichia chaffeensis wakulla streptococcus pyogenes Helicobacter pylori* | 1.87E-001 | 1 | 2.40E-01 | 8.81E-01 | 3.48E-01 | 6.88E-01 | 5.89E-01 | 1.00E+00 | 4.79E-01 | 1.00E+00 | 6.05E-01 | 9.33E-01 | 1.00E+00 | 2.19E-01 |
| 318 | 2 | 139 | *Burkholderia pseudomallei Brucella neotomae* | 4.76E-001 | 11 | 7.65E-02 | 7.56E-01 | 7.56E-01 | 1.00E+00 | 5.05E-01 | 1.00E+00 | 1.00E+00 | 7.67E-02 | 1.00E+00 | 1.00E+00 | 1.00E+00 | 4.18E-02 |
| 320 | 6 | 46 | *Helicobacter pylori kx1 mgep Yersinia enterocolitica p60 bc Pseudomonas aeruginosa fdr440 Brucella melitensis Pseudomonas aeruginosa Porphyromonas gingivalis* | 2.48E-001 | 3 | 5.84E-03 | 6.69E-01 | 6.69E-01 | 1.00E+00 | 1.13E-01 | 4.13E-01 | 1.00E+00 | 5.61E-01 | 7.13E-01 | 5.03E-01 | 4.74E-01 | 7.89E-01 |
| 322 | 4 | 69 | *Yersinia enterocolitica wap bl6 Yersinia enterocolitica wap bc Listeria monocytogenes Helicobacter pylori* | 3.11E-001 | 3 | 2.68E-02 | 6.97E-01 | 6.97E-01 | 7.19E-01 | 6.80E-01 | 1.00E+00 | 2.65E-01 | 3.20E-01 | 1.22E-02 | 1.00E+00 | 1.00E+00 | 1.00E+00 |
| 324 | 4 | 69 | *Helicobacter pylori kx1 mgep Lactobacillus acidophilus streptococcus pneumoniae tigr4 Helicobacter pylori kx1 npgec* | 3.11E-001 | 4 | 9.15E-02 | 9.47E-01 | 3.03E-01 | 2.77E-01 | 9.37E-01 | 1.46E-01 | 2.65E-01 | 1.00E+00 | 4.51E-01 | 7.19E-01 | 1.00E+00 | 1.00E+00 |
| 326 | 5 | 55 | *Bacillus anthracis Pseudomonas aeruginosa fdr440 Brucella melitensis streptococcus pyogenes Mycobacterium tuberculosis* | 2.77E-001 | 6 | 1.11E-03 | 5.00E-01 | 8.28E-01 | 3.93E-01 | 9.82E-01 | 9.23E-01 | 1.00E+00 | 1.31E-01 | 1.00E+00 | 3.93E-01 | 1.00E+00 | 2.88E-01 |
| 328 | 2 | 137 | *streptococcus gordonii Fusobacterium nucleatum* | 4.82E-001 | 48 | < 3.71E-07 | 2.44E-01 | 1.00E+00 | 4.60E-01 | 9.23E-01 | 1.39E-01 | 1.00E+00 | 1.00E+00 | 1.00E+00 | 1.00E+00 | 6.97E-03 | 1.00E+00 |
| 330 | 3 | 90 | *Burkholderia pseudomallei Bacillus anthracis Escherichia coli* | 3.82E-001 | 9 | 1.10E-04 | 1.16E-01 | 1.00E+00 | 6.08E-01 | 8.08E-01 | 1.00E+00 | 1.00E+00 | 1.92E-02 | 7.45E-01 | 1.00E+00 | 1.00E+00 | 1.11E-01 |
| 332 | 19 | 14 | *Helicobacter pylori kx1 mgep Yersinia enterocolitica p60 bl6 Pseudomonas aeruginosa Brucella ovis Ehrlichia chaffeensis arkansa Ehrlichia chaffeensis wakulla Mycobacterium tuberculosis Helicobacter pylori kx2 mgep Yersinia enterocolitica wap bl6 Yersinia enterocolitica wap bc Burkholderia pseudomallei Yersinia enterocolitica p60 bc Lactobacillus acidophilus Ehrlichia chaffeensis liberty Helicobacter pylori kx1 npgec Bacillus anthracis Brucella neotomae Escherichia coli Helicobacter pylori* | 2.69E-006 |  | 1.00E+00 | 9.94E-01 | 3.08E-02 | 9.95E-01 | 9.18E-02 | 9.99E-01 | 8.46E-01 | 2.28E-03 | 1.34E-01 | 9.95E-01 | 1.00E+00 | 3.27E-02 |
| 334 | 3 | 88 | *Bacillus anthracis Brucella ovis Brucella neotomae* | 3.94E-001 | 13 | < 3.71E-07 | 8.84E-01 | 5.00E-01 | 6.08E-01 | 8.08E-01 | 1.00E+00 | 1.00E+00 | 1.92E-02 | 1.00E+00 | 1.00E+00 | 1.00E+00 | 7.32E-03 |
| 336 | 7 | 37 | *Yersinia enterocolitica wap bc Bacillus anthracis Pseudomonas aeruginosa fdr440 Listeria monocytogenes streptococcus pneumoniae d39 Shigella dysenteriae Escherichia coli* | 2.73E-001 | 1 | 4.73E-01 | 4.66E-02 | 9.96E-01 | 2.56E-01 | 9.12E-01 | 8.39E-01 | 4.30E-01 | 8.83E-02 | 1.92E-01 | 6.02E-01 | 1.00E+00 | 8.42E-01 |
| 338 | 6 | 43 | *Yersinia enterocolitica wap bl6 Yersinia enterocolitica wap bc Yersinia enterocolitica p60 bc Bacillus anthracis Escherichia coli Aggregatibacter actinomycetemcomitans* | 2.97E-001 | 2 | 9.05E-02 | 6.69E-01 | 6.69E-01 | 8.60E-01 | 4.39E-01 | 9.56E-01 | 1.00E+00 | 4.71E-03 | 1.08E-01 | 1.00E+00 | 4.74E-01 | 7.89E-01 |
| 340 | 8 | 32 | *Pseudomonas aeruginosa fdr1 Lactobacillus acidophilus Pseudomonas aeruginosa fdr440 Listeria monocytogenes Bifidobacterium bifidum Pseudomonas aeruginosa Helicobacter pylori kx2 mgep Escherichia coli* | 2.58E-001 | 3 | 1.79E-02 | 3.48E-01 | 8.81E-01 | 3.45E-01 | 8.54E-01 | 6.64E-01 | 4.88E-03 | 9.50E-01 | 6.05E-01 | 3.45E-01 | 1.00E+00 | 1.00E+00 |
| 342 | 3 | 84 | *Bifidobacterium bifidum Pseudomonas aeruginosa Aeromonas cavia* | 4.18E-001 | 9 | 3.14E-03 | 8.84E-01 | 5.00E-01 | 6.08E-01 | 8.08E-01 | 1.00E+00 | 2.04E-01 | 1.00E+00 | 7.45E-01 | 6.08E-01 | 1.00E+00 | 1.00E+00 |
| 344 | 4 | 63 | *Yersinia enterocolitica p60 bc Ehrlichia chaffeensis wakulla Bacillus anthracis Aeromonas cavia* | 3.64E-001 | 8 | 6.90E-04 | 9.47E-01 | 3.03E-01 | 7.19E-01 | 6.80E-01 | 1.00E+00 | 1.00E+00 | 3.20E-01 | 4.51E-01 | 1.00E+00 | 1.00E+00 | 1.96E-01 |
| 346 | 4 | 63 | *Ehrlichia chaffeensis arkansa Mycobacterium tuberculosis Aeromonas cavia Shigella dysenteriae* | 3.64E-001 | 7 | 1.86E-02 | 6.97E-01 | 6.97E-01 | 1.00E+00 | 6.80E-01 | 1.00E+00 | 1.00E+00 | 3.20E-01 | 4.51E-01 | 7.19E-01 | 1.00E+00 | 6.34E-01 |
| 348 | 7 | 36 | *Yersinia enterocolitica wap bl6 Yersinia enterocolitica p60 bl6 Pseudomonas aeruginosa fdr440 streptococcus pneumoniae g54 Porphyromonas gingivalis Mycobacterium tuberculosis Brucella ovis* | 2.96E-001 |  | 1.00E+00 | 5.00E-01 | 7.95E-01 | 9.03E-01 | 6.88E-01 | 5.46E-01 | 1.00E+00 | 8.83E-02 | 8.03E-01 | 2.56E-01 | 5.32E-01 | 8.42E-01 |
| 350 | 9 | 28 | *Yersinia enterocolitica wap bc Helicobacter pylori kx1 mgep Porphyromonas gingivalis streptococcus pneumoniae tigr4 Pseudomonas aeruginosa fdr440 Mycobacterium tuberculosis streptococcus pyogenes Helicobacter pylori kx2 mgep Shigella dysenteriae* | 2.42E-001 |  | 1.00E+00 | 5.00E-01 | 7.73E-01 | 7.60E-01 | 7.84E-01 | 2.02E-01 | 1.00E+00 | 5.09E-01 | 4.04E-01 | 4.36E-01 | 6.34E-01 | 1.00E+00 |
| 352 | 4 | 62 | *Helicobacter pylori kx1 mgep Helicobacter pylori kx2 mgep streptococcus pneumoniae tigr4 streptococcus pneumoniae d39* | 3.74E-001 | 3 | 1.82E-01 | 6.97E-01 | 6.97E-01 | 2.77E-01 | 9.37E-01 | 1.63E-02 | 1.00E+00 | 1.00E+00 | 4.51E-01 | 2.77E-01 | 1.00E+00 | 1.00E+00 |
| 354 | 2 | 124 | *streptococcus pneumoniae g54 Aeromonas cavia* | 5.21E-001 | 27 | < 3.71E-07 | 7.56E-01 | 7.56E-01 | 4.60E-01 | 9.23E-01 | 6.23E-01 | 1.00E+00 | 1.00E+00 | 5.92E-01 | 4.60E-01 | 1.00E+00 | 1.00E+00 |
| 356 | 3 | 82 | *Lactobacillus acidophilus Mycobacterium tuberculosis Helicobacter pylori* | 4.31E-001 | 8 | 1.10E-04 | 5.00E-01 | 8.84E-01 | 6.08E-01 | 9.81E-01 | 1.00E+00 | 2.04E-01 | 6.46E-01 | 7.45E-01 | 6.08E-01 | 1.00E+00 | 1.00E+00 |
| 358 | 5 | 49 | *Helicobacter pylori kx2 npgec Ehrlichia chaffeensis arkansa Ehrlichia chaffeensis wakulla Helicobacter pylori kx2 mgep Helicobacter pylori kx1 npgec* | 3.55E-001 | 8 | 1.25E-03 | 1.00E+00 | 2.39E-02 | 1.00E+00 | 1.68E-01 | 2.75E-01 | 1.00E+00 | 1.00E+00 | 2.35E-01 | 1.00E+00 | 1.00E+00 | 2.88E-01 |
| 360 | 3 | 81 | *Porphyromonas gingivalis Shigella dysenteriae Escherichia coli* | 4.37E-001 | 8 | 5.27E-03 | 1.16E-01 | 1.00E+00 | 1.00E+00 | 3.54E-01 | 7.74E-01 | 1.00E+00 | 1.92E-01 | 2.87E-01 | 1.00E+00 | 2.65E-01 | 1.00E+00 |
| 362 | 6 | 40 | *Helicobacter pylori kx1 mgep Yersinia enterocolitica p60 bl6 Bifidobacterium bifidum Helicobacter pylori kx1 npgec Escherichia coli Aggregatibacter actinomycetemcomitans* | 3.55E-001 | 2 | 9.05E-02 | 9.08E-01 | 3.31E-01 | 8.60E-01 | 4.39E-01 | 4.13E-01 | 3.78E-01 | 5.61E-01 | 1.08E-01 | 1.00E+00 | 4.74E-01 | 1.00E+00 |
| 364 | 5 | 48 | *Ehrlichia chaffeensis arkansa Yersinia enterocolitica p60 bl6 Ehrlichia chaffeensis wakulla Bifidobacterium bifidum streptococcus pneumoniae d39* | 3.70E-001 | 1 | 4.73E-01 | 9.76E-01 | 1.72E-01 | 3.93E-01 | 8.69E-01 | 9.23E-01 | 3.23E-01 | 8.32E-01 | 9.05E-01 | 8.00E-01 | 1.00E+00 | 2.88E-01 |
| 366 | 7 | 34 | *Yersinia enterocolitica p60 bl6 Yersinia enterocolitica p60 bc Lactobacillus acidophilus streptococcus pyogenes Porphyromonas gingivalis Aeromonas cavia Escherichia coli* | 3.46E-001 | 1 | 3.67E-01 | 9.53E-01 | 2.05E-01 | 6.02E-01 | 6.88E-01 | 9.76E-01 | 4.30E-01 | 3.12E-01 | 1.92E-01 | 1.00E+00 | 5.32E-01 | 1.00E+00 |
| 368 | 6 | 39 | *Burkholderia pseudomallei Ehrlichia chaffeensis wakulla Bifidobacterium bifidum streptococcus pyogenes Mycobacterium tuberculosis Helicobacter pylori kx2 mgep* | 3.75E-001 | 3 | 1.79E-02 | 9.08E-01 | 3.31E-01 | 5.03E-01 | 9.54E-01 | 9.56E-01 | 3.78E-01 | 5.61E-01 | 9.44E-01 | 8.60E-01 | 1.00E+00 | 3.82E-01 |
| 370 | 8 | 29 | *Yersinia enterocolitica wap bl6 Lactobacillus acidophilus Porphyromonas gingivalis Ehrlichia chaffeensis liberty Ehrlichia chaffeensis arkansa Ehrlichia chaffeensis wakulla Escherichia coli Helicobacter pylori* | 3.51E-001 |  | 1.00E+00 | 8.81E-01 | 3.48E-01 | 9.33E-01 | 2.57E-01 | 9.87E-01 | 4.79E-01 | 7.43E-01 | 6.05E-01 | 1.00E+00 | 5.86E-01 | 2.19E-01 |
| 372 | 11 | 21 | *Helicobacter pylori kx2 npgec Lactobacillus acidophilus Bifidobacterium bifidum Pseudomonas aeruginosa Brucella ovis Helicobacter pylori kx1 npgec Ehrlichia chaffeensis wakulla Brucella melitensis Pseudomonas aeruginosa fdr440 streptococcus pyogenes Escherichia coli* | 1.89E-001 | 2 | 6.40E-02 | 9.84E-01 | 7.95E-02 | 6.07E-01 | 6.18E-01 | 8.91E-01 | 1.63E-01 | 6.82E-01 | 8.52E-01 | 8.66E-01 | 1.00E+00 | 4.36E-01 |
| 374 | 5 | 46 | *Ehrlichia chaffeensis arkansa Ehrlichia chaffeensis wakulla streptococcus pyogenes Brucella ovis streptococcus pneumoniae tigr4* | 4.00E-001 | 2 | 2.83E-01 | 9.76E-01 | 1.72E-01 | 3.93E-01 | 8.69E-01 | 9.23E-01 | 1.00E+00 | 8.32E-01 | 1.00E+00 | 8.00E-01 | 1.00E+00 | 5.72E-02 |
| 376 | 4 | 57 | *Helicobacter pylori kx2 mgep Aeromonas cavia Helicobacter pylori kx1 npgec Escherichia coli* | 4.25E-001 | 4 | 2.86E-02 | 9.47E-01 | 3.03E-01 | 1.00E+00 | 2.45E-01 | 4.95E-01 | 1.00E+00 | 7.55E-01 | 1.22E-02 | 1.00E+00 | 1.00E+00 | 1.00E+00 |
| 378 | 15 | 15 | *Yersinia enterocolitica wap bc Helicobacter pylori kx1 mgep Yersinia enterocolitica p60 bl6 Lactobacillus acidophilus Bifidobacterium bifidum Pseudomonas aeruginosa Ehrlichia chaffeensis liberty Ehrlichia chaffeensis arkansa Ehrlichia chaffeensis wakulla Pseudomonas aeruginosa fdr440 streptococcus pneumoniae g54 streptococcus pyogenes Mycobacterium tuberculosis Escherichia coli Helicobacter pylori* | 9.81E-003 |  | 1.00E+00 | 9.01E-01 | 2.60E-01 | 6.16E-01 | 8.07E-01 | 9.86E-01 | 2.87E-01 | 7.08E-01 | 7.15E-01 | 6.16E-01 | 1.00E+00 | 7.06E-01 |
| 380 | 15 | 15 | *Yersinia enterocolitica wap bl6 Yersinia enterocolitica wap bc Helicobacter pylori kx1 mgep Yersinia enterocolitica p60 bl6 Yersinia enterocolitica p60 bc Pseudomonas aeruginosa streptococcus pneumoniae tigr4 Ehrlichia chaffeensis arkansa streptococcus gordonii Ehrlichia chaffeensis wakulla streptococcus pneumoniae g54 Mycobacterium tuberculosis Helicobacter pylori kx2 mgep Escherichia coli Shigella dysenteriae* | 9.81E-003 |  | 1.00E+00 | 7.40E-01 | 5.00E-01 | 8.52E-01 | 5.66E-01 | 7.88E-01 | 1.00E+00 | 5.86E-02 | 7.56E-02 | 6.16E-01 | 8.43E-01 | 9.15E-01 |
| 382 | 8 | 28 | *Yersinia enterocolitica wap bc Yersinia enterocolitica p60 bl6 Aggregatibacter actinomycetemcomitans Ehrlichia chaffeensis arkansa Ehrlichia chaffeensis wakulla Pseudomonas aeruginosa fdr440 streptococcus pneumoniae g54 Mycobacterium tuberculosis* | 3.86E-001 |  | 1.00E+00 | 6.52E-01 | 6.52E-01 | 9.33E-01 | 5.89E-01 | 6.64E-01 | 1.00E+00 | 4.11E-01 | 8.68E-01 | 3.45E-01 | 5.86E-01 | 5.57E-01 |
| 384 | 7 | 32 | *Yersinia enterocolitica wap bc Ehrlichia chaffeensis arkansa Yersinia enterocolitica p60 bc Ehrlichia chaffeensis wakulla Pseudomonas aeruginosa fdr1 Listeria monocytogenes Ehrlichia chaffeensis liberty* | 4.03E-001 |  | 1.00E+00 | 9.53E-01 | 2.05E-01 | 9.03E-01 | 3.40E-01 | 9.76E-01 | 4.30E-01 | 6.60E-01 | 4.88E-01 | 9.03E-01 | 1.00E+00 | 1.55E-01 |
| 386 | 14 | 16 | *Yersinia enterocolitica wap bl6 Yersinia enterocolitica wap bc Helicobacter pylori kx2 npgec Helicobacter pylori kx1 mgep Burkholderia pseudomallei Yersinia enterocolitica p60 bl6 Yersinia enterocolitica p60 bc streptococcus pneumoniae d39 Helicobacter pylori kx1 npgec Bacillus anthracis Mycobacterium tuberculosis Helicobacter pylori kx2 mgep Escherichia coli Shigella dysenteriae* | 2.63E-002 |  | 1.00E+00 | 8.37E-01 | 3.72E-01 | 9.52E-01 | 3.65E-01 | 7.10E-01 | 1.00E+00 | 6.30E-04 | 1.08E-03 | 9.52E-01 | 1.00E+00 | 8.87E-01 |
| 388 | 8 | 28 | *Yersinia enterocolitica wap bl6 Yersinia enterocolitica p60 bc Listeria monocytogenes streptococcus pyogenes Pseudomonas aeruginosa Porphyromonas gingivalis Helicobacter pylori kx2 mgep Helicobacter pylori kx1 npgec* | 3.86E-001 | 1 | 1.67E-01 | 8.81E-01 | 3.48E-01 | 6.88E-01 | 5.89E-01 | 6.64E-01 | 4.79E-01 | 7.43E-01 | 9.08E-02 | 9.33E-01 | 5.86E-01 | 1.00E+00 |
| 390 | 6 | 36 | *Brucella melitensis Listeria monocytogenes Mycobacterium tuberculosis Fusobacterium nucleatum Brucella ovis Brucella neotomae* | 4.43E-001 | 1 | 6.00E-01 | 6.69E-01 | 6.69E-01 | 8.60E-01 | 7.84E-01 | 9.56E-01 | 3.78E-01 | 4.58E-02 | 9.44E-01 | 8.60E-01 | 4.74E-01 | 1.01E-01 |
| 392 | 4 | 54 | *Helicobacter pylori kx1 mgep Burkholderia pseudomallei Mycobacterium tuberculosis Brucella ovis* | 4.58E-001 | 3 | 1.09E-02 | 6.97E-01 | 6.97E-01 | 1.00E+00 | 6.80E-01 | 8.66E-01 | 1.00E+00 | 6.34E-02 | 8.43E-01 | 7.19E-01 | 1.00E+00 | 1.96E-01 |
| 394 | 5 | 43 | *Helicobacter pylori kx2 npgec streptococcus pneumoniae g54 streptococcus pneumoniae tigr4 Shigella dysenteriae Escherichia coli* | 4.50E-001 | 1 | 6.67E-01 | 1.72E-01 | 9.76E-01 | 3.93E-01 | 8.69E-01 | 2.75E-01 | 1.00E+00 | 4.46E-01 | 2.35E-01 | 3.93E-01 | 1.00E+00 | 1.00E+00 |
| 396 | 6 | 35 | *Pseudomonas aeruginosa fdr1 Pseudomonas aeruginosa fdr440 streptococcus pneumoniae g54 Listeria monocytogenes streptococcus pneumoniae tigr4 Shigella dysenteriae* | 4.67E-001 | 2 | 6.40E-02 | 1.03E-02 | 1.00E+00 | 1.73E-01 | 9.54E-01 | 1.36E-01 | 3.78E-01 | 8.87E-01 | 7.13E-01 | 3.21E-02 | 1.00E+00 | 1.00E+00 |
| 398 | 3 | 70 | *Yersinia enterocolitica p60 bl6 Bifidobacterium bifidum Helicobacter pylori* | 5.08E-001 | 2 | 1.83E-01 | 8.84E-01 | 5.00E-01 | 6.08E-01 | 8.08E-01 | 1.00E+00 | 2.04E-01 | 6.46E-01 | 2.87E-01 | 1.00E+00 | 1.00E+00 | 1.00E+00 |
| 400 | 8 | 26 | *Yersinia enterocolitica wap bc Burkholderia pseudomallei Yersinia enterocolitica p60 bc Lactobacillus acidophilus Listeria monocytogenes Pseudomonas aeruginosa Aeromonas cavia Mycobacterium tuberculosis* | 4.64E-001 |  | 1.00E+00 | 6.52E-01 | 6.52E-01 | 6.88E-01 | 8.54E-01 | 1.00E+00 | 8.78E-02 | 1.46E-01 | 2.94E-01 | 6.88E-01 | 1.00E+00 | 8.82E-01 |
| 402 | 3 | 69 | *Yersinia enterocolitica wap bl6 Yersinia enterocolitica p60 bc streptococcus pneumoniae d39* | 5.14E-001 | 5 | 2.60E-02 | 8.84E-01 | 5.00E-01 | 6.08E-01 | 8.08E-01 | 7.74E-01 | 1.00E+00 | 1.92E-01 | 2.87E-01 | 6.08E-01 | 1.00E+00 | 1.00E+00 |
| 404 | 2 | 103 | *Bacillus anthracis Helicobacter pylori kx1 npgec* | 6.01E-001 | 13 | 1.10E-04 | 7.56E-01 | 7.56E-01 | 4.60E-01 | 9.23E-01 | 6.23E-01 | 1.00E+00 | 4.95E-01 | 5.92E-01 | 1.00E+00 | 1.00E+00 | 3.87E-01 |
| 406 | 6 | 34 | *Ehrlichia chaffeensis wakulla Brucella melitensis Mycobacterium tuberculosis Brucella ovis Escherichia coli Aggregatibacter actinomycetemcomitans* | 4.92E-001 | 2 | 6.40E-02 | 6.69E-01 | 6.69E-01 | 1.00E+00 | 4.39E-01 | 9.56E-01 | 1.00E+00 | 4.58E-02 | 9.44E-01 | 8.60E-01 | 4.74E-01 | 1.01E-01 |
| 408 | 12 | 17 | *Yersinia enterocolitica wap bl6 Helicobacter pylori kx1 mgep Yersinia enterocolitica p60 bl6 Listeria monocytogenes streptococcus pneumoniae d39 Ehrlichia chaffeensis liberty Ehrlichia chaffeensis arkansa Ehrlichia chaffeensis wakulla streptococcus pyogenes Mycobacterium tuberculosis Helicobacter pylori kx2 mgep Escherichia coli* | 1.73E-001 |  | 1.00E+00 | 9.57E-01 | 1.53E-01 | 6.82E-01 | 7.93E-01 | 9.30E-01 | 6.46E-01 | 4.69E-01 | 1.93E-01 | 9.03E-01 | 1.00E+00 | 5.09E-01 |
| 410 | 8 | 25 | *Yersinia enterocolitica wap bc Listeria monocytogenes streptococcus pneumoniae tigr4 Ehrlichia chaffeensis arkansa Ehrlichia chaffeensis wakulla Mycobacterium tuberculosis Helicobacter pylori Shigella dysenteriae* | 5.06E-001 |  | 1.00E+00 | 3.48E-01 | 8.81E-01 | 6.88E-01 | 8.54E-01 | 9.87E-01 | 4.79E-01 | 4.11E-01 | 2.94E-01 | 6.88E-01 | 1.00E+00 | 5.57E-01 |
| 412 | 8 | 25 | *Yersinia enterocolitica wap bl6 Yersinia enterocolitica p60 bc Ehrlichia chaffeensis wakulla Brucella melitensis streptococcus pneumoniae g54 Brucella ovis Brucella neotomae Aeromonas cavia* | 5.06E-001 | 4 | 7.60E-04 | 9.98E-01 | 2.24E-02 | 9.33E-01 | 2.57E-01 | 9.87E-01 | 1.00E+00 | 3.09E-02 | 6.05E-01 | 9.33E-01 | 1.00E+00 | 4.99E-02 |
| 414 | 5 | 40 | *Yersinia enterocolitica wap bc Helicobacter pylori kx2 npgec Helicobacter pylori kx1 mgep Yersinia enterocolitica p60 bc Brucella neotomae* | 5.03E-001 | 5 | 2.39E-03 | 1.00E+00 | 2.39E-02 | 1.00E+00 | 1.68E-01 | 6.41E-01 | 1.00E+00 | 1.31E-01 | 4.69E-02 | 1.00E+00 | 1.00E+00 | 7.21E-01 |
| 416 | 25 | 8 | *Helicobacter pylori kx1 mgep Yersinia enterocolitica p60 bl6 Listeria monocytogenes Bifidobacterium bifidum Pseudomonas aeruginosa streptococcus pneumoniae tigr4 Ehrlichia chaffeensis arkansa Ehrlichia chaffeensis wakulla Pseudomonas aeruginosa fdr1 Pseudomonas aeruginosa fdr440 Brucella melitensis Mycobacterium tuberculosis Helicobacter pylori kx2 mgep Yersinia enterocolitica wap bl6 Yersinia enterocolitica wap bc Burkholderia pseudomallei Helicobacter pylori kx2 npgec Yersinia enterocolitica p60 bc Lactobacillus acidophilus Ehrlichia chaffeensis liberty Helicobacter pylori kx1 npgec streptococcus pneumoniae g54 Brucella neotomae Shigella dysenteriae Escherichia coli* | 5.63E-005 |  | 1.00E+00 | 9.71E-01 | 1.04E-01 | 9.28E-01 | 3.25E-01 | 9.05E-01 | 2.00E-01 | 4.75E-02 | 1.51E-01 | 7.74E-01 | 1.00E+00 | 4.63E-01 |
| 418 | 3 | 66 | *streptococcus gordonii Listeria monocytogenes Escherichia coli* | 5.34E-001 | 5 | 5.16E-01 | 1.16E-01 | 1.00E+00 | 1.63E-01 | 9.81E-01 | 7.74E-01 | 2.04E-01 | 6.46E-01 | 2.87E-01 | 1.00E+00 | 2.65E-01 | 1.00E+00 |
| 420 | 7 | 28 | *Yersinia enterocolitica wap bc Yersinia enterocolitica p60 bl6 Bacillus anthracis Pseudomonas aeruginosa Porphyromonas gingivalis Helicobacter pylori kx2 mgep streptococcus pneumoniae d39* | 5.32E-001 |  | 1.00E+00 | 5.00E-01 | 7.95E-01 | 6.02E-01 | 6.88E-01 | 5.46E-01 | 1.00E+00 | 3.12E-01 | 4.88E-01 | 6.02E-01 | 5.32E-01 | 8.42E-01 |
| 422 | 7 | 28 | *Helicobacter pylori kx1 mgep streptococcus pneumoniae g54 Listeria monocytogenes streptococcus pyogenes Aeromonas cavia streptococcus pneumoniae tigr4 Ehrlichia chaffeensis liberty* | 5.32E-001 |  | 1.00E+00 | 7.95E-01 | 5.00E-01 | 6.35E-02 | 9.86E-01 | 5.46E-01 | 4.30E-01 | 1.00E+00 | 4.88E-01 | 6.02E-01 | 1.00E+00 | 8.42E-01 |
| 424 | 3 | 65 | *Burkholderia pseudomallei Brucella melitensis streptococcus pneumoniae g54* | 5.41E-001 |  | 1.00E+00 | 5.00E-01 | 8.84E-01 | 6.08E-01 | 8.08E-01 | 7.74E-01 | 1.00E+00 | 1.92E-01 | 1.00E+00 | 6.08E-01 | 1.00E+00 | 1.11E-01 |
| 426 | 4 | 48 | *Lactobacillus acidophilus Bifidobacterium bifidum Brucella neotomae Aggregatibacter actinomycetemcomitans* | 5.28E-001 | 4 | 1.17E-02 | 9.47E-01 | 3.03E-01 | 2.77E-01 | 9.37E-01 | 8.66E-01 | 2.02E-02 | 7.55E-01 | 1.00E+00 | 1.00E+00 | 3.41E-01 | 6.34E-01 |
| 428 | 3 | 63 | *Burkholderia pseudomallei Yersinia enterocolitica p60 bl6 Helicobacter pylori kx1 npgec* | 5.55E-001 | 5 | 5.20E-03 | 8.84E-01 | 5.00E-01 | 1.00E+00 | 3.54E-01 | 7.74E-01 | 1.00E+00 | 1.92E-01 | 2.87E-01 | 1.00E+00 | 1.00E+00 | 5.25E-01 |
| 430 | 4 | 47 | *Burkholderia pseudomallei Listeria monocytogenes Bifidobacterium bifidum Porphyromonas gingivalis* | 5.40E-001 | 1 | 1.67E-01 | 3.03E-01 | 9.47E-01 | 2.77E-01 | 9.37E-01 | 8.66E-01 | 2.02E-02 | 7.55E-01 | 8.43E-01 | 1.00E+00 | 3.41E-01 | 6.34E-01 |
| 432 | 3 | 62 | *Yersinia enterocolitica wap bl6 Helicobacter pylori kx2 npgec Yersinia enterocolitica p60 bl6* | 5.62E-001 | 8 | 5.00E-04 | 1.00E+00 | 1.16E-01 | 1.00E+00 | 3.54E-01 | 7.74E-01 | 1.00E+00 | 1.92E-01 | 3.96E-02 | 1.00E+00 | 1.00E+00 | 1.00E+00 |
| 434 | 4 | 46 | *Ehrlichia chaffeensis arkansa Mycobacterium tuberculosis Fusobacterium nucleatum Escherichia coli* | 5.53E-001 |  | 1.00E+00 | 3.03E-01 | 9.47E-01 | 1.00E+00 | 6.80E-01 | 8.66E-01 | 1.00E+00 | 3.20E-01 | 8.43E-01 | 7.19E-01 | 3.41E-01 | 6.34E-01 |
| 436 | 8 | 23 | *Yersinia enterocolitica wap bc Helicobacter pylori kx1 mgep Brucella ovis Pseudomonas aeruginosa fdr1 Brucella melitensis Pseudomonas aeruginosa fdr440 Brucella neotomae Helicobacter pylori kx2 mgep* | 5.93E-001 | 2 | 4.07E-02 | 9.78E-01 | 1.19E-01 | 1.00E+00 | 4.96E-02 | 3.51E-01 | 1.00E+00 | 1.46E-01 | 6.05E-01 | 6.88E-01 | 1.00E+00 | 2.19E-01 |
| 438 | 4 | 46 | *Lactobacillus acidophilus Pseudomonas aeruginosa streptococcus pneumoniae d39 Escherichia coli* | 5.53E-001 |  | 1.00E+00 | 3.03E-01 | 9.47E-01 | 2.77E-01 | 9.37E-01 | 8.66E-01 | 2.65E-01 | 7.55E-01 | 8.43E-01 | 2.77E-01 | 1.00E+00 | 1.00E+00 |
| 440 | 3 | 61 | *streptococcus gordonii streptococcus pneumoniae g54 Shigella dysenteriae* | 5.69E-001 |  | 1.00E+00 | 1.16E-01 | 1.00E+00 | 1.63E-01 | 9.81E-01 | 3.21E-01 | 1.00E+00 | 6.46E-01 | 7.45E-01 | 6.08E-01 | 2.65E-01 | 1.00E+00 |
| 442 | 3 | 60 | *Helicobacter pylori kx2 npgec streptococcus pyogenes Mycobacterium tuberculosis* | 5.76E-001 | 7 | 1.50E-03 | 8.84E-01 | 5.00E-01 | 6.08E-01 | 9.81E-01 | 7.74E-01 | 1.00E+00 | 6.46E-01 | 7.45E-01 | 6.08E-01 | 1.00E+00 | 1.00E+00 |
| 444 | 10 | 18 | *Yersinia enterocolitica wap bl6 Yersinia enterocolitica wap bc Helicobacter pylori kx1 mgep Yersinia enterocolitica p60 bl6 Listeria monocytogenes Fusobacterium nucleatum streptococcus pneumoniae tigr4 Mycobacterium tuberculosis Helicobacter pylori kx2 mgep Shigella dysenteriae* | 5.24E-001 |  | 1.00E+00 | 6.41E-01 | 6.41E-01 | 8.19E-01 | 7.04E-01 | 5.85E-01 | 5.68E-01 | 9.63E-02 | 1.44E-02 | 8.19E-01 | 6.79E-01 | 1.00E+00 |
| 446 | 4 | 44 | *Yersinia enterocolitica p60 bl6 Yersinia enterocolitica p60 bc Pseudomonas aeruginosa fdr1 Pseudomonas aeruginosa* | 5.80E-001 | 4 | 5.13E-03 | 6.97E-01 | 6.97E-01 | 1.00E+00 | 2.45E-01 | 8.66E-01 | 1.00E+00 | 3.20E-01 | 4.51E-01 | 2.77E-01 | 1.00E+00 | 1.00E+00 |
| 448 | 4 | 44 | *Helicobacter pylori kx2 npgec Bacillus anthracis Pseudomonas aeruginosa fdr440 Pseudomonas aeruginosa* | 5.80E-001 | 5 | 5.20E-03 | 3.03E-01 | 9.47E-01 | 7.19E-01 | 6.80E-01 | 4.95E-01 | 1.00E+00 | 7.55E-01 | 8.43E-01 | 2.77E-01 | 1.00E+00 | 6.34E-01 |
| 450 | 8 | 22 | *Yersinia enterocolitica wap bc Helicobacter pylori kx1 mgep Lactobacillus acidophilus Aeromonas cavia Aggregatibacter actinomycetemcomitans Mycobacterium tuberculosis Helicobacter pylori kx2 mgep Escherichia coli* | 6.38E-001 |  | 1.00E+00 | 8.81E-01 | 3.48E-01 | 9.33E-01 | 5.89E-01 | 6.64E-01 | 4.79E-01 | 4.11E-01 | 9.08E-02 | 9.33E-01 | 5.86E-01 | 1.00E+00 |
| 452 | 22 | 8 | *Helicobacter pylori kx1 mgep Yersinia enterocolitica p60 bl6 Bifidobacterium bifidum Pseudomonas aeruginosa Porphyromonas gingivalis Brucella ovis Ehrlichia chaffeensis arkansa Ehrlichia chaffeensis wakulla Pseudomonas aeruginosa fdr440 Brucella melitensis streptococcus pyogenes Helicobacter pylori kx2 mgep Yersinia enterocolitica wap bl6 Yersinia enterocolitica wap bc Yersinia enterocolitica p60 bc Lactobacillus acidophilus Aeromonas cavia Helicobacter pylori kx1 npgec Bacillus anthracis Brucella neotomae Escherichia coli Helicobacter pylori* | 1.17E-005 |  | 1.00E+00 | 1.00E+00 | 2.40E-03 | 9.45E-01 | 1.11E-01 | 9.94E-01 | 5.37E-01 | 6.37E-02 | 1.45E-01 | 9.99E-01 | 9.57E-01 | 2.79E-01 |
| 454 | 7 | 25 | *streptococcus gordonii Eubacterium rectale Pseudomonas aeruginosa fdr1234 Staphylococcus aureus Listeria monocytogenes Fusobacterium nucleatum Shigella dysenteriae* | 6.39E-001 |  | 1.00E+00 | 4.66E-02 | 9.96E-01 | 6.35E-02 | 9.86E-01 | 2.36E-01 | 4.30E-01 | 9.25E-01 | 4.88E-01 | 6.02E-01 | 1.23E-01 | 1.00E+00 |
| 456 | 4 | 43 | *Lactobacillus acidophilus Pseudomonas aeruginosa fdr440 Aeromonas cavia streptococcus pneumoniae tigr4* | 5.94E-001 |  | 1.00E+00 | 6.97E-01 | 6.97E-01 | 2.77E-01 | 9.37E-01 | 4.95E-01 | 2.65E-01 | 1.00E+00 | 8.43E-01 | 2.77E-01 | 1.00E+00 | 1.00E+00 |
| 458 | 3 | 57 | *Porphyromonas gingivalis Mycobacterium tuberculosis Brucella neotomae* | 5.99E-001 | 10 | < 3.71E-07 | 5.00E-01 | 8.84E-01 | 1.00E+00 | 8.08E-01 | 7.74E-01 | 1.00E+00 | 1.92E-01 | 1.00E+00 | 6.08E-01 | 2.65E-01 | 5.25E-01 |
| 460 | 9 | 19 | *Yersinia enterocolitica wap bl6 Yersinia enterocolitica p60 bl6 Yersinia enterocolitica p60 bc Listeria monocytogenes Porphyromonas gingivalis Aggregatibacter actinomycetemcomitans Pseudomonas aeruginosa fdr440 Mycobacterium tuberculosis Escherichia coli* | 6.60E-001 | 1 | 2.40E-01 | 2.27E-01 | 9.35E-01 | 9.55E-01 | 4.91E-01 | 7.60E-01 | 5.25E-01 | 5.79E-02 | 1.56E-01 | 7.60E-01 | 1.96E-01 | 1.00E+00 |
| 462 | 3 | 57 | *Ehrlichia chaffeensis arkansa streptococcus gordonii Ehrlichia chaffeensis wakulla* | 5.99E-001 | 18 | < 3.71E-07 | 8.84E-01 | 5.00E-01 | 6.08E-01 | 8.08E-01 | 7.74E-01 | 1.00E+00 | 1.00E+00 | 1.00E+00 | 1.00E+00 | 2.65E-01 | 1.11E-01 |
| 464 | 10 | 17 | *Yersinia enterocolitica wap bl6 Yersinia enterocolitica p60 bl6 Yersinia enterocolitica p60 bc Lactobacillus acidophilus Ehrlichia chaffeensis liberty Aggregatibacter actinomycetemcomitans Ehrlichia chaffeensis arkansa Ehrlichia chaffeensis wakulla Mycobacterium tuberculosis Helicobacter pylori* | 5.72E-001 |  | 1.00E+00 | 9.67E-01 | 1.39E-01 | 9.70E-01 | 4.00E-01 | 9.96E-01 | 5.68E-01 | 2.96E-01 | 5.13E-01 | 9.70E-01 | 6.79E-01 | 3.62E-01 |
| 466 | 13 | 13 | *Yersinia enterocolitica wap bl6 Yersinia enterocolitica wap bc Burkholderia pseudomallei Yersinia enterocolitica p60 bl6 Yersinia enterocolitica p60 bc Bifidobacterium bifidum Pseudomonas aeruginosa streptococcus pneumoniae d39 Ehrlichia chaffeensis wakulla Bacillus anthracis Brucella melitensis streptococcus pyogenes Escherichia coli* | 2.06E-001 |  | 1.00E+00 | 9.10E-01 | 2.53E-01 | 4.62E-01 | 7.23E-01 | 1.00E+00 | 6.82E-01 | 3.01E-03 | 5.34E-01 | 9.31E-01 | 1.00E+00 | 2.75E-01 |
| 468 | 3 | 56 | *Helicobacter pylori kx2 npgec Ehrlichia chaffeensis arkansa Brucella melitensis* | 6.07E-001 | 4 | 5.53E-02 | 1.00E+00 | 1.16E-01 | 1.00E+00 | 3.54E-01 | 7.74E-01 | 1.00E+00 | 6.46E-01 | 7.45E-01 | 1.00E+00 | 1.00E+00 | 1.11E-01 |
| 470 | 2 | 84 | *Brucella ovis Aeromonas cavia* | 6.53E-001 | 27 | < 3.71E-07 | 1.00E+00 | 2.44E-01 | 1.00E+00 | 5.05E-01 | 1.00E+00 | 1.00E+00 | 4.95E-01 | 5.92E-01 | 1.00E+00 | 1.00E+00 | 3.87E-01 |
| 472 | 4 | 42 | *Ehrlichia chaffeensis wakulla Pseudomonas aeruginosa Ehrlichia chaffeensis liberty Aggregatibacter actinomycetemcomitans* | 6.08E-001 | 4 | 5.13E-03 | 6.97E-01 | 6.97E-01 | 1.00E+00 | 2.45E-01 | 8.66E-01 | 1.00E+00 | 1.00E+00 | 1.00E+00 | 7.19E-01 | 3.41E-01 | 1.96E-01 |
| 474 | 3 | 55 | *Fusobacterium nucleatum Shigella dysenteriae Escherichia coli* | 6.15E-001 | 3 | 9.65E-01 | 1.16E-01 | 1.00E+00 | 1.00E+00 | 3.54E-01 | 7.74E-01 | 1.00E+00 | 1.92E-01 | 2.87E-01 | 1.00E+00 | 2.65E-01 | 1.00E+00 |
| 476 | 3 | 54 | *Helicobacter pylori kx1 mgep Pseudomonas aeruginosa fdr1 Escherichia coli* | 6.23E-001 | 7 | 1.50E-03 | 5.00E-01 | 8.84E-01 | 1.00E+00 | 3.54E-01 | 3.21E-01 | 1.00E+00 | 6.46E-01 | 2.87E-01 | 6.08E-01 | 1.00E+00 | 1.00E+00 |
| 478 | 6 | 27 | *Helicobacter pylori kx1 mgep Lactobacillus acidophilus streptococcus pneumoniae g54 Bifidobacterium bifidum Aeromonas cavia Helicobacter pylori kx1 npgec* | 6.87E-001 | 1 | 3.07E-01 | 9.90E-01 | 9.18E-02 | 1.73E-01 | 9.54E-01 | 4.13E-01 | 4.88E-02 | 1.00E+00 | 3.61E-01 | 8.60E-01 | 1.00E+00 | 1.00E+00 |
| 480 | 3 | 54 | *streptococcus pneumoniae g54 streptococcus pneumoniae d39 Shigella dysenteriae* | 6.23E-001 | 1 | 8.78E-01 | 1.16E-01 | 1.00E+00 | 1.63E-01 | 9.81E-01 | 3.21E-01 | 1.00E+00 | 6.46E-01 | 7.45E-01 | 1.63E-01 | 1.00E+00 | 1.00E+00 |
| 482 | 10 | 16 | *Yersinia enterocolitica wap bl6 Yersinia enterocolitica wap bc Yersinia enterocolitica p60 bl6 Lactobacillus acidophilus Bifidobacterium bifidum streptococcus pneumoniae d39 Aggregatibacter actinomycetemcomitans Ehrlichia chaffeensis wakulla Escherichia coli Shigella dysenteriae* | 6.21E-001 |  | 1.00E+00 | 8.61E-01 | 3.59E-01 | 5.24E-01 | 7.04E-01 | 9.62E-01 | 1.36E-01 | 9.63E-02 | 2.39E-01 | 9.70E-01 | 6.79E-01 | 9.37E-01 |
| 484 | 4 | 39 | *Helicobacter pylori kx2 npgec Listeria monocytogenes Brucella ovis Escherichia coli* | 6.53E-001 | 3 | 8.09E-02 | 6.97E-01 | 6.97E-01 | 7.19E-01 | 6.80E-01 | 8.66E-01 | 2.65E-01 | 3.20E-01 | 1.22E-01 | 1.00E+00 | 1.00E+00 | 6.34E-01 |
| 486 | 7 | 22 | *Helicobacter pylori kx2 npgec Ehrlichia chaffeensis arkansa Ehrlichia chaffeensis wakulla Pseudomonas aeruginosa Mycobacterium tuberculosis streptococcus pneumoniae tigr4 streptococcus pneumoniae d39* | 7.49E-001 | 1 | 3.07E-01 | 5.00E-01 | 7.95E-01 | 6.02E-01 | 9.12E-01 | 5.46E-01 | 1.00E+00 | 9.25E-01 | 9.67E-01 | 6.35E-02 | 1.00E+00 | 4.72E-01 |
| 488 | 4 | 38 | *Ehrlichia chaffeensis arkansa Listeria monocytogenes Shigella dysenteriae Aggregatibacter actinomycetemcomitans* | 6.69E-001 | 1 | 3.67E-01 | 3.03E-01 | 9.47E-01 | 7.19E-01 | 6.80E-01 | 8.66E-01 | 2.65E-01 | 7.55E-01 | 4.51E-01 | 1.00E+00 | 3.41E-01 | 6.34E-01 |
| 490 | 5 | 30 | *Ehrlichia chaffeensis wakulla Brucella melitensis Brucella ovis Brucella neotomae Helicobacter pylori* | 7.12E-001 | 4 | 3.06E-03 | 9.76E-01 | 1.72E-01 | 1.00E+00 | 1.68E-01 | 1.00E+00 | 1.00E+00 | 1.31E-01 | 9.05E-01 | 1.00E+00 | 1.00E+00 | 5.04E-03 |
| 492 | 7 | 21 | *Burkholderia pseudomallei Ehrlichia chaffeensis wakulla Bacillus anthracis Listeria monocytogenes streptococcus pyogenes Brucella ovis Brucella neotomae* | 7.84E-001 |  | 1.00E+00 | 7.95E-01 | 5.00E-01 | 2.56E-01 | 9.12E-01 | 1.00E+00 | 4.30E-01 | 8.83E-02 | 9.67E-01 | 1.00E+00 | 1.00E+00 | 2.57E-03 |
| 494 | 5 | 29 | *Ehrlichia chaffeensis arkansa streptococcus gordonii Listeria monocytogenes streptococcus pyogenes Fusobacterium nucleatum* | 7.34E-001 |  | 1.00E+00 | 5.00E-01 | 8.28E-01 | 1.03E-01 | 9.82E-01 | 6.41E-01 | 3.23E-01 | 1.00E+00 | 9.05E-01 | 1.00E+00 | 6.29E-02 | 7.21E-01 |
| 496 | 6 | 24 | *Burkholderia pseudomallei Lactobacillus acidophilus Brucella melitensis Bifidobacterium bifidum Brucella neotomae Aeromonas cavia* | 7.74E-001 |  | 1.00E+00 | 9.90E-01 | 9.18E-02 | 5.03E-01 | 7.84E-01 | 1.00E+00 | 4.88E-02 | 2.16E-01 | 9.44E-01 | 1.00E+00 | 1.00E+00 | 1.01E-01 |
| 498 | 3 | 48 | *Yersinia enterocolitica wap bc Burkholderia pseudomallei streptococcus pneumoniae d39* | 6.79E-001 | 1 | 3.07E-01 | 5.00E-01 | 8.84E-01 | 6.08E-01 | 8.08E-01 | 7.74E-01 | 1.00E+00 | 1.92E-01 | 7.45E-01 | 6.08E-01 | 1.00E+00 | 5.25E-01 |
| 500 | 3 | 47 | *streptococcus gordonii streptococcus pneumoniae g54 Brucella neotomae* | 6.90E-001 | 4 | 3.64E-02 | 5.00E-01 | 8.84E-01 | 1.63E-01 | 9.81E-01 | 3.21E-01 | 1.00E+00 | 6.46E-01 | 1.00E+00 | 6.08E-01 | 2.65E-01 | 5.25E-01 |
| 502 | 2 | 70 | *Ehrlichia chaffeensis wakulla Fusobacterium nucleatum* | 7.16E-001 | 22 | < 3.71E-07 | 7.56E-01 | 7.56E-01 | 1.00E+00 | 5.05E-01 | 6.23E-01 | 1.00E+00 | 1.00E+00 | 1.00E+00 | 1.00E+00 | 1.84E-01 | 3.87E-01 |
| 504 | 4 | 35 | *Ehrlichia chaffeensis wakulla Pseudomonas aeruginosa fdr1 Helicobacter pylori kx2 mgep streptococcus pneumoniae tigr4* | 7.17E-001 | 3 | 1.79E-02 | 6.97E-01 | 6.97E-01 | 7.19E-01 | 6.80E-01 | 1.46E-01 | 1.00E+00 | 1.00E+00 | 8.43E-01 | 2.77E-01 | 1.00E+00 | 6.34E-01 |
| 506 | 5 | 28 | *Ehrlichia chaffeensis wakulla Porphyromonas gingivalis streptococcus pneumoniae d39 Escherichia coli Ehrlichia chaffeensis liberty* | 7.57E-001 | 1 | 3.07E-01 | 5.00E-01 | 8.28E-01 | 8.00E-01 | 5.54E-01 | 6.41E-01 | 1.00E+00 | 8.32E-01 | 9.05E-01 | 8.00E-01 | 4.10E-01 | 2.88E-01 |
| 508 | 5 | 28 | *Helicobacter pylori kx2 npgec Bifidobacterium bifidum Pseudomonas aeruginosa Porphyromonas gingivalis Escherichia coli* | 7.57E-001 | 2 | 4.07E-02 | 5.00E-01 | 8.28E-01 | 8.00E-01 | 5.54E-01 | 6.41E-01 | 3.23E-01 | 8.32E-01 | 5.96E-01 | 8.00E-01 | 4.10E-01 | 1.00E+00 |
| 510 | 7 | 20 | *Yersinia enterocolitica wap bc Yersinia enterocolitica p60 bc Lactobacillus acidophilus Bifidobacterium bifidum streptococcus pyogenes Mycobacterium tuberculosis streptococcus pneumoniae d39* | 8.17E-001 |  | 1.00E+00 | 9.53E-01 | 2.05E-01 | 6.35E-02 | 9.99E-01 | 9.76E-01 | 6.71E-02 | 3.12E-01 | 8.03E-01 | 6.02E-01 | 1.00E+00 | 1.00E+00 |
| 512 | 20 | 7 | *Helicobacter pylori kx1 mgep Yersinia enterocolitica p60 bl6 Bifidobacterium bifidum Pseudomonas aeruginosa Ehrlichia chaffeensis arkansa Ehrlichia chaffeensis wakulla Brucella melitensis streptococcus pyogenes Helicobacter pylori kx2 mgep Yersinia enterocolitica wap bl6 Yersinia enterocolitica wap bc Helicobacter pylori kx2 npgec Yersinia enterocolitica p60 bc Lactobacillus acidophilus Aeromonas cavia Ehrlichia chaffeensis liberty Helicobacter pylori kx1 npgec Bacillus anthracis Escherichia coli Helicobacter pylori* | 3.69E-003 |  | 1.00E+00 | 1.00E+00 | 2.50E-04 | 8.90E-01 | 2.04E-01 | 9.96E-01 | 4.63E-01 | 2.96E-01 | 1.46E-02 | 1.00E+00 | 1.00E+00 | 4.35E-01 |
| 514 | 2 | 69 | *streptococcus gordonii Mycobacterium tuberculosis* | 7.21E-001 | 9 | 3.90E-02 | 2.44E-01 | 1.00E+00 | 4.60E-01 | 1.00E+00 | 6.23E-01 | 1.00E+00 | 4.95E-01 | 1.00E+00 | 4.60E-01 | 1.84E-01 | 1.00E+00 |
| 516 | 3 | 46 | *Pseudomonas aeruginosa fdr440 Helicobacter pylori kx2 mgep Aggregatibacter actinomycetemcomitans* | 7.00E-001 | 3 | 9.86E-02 | 5.00E-01 | 8.84E-01 | 1.00E+00 | 3.54E-01 | 4.88E-02 | 1.00E+00 | 1.00E+00 | 7.45E-01 | 6.08E-01 | 2.65E-01 | 1.00E+00 |
| 518 | 2 | 69 | *Pseudomonas aeruginosa fdr440 Helicobacter pylori* | 7.21E-001 | 7 | 4.55E-03 | 2.44E-01 | 1.00E+00 | 1.00E+00 | 5.05E-01 | 6.23E-01 | 1.00E+00 | 1.00E+00 | 5.92E-01 | 4.60E-01 | 1.00E+00 | 1.00E+00 |
| 520 | 6 | 23 | *Burkholderia pseudomallei Ehrlichia chaffeensis wakulla Lactobacillus acidophilus Bifidobacterium bifidum Helicobacter pylori Escherichia coli* | 8.02E-001 |  | 1.00E+00 | 6.69E-01 | 6.69E-01 | 5.03E-01 | 7.84E-01 | 1.00E+00 | 4.88E-02 | 5.61E-01 | 7.13E-01 | 1.00E+00 | 1.00E+00 | 3.82E-01 |
| 522 | 3 | 45 | *streptococcus pneumoniae g54 Fusobacterium nucleatum streptococcus pneumoniae tigr4* | 7.10E-001 |  | 1.00E+00 | 1.16E-01 | 1.00E+00 | 1.63E-01 | 9.81E-01 | 4.88E-02 | 1.00E+00 | 1.00E+00 | 1.00E+00 | 1.63E-01 | 2.65E-01 | 1.00E+00 |
| 524 | 6 | 22 | *streptococcus gordonii Pseudomonas aeruginosa fdr440 Helicobacter pylori kx2 mgep streptococcus pneumoniae tigr4 Shigella dysenteriae Escherichia coli* | 8.28E-001 |  | 1.00E+00 | 9.18E-02 | 9.90E-01 | 5.03E-01 | 7.84E-01 | 1.36E-01 | 1.00E+00 | 5.61E-01 | 3.61E-01 | 5.03E-01 | 4.74E-01 | 1.00E+00 |
| 526 | 3 | 44 | *Ehrlichia chaffeensis wakulla streptococcus pneumoniae g54 Helicobacter pylori* | 7.20E-001 |  | 1.00E+00 | 5.00E-01 | 8.84E-01 | 6.08E-01 | 8.08E-01 | 7.74E-01 | 1.00E+00 | 1.00E+00 | 7.45E-01 | 6.08E-01 | 1.00E+00 | 5.25E-01 |
| 528 | 4 | 33 | *Helicobacter pylori kx2 npgec Lactobacillus acidophilus Helicobacter pylori kx2 mgep Ehrlichia chaffeensis liberty* | 7.50E-001 | 4 | 7.97E-03 | 1.00E+00 | 5.35E-02 | 7.19E-01 | 6.80E-01 | 4.95E-01 | 2.65E-01 | 1.00E+00 | 4.51E-01 | 1.00E+00 | 1.00E+00 | 6.34E-01 |
| 530 | 6 | 22 | *Yersinia enterocolitica wap bl6 Ehrlichia chaffeensis arkansa Burkholderia pseudomallei Pseudomonas aeruginosa fdr1 Pseudomonas aeruginosa fdr440 Escherichia coli* | 8.28E-001 | 1 | 1.67E-01 | 3.31E-01 | 9.08E-01 | 1.00E+00 | 1.13E-01 | 7.55E-01 | 1.00E+00 | 2.16E-01 | 7.13E-01 | 5.03E-01 | 1.00E+00 | 3.82E-01 |
| 532 | 5 | 26 | *Ehrlichia chaffeensis arkansa Burkholderia pseudomallei Ehrlichia chaffeensis wakulla Aeromonas cavia Ehrlichia chaffeensis liberty* | 8.01E-001 | 3 | 2.68E-02 | 9.76E-01 | 1.72E-01 | 1.00E+00 | 1.68E-01 | 1.00E+00 | 1.00E+00 | 8.32E-01 | 9.05E-01 | 1.00E+00 | 1.00E+00 | 5.04E-03 |
| 534 | 8 | 16 | *Yersinia enterocolitica wap bl6 Yersinia enterocolitica p60 bl6 Yersinia enterocolitica p60 bc Ehrlichia chaffeensis wakulla Pseudomonas aeruginosa fdr1 streptococcus pneumoniae g54 Brucella ovis Brucella neotomae* | 8.83E-001 | 4 | 7.60E-04 | 9.78E-01 | 1.19E-01 | 9.33E-01 | 2.57E-01 | 8.98E-01 | 1.00E+00 | 3.09E-02 | 6.05E-01 | 6.88E-01 | 1.00E+00 | 2.19E-01 |
| 536 | 4 | 32 | *Brucella melitensis streptococcus pneumoniae g54 streptococcus pyogenes Helicobacter pylori kx1 npgec* | 7.67E-001 | 3 | 3.76E-02 | 9.47E-01 | 3.03E-01 | 2.77E-01 | 9.37E-01 | 4.95E-01 | 1.00E+00 | 7.55E-01 | 8.43E-01 | 7.19E-01 | 1.00E+00 | 6.34E-01 |
| 538 | 9 | 14 | *Helicobacter pylori kx1 mgep Yersinia enterocolitica p60 bl6 Yersinia enterocolitica p60 bc Listeria monocytogenes Ehrlichia chaffeensis wakulla streptococcus gordonii Pseudomonas aeruginosa fdr440 Mycobacterium tuberculosis Helicobacter pylori kx2 mgep* | 8.60E-001 |  | 1.00E+00 | 7.73E-01 | 5.00E-01 | 7.60E-01 | 7.84E-01 | 4.71E-01 | 5.25E-01 | 5.09E-01 | 1.56E-01 | 7.60E-01 | 6.34E-01 | 9.14E-01 |
| 540 | 21 | 6 | *Helicobacter pylori kx1 mgep Yersinia enterocolitica p60 bl6 Listeria monocytogenes Pseudomonas aeruginosa streptococcus pneumoniae tigr4 Ehrlichia chaffeensis arkansa Ehrlichia chaffeensis wakulla Pseudomonas aeruginosa fdr440 Mycobacterium tuberculosis Helicobacter pylori kx2 mgep Yersinia enterocolitica wap bl6 Yersinia enterocolitica wap bc Yersinia enterocolitica p60 bc Lactobacillus acidophilus Aeromonas cavia Helicobacter pylori kx1 npgec Bacillus anthracis streptococcus pneumoniae g54 Escherichia coli Helicobacter pylori Shigella dysenteriae* | 3.52E-003 |  | 1.00E+00 | 7.31E-01 | 5.00E-01 | 7.58E-01 | 6.33E-01 | 9.45E-01 | 5.00E-01 | 1.53E-01 | 4.42E-03 | 7.58E-01 | 1.00E+00 | 9.35E-01 |
| 542 | 6 | 21 | *Burkholderia pseudomallei Yersinia enterocolitica p60 bl6 Brucella neotomae streptococcus pneumoniae tigr4 Shigella dysenteriae Aggregatibacter actinomycetemcomitans* | 8.54E-001 |  | 1.00E+00 | 3.31E-01 | 9.08E-01 | 8.60E-01 | 4.39E-01 | 7.55E-01 | 1.00E+00 | 4.58E-02 | 7.13E-01 | 8.60E-01 | 4.74E-01 | 3.82E-01 |
| 544 | 9 | 14 | *Yersinia enterocolitica wap bc Listeria monocytogenes Pseudomonas aeruginosa streptococcus pneumoniae tigr4 streptococcus pneumoniae d39 streptococcus gordonii Bacillus anthracis Mycobacterium tuberculosis Shigella dysenteriae* | 8.60E-001 |  | 1.00E+00 | 1.02E-02 | 9.99E-01 | 3.76E-02 | 9.99E-01 | 7.60E-01 | 5.25E-01 | 2.16E-01 | 7.06E-01 | 1.63E-01 | 6.34E-01 | 9.14E-01 |
| 546 | 5 | 25 | *Yersinia enterocolitica wap bl6 Pseudomonas aeruginosa fdr1 Listeria monocytogenes streptococcus pyogenes Mycobacterium tuberculosis* | 8.23E-001 | 2 | 4.07E-02 | 5.00E-01 | 8.28E-01 | 3.93E-01 | 9.82E-01 | 9.23E-01 | 3.23E-01 | 4.46E-01 | 5.96E-01 | 3.93E-01 | 1.00E+00 | 1.00E+00 |
| 548 | 4 | 31 | *Yersinia enterocolitica wap bc Pseudomonas aeruginosa fdr1 streptococcus pneumoniae g54 Mycobacterium tuberculosis* | 7.83E-001 | 3 | 5.84E-03 | 3.03E-01 | 9.47E-01 | 7.19E-01 | 9.37E-01 | 4.95E-01 | 1.00E+00 | 3.20E-01 | 8.43E-01 | 4.87E-02 | 1.00E+00 | 1.00E+00 |
| 550 | 11 | 11 | *Yersinia enterocolitica wap bl6 Yersinia enterocolitica p60 bl6 Yersinia enterocolitica p60 bc Lactobacillus acidophilus Listeria monocytogenes Bifidobacterium bifidum Ehrlichia chaffeensis liberty Ehrlichia chaffeensis arkansa Ehrlichia chaffeensis wakulla streptococcus gordonii Escherichia coli* | 7.13E-001 |  | 1.00E+00 | 9.84E-01 | 7.95E-02 | 3.03E-01 | 8.54E-01 | 9.98E-01 | 1.44E-02 | 3.82E-01 | 3.33E-01 | 1.00E+00 | 7.19E-01 | 4.36E-01 |
| 552 | 5 | 24 | *Helicobacter pylori kx1 mgep Burkholderia pseudomallei streptococcus pyogenes Porphyromonas gingivalis Ehrlichia chaffeensis liberty* | 8.43E-001 | 1 | 1.67E-01 | 8.28E-01 | 5.00E-01 | 8.00E-01 | 5.54E-01 | 6.41E-01 | 1.00E+00 | 8.32E-01 | 9.05E-01 | 1.00E+00 | 4.10E-01 | 2.88E-01 |
| 554 | 4 | 30 | *Yersinia enterocolitica p60 bl6 Listeria monocytogenes Porphyromonas gingivalis Helicobacter pylori* | 7.99E-001 |  | 1.00E+00 | 3.03E-01 | 9.47E-01 | 7.19E-01 | 6.80E-01 | 8.66E-01 | 2.65E-01 | 7.55E-01 | 1.22E-01 | 1.00E+00 | 3.41E-01 | 1.00E+00 |
| 556 | 8 | 15 | *Yersinia enterocolitica wap bl6 Yersinia enterocolitica p60 bl6 Yersinia enterocolitica p60 bc Lactobacillus acidophilus Aeromonas cavia streptococcus pneumoniae d39 Ehrlichia chaffeensis arkansa Ehrlichia chaffeensis wakulla* | 9.12E-001 |  | 1.00E+00 | 9.98E-01 | 2.24E-02 | 6.88E-01 | 5.89E-01 | 9.87E-01 | 4.79E-01 | 4.11E-01 | 2.94E-01 | 9.33E-01 | 1.00E+00 | 5.57E-01 |
| 558 | 7 | 17 | *Yersinia enterocolitica wap bc Ehrlichia chaffeensis arkansa Yersinia enterocolitica p60 bl6 Yersinia enterocolitica p60 bc Ehrlichia chaffeensis wakulla streptococcus pneumoniae g54 Fusobacterium nucleatum* | 9.04E-001 |  | 1.00E+00 | 9.53E-01 | 2.05E-01 | 9.03E-01 | 3.40E-01 | 8.39E-01 | 1.00E+00 | 3.12E-01 | 4.88E-01 | 9.03E-01 | 5.32E-01 | 4.72E-01 |
| 560 | 7 | 17 | *Yersinia enterocolitica wap bc Ehrlichia chaffeensis arkansa Yersinia enterocolitica p60 bc streptococcus pneumoniae g54 Porphyromonas gingivalis Mycobacterium tuberculosis Aeromonas cavia* | 9.04E-001 |  | 1.00E+00 | 7.95E-01 | 5.00E-01 | 9.03E-01 | 6.88E-01 | 8.39E-01 | 1.00E+00 | 3.12E-01 | 4.88E-01 | 6.02E-01 | 5.32E-01 | 8.42E-01 |
| 562 | 3 | 39 | *Helicobacter pylori kx1 mgep streptococcus pneumoniae g54 Aggregatibacter actinomycetemcomitans* | 7.72E-001 | 1 | 8.08E-01 | 5.00E-01 | 8.84E-01 | 6.08E-01 | 8.08E-01 | 4.88E-02 | 1.00E+00 | 1.00E+00 | 7.45E-01 | 6.08E-01 | 2.65E-01 | 1.00E+00 |
| 564 | 3 | 38 | *Mycobacterium tuberculosis streptococcus pneumoniae d39 Helicobacter pylori kx1 npgec* | 7.82E-001 | 1 | 5.61E-01 | 5.00E-01 | 8.84E-01 | 6.08E-01 | 9.81E-01 | 3.21E-01 | 1.00E+00 | 6.46E-01 | 7.45E-01 | 1.63E-01 | 1.00E+00 | 1.00E+00 |
| 566 | 6 | 19 | *Yersinia enterocolitica wap bc Yersinia enterocolitica p60 bc Pseudomonas aeruginosa fdr1 Porphyromonas gingivalis Helicobacter pylori kx2 mgep Escherichia coli* | 9.00E-001 | 2 | 7.65E-03 | 6.69E-01 | 6.69E-01 | 1.00E+00 | 1.13E-01 | 4.13E-01 | 1.00E+00 | 2.16E-01 | 1.08E-01 | 8.60E-01 | 4.74E-01 | 1.00E+00 |
| 568 | 10 | 11 | *Helicobacter pylori kx1 mgep Bifidobacterium bifidum Pseudomonas aeruginosa Fusobacterium nucleatum streptococcus pneumoniae tigr4 Ehrlichia chaffeensis wakulla streptococcus pneumoniae g54 Mycobacterium tuberculosis Helicobacter pylori kx2 mgep Escherichia coli* | 8.49E-001 |  | 1.00E+00 | 3.59E-01 | 8.61E-01 | 5.24E-01 | 9.04E-01 | 3.00E-01 | 5.68E-01 | 8.63E-01 | 7.88E-01 | 2.29E-01 | 6.79E-01 | 9.37E-01 |
| 570 | 6 | 18 | *Helicobacter pylori kx2 npgec Ehrlichia chaffeensis arkansa Lactobacillus acidophilus Mycobacterium tuberculosis Aeromonas cavia Escherichia coli* | 9.20E-001 |  | 1.00E+00 | 9.08E-01 | 3.31E-01 | 8.60E-01 | 7.84E-01 | 9.56E-01 | 3.78E-01 | 5.61E-01 | 3.61E-01 | 8.60E-01 | 1.00E+00 | 7.89E-01 |
| 572 | 2 | 53 | *Fusobacterium nucleatum Helicobacter pylori kx2 mgep* | 8.00E-001 |  | 1.00E+00 | 7.56E-01 | 7.56E-01 | 1.00E+00 | 5.05E-01 | 1.39E-01 | 1.00E+00 | 1.00E+00 | 5.92E-01 | 1.00E+00 | 1.84E-01 | 1.00E+00 |
| 574 | 2 | 53 | *Porphyromonas gingivalis Helicobacter pylori kx1 npgec* | 8.00E-001 | 10 | < 3.71E-07 | 7.56E-01 | 7.56E-01 | 1.00E+00 | 5.05E-01 | 1.39E-01 | 1.00E+00 | 1.00E+00 | 5.92E-01 | 1.00E+00 | 1.84E-01 | 1.00E+00 |
| 576 | 3 | 35 | *Yersinia enterocolitica wap bc Yersinia enterocolitica p60 bl6 streptococcus gordonii* | 8.11E-001 | 1 | 3.07E-01 | 8.84E-01 | 5.00E-01 | 6.08E-01 | 8.08E-01 | 7.74E-01 | 1.00E+00 | 1.92E-01 | 2.87E-01 | 1.00E+00 | 2.65E-01 | 1.00E+00 |
| 578 | 5 | 21 | *streptococcus gordonii streptococcus pneumoniae g54 Listeria monocytogenes streptococcus pneumoniae tigr4 Ehrlichia chaffeensis liberty* | 9.00E-001 |  | 1.00E+00 | 1.72E-01 | 9.76E-01 | 1.26E-02 | 9.99E-01 | 2.75E-01 | 3.23E-01 | 1.00E+00 | 9.05E-01 | 3.93E-01 | 4.10E-01 | 7.21E-01 |
| 580 | 4 | 26 | *Helicobacter pylori kx2 npgec streptococcus pneumoniae g54 streptococcus pyogenes Fusobacterium nucleatum* | 8.60E-001 |  | 1.00E+00 | 6.97E-01 | 6.97E-01 | 2.77E-01 | 9.37E-01 | 1.46E-01 | 1.00E+00 | 1.00E+00 | 8.43E-01 | 7.19E-01 | 3.41E-01 | 1.00E+00 |
| 582 | 4 | 26 | *streptococcus pyogenes streptococcus pneumoniae d39 Shigella dysenteriae Escherichia coli* | 8.60E-001 | 2 | 1.20E-01 | 3.03E-01 | 9.47E-01 | 2.77E-01 | 9.37E-01 | 8.66E-01 | 1.00E+00 | 3.20E-01 | 4.51E-01 | 7.19E-01 | 1.00E+00 | 1.00E+00 |
| 584 | 3 | 34 | *Listeria monocytogenes Bifidobacterium bifidum Fusobacterium nucleatum* | 8.20E-001 | 2 | 4.14E-01 | 5.00E-01 | 8.84E-01 | 1.63E-01 | 9.81E-01 | 7.74E-01 | 1.03E-02 | 1.00E+00 | 7.45E-01 | 1.00E+00 | 2.65E-01 | 1.00E+00 |
| 586 | 10 | 10 | *Yersinia enterocolitica wap bc Yersinia enterocolitica p60 bc Listeria monocytogenes Fusobacterium nucleatum Ehrlichia chaffeensis wakulla Bacillus anthracis Pseudomonas aeruginosa fdr440 Mycobacterium tuberculosis Escherichia coli Shigella dysenteriae* | 8.85E-001 |  | 1.00E+00 | 1.39E-01 | 9.67E-01 | 8.19E-01 | 7.04E-01 | 9.62E-01 | 5.68E-01 | 1.96E-02 | 2.39E-01 | 8.19E-01 | 6.79E-01 | 7.01E-01 |
| 588 | 5 | 20 | *Helicobacter pylori kx2 npgec Helicobacter pylori kx1 mgep Burkholderia pseudomallei Brucella melitensis Brucella ovis* | 9.16E-001 |  | 1.00E+00 | 9.76E-01 | 1.72E-01 | 1.00E+00 | 1.68E-01 | 6.41E-01 | 1.00E+00 | 1.31E-01 | 5.96E-01 | 1.00E+00 | 1.00E+00 | 5.72E-02 |
| 590 | 3 | 33 | *streptococcus gordonii Brucella melitensis Brucella ovis* | 8.30E-001 | 11 | 2.02E-03 | 8.84E-01 | 5.00E-01 | 6.08E-01 | 8.08E-01 | 7.74E-01 | 1.00E+00 | 1.92E-01 | 1.00E+00 | 1.00E+00 | 2.65E-01 | 1.11E-01 |
| 592 | 3 | 33 | *Helicobacter pylori kx1 mgep Brucella melitensis streptococcus pneumoniae d39* | 8.30E-001 | 1 | 4.73E-01 | 8.84E-01 | 5.00E-01 | 6.08E-01 | 8.08E-01 | 3.21E-01 | 1.00E+00 | 6.46E-01 | 7.45E-01 | 6.08E-01 | 1.00E+00 | 5.25E-01 |
| 594 | 3 | 33 | *streptococcus pneumoniae g54 Staphylococcus aureus Fusobacterium nucleatum* | 8.30E-001 | 15 | 1.34E-02 | 1.16E-01 | 1.00E+00 | 1.63E-01 | 9.81E-01 | 4.88E-02 | 1.00E+00 | 1.00E+00 | 1.00E+00 | 1.63E-01 | 2.65E-01 | 1.00E+00 |
| 596 | 7 | 14 | *Ehrlichia chaffeensis arkansa Ehrlichia chaffeensis wakulla Pseudomonas aeruginosa fdr1 Pseudomonas aeruginosa fdr440 Listeria monocytogenes Pseudomonas aeruginosa Aeromonas cavia* | 9.62E-001 | 1 | 8.75E-02 | 5.00E-01 | 7.95E-01 | 9.03E-01 | 3.40E-01 | 8.39E-01 | 4.30E-01 | 1.00E+00 | 8.03E-01 | 2.56E-01 | 1.00E+00 | 4.72E-01 |
| 598 | 12 | 8 | *Yersinia enterocolitica wap bl6 Yersinia enterocolitica p60 bl6 Lactobacillus acidophilus Bifidobacterium bifidum Porphyromonas gingivalis streptococcus pneumoniae d39 Ehrlichia chaffeensis arkansa Ehrlichia chaffeensis wakulla Pseudomonas aeruginosa fdr440 streptococcus pyogenes Escherichia coli Helicobacter pylori* | 7.67E-001 |  | 1.00E+00 | 8.47E-01 | 3.67E-01 | 3.82E-01 | 7.93E-01 | 9.30E-01 | 1.92E-01 | 7.54E-01 | 7.08E-01 | 9.03E-01 | 7.55E-01 | 8.10E-01 |
| 600 | 7 | 13 | *Yersinia enterocolitica wap bl6 Yersinia enterocolitica p60 bl6 streptococcus gordonii Ehrlichia chaffeensis wakulla Fusobacterium nucleatum Shigella dysenteriae Escherichia coli* | 9.74E-001 |  | 1.00E+00 | 5.00E-01 | 7.95E-01 | 9.03E-01 | 3.40E-01 | 8.39E-01 | 1.00E+00 | 8.83E-02 | 1.92E-01 | 1.00E+00 | 1.23E-01 | 8.42E-01 |
| 602 | 13 | 7 | *Yersinia enterocolitica wap bl6 Yersinia enterocolitica wap bc Yersinia enterocolitica p60 bc Eubacterium rectale Lactobacillus acidophilus Listeria monocytogenes Bifidobacterium bifidum Helicobacter pylori kx1 npgec Ehrlichia chaffeensis liberty Ehrlichia chaffeensis wakulla streptococcus pyogenes Mycobacterium tuberculosis Escherichia coli* | 7.12E-001 |  | 1.00E+00 | 9.97E-01 | 2.15E-02 | 2.01E-01 | 9.79E-01 | 1.00E+00 | 2.49E-02 | 2.77E-01 | 9.88E-02 | 9.92E-01 | 1.00E+00 | 8.52E-01 |
| 604 | 2 | 45 | *Pseudomonas aeruginosa fdr1 Brucella neotomae* | 8.32E-001 | 13 | < 3.71E-07 | 7.56E-01 | 7.56E-01 | 1.00E+00 | 5.05E-01 | 6.23E-01 | 1.00E+00 | 4.95E-01 | 1.00E+00 | 4.60E-01 | 1.00E+00 | 3.87E-01 |
| 606 | 3 | 30 | *Bacillus anthracis streptococcus pneumoniae g54 Aeromonas cavia* | 8.59E-001 |  | 1.00E+00 | 5.00E-01 | 8.84E-01 | 1.63E-01 | 9.81E-01 | 7.74E-01 | 1.00E+00 | 6.46E-01 | 7.45E-01 | 6.08E-01 | 1.00E+00 | 5.25E-01 |
| 608 | 15 | 6 | *Yersinia enterocolitica wap bl6 Yersinia enterocolitica wap bc Yersinia enterocolitica p60 bl6 Yersinia enterocolitica p60 bc Lactobacillus acidophilus Listeria monocytogenes Fusobacterium nucleatum streptococcus pneumoniae tigr4 Brucella melitensis streptococcus pneumoniae g54 Mycobacterium tuberculosis Brucella neotomae Helicobacter pylori kx2 mgep Shigella dysenteriae Escherichia coli* | 5.14E-001 |  | 1.00E+00 | 7.40E-01 | 5.00E-01 | 6.16E-01 | 8.07E-01 | 9.31E-01 | 2.87E-01 | 1.42E-03 | 7.56E-02 | 8.52E-01 | 8.43E-01 | 9.15E-01 |
| 610 | 6 | 15 | *Yersinia enterocolitica wap bc Ehrlichia chaffeensis arkansa streptococcus gordonii Pseudomonas aeruginosa fdr440 Mycobacterium tuberculosis Escherichia coli* | 9.65E-001 |  | 1.00E+00 | 3.31E-01 | 9.08E-01 | 8.60E-01 | 7.84E-01 | 7.55E-01 | 1.00E+00 | 2.16E-01 | 7.13E-01 | 5.03E-01 | 4.74E-01 | 7.89E-01 |
| 612 | 6 | 15 | *Eubacterium rectale streptococcus pneumoniae g54 Listeria monocytogenes Helicobacter pylori kx2 mgep Shigella dysenteriae Escherichia coli* | 9.65E-001 |  | 1.00E+00 | 3.31E-01 | 9.08E-01 | 1.73E-01 | 9.54E-01 | 7.55E-01 | 3.78E-01 | 5.61E-01 | 1.64E-02 | 8.60E-01 | 1.00E+00 | 1.00E+00 |
| 614 | 2 | 44 | *Helicobacter pylori Helicobacter pylori kx1 npgec* | 8.36E-001 | 1 | 4.23E-01 | 7.56E-01 | 7.56E-01 | 1.00E+00 | 5.05E-01 | 6.23E-01 | 1.00E+00 | 1.00E+00 | 1.22E-01 | 1.00E+00 | 1.00E+00 | 1.00E+00 |
| 616 | 4 | 22 | *Ehrlichia chaffeensis arkansa Listeria monocytogenes Fusobacterium nucleatum Shigella dysenteriae* | 9.15E-001 |  | 1.00E+00 | 3.03E-01 | 9.47E-01 | 7.19E-01 | 6.80E-01 | 8.66E-01 | 2.65E-01 | 7.55E-01 | 4.51E-01 | 1.00E+00 | 3.41E-01 | 6.34E-01 |
| 618 | 4 | 22 | *Yersinia enterocolitica wap bc Ehrlichia chaffeensis arkansa Porphyromonas gingivalis Aggregatibacter actinomycetemcomitans* | 9.15E-001 | 3 | 2.68E-02 | 6.97E-01 | 6.97E-01 | 1.00E+00 | 2.45E-01 | 4.95E-01 | 1.00E+00 | 7.55E-01 | 8.43E-01 | 1.00E+00 | 3.91E-02 | 6.34E-01 |
| 620 | 5 | 17 | *Ehrlichia chaffeensis wakulla Pseudomonas aeruginosa fdr440 Listeria monocytogenes streptococcus pneumoniae d39 Aggregatibacter actinomycetemcomitans* | 9.57E-001 |  | 1.00E+00 | 1.72E-01 | 9.76E-01 | 3.93E-01 | 8.69E-01 | 2.75E-01 | 3.23E-01 | 1.00E+00 | 9.05E-01 | 3.93E-01 | 4.10E-01 | 7.21E-01 |
| 622 | 5 | 17 | *Yersinia enterocolitica wap bc streptococcus gordonii streptococcus pneumoniae g54 Listeria monocytogenes Fusobacterium nucleatum* | 9.57E-001 |  | 1.00E+00 | 1.72E-01 | 9.76E-01 | 1.03E-01 | 9.82E-01 | 2.75E-01 | 3.23E-01 | 8.32E-01 | 5.96E-01 | 8.00E-01 | 6.29E-02 | 1.00E+00 |
| 624 | 5 | 17 | *Ehrlichia chaffeensis arkansa Ehrlichia chaffeensis wakulla Mycobacterium tuberculosis Fusobacterium nucleatum Ehrlichia chaffeensis liberty* | 9.57E-001 |  | 1.00E+00 | 8.28E-01 | 5.00E-01 | 1.00E+00 | 5.54E-01 | 9.23E-01 | 1.00E+00 | 8.32E-01 | 1.00E+00 | 8.00E-01 | 4.10E-01 | 5.72E-02 |
| 626 | 3 | 28 | *streptococcus gordonii Pseudomonas aeruginosa Helicobacter pylori kx2 mgep* | 8.80E-001 | 1 | 2.40E-01 | 5.00E-01 | 8.84E-01 | 6.08E-01 | 8.08E-01 | 3.21E-01 | 1.00E+00 | 1.00E+00 | 7.45E-01 | 6.08E-01 | 2.65E-01 | 1.00E+00 |
| 628 | 9 | 9 | *Yersinia enterocolitica wap bc Yersinia enterocolitica p60 bc Pseudomonas aeruginosa Fusobacterium nucleatum streptococcus pneumoniae tigr4 streptococcus pneumoniae d39 Aggregatibacter actinomycetemcomitans Ehrlichia chaffeensis wakulla Shigella dysenteriae* | 9.64E-001 |  | 1.00E+00 | 2.27E-01 | 9.35E-01 | 7.60E-01 | 4.91E-01 | 4.71E-01 | 1.00E+00 | 5.09E-01 | 7.06E-01 | 4.36E-01 | 1.96E-01 | 9.14E-01 |
| 630 | 3 | 27 | *Ehrlichia chaffeensis arkansa Pseudomonas aeruginosa fdr1 Brucella melitensis* | 8.90E-001 | 12 | < 3.71E-07 | 8.84E-01 | 5.00E-01 | 1.00E+00 | 3.54E-01 | 7.74E-01 | 1.00E+00 | 6.46E-01 | 1.00E+00 | 6.08E-01 | 1.00E+00 | 1.11E-01 |
| 632 | 5 | 16 | *streptococcus gordonii Ehrlichia chaffeensis wakulla Lactobacillus acidophilus streptococcus pneumoniae g54 Mycobacterium tuberculosis* | 9.67E-001 |  | 1.00E+00 | 5.00E-01 | 8.28E-01 | 1.03E-01 | 9.99E-01 | 6.41E-01 | 3.23E-01 | 8.32E-01 | 1.00E+00 | 3.93E-01 | 4.10E-01 | 7.21E-01 |
| 634 | 10 | 8 | *Helicobacter pylori kx1 mgep Yersinia enterocolitica p60 bc Listeria monocytogenes Aggregatibacter actinomycetemcomitans Ehrlichia chaffeensis arkansa Ehrlichia chaffeensis wakulla streptococcus gordonii Mycobacterium tuberculosis Helicobacter pylori kx2 mgep Escherichia coli* | 9.41E-001 |  | 1.00E+00 | 6.41E-01 | 6.41E-01 | 8.19E-01 | 7.04E-01 | 5.85E-01 | 5.68E-01 | 6.00E-01 | 2.39E-01 | 9.70E-01 | 2.36E-01 | 7.01E-01 |
| 636 | 20 | 4 | *Helicobacter pylori kx1 mgep Yersinia enterocolitica p60 bl6 Listeria monocytogenes Pseudomonas aeruginosa Fusobacterium nucleatum streptococcus pneumoniae tigr4 Ehrlichia chaffeensis arkansa Ehrlichia chaffeensis wakulla Mycobacterium tuberculosis Helicobacter pylori kx2 mgep Yersinia enterocolitica wap bl6 Yersinia enterocolitica wap bc Burkholderia pseudomallei Helicobacter pylori kx2 npgec Ehrlichia chaffeensis liberty streptococcus pneumoniae d39 Bacillus anthracis streptococcus pneumoniae g54 Escherichia coli Shigella dysenteriae* | 1.33E-001 |  | 1.00E+00 | 3.79E-01 | 8.23E-01 | 6.97E-01 | 7.04E-01 | 7.61E-01 | 8.66E-01 | 1.11E-01 | 1.91E-01 | 6.97E-01 | 9.35E-01 | 4.35E-01 |
| 638 | 7 | 11 | *Yersinia enterocolitica p60 bl6 Listeria monocytogenes Porphyromonas gingivalis Mycobacterium tuberculosis Fusobacterium nucleatum Helicobacter pylori kx2 mgep streptococcus pneumoniae d39* | 9.90E-001 |  | 1.00E+00 | 2.05E-01 | 9.53E-01 | 6.02E-01 | 9.12E-01 | 2.36E-01 | 4.30E-01 | 6.60E-01 | 4.88E-01 | 6.02E-01 | 1.23E-01 | 1.00E+00 |
| 640 | 5 | 15 | *Burkholderia pseudomallei Ehrlichia chaffeensis wakulla Pseudomonas aeruginosa fdr1 Pseudomonas aeruginosa Porphyromonas gingivalis* | 9.76E-001 |  | 1.00E+00 | 1.72E-01 | 9.76E-01 | 1.00E+00 | 1.68E-01 | 6.41E-01 | 1.00E+00 | 8.32E-01 | 1.00E+00 | 3.93E-01 | 4.10E-01 | 2.88E-01 |
| 642 | 5 | 15 | *Lactobacillus acidophilus streptococcus pneumoniae g54 Pseudomonas aeruginosa Aeromonas cavia Aggregatibacter actinomycetemcomitans* | 9.76E-001 |  | 1.00E+00 | 5.00E-01 | 8.28E-01 | 3.93E-01 | 8.69E-01 | 6.41E-01 | 3.23E-01 | 1.00E+00 | 9.05E-01 | 3.93E-01 | 4.10E-01 | 1.00E+00 |
| 644 | 5 | 15 | *Pseudomonas aeruginosa fdr440 Listeria monocytogenes Fusobacterium nucleatum Escherichia coli Ehrlichia chaffeensis liberty* | 9.76E-001 |  | 1.00E+00 | 1.72E-01 | 9.76E-01 | 8.00E-01 | 5.54E-01 | 6.41E-01 | 3.23E-01 | 8.32E-01 | 5.96E-01 | 8.00E-01 | 4.10E-01 | 7.21E-01 |
| 646 | 2 | 37 | *streptococcus gordonii Bifidobacterium bifidum* | 8.69E-001 | 4 | 3.67E-01 | 7.56E-01 | 7.56E-01 | 6.39E-02 | 1.00E+00 | 6.23E-01 | 1.39E-01 | 1.00E+00 | 1.00E+00 | 1.00E+00 | 1.84E-01 | 1.00E+00 |
| 648 | 9 | 8 | *Yersinia enterocolitica wap bl6 Yersinia enterocolitica wap bc Yersinia enterocolitica p60 bl6 Yersinia enterocolitica p60 bc Brucella ovis Ehrlichia chaffeensis wakulla streptococcus gordonii Bacillus anthracis Mycobacterium tuberculosis* | 9.76E-001 |  | 1.00E+00 | 9.35E-01 | 2.27E-01 | 7.60E-01 | 7.84E-01 | 9.93E-01 | 1.00E+00 | 8.10E-04 | 4.04E-01 | 9.55E-01 | 6.34E-01 | 2.88E-01 |
| 650 | 8 | 9 | *Yersinia enterocolitica wap bl6 Helicobacter pylori kx2 npgec Yersinia enterocolitica p60 bc Ehrlichia chaffeensis arkansa Ehrlichia chaffeensis wakulla Pseudomonas aeruginosa fdr440 Mycobacterium tuberculosis Helicobacter pylori* | 9.87E-001 |  | 1.00E+00 | 8.81E-01 | 3.48E-01 | 1.00E+00 | 2.57E-01 | 8.98E-01 | 1.00E+00 | 4.11E-01 | 2.94E-01 | 6.88E-01 | 1.00E+00 | 5.57E-01 |
| 652 | 6 | 12 | *Yersinia enterocolitica wap bl6 Yersinia enterocolitica p60 bl6 Yersinia enterocolitica p60 bc Pseudomonas aeruginosa Fusobacterium nucleatum Helicobacter pylori kx1 npgec* | 9.89E-001 |  | 1.00E+00 | 9.08E-01 | 3.31E-01 | 1.00E+00 | 1.13E-01 | 7.55E-01 | 1.00E+00 | 2.16E-01 | 1.08E-01 | 8.60E-01 | 4.74E-01 | 1.00E+00 |
| 654 | 6 | 12 | *Ehrlichia chaffeensis arkansa streptococcus gordonii Ehrlichia chaffeensis wakulla streptococcus pyogenes Mycobacterium tuberculosis Escherichia coli* | 9.89E-001 |  | 1.00E+00 | 6.69E-01 | 6.69E-01 | 5.03E-01 | 9.54E-01 | 9.56E-01 | 1.00E+00 | 5.61E-01 | 9.44E-01 | 8.60E-01 | 4.74E-01 | 3.82E-01 |
| 656 | 5 | 14 | *Pseudomonas aeruginosa fdr440 Pseudomonas aeruginosa Fusobacterium nucleatum streptococcus pneumoniae tigr4 Escherichia coli* | 9.83E-001 |  | 1.00E+00 | 2.39E-02 | 1.00E+00 | 8.00E-01 | 5.54E-01 | 2.75E-01 | 1.00E+00 | 8.32E-01 | 9.05E-01 | 1.03E-01 | 4.10E-01 | 1.00E+00 |
| 658 | 4 | 17 | *Pseudomonas aeruginosa fdr1 Pseudomonas aeruginosa streptococcus pneumoniae d39 Escherichia coli* | 9.68E-001 |  | 1.00E+00 | 5.35E-02 | 1.00E+00 | 7.19E-01 | 6.80E-01 | 4.95E-01 | 1.00E+00 | 7.55E-01 | 8.43E-01 | 4.87E-02 | 1.00E+00 | 1.00E+00 |
| 660 | 6 | 11 | *Yersinia enterocolitica p60 bl6 Yersinia enterocolitica p60 bc streptococcus gordonii Brucella melitensis Brucella neotomae Escherichia coli* | 9.94E-001 |  | 1.00E+00 | 9.08E-01 | 3.31E-01 | 8.60E-01 | 4.39E-01 | 9.56E-01 | 1.00E+00 | 4.71E-03 | 3.61E-01 | 1.00E+00 | 4.74E-01 | 3.82E-01 |
| 662 | 11 | 6 | *Lactobacillus acidophilus Listeria monocytogenes Bifidobacterium bifidum Fusobacterium nucleatum Brucella ovis Ehrlichia chaffeensis arkansa streptococcus gordonii streptococcus pneumoniae g54 Mycobacterium tuberculosis Brucella neotomae Escherichia coli* | 9.45E-001 |  | 1.00E+00 | 5.00E-01 | 7.58E-01 | 1.00E-01 | 9.94E-01 | 8.91E-01 | 1.44E-02 | 3.82E-01 | 9.67E-01 | 8.66E-01 | 2.77E-01 | 4.36E-01 |
| 664 | 13 | 5 | *Yersinia enterocolitica wap bc Yersinia enterocolitica p60 bl6 Yersinia enterocolitica p60 bc Listeria monocytogenes Bifidobacterium bifidum Pseudomonas aeruginosa Fusobacterium nucleatum Ehrlichia chaffeensis arkansa Ehrlichia chaffeensis wakulla streptococcus pyogenes Mycobacterium tuberculosis Shigella dysenteriae Escherichia coli* | 8.70E-001 |  | 1.00E+00 | 7.47E-01 | 5.00E-01 | 7.49E-01 | 7.23E-01 | 1.00E+00 | 2.22E-01 | 9.52E-02 | 2.73E-01 | 9.31E-01 | 7.88E-01 | 8.52E-01 |
| 666 | 4 | 16 | *Yersinia enterocolitica p60 bc Ehrlichia chaffeensis wakulla Fusobacterium nucleatum Brucella neotomae* | 9.75E-001 | 4 | 7.97E-03 | 9.47E-01 | 3.03E-01 | 1.00E+00 | 2.45E-01 | 8.66E-01 | 1.00E+00 | 3.20E-01 | 8.43E-01 | 1.00E+00 | 3.41E-01 | 1.96E-01 |
| 668 | 4 | 16 | *Bacillus anthracis streptococcus pneumoniae g54 Fusobacterium nucleatum Escherichia coli* | 9.75E-001 |  | 1.00E+00 | 5.35E-02 | 1.00E+00 | 2.77E-01 | 9.37E-01 | 4.95E-01 | 1.00E+00 | 3.20E-01 | 8.43E-01 | 7.19E-01 | 3.41E-01 | 6.34E-01 |
| 670 | 4 | 16 | *Yersinia enterocolitica p60 bl6 Lactobacillus acidophilus Bifidobacterium bifidum Fusobacterium nucleatum* | 9.75E-001 |  | 1.00E+00 | 9.47E-01 | 3.03E-01 | 2.77E-01 | 9.37E-01 | 8.66E-01 | 2.02E-02 | 7.55E-01 | 8.43E-01 | 1.00E+00 | 3.41E-01 | 1.00E+00 |
| 672 | 3 | 21 | *Helicobacter pylori kx2 npgec Ehrlichia chaffeensis arkansa streptococcus gordonii* | 9.46E-001 |  | 1.00E+00 | 8.84E-01 | 5.00E-01 | 6.08E-01 | 8.08E-01 | 3.21E-01 | 1.00E+00 | 1.00E+00 | 7.45E-01 | 1.00E+00 | 2.65E-01 | 5.25E-01 |
| 674 | 7 | 9 | *Yersinia enterocolitica p60 bl6 Brucella melitensis Listeria monocytogenes Fusobacterium nucleatum Brucella ovis Brucella neotomae Shigella dysenteriae* | 9.96E-001 |  | 1.00E+00 | 7.95E-01 | 5.00E-01 | 9.03E-01 | 3.40E-01 | 9.76E-01 | 4.30E-01 | 1.38E-02 | 4.88E-01 | 1.00E+00 | 5.32E-01 | 1.55E-01 |
| 676 | 3 | 21 | *streptococcus gordonii Staphylococcus aureus Escherichia coli* | 9.46E-001 |  | 1.00E+00 | 1.16E-01 | 1.00E+00 | 1.63E-01 | 9.81E-01 | 3.21E-01 | 1.00E+00 | 6.46E-01 | 7.45E-01 | 6.08E-01 | 2.65E-01 | 1.00E+00 |
| 678 | 3 | 20 | *streptococcus gordonii Ehrlichia chaffeensis wakulla Aeromonas cavia* | 9.54E-001 | 16 | < 3.71E-07 | 8.84E-01 | 5.00E-01 | 6.08E-01 | 8.08E-01 | 7.74E-01 | 1.00E+00 | 1.00E+00 | 7.45E-01 | 1.00E+00 | 2.65E-01 | 5.25E-01 |
| 680 | 10 | 6 | *Yersinia enterocolitica wap bc Listeria monocytogenes Ehrlichia chaffeensis liberty Ehrlichia chaffeensis arkansa Ehrlichia chaffeensis wakulla Pseudomonas aeruginosa fdr1 streptococcus pneumoniae g54 streptococcus pyogenes Mycobacterium tuberculosis Helicobacter pylori* | 9.75E-001 |  | 1.00E+00 | 6.41E-01 | 6.41E-01 | 5.24E-01 | 9.04E-01 | 9.62E-01 | 5.68E-01 | 8.63E-01 | 7.88E-01 | 5.24E-01 | 1.00E+00 | 3.62E-01 |
| 682 | 4 | 15 | *Burkholderia pseudomallei Ehrlichia chaffeensis wakulla Mycobacterium tuberculosis Fusobacterium nucleatum* | 9.81E-001 |  | 1.00E+00 | 3.03E-01 | 9.47E-01 | 1.00E+00 | 6.80E-01 | 8.66E-01 | 1.00E+00 | 3.20E-01 | 1.00E+00 | 7.19E-01 | 3.41E-01 | 1.96E-01 |
| 684 | 12 | 5 | *Yersinia enterocolitica wap bl6 Yersinia enterocolitica wap bc Yersinia enterocolitica p60 bl6 Listeria monocytogenes Pseudomonas aeruginosa streptococcus pneumoniae tigr4 Ehrlichia chaffeensis arkansa Ehrlichia chaffeensis wakulla streptococcus gordonii streptococcus pneumoniae g54 streptococcus pyogenes Shigella dysenteriae* | 9.30E-001 |  | 1.00E+00 | 6.33E-01 | 6.33E-01 | 1.46E-01 | 9.39E-01 | 9.30E-01 | 6.46E-01 | 4.69E-01 | 4.34E-01 | 6.82E-01 | 7.55E-01 | 8.10E-01 |
| 686 | 3 | 19 | *streptococcus gordonii Brucella neotomae streptococcus pneumoniae tigr4* | 9.60E-001 |  | 1.00E+00 | 5.00E-01 | 8.84E-01 | 1.63E-01 | 9.81E-01 | 3.21E-01 | 1.00E+00 | 6.46E-01 | 1.00E+00 | 6.08E-01 | 2.65E-01 | 5.25E-01 |
| 688 | 3 | 19 | *streptococcus gordonii Pseudomonas aeruginosa fdr1 streptococcus pneumoniae g54* | 9.60E-001 | 15 | 5.60E-04 | 1.16E-01 | 1.00E+00 | 1.63E-01 | 9.81E-01 | 4.88E-02 | 1.00E+00 | 1.00E+00 | 1.00E+00 | 1.63E-01 | 2.65E-01 | 1.00E+00 |
| 690 | 3 | 19 | *streptococcus gordonii Ehrlichia chaffeensis wakulla streptococcus pneumoniae d39* | 9.60E-001 |  | 1.00E+00 | 5.00E-01 | 8.84E-01 | 1.63E-01 | 9.81E-01 | 3.21E-01 | 1.00E+00 | 1.00E+00 | 1.00E+00 | 6.08E-01 | 2.65E-01 | 5.25E-01 |
| 692 | 8 | 7 | *Yersinia enterocolitica wap bl6 Yersinia enterocolitica p60 bl6 Yersinia enterocolitica p60 bc Ehrlichia chaffeensis wakulla Pseudomonas aeruginosa fdr440 Fusobacterium nucleatum Escherichia coli Aggregatibacter actinomycetemcomitans* | 9.94E-001 |  | 1.00E+00 | 6.52E-01 | 6.52E-01 | 1.00E+00 | 4.96E-02 | 6.64E-01 | 1.00E+00 | 1.46E-01 | 2.94E-01 | 9.33E-01 | 1.58E-01 | 8.82E-01 |
| 694 | 4 | 14 | *Helicobacter pylori kx1 mgep Yersinia enterocolitica p60 bl6 streptococcus gordonii Bacillus anthracis* | 9.86E-001 |  | 1.00E+00 | 6.97E-01 | 6.97E-01 | 2.77E-01 | 9.37E-01 | 4.95E-01 | 1.00E+00 | 3.20E-01 | 4.51E-01 | 1.00E+00 | 3.41E-01 | 6.34E-01 |
| 696 | 7 | 8 | *Ehrlichia chaffeensis arkansa streptococcus gordonii Ehrlichia chaffeensis wakulla Pseudomonas aeruginosa Porphyromonas gingivalis Mycobacterium tuberculosis Fusobacterium nucleatum* | 9.97E-001 |  | 1.00E+00 | 2.05E-01 | 9.53E-01 | 9.03E-01 | 6.88E-01 | 5.46E-01 | 1.00E+00 | 9.25E-01 | 1.00E+00 | 6.02E-01 | 1.13E-02 | 4.72E-01 |
| 698 | 5 | 11 | *Yersinia enterocolitica wap bl6 Ehrlichia chaffeensis arkansa Ehrlichia chaffeensis wakulla streptococcus pyogenes Fusobacterium nucleatum* | 9.95E-001 |  | 1.00E+00 | 9.76E-01 | 1.72E-01 | 8.00E-01 | 5.54E-01 | 9.23E-01 | 1.00E+00 | 8.32E-01 | 9.05E-01 | 1.00E+00 | 4.10E-01 | 2.88E-01 |
| 700 | 5 | 11 | *Ehrlichia chaffeensis arkansa streptococcus gordonii Lactobacillus acidophilus Listeria monocytogenes streptococcus pyogenes* | 9.95E-001 |  | 1.00E+00 | 8.28E-01 | 5.00E-01 | 1.26E-02 | 9.99E-01 | 9.23E-01 | 3.31E-02 | 1.00E+00 | 9.05E-01 | 1.00E+00 | 4.10E-01 | 7.21E-01 |
| 702 | 3 | 18 | *Brucella melitensis Fusobacterium nucleatum streptococcus pneumoniae tigr4* | 9.67E-001 |  | 1.00E+00 | 5.00E-01 | 8.84E-01 | 6.08E-01 | 8.08E-01 | 3.21E-01 | 1.00E+00 | 6.46E-01 | 1.00E+00 | 6.08E-01 | 2.65E-01 | 5.25E-01 |
| 704 | 3 | 18 | *Burkholderia pseudomallei streptococcus gordonii Escherichia coli* | 9.67E-001 |  | 1.00E+00 | 1.16E-01 | 1.00E+00 | 6.08E-01 | 8.08E-01 | 7.74E-01 | 1.00E+00 | 1.92E-01 | 7.45E-01 | 1.00E+00 | 2.65E-01 | 5.25E-01 |
| 706 | 18 | 3 | *Helicobacter pylori kx1 mgep Yersinia enterocolitica p60 bl6 Listeria monocytogenes Pseudomonas aeruginosa streptococcus pneumoniae tigr4 Ehrlichia chaffeensis arkansa streptococcus gordonii Ehrlichia chaffeensis wakulla Pseudomonas aeruginosa fdr1 Pseudomonas aeruginosa fdr440 Mycobacterium tuberculosis Helicobacter pylori kx2 mgep Yersinia enterocolitica wap bl6 Yersinia enterocolitica wap bc Yersinia enterocolitica p60 bc streptococcus pneumoniae g54 Escherichia coli Shigella dysenteriae* | 5.70E-001 |  | 1.00E+00 | 3.78E-01 | 8.25E-01 | 8.04E-01 | 6.00E-01 | 5.89E-01 | 8.24E-01 | 1.74E-01 | 8.90E-02 | 2.87E-01 | 9.05E-01 | 9.67E-01 |
| 708 | 13 | 4 | *Yersinia enterocolitica wap bl6 Yersinia enterocolitica wap bc Yersinia enterocolitica p60 bl6 Yersinia enterocolitica p60 bc Lactobacillus acidophilus Listeria monocytogenes Aeromonas cavia Ehrlichia chaffeensis arkansa Ehrlichia chaffeensis wakulla streptococcus gordonii Bacillus anthracis Mycobacterium tuberculosis Escherichia coli* | 9.21E-001 |  | 1.00E+00 | 9.10E-01 | 2.53E-01 | 4.62E-01 | 9.05E-01 | 1.00E+00 | 2.22E-01 | 2.14E-02 | 9.88E-02 | 9.92E-01 | 7.88E-01 | 5.79E-01 |
| 710 | 5 | 10 | *Ehrlichia chaffeensis wakulla Pseudomonas aeruginosa fdr1 streptococcus pneumoniae g54 Brucella neotomae Aeromonas cavia* | 9.97E-001 | 3 | 2.50E-03 | 8.28E-01 | 5.00E-01 | 8.00E-01 | 5.54E-01 | 6.41E-01 | 1.00E+00 | 8.32E-01 | 9.05E-01 | 3.93E-01 | 1.00E+00 | 2.88E-01 |
| 712 | 5 | 10 | *Listeria monocytogenes Fusobacterium nucleatum streptococcus pneumoniae tigr4 Helicobacter pylori kx1 npgec Escherichia coli* | 9.97E-001 |  | 1.00E+00 | 1.72E-01 | 9.76E-01 | 3.93E-01 | 8.69E-01 | 2.75E-01 | 3.23E-01 | 8.32E-01 | 2.35E-01 | 8.00E-01 | 4.10E-01 | 1.00E+00 |
| 714 | 7 | 7 | *streptococcus pneumoniae g54 Listeria monocytogenes streptococcus pyogenes Mycobacterium tuberculosis Fusobacterium nucleatum streptococcus pneumoniae tigr4 Shigella dysenteriae* | 9.98E-001 |  | 1.00E+00 | 4.66E-02 | 9.96E-01 | 6.35E-02 | 9.99E-01 | 5.46E-01 | 4.30E-01 | 6.60E-01 | 8.03E-01 | 2.56E-01 | 5.32E-01 | 1.00E+00 |
| 716 | 6 | 8 | *Yersinia enterocolitica wap bl6 Ehrlichia chaffeensis arkansa Ehrlichia chaffeensis wakulla Fusobacterium nucleatum Brucella ovis Shigella dysenteriae* | 9.99E-001 |  | 1.00E+00 | 9.08E-01 | 3.31E-01 | 1.00E+00 | 1.13E-01 | 9.56E-01 | 1.00E+00 | 2.16E-01 | 7.13E-01 | 1.00E+00 | 4.74E-01 | 1.01E-01 |
| 718 | 6 | 8 | *Ehrlichia chaffeensis wakulla Lactobacillus acidophilus Brucella melitensis Bifidobacterium bifidum Fusobacterium nucleatum Brucella neotomae* | 9.99E-001 |  | 1.00E+00 | 9.90E-01 | 9.18E-02 | 5.03E-01 | 7.84E-01 | 9.56E-01 | 4.88E-02 | 5.61E-01 | 1.00E+00 | 1.00E+00 | 4.74E-01 | 1.01E-01 |
| 720 | 4 | 12 | *Helicobacter pylori kx1 mgep Fusobacterium nucleatum Helicobacter pylori kx2 mgep Ehrlichia chaffeensis liberty* | 9.93E-001 |  | 1.00E+00 | 9.47E-01 | 3.03E-01 | 1.00E+00 | 2.45E-01 | 1.46E-01 | 1.00E+00 | 1.00E+00 | 4.51E-01 | 1.00E+00 | 3.41E-01 | 6.34E-01 |
| 722 | 6 | 8 | *Yersinia enterocolitica wap bc Ehrlichia chaffeensis wakulla Fusobacterium nucleatum Shigella dysenteriae Helicobacter pylori kx1 npgec Ehrlichia chaffeensis liberty* | 9.99E-001 |  | 1.00E+00 | 9.08E-01 | 3.31E-01 | 1.00E+00 | 1.13E-01 | 7.55E-01 | 1.00E+00 | 5.61E-01 | 3.61E-01 | 1.00E+00 | 4.74E-01 | 3.82E-01 |
| 724 | 2 | 23 | *Helicobacter pylori kx2 npgec Aggregatibacter actinomycetemcomitans* | 9.45E-001 | 1 | 7.69E-01 | 7.56E-01 | 7.56E-01 | 1.00E+00 | 5.05E-01 | 1.39E-01 | 1.00E+00 | 1.00E+00 | 5.92E-01 | 1.00E+00 | 1.84E-01 | 1.00E+00 |
| 726 | 23 | 2 | *Helicobacter pylori kx1 mgep Yersinia enterocolitica p60 bl6 Listeria monocytogenes Pseudomonas aeruginosa Fusobacterium nucleatum streptococcus pneumoniae tigr4 Ehrlichia chaffeensis arkansa streptococcus gordonii Ehrlichia chaffeensis wakulla Mycobacterium tuberculosis Helicobacter pylori kx2 mgep Yersinia enterocolitica wap bl6 Yersinia enterocolitica wap bc Burkholderia pseudomallei Yersinia enterocolitica p60 bc Lactobacillus acidophilus streptococcus pneumoniae d39 Helicobacter pylori kx1 npgec Bacillus anthracis streptococcus pneumoniae g54 Escherichia coli Helicobacter pylori Shigella dysenteriae* | 2.31E-001 |  | 1.00E+00 | 2.68E-01 | 8.93E-01 | 3.71E-01 | 9.08E-01 | 7.90E-01 | 5.73E-01 | 9.18E-02 | 6.85E-02 | 8.59E-01 | 7.66E-01 | 8.60E-01 |
| 728 | 5 | 9 | *Lactobacillus acidophilus streptococcus pyogenes Mycobacterium tuberculosis Fusobacterium nucleatum Escherichia coli* | 9.98E-001 | 1 | 6.00E-01 | 5.00E-01 | 8.28E-01 | 3.93E-01 | 9.82E-01 | 9.23E-01 | 3.23E-01 | 4.46E-01 | 9.05E-01 | 8.00E-01 | 4.10E-01 | 1.00E+00 |
| 730 | 5 | 9 | *Ehrlichia chaffeensis arkansa Listeria monocytogenes Mycobacterium tuberculosis Fusobacterium nucleatum Aeromonas cavia* | 9.98E-001 |  | 1.00E+00 | 5.00E-01 | 8.28E-01 | 8.00E-01 | 8.69E-01 | 9.23E-01 | 3.23E-01 | 8.32E-01 | 5.96E-01 | 8.00E-01 | 4.10E-01 | 7.21E-01 |
| 732 | 4 | 11 | *streptococcus gordonii Eubacterium rectale Fusobacterium nucleatum streptococcus pneumoniae tigr4* | 9.95E-001 |  | 1.00E+00 | 3.03E-01 | 9.47E-01 | 4.87E-02 | 9.96E-01 | 1.46E-01 | 1.00E+00 | 1.00E+00 | 8.43E-01 | 7.19E-01 | 3.91E-02 | 1.00E+00 |
| 734 | 14 | 3 | *Yersinia enterocolitica wap bl6 Yersinia enterocolitica wap bc Helicobacter pylori kx1 mgep Yersinia enterocolitica p60 bl6 Yersinia enterocolitica p60 bc Eubacterium rectale Lactobacillus acidophilus Listeria monocytogenes Bifidobacterium bifidum Pseudomonas aeruginosa Helicobacter pylori kx1 npgec Ehrlichia chaffeensis wakulla Mycobacterium tuberculosis Escherichia coli* | 9.19E-001 |  | 1.00E+00 | 9.90E-01 | 5.01E-02 | 5.41E-01 | 8.61E-01 | 9.97E-01 | 3.17E-02 | 1.39E-01 | 8.72E-03 | 9.52E-01 | 1.00E+00 | 9.85E-01 |
| 736 | 14 | 3 | *Yersinia enterocolitica wap bc Helicobacter pylori kx2 npgec Helicobacter pylori kx1 mgep Pseudomonas aeruginosa Aeromonas cavia streptococcus pneumoniae tigr4 streptococcus gordonii Ehrlichia chaffeensis wakulla Pseudomonas aeruginosa fdr440 streptococcus pneumoniae g54 Mycobacterium tuberculosis Helicobacter pylori kx2 mgep Shigella dysenteriae Escherichia coli* | 9.19E-001 |  | 1.00E+00 | 3.72E-01 | 8.37E-01 | 8.05E-01 | 6.47E-01 | 2.15E-01 | 1.00E+00 | 6.35E-01 | 1.53E-01 | 2.64E-01 | 8.17E-01 | 9.85E-01 |
| 738 | 8 | 5 | *Yersinia enterocolitica wap bl6 Burkholderia pseudomallei Yersinia enterocolitica p60 bl6 Yersinia enterocolitica p60 bc Porphyromonas gingivalis Fusobacterium nucleatum Brucella melitensis Escherichia coli* | 9.97E-001 |  | 1.00E+00 | 6.52E-01 | 6.52E-01 | 1.00E+00 | 4.96E-02 | 8.98E-01 | 1.00E+00 | 3.61E-03 | 2.94E-01 | 1.00E+00 | 1.58E-01 | 5.57E-01 |
| 740 | 8 | 5 | *Yersinia enterocolitica wap bl6 Yersinia enterocolitica p60 bl6 Yersinia enterocolitica p60 bc streptococcus gordonii Ehrlichia chaffeensis wakulla streptococcus pneumoniae g54 Fusobacterium nucleatum Aeromonas cavia* | 9.97E-001 | 9 | < 3.71E-07 | 8.81E-01 | 3.48E-01 | 6.88E-01 | 5.89E-01 | 6.64E-01 | 1.00E+00 | 4.11E-01 | 2.94E-01 | 9.33E-01 | 1.58E-01 | 8.82E-01 |
| 742 | 5 | 8 | *streptococcus gordonii Pseudomonas aeruginosa fdr440 Listeria monocytogenes Mycobacterium tuberculosis Aggregatibacter actinomycetemcomitans* | 9.99E-001 |  | 1.00E+00 | 2.39E-02 | 1.00E+00 | 3.93E-01 | 9.82E-01 | 2.75E-01 | 3.23E-01 | 8.32E-01 | 9.05E-01 | 3.93E-01 | 6.29E-02 | 1.00E+00 |
| 744 | 5 | 8 | *Yersinia enterocolitica wap bc Yersinia enterocolitica p60 bl6 streptococcus gordonii Listeria monocytogenes Porphyromonas gingivalis* | 9.99E-001 |  | 1.00E+00 | 5.00E-01 | 8.28E-01 | 3.93E-01 | 8.69E-01 | 6.41E-01 | 3.23E-01 | 4.46E-01 | 2.35E-01 | 1.00E+00 | 6.29E-02 | 1.00E+00 |
| 746 | 2 | 19 | *Staphylococcus aureus Helicobacter pylori kx2 mgep* | 9.60E-001 |  | 1.00E+00 | 7.56E-01 | 7.56E-01 | 4.60E-01 | 9.23E-01 | 1.39E-01 | 1.00E+00 | 1.00E+00 | 5.92E-01 | 4.60E-01 | 1.00E+00 | 1.00E+00 |
| 748 | 4 | 9 | *Burkholderia pseudomallei Brucella melitensis Fusobacterium nucleatum Brucella ovis* | 9.98E-001 |  | 1.00E+00 | 6.97E-01 | 6.97E-01 | 1.00E+00 | 2.45E-01 | 8.66E-01 | 1.00E+00 | 6.34E-02 | 1.00E+00 | 1.00E+00 | 3.41E-01 | 2.59E-02 |
| 750 | 4 | 9 | *streptococcus gordonii streptococcus pneumoniae g54 Brucella neotomae Aeromonas cavia* | 9.98E-001 | 3 | 1.09E-02 | 6.97E-01 | 6.97E-01 | 2.77E-01 | 9.37E-01 | 4.95E-01 | 1.00E+00 | 7.55E-01 | 8.43E-01 | 7.19E-01 | 3.41E-01 | 6.34E-01 |
| 752 | 6 | 6 | *Ehrlichia chaffeensis arkansa streptococcus gordonii Ehrlichia chaffeensis wakulla Listeria monocytogenes Helicobacter pylori Ehrlichia chaffeensis liberty* | 9.99E-001 |  | 1.00E+00 | 6.69E-01 | 6.69E-01 | 5.03E-01 | 7.84E-01 | 9.56E-01 | 3.78E-01 | 1.00E+00 | 7.13E-01 | 1.00E+00 | 4.74E-01 | 1.01E-01 |
| 754 | 4 | 9 | *Helicobacter pylori kx2 npgec streptococcus pneumoniae g54 Fusobacterium nucleatum Helicobacter pylori kx1 npgec* | 9.98E-001 | 4 | 4.53E-02 | 6.97E-01 | 6.97E-01 | 7.19E-01 | 6.80E-01 | 1.63E-02 | 1.00E+00 | 1.00E+00 | 4.51E-01 | 7.19E-01 | 3.41E-01 | 1.00E+00 |
| 756 | 4 | 9 | *streptococcus gordonii streptococcus pyogenes Fusobacterium nucleatum Shigella dysenteriae* | 9.98E-001 | 1 | 5.61E-01 | 3.03E-01 | 9.47E-01 | 2.77E-01 | 9.37E-01 | 4.95E-01 | 1.00E+00 | 7.55E-01 | 8.43E-01 | 1.00E+00 | 3.91E-02 | 1.00E+00 |
| 758 | 5 | 7 | *Helicobacter pylori kx1 mgep Brucella melitensis Pseudomonas aeruginosa Fusobacterium nucleatum streptococcus pneumoniae d39* | 1.00E+000 |  | 1.00E+00 | 5.00E-01 | 8.28E-01 | 8.00E-01 | 5.54E-01 | 2.75E-01 | 1.00E+00 | 8.32E-01 | 9.05E-01 | 3.93E-01 | 4.10E-01 | 7.21E-01 |
| 760 | 7 | 5 | *Yersinia enterocolitica wap bc Ehrlichia chaffeensis wakulla Brucella melitensis streptococcus pneumoniae g54 streptococcus pyogenes Fusobacterium nucleatum Shigella dysenteriae* | 9.99E-001 |  | 1.00E+00 | 7.95E-01 | 5.00E-01 | 6.02E-01 | 6.88E-01 | 8.39E-01 | 1.00E+00 | 3.12E-01 | 8.03E-01 | 9.03E-01 | 5.32E-01 | 4.72E-01 |
| 762 | 5 | 7 | *Helicobacter pylori kx2 npgec streptococcus gordonii Lactobacillus acidophilus streptococcus pneumoniae g54 Bifidobacterium bifidum* | 1.00E+000 |  | 1.00E+00 | 8.28E-01 | 5.00E-01 | 1.26E-02 | 9.99E-01 | 2.75E-01 | 3.31E-02 | 1.00E+00 | 9.05E-01 | 8.00E-01 | 4.10E-01 | 1.00E+00 |
| 764 | 7 | 5 | *Pseudomonas aeruginosa fdr440 Mycobacterium tuberculosis Fusobacterium nucleatum Helicobacter pylori kx2 mgep streptococcus pneumoniae tigr4 Escherichia coli Ehrlichia chaffeensis liberty* | 9.99E-001 |  | 1.00E+00 | 2.05E-01 | 9.53E-01 | 9.03E-01 | 6.88E-01 | 2.36E-01 | 1.00E+00 | 6.60E-01 | 8.03E-01 | 2.56E-01 | 5.32E-01 | 8.42E-01 |
| 766 | 11 | 3 | *Yersinia enterocolitica wap bl6 Yersinia enterocolitica p60 bl6 Yersinia enterocolitica p60 bc Fusobacterium nucleatum Brucella ovis Ehrlichia chaffeensis wakulla streptococcus gordonii Pseudomonas aeruginosa fdr1 Brucella melitensis streptococcus pneumoniae g54 Brucella neotomae* | 9.86E-001 | 1 | 1.67E-01 | 9.21E-01 | 2.42E-01 | 8.66E-01 | 3.18E-01 | 6.86E-01 | 1.00E+00 | 3.63E-02 | 8.52E-01 | 8.66E-01 | 2.77E-01 | 1.63E-01 |
| 768 | 3 | 11 | *Yersinia enterocolitica wap bc streptococcus gordonii Helicobacter pylori kx1 npgec* | 9.93E-001 |  | 1.00E+00 | 8.84E-01 | 5.00E-01 | 6.08E-01 | 8.08E-01 | 3.21E-01 | 1.00E+00 | 6.46E-01 | 2.87E-01 | 1.00E+00 | 2.65E-01 | 1.00E+00 |
| 770 | 11 | 3 | *Helicobacter pylori kx1 mgep Lactobacillus acidophilus Pseudomonas aeruginosa Ehrlichia chaffeensis liberty Ehrlichia chaffeensis arkansa streptococcus gordonii Ehrlichia chaffeensis wakulla streptococcus pyogenes Mycobacterium tuberculosis Helicobacter pylori kx2 mgep Escherichia coli* | 9.86E-001 |  | 1.00E+00 | 9.21E-01 | 2.42E-01 | 6.07E-01 | 8.54E-01 | 8.91E-01 | 6.08E-01 | 9.03E-01 | 8.52E-01 | 8.66E-01 | 7.19E-01 | 4.36E-01 |
| 772 | 11 | 3 | *Yersinia enterocolitica wap bc Listeria monocytogenes Pseudomonas aeruginosa Fusobacterium nucleatum Ehrlichia chaffeensis liberty Ehrlichia chaffeensis arkansa streptococcus gordonii Ehrlichia chaffeensis wakulla streptococcus pyogenes Mycobacterium tuberculosis Escherichia coli* | 9.86E-001 |  | 1.00E+00 | 5.00E-01 | 7.58E-01 | 6.07E-01 | 8.54E-01 | 9.78E-01 | 6.08E-01 | 6.82E-01 | 8.52E-01 | 8.66E-01 | 2.77E-01 | 4.36E-01 |
| 774 | 2 | 15 | *Staphylococcus aureus Aeromonas cavia* | 9.76E-001 | 22 | < 3.71E-07 | 7.56E-01 | 7.56E-01 | 4.60E-01 | 9.23E-01 | 6.23E-01 | 1.00E+00 | 1.00E+00 | 5.92E-01 | 4.60E-01 | 1.00E+00 | 1.00E+00 |
| 776 | 3 | 10 | *streptococcus gordonii Bacillus anthracis Ehrlichia chaffeensis liberty* | 9.95E-001 |  | 1.00E+00 | 5.00E-01 | 8.84E-01 | 1.63E-01 | 9.81E-01 | 7.74E-01 | 1.00E+00 | 6.46E-01 | 1.00E+00 | 1.00E+00 | 2.65E-01 | 1.11E-01 |
| 778 | 5 | 6 | *streptococcus gordonii Pseudomonas aeruginosa Brucella ovis streptococcus pneumoniae d39 Escherichia coli* | 1.00E+000 |  | 1.00E+00 | 1.72E-01 | 9.76E-01 | 3.93E-01 | 8.69E-01 | 6.41E-01 | 1.00E+00 | 4.46E-01 | 9.05E-01 | 3.93E-01 | 4.10E-01 | 7.21E-01 |
| 780 | 10 | 3 | *Yersinia enterocolitica wap bl6 Yersinia enterocolitica p60 bl6 Yersinia enterocolitica p60 bc Listeria monocytogenes Fusobacterium nucleatum Aeromonas cavia Ehrlichia chaffeensis arkansa Ehrlichia chaffeensis wakulla Escherichia coli Helicobacter pylori* | 9.93E-001 |  | 1.00E+00 | 8.61E-01 | 3.59E-01 | 9.70E-01 | 1.37E-01 | 9.96E-01 | 5.68E-01 | 2.96E-01 | 1.44E-02 | 1.00E+00 | 6.79E-01 | 7.01E-01 |
| 782 | 3 | 10 | *Yersinia enterocolitica wap bc Eubacterium rectale Bifidobacterium bifidum* | 9.95E-001 |  | 1.00E+00 | 1.00E+00 | 1.16E-01 | 1.63E-01 | 9.81E-01 | 1.00E+00 | 2.04E-01 | 6.46E-01 | 2.87E-01 | 1.00E+00 | 1.00E+00 | 1.00E+00 |
| 784 | 3 | 10 | *Pseudomonas aeruginosa fdr440 Staphylococcus aureus Listeria monocytogenes* | 9.95E-001 |  | 1.00E+00 | 1.16E-01 | 1.00E+00 | 1.63E-01 | 9.81E-01 | 3.21E-01 | 2.04E-01 | 1.00E+00 | 7.45E-01 | 1.63E-01 | 1.00E+00 | 1.00E+00 |
| 786 | 15 | 2 | *Yersinia enterocolitica wap bl6 Yersinia enterocolitica wap bc Yersinia enterocolitica p60 bl6 Yersinia enterocolitica p60 bc Lactobacillus acidophilus Listeria monocytogenes Bifidobacterium bifidum Porphyromonas gingivalis Aeromonas cavia Ehrlichia chaffeensis liberty Ehrlichia chaffeensis arkansa streptococcus gordonii Ehrlichia chaffeensis wakulla streptococcus pyogenes Escherichia coli* | 9.22E-001 |  | 1.00E+00 | 9.96E-01 | 2.58E-02 | 3.33E-01 | 8.07E-01 | 9.98E-01 | 3.96E-02 | 4.34E-01 | 2.20E-01 | 1.00E+00 | 4.51E-01 | 7.06E-01 |
| 788 | 10 | 3 | *Listeria monocytogenes Bifidobacterium bifidum Pseudomonas aeruginosa Porphyromonas gingivalis Fusobacterium nucleatum Ehrlichia chaffeensis liberty Ehrlichia chaffeensis arkansa Ehrlichia chaffeensis wakulla streptococcus pyogenes Mycobacterium tuberculosis* | 9.93E-001 |  | 1.00E+00 | 6.41E-01 | 6.41E-01 | 5.24E-01 | 9.04E-01 | 9.62E-01 | 1.36E-01 | 9.80E-01 | 9.94E-01 | 8.19E-01 | 2.36E-01 | 3.62E-01 |
| 790 | 4 | 7 | *Yersinia enterocolitica wap bl6 Yersinia enterocolitica p60 bl6 Yersinia enterocolitica p60 bc Staphylococcus aureus* | 9.99E-001 | 22 | < 3.71E-07 | 9.47E-01 | 3.03E-01 | 7.19E-01 | 6.80E-01 | 8.66E-01 | 1.00E+00 | 6.34E-02 | 1.22E-01 | 7.19E-01 | 1.00E+00 | 1.00E+00 |
| 792 | 4 | 7 | *Burkholderia pseudomallei Listeria monocytogenes Fusobacterium nucleatum Brucella neotomae* | 9.99E-001 |  | 1.00E+00 | 3.03E-01 | 9.47E-01 | 7.19E-01 | 6.80E-01 | 8.66E-01 | 2.65E-01 | 3.20E-01 | 8.43E-01 | 1.00E+00 | 3.41E-01 | 1.96E-01 |
| 794 | 4 | 7 | *Ehrlichia chaffeensis arkansa Eubacterium rectale Ehrlichia chaffeensis wakulla Listeria monocytogenes* | 9.99E-001 |  | 1.00E+00 | 9.47E-01 | 3.03E-01 | 2.77E-01 | 9.37E-01 | 1.00E+00 | 2.65E-01 | 1.00E+00 | 4.51E-01 | 1.00E+00 | 1.00E+00 | 1.96E-01 |
| 796 | 9 | 3 | *Listeria monocytogenes Fusobacterium nucleatum Brucella ovis Aeromonas cavia Ehrlichia chaffeensis arkansa Brucella melitensis streptococcus pyogenes Brucella neotomae Escherichia coli* | 9.96E-001 |  | 1.00E+00 | 9.35E-01 | 2.27E-01 | 7.60E-01 | 4.91E-01 | 9.93E-01 | 5.25E-01 | 2.16E-01 | 7.06E-01 | 1.00E+00 | 6.34E-01 | 7.97E-02 |
| 798 | 3 | 9 | *Lactobacillus acidophilus Staphylococcus aureus Bifidobacterium bifidum* | 9.97E-001 |  | 1.00E+00 | 8.84E-01 | 5.00E-01 | 1.44E-02 | 1.00E+00 | 7.74E-01 | 1.03E-02 | 1.00E+00 | 1.00E+00 | 6.08E-01 | 1.00E+00 | 1.00E+00 |
| 800 | 9 | 3 | *Lactobacillus acidophilus Bifidobacterium bifidum Fusobacterium nucleatum Aeromonas cavia Aggregatibacter actinomycetemcomitans Ehrlichia chaffeensis wakulla Mycobacterium tuberculosis Brucella neotomae Escherichia coli* | 9.96E-001 |  | 1.00E+00 | 7.73E-01 | 5.00E-01 | 7.60E-01 | 7.84E-01 | 9.37E-01 | 1.11E-01 | 5.09E-01 | 9.15E-01 | 9.55E-01 | 1.96E-01 | 6.33E-01 |
| 802 | 5 | 5 | *streptococcus gordonii Ehrlichia chaffeensis wakulla Brucella melitensis Brucella neotomae Aeromonas cavia* | 1.00E+000 | 3 | 3.76E-02 | 9.76E-01 | 1.72E-01 | 8.00E-01 | 5.54E-01 | 9.23E-01 | 1.00E+00 | 4.46E-01 | 9.05E-01 | 1.00E+00 | 4.10E-01 | 5.72E-02 |
| 804 | 6 | 4 | *streptococcus gordonii Pseudomonas aeruginosa fdr1 streptococcus pneumoniae g54 Listeria monocytogenes Brucella neotomae Escherichia coli* | 1.00E+000 |  | 1.00E+00 | 9.18E-02 | 9.90E-01 | 1.73E-01 | 9.54E-01 | 4.13E-01 | 3.78E-01 | 5.61E-01 | 7.13E-01 | 5.03E-01 | 4.74E-01 | 7.89E-01 |
| 806 | 6 | 4 | *Yersinia enterocolitica p60 bc streptococcus gordonii streptococcus pneumoniae g54 Staphylococcus aureus Fusobacterium nucleatum Brucella neotomae* | 1.00E+000 | 2 | 6.40E-02 | 3.31E-01 | 9.08E-01 | 1.73E-01 | 9.54E-01 | 1.36E-01 | 1.00E+00 | 5.61E-01 | 9.44E-01 | 5.03E-01 | 9.10E-02 | 7.89E-01 |
| 808 | 6 | 4 | *Ehrlichia chaffeensis arkansa Ehrlichia chaffeensis wakulla streptococcus pyogenes Mycobacterium tuberculosis Fusobacterium nucleatum Brucella ovis* | 1.00E+000 |  | 1.00E+00 | 9.08E-01 | 3.31E-01 | 8.60E-01 | 7.84E-01 | 9.56E-01 | 1.00E+00 | 5.61E-01 | 1.00E+00 | 8.60E-01 | 4.74E-01 | 1.01E-01 |
| 810 | 11 | 2 | *Lactobacillus acidophilus Staphylococcus aureus Bifidobacterium bifidum Aeromonas cavia Ehrlichia chaffeensis liberty Ehrlichia chaffeensis arkansa Ehrlichia chaffeensis wakulla streptococcus pyogenes Helicobacter pylori kx2 mgep Escherichia coli Helicobacter pylori* | 9.90E-001 |  | 1.00E+00 | 9.84E-01 | 7.95E-02 | 3.03E-01 | 8.54E-01 | 9.78E-01 | 1.63E-01 | 9.87E-01 | 6.16E-01 | 9.80E-01 | 1.00E+00 | 4.36E-01 |
| 812 | 7 | 3 | *Ehrlichia chaffeensis wakulla Lactobacillus acidophilus Bifidobacterium bifidum Fusobacterium nucleatum Helicobacter pylori kx2 mgep Helicobacter pylori Escherichia coli* | 9.99E-001 |  | 1.00E+00 | 7.95E-01 | 5.00E-01 | 6.02E-01 | 6.88E-01 | 8.39E-01 | 6.71E-02 | 9.25E-01 | 4.88E-01 | 1.00E+00 | 5.32E-01 | 8.42E-01 |
| 814 | 7 | 3 | *Ehrlichia chaffeensis arkansa Listeria monocytogenes streptococcus pyogenes Mycobacterium tuberculosis Fusobacterium nucleatum Brucella neotomae Helicobacter pylori kx1 npgec* | 9.99E-001 |  | 1.00E+00 | 7.95E-01 | 5.00E-01 | 6.02E-01 | 9.12E-01 | 8.39E-01 | 4.30E-01 | 6.60E-01 | 8.03E-01 | 9.03E-01 | 5.32E-01 | 4.72E-01 |
| 816 | 2 | 10 | *Staphylococcus aureus Pseudomonas aeruginosa* | 9.94E-001 |  | 1.00E+00 | 2.44E-01 | 1.00E+00 | 4.60E-01 | 9.23E-01 | 6.23E-01 | 1.00E+00 | 1.00E+00 | 1.00E+00 | 6.39E-02 | 1.00E+00 | 1.00E+00 |
| 818 | 4 | 5 | *Ehrlichia chaffeensis wakulla Fusobacterium nucleatum Brucella ovis Aeromonas cavia* | 1.00E+000 | 7 | 1.00E-05 | 9.47E-01 | 3.03E-01 | 1.00E+00 | 2.45E-01 | 8.66E-01 | 1.00E+00 | 7.55E-01 | 8.43E-01 | 1.00E+00 | 3.41E-01 | 1.96E-01 |
| 820 | 10 | 2 | *Yersinia enterocolitica wap bl6 Yersinia enterocolitica wap bc Yersinia enterocolitica p60 bl6 Yersinia enterocolitica p60 bc Eubacterium rectale Lactobacillus acidophilus Listeria monocytogenes Bifidobacterium bifidum Porphyromonas gingivalis Helicobacter pylori kx1 npgec* | 9.94E-001 |  | 1.00E+00 | 9.96E-01 | 3.35E-02 | 2.29E-01 | 9.04E-01 | 9.62E-01 | 1.05E-02 | 2.96E-01 | 1.44E-02 | 1.00E+00 | 6.79E-01 | 1.00E+00 |
| 822 | 4 | 5 | *Brucella melitensis streptococcus pneumoniae g54 Staphylococcus aureus Brucella neotomae* | 1.00E+000 | 3 | 6.48E-02 | 6.97E-01 | 6.97E-01 | 2.77E-01 | 9.37E-01 | 4.95E-01 | 1.00E+00 | 3.20E-01 | 1.00E+00 | 2.77E-01 | 1.00E+00 | 1.96E-01 |
| 824 | 10 | 2 | *Listeria monocytogenes Fusobacterium nucleatum Aeromonas cavia Ehrlichia chaffeensis liberty Ehrlichia chaffeensis arkansa Ehrlichia chaffeensis wakulla streptococcus pneumoniae g54 streptococcus pyogenes Mycobacterium tuberculosis Escherichia coli* | 9.94E-001 |  | 1.00E+00 | 6.41E-01 | 6.41E-01 | 5.24E-01 | 9.04E-01 | 9.62E-01 | 5.68E-01 | 8.63E-01 | 7.88E-01 | 8.19E-01 | 6.79E-01 | 3.62E-01 |
| 826 | 10 | 2 | *Lactobacillus acidophilus Listeria monocytogenes Pseudomonas aeruginosa Fusobacterium nucleatum Aeromonas cavia Ehrlichia chaffeensis arkansa streptococcus gordonii Ehrlichia chaffeensis wakulla Mycobacterium tuberculosis Helicobacter pylori* | 9.94E-001 |  | 1.00E+00 | 3.59E-01 | 8.61E-01 | 5.24E-01 | 9.04E-01 | 9.62E-01 | 1.36E-01 | 9.80E-01 | 7.88E-01 | 8.19E-01 | 2.36E-01 | 7.01E-01 |
| 828 | 2 | 9 | *Eubacterium rectale Brucella neotomae* | 9.96E-001 |  | 1.00E+00 | 1.00E+00 | 2.44E-01 | 4.60E-01 | 9.23E-01 | 1.00E+00 | 1.00E+00 | 4.95E-01 | 5.92E-01 | 1.00E+00 | 1.00E+00 | 3.87E-01 |
| 830 | 3 | 6 | *streptococcus gordonii Pseudomonas aeruginosa fdr1 Pseudomonas aeruginosa* | 1.00E+000 |  | 1.00E+00 | 1.16E-01 | 1.00E+00 | 6.08E-01 | 8.08E-01 | 3.21E-01 | 1.00E+00 | 1.00E+00 | 1.00E+00 | 1.63E-01 | 2.65E-01 | 1.00E+00 |
| 832 | 6 | 3 | *Ehrlichia chaffeensis arkansa streptococcus gordonii Ehrlichia chaffeensis wakulla streptococcus pneumoniae g54 Staphylococcus aureus Aeromonas cavia* | 1.00E+000 | 7 | 4.00E-05 | 6.69E-01 | 6.69E-01 | 1.73E-01 | 9.54E-01 | 4.13E-01 | 1.00E+00 | 1.00E+00 | 9.44E-01 | 5.03E-01 | 4.74E-01 | 3.82E-01 |
| 834 | 6 | 3 | *Helicobacter pylori kx2 npgec streptococcus gordonii Lactobacillus acidophilus streptococcus pneumoniae g54 Aeromonas cavia Helicobacter pylori kx1 npgec* | 1.00E+000 |  | 1.00E+00 | 9.08E-01 | 3.31E-01 | 1.73E-01 | 9.54E-01 | 1.36E-01 | 3.78E-01 | 1.00E+00 | 3.61E-01 | 8.60E-01 | 4.74E-01 | 1.00E+00 |
| 836 | 2 | 8 | *Burkholderia pseudomallei Staphylococcus aureus* | 9.98E-001 |  | 1.00E+00 | 2.44E-01 | 1.00E+00 | 4.60E-01 | 9.23E-01 | 6.23E-01 | 1.00E+00 | 4.95E-01 | 1.00E+00 | 4.60E-01 | 1.00E+00 | 3.87E-01 |
| 838 | 4 | 4 | *Eubacterium rectale Ehrlichia chaffeensis wakulla Listeria monocytogenes Helicobacter pylori kx2 mgep* | 1.00E+000 |  | 1.00E+00 | 9.47E-01 | 3.03E-01 | 2.77E-01 | 9.37E-01 | 8.66E-01 | 2.65E-01 | 1.00E+00 | 1.22E-01 | 1.00E+00 | 1.00E+00 | 6.34E-01 |
| 840 | 5 | 3 | *streptococcus gordonii Pseudomonas aeruginosa fdr1 Brucella melitensis Brucella ovis Escherichia coli* | 1.00E+000 |  | 1.00E+00 | 5.00E-01 | 8.28E-01 | 8.00E-01 | 5.54E-01 | 6.41E-01 | 1.00E+00 | 1.31E-01 | 9.05E-01 | 8.00E-01 | 4.10E-01 | 2.88E-01 |
| 842 | 7 | 2 | *streptococcus gordonii Eubacterium rectale Brucella melitensis Fusobacterium nucleatum Brucella ovis Brucella neotomae streptococcus pneumoniae tigr4* | 9.99E-001 |  | 1.00E+00 | 7.95E-01 | 5.00E-01 | 2.56E-01 | 9.12E-01 | 5.46E-01 | 1.00E+00 | 3.12E-01 | 9.67E-01 | 9.03E-01 | 1.23E-01 | 1.55E-01 |
| 844 | 7 | 2 | *Burkholderia pseudomallei Lactobacillus acidophilus Brucella melitensis Staphylococcus aureus Bifidobacterium bifidum Brucella ovis Aeromonas cavia* | 9.99E-001 |  | 1.00E+00 | 9.53E-01 | 2.05E-01 | 2.56E-01 | 9.12E-01 | 9.76E-01 | 6.71E-02 | 3.12E-01 | 9.67E-01 | 9.03E-01 | 1.00E+00 | 1.55E-01 |
| 846 | 7 | 2 | *Burkholderia pseudomallei Lactobacillus acidophilus streptococcus pneumoniae g54 Staphylococcus aureus Bifidobacterium bifidum Pseudomonas aeruginosa Brucella neotomae* | 9.99E-001 |  | 1.00E+00 | 5.00E-01 | 7.95E-01 | 6.35E-02 | 9.86E-01 | 8.39E-01 | 6.71E-02 | 6.60E-01 | 1.00E+00 | 2.56E-01 | 1.00E+00 | 4.72E-01 |
| 848 | 4 | 3 | *Ehrlichia chaffeensis wakulla Staphylococcus aureus Listeria monocytogenes Aeromonas cavia* | 1.00E+000 |  | 1.00E+00 | 6.97E-01 | 6.97E-01 | 2.77E-01 | 9.37E-01 | 8.66E-01 | 2.65E-01 | 1.00E+00 | 4.51E-01 | 7.19E-01 | 1.00E+00 | 6.34E-01 |
| 850 | 3 | 4 | *streptococcus pneumoniae g54 Fusobacterium nucleatum Helicobacter pylori* | 1.00E+000 |  | 1.00E+00 | 1.16E-01 | 1.00E+00 | 6.08E-01 | 8.08E-01 | 3.21E-01 | 1.00E+00 | 1.00E+00 | 7.45E-01 | 6.08E-01 | 2.65E-01 | 1.00E+00 |
| 852 | 3 | 4 | *Eubacterium rectale Ehrlichia chaffeensis wakulla Brucella melitensis* | 1.00E+000 |  | 1.00E+00 | 1.00E+00 | 1.16E-01 | 6.08E-01 | 8.08E-01 | 1.00E+00 | 1.00E+00 | 6.46E-01 | 7.45E-01 | 1.00E+00 | 1.00E+00 | 1.11E-01 |
| 854 | 4 | 3 | *Yersinia enterocolitica p60 bl6 Yersinia enterocolitica p60 bc Brucella melitensis Staphylococcus aureus* | 1.00E+000 | 8 | 4.00E-05 | 9.47E-01 | 3.03E-01 | 7.19E-01 | 6.80E-01 | 8.66E-01 | 1.00E+00 | 6.34E-02 | 4.51E-01 | 7.19E-01 | 1.00E+00 | 6.34E-01 |
| 856 | 6 | 2 | *streptococcus gordonii Lactobacillus acidophilus streptococcus pneumoniae g54 Staphylococcus aureus Brucella neotomae Aeromonas cavia* | 1.00E+000 |  | 1.00E+00 | 6.69E-01 | 6.69E-01 | 3.21E-02 | 9.95E-01 | 4.13E-01 | 3.78E-01 | 8.87E-01 | 9.44E-01 | 5.03E-01 | 4.74E-01 | 7.89E-01 |
| 858 | 4 | 3 | *Burkholderia pseudomallei Eubacterium rectale Listeria monocytogenes Mycobacterium tuberculosis* | 1.00E+000 |  | 1.00E+00 | 3.03E-01 | 9.47E-01 | 2.77E-01 | 9.96E-01 | 1.00E+00 | 2.65E-01 | 3.20E-01 | 4.51E-01 | 7.19E-01 | 1.00E+00 | 6.34E-01 |
| 860 | 2 | 5 | *Staphylococcus aureus Porphyromonas gingivalis* | 1.00E+000 |  | 1.00E+00 | 2.44E-01 | 1.00E+00 | 4.60E-01 | 9.23E-01 | 1.39E-01 | 1.00E+00 | 1.00E+00 | 1.00E+00 | 4.60E-01 | 1.84E-01 | 1.00E+00 |
| 862 | 5 | 2 | *Brucella melitensis Staphylococcus aureus Pseudomonas aeruginosa Brucella ovis Escherichia coli* | 1.00E+000 |  | 1.00E+00 | 5.00E-01 | 8.28E-01 | 8.00E-01 | 5.54E-01 | 9.23E-01 | 1.00E+00 | 1.31E-01 | 9.05E-01 | 3.93E-01 | 1.00E+00 | 2.88E-01 |
| 864 | 3 | 3 | *Eubacterium rectale Ehrlichia chaffeensis wakulla streptococcus pneumoniae g54* | 1.00E+000 |  | 1.00E+00 | 8.84E-01 | 5.00E-01 | 1.63E-01 | 9.81E-01 | 7.74E-01 | 1.00E+00 | 1.00E+00 | 7.45E-01 | 6.08E-01 | 1.00E+00 | 5.25E-01 |
| 866 | 3 | 3 | *Yersinia enterocolitica wap bl6 Yersinia enterocolitica wap bc Staphylococcus aureus* | 1.00E+000 |  | 1.00E+00 | 8.84E-01 | 5.00E-01 | 6.08E-01 | 8.08E-01 | 7.74E-01 | 1.00E+00 | 1.92E-01 | 2.87E-01 | 6.08E-01 | 1.00E+00 | 1.00E+00 |
| 868 | 4 | 2 | *streptococcus pneumoniae g54 Staphylococcus aureus Listeria monocytogenes Aeromonas cavia* | 1.00E+000 |  | 1.00E+00 | 3.03E-01 | 9.47E-01 | 4.87E-02 | 9.96E-01 | 4.95E-01 | 2.65E-01 | 1.00E+00 | 4.51E-01 | 2.77E-01 | 1.00E+00 | 1.00E+00 |
| 870 | 2 | 3 | *Staphylococcus aureus Aggregatibacter actinomycetemcomitans* | 1.00E+000 |  | 1.00E+00 | 2.44E-01 | 1.00E+00 | 4.60E-01 | 9.23E-01 | 1.39E-01 | 1.00E+00 | 1.00E+00 | 1.00E+00 | 4.60E-01 | 1.84E-01 | 1.00E+00 |
| 872 | 2 | 3 | *Helicobacter pylori kx2 npgec Staphylococcus aureus* | 1.00E+000 |  | 1.00E+00 | 7.56E-01 | 7.56E-01 | 4.60E-01 | 9.23E-01 | 1.39E-01 | 1.00E+00 | 1.00E+00 | 5.92E-01 | 4.60E-01 | 1.00E+00 | 1.00E+00 |
| 874 | 3 | 2 | *streptococcus gordonii Staphylococcus aureus Pseudomonas aeruginosa* | 1.00E+000 |  | 1.00E+00 | 1.16E-01 | 1.00E+00 | 1.63E-01 | 9.81E-01 | 3.21E-01 | 1.00E+00 | 1.00E+00 | 1.00E+00 | 1.63E-01 | 2.65E-01 | 1.00E+00 |
| 876 | 2 | 3 | *Staphylococcus aureus streptococcus pneumoniae d39* | 1.00E+000 |  | 1.00E+00 | 2.44E-01 | 1.00E+00 | 6.39E-02 | 1.00E+00 | 1.39E-01 | 1.00E+00 | 1.00E+00 | 1.00E+00 | 6.39E-02 | 1.00E+00 | 1.00E+00 |
| 878 | 2 | 3 | *Helicobacter pylori kx1 mgep Staphylococcus aureus* | 1.00E+000 |  | 1.00E+00 | 7.56E-01 | 7.56E-01 | 4.60E-01 | 9.23E-01 | 1.39E-01 | 1.00E+00 | 1.00E+00 | 5.92E-01 | 4.60E-01 | 1.00E+00 | 1.00E+00 |
