# Supplementary material for: The Landscape of Host Transcriptional Response Programs Commonly Perturbed by Bacterial Pathogens: Towards Host-Oriented Broad-Spectrum Drug Targets
Source: PLoS One. 2013 Mar 13;8(3):e58553. doi: 10.1371/journal.pone.0058553 (PMC3596304; doi:10.1371/journal.pone.0058553)
Supplement: Table S3 — Down-regulated biclusters. It contains detail information on all down-regulated biclusters. This include: bicluster ID, list of pathogens and gene sets in bicluster, -values indicating statistical significance of bicluster and enrichment of these biclusters in various attributes such as drug targets and host type. (HTML) [file pone.0058553.s004.html]

**Summary of Down-regulated Biclusters and their Enrichment in Various Attributes** 

*Click on the "Bicluster ID" to view details. Statistically significant biclusters are highlighted in green*

|  |  |  |  |  |  |  |  |  |  |  |  |  |  |  |  |  |  |
| --- | --- | --- | --- | --- | --- | --- | --- | --- | --- | --- | --- | --- | --- | --- | --- | --- | --- |
| **Bicluster ID** | **# Pathogens** | **# Genesets** | **List of Pathogens** | **Bicluster p-value** | **# Targets** | **Target Enrich. (p-value)** | ***Mus musclus Enrich. (p-value)*** | ***Homo sapiens Enrich. (p-value)*** | **Grm. Pos. Enrich. (p-value)** | **Grm. Neg. Enrich. (p-value)** | **Epithelial Enrich. (p-value)** | **Dendritic Enrich. (p-value)** | **Macrophage Enrch. (p-value)** | **GI Enrich. (p-value)** | **Resp. Enrich. (p-value)** | **Oral Cavity Enrich. (p-value)** | **Hemato. Enrich. (p-value)** |
| 0 | 2 | 1443 | *Helicobacter pylori kx2 npgec Helicobacter pylori kx1 npgec* | < 3.56E-004 | 386 | < 1.67E-026 | 1.00E+000 | 2.44E-001 | 1.00E+000 | 5.05E-001 | 1.39E-001 | 1.00E+000 | 1.00E+000 | 1.22E-001 | 1.00E+000 | 1.00E+000 | 1.00E+000 |
| 2 | 2 | 743 | *Lactobacillus acidophilus Bifidobacterium bifidum* | < 3.56E-004 | 225 | 1.03E-011 | 1.00E+000 | 2.44E-001 | 6.39E-002 | 1.00E+000 | 1.00E+000 | 3.48E-003 | 1.00E+000 | 1.00E+000 | 1.00E+000 | 1.00E+000 | 1.00E+000 |
| 4 | 4 | 343 | *Helicobacter pylori kx2 npgec Burkholderia pseudomallei Bifidobacterium bifidum Helicobacter pylori kx1 npgec* | < 3.56E-004 | 50 | 5.16E-013 | 9.47E-001 | 3.03E-001 | 7.19E-001 | 6.80E-001 | 4.95E-001 | 2.65E-001 | 7.55E-001 | 4.51E-001 | 1.00E+000 | 1.00E+000 | 6.34E-001 |
| 358 | 2 | 71 | *Ehrlichia chaffeensis wakulla Porphyromonas gingivalis* | 1.00E+000 | 130 | < 1.67E-026 | 7.56E-001 | 7.56E-001 | 1.00E+000 | 5.05E-001 | 6.23E-001 | 1.00E+000 | 1.00E+000 | 1.00E+000 | 1.00E+000 | 1.84E-001 | 3.87E-001 |
| 898 | 2 | 28 | *Burkholderia pseudomallei Pseudomonas aeruginosa fdr875* | 1.00E+000 | 7 | 4.00E-001 | 2.44E-001 | 1.00E+000 | 1.00E+000 | 5.05E-001 | 6.23E-001 | 1.00E+000 | 4.95E-001 | 1.00E+000 | 4.60E-001 | 1.00E+000 | 3.87E-001 |
| 548 | 2 | 48 | *streptococcus gordonii Porphyromonas gingivalis* | 1.00E+000 | 37 | 8.87E-001 | 2.44E-001 | 1.00E+000 | 4.60E-001 | 9.23E-001 | 1.39E-001 | 1.00E+000 | 1.00E+000 | 1.00E+000 | 1.00E+000 | 6.97E-003 | 1.00E+000 |
| 1954 | 2 | 3 | *Helicobacter pylori kx2 npgec Helicobacter pylori* | 1.00E+000 | 0 | 1.00E+000 | 7.56E-001 | 7.56E-001 | 1.00E+000 | 5.05E-001 | 6.23E-001 | 1.00E+000 | 1.00E+000 | 1.22E-001 | 1.00E+000 | 1.00E+000 | 1.00E+000 |
| 218 | 2 | 104 | *Pseudomonas aeruginosa fdr1 Porphyromonas gingivalis* | 1.00E+000 | 123 | < 1.67E-026 | 2.44E-001 | 1.00E+000 | 1.00E+000 | 5.05E-001 | 1.39E-001 | 1.00E+000 | 1.00E+000 | 1.00E+000 | 4.60E-001 | 1.84E-001 | 1.00E+000 |
| 772 | 2 | 33 | *streptococcus pyogenes Aeromonas cavia* | 1.00E+000 | 95 | < 1.67E-026 | 1.00E+000 | 2.44E-001 | 4.60E-001 | 9.23E-001 | 1.00E+000 | 1.00E+000 | 1.00E+000 | 5.92E-001 | 1.00E+000 | 1.00E+000 | 1.00E+000 |
| 1876 | 2 | 5 | *Yersinia enterocolitica p60 bc Eubacterium rectale* | 1.00E+000 | 3 | 8.63E-001 | 1.00E+000 | 2.44E-001 | 4.60E-001 | 9.23E-001 | 1.00E+000 | 1.00E+000 | 4.95E-001 | 1.22E-001 | 1.00E+000 | 1.00E+000 | 1.00E+000 |
| 8 | 2 | 462 | *Aeromonas cavia Helicobacter pylori kx1 npgec* | 1.00E+000 | 216 | 1.88E-011 | 1.00E+000 | 2.44E-001 | 1.00E+000 | 5.05E-001 | 6.23E-001 | 1.00E+000 | 1.00E+000 | 1.22E-001 | 1.00E+000 | 1.00E+000 | 1.00E+000 |
| 98 | 2 | 168 | *Aeromonas cavia streptococcus pneumoniae d39* | 1.00E+000 | 137 | 3.44E-007 | 7.56E-001 | 7.56E-001 | 4.60E-001 | 9.23E-001 | 6.23E-001 | 1.00E+000 | 1.00E+000 | 5.92E-001 | 4.60E-001 | 1.00E+000 | 1.00E+000 |
| 100 | 2 | 163 | *Pseudomonas aeruginosa fdr440 Mycobacterium tuberculosis* | 1.00E+000 | 60 | 1.14E-003 | 2.44E-001 | 1.00E+000 | 1.00E+000 | 9.23E-001 | 6.23E-001 | 1.00E+000 | 4.95E-001 | 1.00E+000 | 6.39E-002 | 1.00E+000 | 1.00E+000 |
| 94 | 2 | 172 | *Bifidobacterium bifidum Helicobacter pylori kx2 mgep* | 1.00E+000 | 67 | 1.45E-012 | 1.00E+000 | 2.44E-001 | 4.60E-001 | 9.23E-001 | 6.23E-001 | 1.39E-001 | 1.00E+000 | 5.92E-001 | 1.00E+000 | 1.00E+000 | 1.00E+000 |
| 1622 | 2 | 10 | *Ehrlichia chaffeensis arkansa Listeria monocytogenes* | 1.00E+000 | 31 | 4.62E-012 | 7.56E-001 | 7.56E-001 | 4.60E-001 | 9.23E-001 | 1.00E+000 | 1.39E-001 | 1.00E+000 | 5.92E-001 | 1.00E+000 | 1.00E+000 | 3.87E-001 |
| 20 | 2 | 352 | *Helicobacter pylori kx1 npgec Aggregatibacter actinomycetemcomitans* | 1.00E+000 | 130 | 6.48E-009 | 7.56E-001 | 7.56E-001 | 1.00E+000 | 5.05E-001 | 1.39E-001 | 1.00E+000 | 1.00E+000 | 5.92E-001 | 1.00E+000 | 1.84E-001 | 1.00E+000 |
| 700 | 2 | 38 | *Ehrlichia chaffeensis wakulla Mycobacterium tuberculosis* | 1.00E+000 | 44 | 4.88E-015 | 7.56E-001 | 7.56E-001 | 1.00E+000 | 9.23E-001 | 1.00E+000 | 1.00E+000 | 4.95E-001 | 1.00E+000 | 4.60E-001 | 1.00E+000 | 3.87E-001 |
| 1140 | 2 | 20 | *Pseudomonas aeruginosa fdr1 Pseudomonas aeruginosa fdr875* | 1.00E+000 | 13 | 4.85E-001 | 2.44E-001 | 1.00E+000 | 1.00E+000 | 5.05E-001 | 1.39E-001 | 1.00E+000 | 1.00E+000 | 1.00E+000 | 6.39E-002 | 1.00E+000 | 1.00E+000 |
| 1414 | 2 | 14 | *Pseudomonas aeruginosa fdr875 Aeromonas cavia* | 1.00E+000 | 7 | 4.43E-002 | 7.56E-001 | 7.56E-001 | 1.00E+000 | 5.05E-001 | 6.23E-001 | 1.00E+000 | 1.00E+000 | 5.92E-001 | 4.60E-001 | 1.00E+000 | 1.00E+000 |
| 110 | 2 | 157 | *Ehrlichia chaffeensis arkansa Ehrlichia chaffeensis wakulla* | 1.00E+000 | 345 | 2.89E-011 | 1.00E+000 | 2.44E-001 | 1.00E+000 | 5.05E-001 | 1.00E+000 | 1.00E+000 | 1.00E+000 | 1.00E+000 | 1.00E+000 | 1.00E+000 | 4.18E-002 |
| 1944 | 2 | 3 | *streptococcus pneumoniae tigr4 streptococcus pneumoniae d39* | 1.00E+000 | 2 | 8.23E-001 | 2.44E-001 | 1.00E+000 | 6.39E-002 | 1.00E+000 | 1.39E-001 | 1.00E+000 | 1.00E+000 | 1.00E+000 | 6.39E-002 | 1.00E+000 | 1.00E+000 |
| 264 | 2 | 87 | *Pseudomonas aeruginosa fdr440 Helicobacter pylori kx2 mgep* | 1.00E+000 | 47 | 7.07E-006 | 7.56E-001 | 7.56E-001 | 1.00E+000 | 5.05E-001 | 1.39E-001 | 1.00E+000 | 1.00E+000 | 5.92E-001 | 4.60E-001 | 1.00E+000 | 1.00E+000 |
| 48 | 2 | 252 | *Burkholderia pseudomallei Pseudomonas aeruginosa* | 1.00E+000 | 96 | 3.59E-006 | 2.44E-001 | 1.00E+000 | 1.00E+000 | 5.05E-001 | 1.00E+000 | 1.00E+000 | 4.95E-001 | 1.00E+000 | 4.60E-001 | 1.00E+000 | 3.87E-001 |
| 1574 | 2 | 11 | *Brucella neotomae Helicobacter pylori kx1 npgec* | 1.00E+000 | 4 | 1.63E-002 | 1.00E+000 | 2.44E-001 | 1.00E+000 | 5.05E-001 | 6.23E-001 | 1.00E+000 | 4.95E-001 | 5.92E-001 | 1.00E+000 | 1.00E+000 | 3.87E-001 |
| 128 | 2 | 148 | *Helicobacter pylori kx2 npgec Yersinia enterocolitica p60 bl6* | 1.00E+000 | 148 | < 1.67E-026 | 1.00E+000 | 2.44E-001 | 1.00E+000 | 5.05E-001 | 6.23E-001 | 1.00E+000 | 4.95E-001 | 1.22E-001 | 1.00E+000 | 1.00E+000 | 1.00E+000 |
| 28 | 2 | 321 | *Burkholderia pseudomallei Staphylococcus aureus* | 1.00E+000 | 228 | 2.84E-011 | 2.44E-001 | 1.00E+000 | 4.60E-001 | 9.23E-001 | 6.23E-001 | 1.00E+000 | 4.95E-001 | 1.00E+000 | 4.60E-001 | 1.00E+000 | 3.87E-001 |
| 40 | 2 | 267 | *Yersinia enterocolitica wap bl6 Helicobacter pylori kx1 npgec* | 1.00E+000 | 177 | 2.21E-011 | 1.00E+000 | 2.44E-001 | 1.00E+000 | 5.05E-001 | 6.23E-001 | 1.00E+000 | 4.95E-001 | 1.22E-001 | 1.00E+000 | 1.00E+000 | 1.00E+000 |
| 1410 | 2 | 14 | *Pseudomonas aeruginosa fdr1234 Pseudomonas aeruginosa fdr875* | 1.00E+000 | 22 | 1.12E-002 | 2.44E-001 | 1.00E+000 | 1.00E+000 | 5.05E-001 | 1.39E-001 | 1.00E+000 | 1.00E+000 | 1.00E+000 | 6.39E-002 | 1.00E+000 | 1.00E+000 |
| 1218 | 2 | 18 | *Anaplasma phagocytophilum Mycobacterium tuberculosis* | 1.00E+000 | 20 | 1.56E-007 | 2.44E-001 | 1.00E+000 | 1.00E+000 | 9.23E-001 | 1.00E+000 | 1.00E+000 | 4.95E-001 | 1.00E+000 | 4.60E-001 | 1.00E+000 | 3.87E-001 |
| 1806 | 2 | 7 | *Ehrlichia chaffeensis wakulla Fusobacterium nucleatum* | 1.00E+000 | 28 | 3.33E-004 | 7.56E-001 | 7.56E-001 | 1.00E+000 | 5.05E-001 | 6.23E-001 | 1.00E+000 | 1.00E+000 | 1.00E+000 | 1.00E+000 | 1.84E-001 | 3.87E-001 |
| 1958 | 2 | 2 | *Pseudomonas aeruginosa fdr1234 streptococcus pneumoniae tigr4* | 1.00E+000 | 2 | 4.76E-001 | 2.44E-001 | 1.00E+000 | 4.60E-001 | 9.23E-001 | 1.39E-001 | 1.00E+000 | 1.00E+000 | 1.00E+000 | 6.39E-002 | 1.00E+000 | 1.00E+000 |
| 1962 | 2 | 2 | *Brucella melitensis Helicobacter pylori kx1 npgec* | 1.00E+000 | 0 | 1.00E+000 | 1.00E+000 | 2.44E-001 | 1.00E+000 | 5.05E-001 | 6.23E-001 | 1.00E+000 | 4.95E-001 | 5.92E-001 | 1.00E+000 | 1.00E+000 | 3.87E-001 |
| 1748 | 2 | 8 | *Burkholderia pseudomallei Brucella ovis* | 1.00E+000 | 2 | 2.49E-001 | 7.56E-001 | 7.56E-001 | 1.00E+000 | 5.05E-001 | 1.00E+000 | 1.00E+000 | 7.67E-002 | 1.00E+000 | 1.00E+000 | 1.00E+000 | 4.18E-002 |
| 830 | 2 | 31 | *Ehrlichia chaffeensis arkansa Mycobacterium tuberculosis* | 1.00E+000 | 63 | < 1.67E-026 | 7.56E-001 | 7.56E-001 | 1.00E+000 | 9.23E-001 | 1.00E+000 | 1.00E+000 | 4.95E-001 | 1.00E+000 | 4.60E-001 | 1.00E+000 | 3.87E-001 |
| 154 | 2 | 131 | *streptococcus gordonii streptococcus pneumoniae d39* | 1.00E+000 | 98 | 9.35E-001 | 2.44E-001 | 1.00E+000 | 6.39E-002 | 1.00E+000 | 1.39E-001 | 1.00E+000 | 1.00E+000 | 1.00E+000 | 4.60E-001 | 1.84E-001 | 1.00E+000 |
| 1942 | 2 | 3 | *streptococcus gordonii Pseudomonas aeruginosa fdr1234* | 1.00E+000 | 3 | 1.59E-001 | 2.44E-001 | 1.00E+000 | 4.60E-001 | 9.23E-001 | 1.39E-001 | 1.00E+000 | 1.00E+000 | 1.00E+000 | 4.60E-001 | 1.84E-001 | 1.00E+000 |
| 1960 | 2 | 2 | *Pseudomonas aeruginosa fdr1234 Fusobacterium nucleatum* | 1.00E+000 | 1 | 5.61E-001 | 2.44E-001 | 1.00E+000 | 1.00E+000 | 5.05E-001 | 1.39E-001 | 1.00E+000 | 1.00E+000 | 1.00E+000 | 4.60E-001 | 1.84E-001 | 1.00E+000 |
| 334 | 2 | 74 | *streptococcus gordonii Aeromonas cavia* | 1.00E+000 | 60 | 4.22E-005 | 7.56E-001 | 7.56E-001 | 4.60E-001 | 9.23E-001 | 6.23E-001 | 1.00E+000 | 1.00E+000 | 5.92E-001 | 1.00E+000 | 1.84E-001 | 1.00E+000 |
| 770 | 2 | 33 | *Yersinia enterocolitica wap bl6 streptococcus gordonii* | 1.00E+000 | 36 | 7.59E-002 | 7.56E-001 | 7.56E-001 | 4.60E-001 | 9.23E-001 | 6.23E-001 | 1.00E+000 | 4.95E-001 | 5.92E-001 | 1.00E+000 | 1.84E-001 | 1.00E+000 |
| 544 | 2 | 48 | *Fusobacterium nucleatum Aggregatibacter actinomycetemcomitans* | 1.00E+000 | 54 | 4.03E-001 | 2.44E-001 | 1.00E+000 | 1.00E+000 | 5.05E-001 | 1.39E-001 | 1.00E+000 | 1.00E+000 | 1.00E+000 | 1.00E+000 | 6.97E-003 | 1.00E+000 |
| 1196 | 2 | 18 | *Pseudomonas aeruginosa fdr875 Staphylococcus aureus* | 1.00E+000 | 27 | 4.37E-002 | 2.44E-001 | 1.00E+000 | 4.60E-001 | 9.23E-001 | 1.39E-001 | 1.00E+000 | 1.00E+000 | 1.00E+000 | 6.39E-002 | 1.00E+000 | 1.00E+000 |
| 1464 | 2 | 13 | *Burkholderia pseudomallei Eubacterium rectale* | 1.00E+000 | 6 | 7.23E-001 | 7.56E-001 | 7.56E-001 | 4.60E-001 | 9.23E-001 | 1.00E+000 | 1.00E+000 | 4.95E-001 | 5.92E-001 | 1.00E+000 | 1.00E+000 | 3.87E-001 |
| 46 | 2 | 258 | *Mycobacterium tuberculosis Helicobacter pylori kx1 npgec* | 1.00E+000 | 88 | < 1.67E-026 | 7.56E-001 | 7.56E-001 | 1.00E+000 | 9.23E-001 | 6.23E-001 | 1.00E+000 | 4.95E-001 | 5.92E-001 | 4.60E-001 | 1.00E+000 | 1.00E+000 |
| 608 | 2 | 43 | *Pseudomonas aeruginosa fdr1234 Porphyromonas gingivalis* | 1.00E+000 | 50 | 1.08E-005 | 2.44E-001 | 1.00E+000 | 1.00E+000 | 5.05E-001 | 1.39E-001 | 1.00E+000 | 1.00E+000 | 1.00E+000 | 4.60E-001 | 1.84E-001 | 1.00E+000 |
| 190 | 2 | 115 | *Pseudomonas aeruginosa fdr440 Pseudomonas aeruginosa* | 1.00E+000 | 55 | 1.52E-004 | 2.44E-001 | 1.00E+000 | 1.00E+000 | 5.05E-001 | 6.23E-001 | 1.00E+000 | 1.00E+000 | 1.00E+000 | 6.39E-002 | 1.00E+000 | 1.00E+000 |
| 1808 | 2 | 7 | *Eubacterium rectale streptococcus pneumoniae d39* | 1.00E+000 | 12 | 4.48E-002 | 7.56E-001 | 7.56E-001 | 6.39E-002 | 1.00E+000 | 6.23E-001 | 1.00E+000 | 1.00E+000 | 5.92E-001 | 4.60E-001 | 1.00E+000 | 1.00E+000 |
| 148 | 2 | 133 | *Porphyromonas gingivalis Helicobacter pylori kx2 mgep* | 1.00E+000 | 115 | < 1.67E-026 | 7.56E-001 | 7.56E-001 | 1.00E+000 | 5.05E-001 | 1.39E-001 | 1.00E+000 | 1.00E+000 | 5.92E-001 | 1.00E+000 | 1.84E-001 | 1.00E+000 |
| 106 | 2 | 159 | *Burkholderia pseudomallei Yersinia enterocolitica p60 bc* | 1.00E+000 | 112 | 1.52E-013 | 7.56E-001 | 7.56E-001 | 1.00E+000 | 5.05E-001 | 1.00E+000 | 1.00E+000 | 7.67E-002 | 5.92E-001 | 1.00E+000 | 1.00E+000 | 3.87E-001 |
| 86 | 2 | 180 | *streptococcus gordonii Fusobacterium nucleatum* | 1.00E+000 | 132 | 2.37E-001 | 2.44E-001 | 1.00E+000 | 4.60E-001 | 9.23E-001 | 1.39E-001 | 1.00E+000 | 1.00E+000 | 1.00E+000 | 1.00E+000 | 6.97E-003 | 1.00E+000 |
| 186 | 2 | 116 | *Aeromonas cavia Ehrlichia chaffeensis liberty* | 1.00E+000 | 169 | < 1.67E-026 | 1.00E+000 | 2.44E-001 | 1.00E+000 | 5.05E-001 | 1.00E+000 | 1.00E+000 | 1.00E+000 | 5.92E-001 | 1.00E+000 | 1.00E+000 | 3.87E-001 |
| 238 | 2 | 97 | *Helicobacter pylori kx2 npgec Fusobacterium nucleatum* | 1.00E+000 | 51 | 7.64E-003 | 7.56E-001 | 7.56E-001 | 1.00E+000 | 5.05E-001 | 1.39E-001 | 1.00E+000 | 1.00E+000 | 5.92E-001 | 1.00E+000 | 1.84E-001 | 1.00E+000 |
| 10 | 2 | 454 | *Helicobacter pylori kx2 mgep Helicobacter pylori kx1 npgec* | 1.00E+000 | 224 | 1.69E-011 | 1.00E+000 | 2.44E-001 | 1.00E+000 | 5.05E-001 | 1.39E-001 | 1.00E+000 | 1.00E+000 | 1.22E-001 | 1.00E+000 | 1.00E+000 | 1.00E+000 |
| 712 | 2 | 37 | *Helicobacter pylori kx1 mgep Aggregatibacter actinomycetemcomitans* | 1.00E+000 | 26 | 1.49E-002 | 7.56E-001 | 7.56E-001 | 1.00E+000 | 5.05E-001 | 1.39E-001 | 1.00E+000 | 1.00E+000 | 5.92E-001 | 1.00E+000 | 1.84E-001 | 1.00E+000 |
| 66 | 2 | 216 | *Staphylococcus aureus Pseudomonas aeruginosa* | 1.00E+000 | 140 | 7.71E-012 | 2.44E-001 | 1.00E+000 | 4.60E-001 | 9.23E-001 | 6.23E-001 | 1.00E+000 | 1.00E+000 | 1.00E+000 | 6.39E-002 | 1.00E+000 | 1.00E+000 |
| 306 | 2 | 77 | *Helicobacter pylori kx2 mgep Ehrlichia chaffeensis liberty* | 1.00E+000 | 124 | < 1.67E-026 | 1.00E+000 | 2.44E-001 | 1.00E+000 | 5.05E-001 | 6.23E-001 | 1.00E+000 | 1.00E+000 | 5.92E-001 | 1.00E+000 | 1.00E+000 | 3.87E-001 |
| 30 | 2 | 319 | *streptococcus pneumoniae d39 Helicobacter pylori kx1 npgec* | 1.00E+000 | 176 | 9.06E-006 | 7.56E-001 | 7.56E-001 | 4.60E-001 | 9.23E-001 | 1.39E-001 | 1.00E+000 | 1.00E+000 | 5.92E-001 | 4.60E-001 | 1.00E+000 | 1.00E+000 |
| 252 | 2 | 91 | *Ehrlichia chaffeensis arkansa Bifidobacterium bifidum* | 1.00E+000 | 68 | < 1.67E-026 | 1.00E+000 | 2.44E-001 | 4.60E-001 | 9.23E-001 | 1.00E+000 | 1.39E-001 | 1.00E+000 | 1.00E+000 | 1.00E+000 | 1.00E+000 | 3.87E-001 |
| 146 | 2 | 134 | *Staphylococcus aureus Escherichia coli* | 1.00E+000 | 123 | 9.52E-006 | 2.44E-001 | 1.00E+000 | 4.60E-001 | 9.23E-001 | 6.23E-001 | 1.00E+000 | 4.95E-001 | 5.92E-001 | 4.60E-001 | 1.00E+000 | 1.00E+000 |
| 1952 | 3 | 2 | *Burkholderia pseudomallei Eubacterium rectale Helicobacter pylori kx2 mgep* | 1.00E+000 | 0 | 1.00E+000 | 8.84E-001 | 5.00E-001 | 6.08E-001 | 8.08E-001 | 7.74E-001 | 1.00E+000 | 6.46E-001 | 2.87E-001 | 1.00E+000 | 1.00E+000 | 5.25E-001 |
| 1946 | 3 | 2 | *Pseudomonas aeruginosa fdr1 Pseudomonas aeruginosa fdr440 Fusobacterium nucleatum* | 1.00E+000 | 0 | 1.00E+000 | 1.16E-001 | 1.00E+000 | 1.00E+000 | 3.54E-001 | 4.88E-002 | 1.00E+000 | 1.00E+000 | 1.00E+000 | 1.63E-001 | 2.65E-001 | 1.00E+000 |
| 1950 | 3 | 2 | *Pseudomonas aeruginosa Brucella ovis Escherichia coli* | 1.00E+000 | 0 | 1.00E+000 | 5.00E-001 | 8.84E-001 | 1.00E+000 | 3.54E-001 | 1.00E+000 | 1.00E+000 | 1.92E-001 | 7.45E-001 | 6.08E-001 | 1.00E+000 | 5.25E-001 |
| 1948 | 3 | 2 | *Burkholderia pseudomallei streptococcus gordonii Pseudomonas aeruginosa fdr1* | 1.00E+000 | 0 | 1.00E+000 | 1.16E-001 | 1.00E+000 | 6.08E-001 | 8.08E-001 | 3.21E-001 | 1.00E+000 | 6.46E-001 | 1.00E+000 | 6.08E-001 | 2.65E-001 | 5.25E-001 |
| 1940 | 3 | 2 | *Ehrlichia chaffeensis wakulla Pseudomonas aeruginosa fdr1234 Ehrlichia chaffeensis liberty* | 1.00E+000 | 4 | 1.65E-003 | 8.84E-001 | 5.00E-001 | 1.00E+000 | 3.54E-001 | 7.74E-001 | 1.00E+000 | 1.00E+000 | 1.00E+000 | 6.08E-001 | 1.00E+000 | 1.11E-001 |
| 1938 | 3 | 2 | *Burkholderia pseudomallei streptococcus gordonii Listeria monocytogenes* | 1.00E+000 | 0 | 1.00E+000 | 1.16E-001 | 1.00E+000 | 1.63E-001 | 9.81E-001 | 7.74E-001 | 2.04E-001 | 6.46E-001 | 7.45E-001 | 1.00E+000 | 2.65E-001 | 5.25E-001 |
| 1956 | 3 | 2 | *Ehrlichia chaffeensis arkansa Listeria monocytogenes Pseudomonas aeruginosa* | 1.00E+000 | 6 | 6.85E-005 | 5.00E-001 | 8.84E-001 | 6.08E-001 | 8.08E-001 | 1.00E+000 | 2.04E-001 | 1.00E+000 | 7.45E-001 | 6.08E-001 | 1.00E+000 | 5.25E-001 |
| 1932 | 4 | 2 | *Ehrlichia chaffeensis wakulla Pseudomonas aeruginosa fdr440 Aeromonas cavia Helicobacter pylori kx1 npgec* | 1.00E+000 | 0 | 1.00E+000 | 9.47E-001 | 3.03E-001 | 1.00E+000 | 2.45E-001 | 4.95E-001 | 1.00E+000 | 1.00E+000 | 4.51E-001 | 7.19E-001 | 1.00E+000 | 6.34E-001 |
| 1924 | 4 | 2 | *Burkholderia pseudomallei Brucella ovis Brucella neotomae Helicobacter pylori kx1 npgec* | 1.00E+000 | 0 | 1.00E+000 | 9.47E-001 | 3.03E-001 | 1.00E+000 | 2.45E-001 | 8.66E-001 | 1.00E+000 | 6.34E-002 | 8.43E-001 | 1.00E+000 | 1.00E+000 | 2.59E-002 |
| 1934 | 4 | 2 | *Yersinia enterocolitica wap bl6 Pseudomonas aeruginosa fdr875 Aeromonas cavia Helicobacter pylori kx1 npgec* | 1.00E+000 | 0 | 1.00E+000 | 9.47E-001 | 3.03E-001 | 1.00E+000 | 2.45E-001 | 4.95E-001 | 1.00E+000 | 7.55E-001 | 1.22E-001 | 7.19E-001 | 1.00E+000 | 1.00E+000 |
| 1936 | 4 | 2 | *Helicobacter pylori kx2 npgec Brucella neotomae Aeromonas cavia Helicobacter pylori kx1 npgec* | 1.00E+000 | 0 | 1.00E+000 | 1.00E+000 | 5.35E-002 | 1.00E+000 | 2.45E-001 | 4.95E-001 | 1.00E+000 | 7.55E-001 | 1.22E-001 | 1.00E+000 | 1.00E+000 | 6.34E-001 |
| 1926 | 4 | 2 | *Helicobacter pylori kx2 npgec streptococcus gordonii Brucella ovis Helicobacter pylori kx1 npgec* | 1.00E+000 | 0 | 1.00E+000 | 9.47E-001 | 3.03E-001 | 7.19E-001 | 6.80E-001 | 1.46E-001 | 1.00E+000 | 7.55E-001 | 4.51E-001 | 1.00E+000 | 3.41E-001 | 6.34E-001 |
| 1928 | 4 | 2 | *Helicobacter pylori kx2 npgec Anaplasma phagocytophilum Fusobacterium nucleatum Helicobacter pylori kx1 npgec* | 1.00E+000 | 0 | 1.00E+000 | 6.97E-001 | 6.97E-001 | 1.00E+000 | 2.45E-001 | 1.46E-001 | 1.00E+000 | 1.00E+000 | 4.51E-001 | 1.00E+000 | 3.41E-001 | 6.34E-001 |
| 1930 | 4 | 2 | *Helicobacter pylori kx2 npgec Burkholderia pseudomallei Brucella ovis Escherichia coli* | 1.00E+000 | 0 | 1.00E+000 | 6.97E-001 | 6.97E-001 | 1.00E+000 | 2.45E-001 | 8.66E-001 | 1.00E+000 | 6.34E-002 | 4.51E-001 | 1.00E+000 | 1.00E+000 | 1.96E-001 |
| 1914 | 4 | 2 | *Ehrlichia chaffeensis arkansa Burkholderia pseudomallei Listeria monocytogenes Escherichia coli* | 1.00E+000 | 8 | 3.38E-009 | 3.03E-001 | 9.47E-001 | 7.19E-001 | 6.80E-001 | 1.00E+000 | 2.65E-001 | 3.20E-001 | 4.51E-001 | 1.00E+000 | 1.00E+000 | 1.96E-001 |
| 1922 | 4 | 2 | *Staphylococcus aureus Bifidobacterium bifidum Brucella ovis Escherichia coli* | 1.00E+000 | 0 | 1.00E+000 | 6.97E-001 | 6.97E-001 | 2.77E-001 | 9.37E-001 | 8.66E-001 | 2.65E-001 | 3.20E-001 | 8.43E-001 | 7.19E-001 | 1.00E+000 | 6.34E-001 |
| 1886 | 5 | 2 | *Helicobacter pylori kx1 mgep Lactobacillus acidophilus Pseudomonas aeruginosa fdr440 Porphyromonas gingivalis Helicobacter pylori kx2 mgep* | 1.00E+000 | 0 | 1.00E+000 | 8.28E-001 | 5.00E-001 | 8.00E-001 | 5.54E-001 | 6.08E-002 | 3.23E-001 | 1.00E+000 | 5.96E-001 | 8.00E-001 | 4.10E-001 | 1.00E+000 |
| 1894 | 5 | 2 | *Yersinia enterocolitica p60 bc Pseudomonas aeruginosa fdr875 Porphyromonas gingivalis Mycobacterium tuberculosis Aggregatibacter actinomycetemcomitans* | 1.00E+000 | 1 | 2.40E-001 | 1.72E-001 | 9.76E-001 | 1.00E+000 | 5.54E-001 | 2.75E-001 | 1.00E+000 | 4.46E-001 | 9.05E-001 | 3.93E-001 | 6.29E-002 | 1.00E+000 |
| 1884 | 5 | 2 | *Helicobacter pylori kx1 mgep Pseudomonas aeruginosa fdr1 Pseudomonas aeruginosa fdr1234 Helicobacter pylori kx2 mgep Aggregatibacter actinomycetemcomitans* | 1.00E+000 | 2 | 4.07E-002 | 5.00E-001 | 8.28E-001 | 1.00E+000 | 1.68E-001 | 5.13E-003 | 1.00E+000 | 1.00E+000 | 5.96E-001 | 3.93E-001 | 4.10E-001 | 1.00E+000 |
| 1878 | 5 | 2 | *Helicobacter pylori kx2 npgec Bacillus anthracis Pseudomonas aeruginosa Helicobacter pylori kx1 npgec Escherichia coli* | 1.00E+000 | 0 | 1.00E+000 | 5.00E-001 | 8.28E-001 | 8.00E-001 | 5.54E-001 | 6.41E-001 | 1.00E+000 | 4.46E-001 | 2.35E-001 | 8.00E-001 | 1.00E+000 | 7.21E-001 |
| 1892 | 5 | 2 | *Burkholderia pseudomallei Pseudomonas aeruginosa fdr875 Bifidobacterium bifidum Helicobacter pylori kx2 mgep Aeromonas cavia* | 1.00E+000 | 0 | 1.00E+000 | 8.28E-001 | 5.00E-001 | 8.00E-001 | 5.54E-001 | 6.41E-001 | 3.23E-001 | 8.32E-001 | 5.96E-001 | 8.00E-001 | 1.00E+000 | 7.21E-001 |
| 1890 | 5 | 2 | *Helicobacter pylori kx2 npgec Pseudomonas aeruginosa fdr875 Listeria monocytogenes Porphyromonas gingivalis Helicobacter pylori kx1 npgec* | 1.00E+000 | 0 | 1.00E+000 | 5.00E-001 | 8.28E-001 | 8.00E-001 | 5.54E-001 | 6.08E-002 | 3.23E-001 | 1.00E+000 | 2.35E-001 | 8.00E-001 | 4.10E-001 | 1.00E+000 |
| 1882 | 5 | 2 | *Helicobacter pylori kx2 npgec Brucella ovis Aeromonas cavia Helicobacter pylori kx1 npgec Escherichia coli* | 1.00E+000 | 0 | 1.00E+000 | 9.76E-001 | 1.72E-001 | 1.00E+000 | 1.68E-001 | 6.41E-001 | 1.00E+000 | 4.46E-001 | 4.69E-002 | 1.00E+000 | 1.00E+000 | 7.21E-001 |
| 1898 | 5 | 2 | *Ehrlichia chaffeensis arkansa Ehrlichia chaffeensis wakulla Pseudomonas aeruginosa fdr875 Staphylococcus aureus Aggregatibacter actinomycetemcomitans* | 1.00E+000 | 1 | 2.40E-001 | 5.00E-001 | 8.28E-001 | 8.00E-001 | 5.54E-001 | 2.75E-001 | 1.00E+000 | 1.00E+000 | 1.00E+000 | 3.93E-001 | 4.10E-001 | 2.88E-001 |
| 1888 | 5 | 2 | *Ehrlichia chaffeensis arkansa Burkholderia pseudomallei Ehrlichia chaffeensis wakulla Bifidobacterium bifidum Brucella ovis* | 1.00E+000 | 0 | 1.00E+000 | 9.76E-001 | 1.72E-001 | 8.00E-001 | 5.54E-001 | 1.00E+000 | 3.23E-001 | 4.46E-001 | 1.00E+000 | 1.00E+000 | 1.00E+000 | 5.04E-003 |
| 1880 | 5 | 2 | *Burkholderia pseudomallei Ehrlichia chaffeensis wakulla Mycobacterium tuberculosis Aeromonas cavia streptococcus pneumoniae d39* | 1.00E+000 | 0 | 1.00E+000 | 5.00E-001 | 8.28E-001 | 8.00E-001 | 8.69E-001 | 9.23E-001 | 1.00E+000 | 4.46E-001 | 9.05E-001 | 3.93E-001 | 1.00E+000 | 2.88E-001 |
| 1896 | 5 | 2 | *Yersinia enterocolitica wap bl6 Helicobacter pylori kx2 npgec Ehrlichia chaffeensis wakulla Pseudomonas aeruginosa fdr875 Bifidobacterium bifidum* | 1.00E+000 | 0 | 1.00E+000 | 9.76E-001 | 1.72E-001 | 8.00E-001 | 5.54E-001 | 6.41E-001 | 3.23E-001 | 8.32E-001 | 5.96E-001 | 8.00E-001 | 1.00E+000 | 7.21E-001 |
| 1870 | 6 | 2 | *Helicobacter pylori kx1 mgep Burkholderia pseudomallei Pseudomonas aeruginosa fdr1 Pseudomonas aeruginosa fdr440 Helicobacter pylori kx2 mgep Aggregatibacter actinomycetemcomitans* | 1.00E+000 | 0 | 1.00E+000 | 3.31E-001 | 9.08E-001 | 1.00E+000 | 1.13E-001 | 2.32E-002 | 1.00E+000 | 8.87E-001 | 7.13E-001 | 5.03E-001 | 4.74E-001 | 7.89E-001 |
| 1842 | 6 | 2 | *Burkholderia pseudomallei streptococcus gordonii Lactobacillus acidophilus Pseudomonas aeruginosa fdr440 Fusobacterium nucleatum Aeromonas cavia* | 1.00E+000 | 0 | 1.00E+000 | 3.31E-001 | 9.08E-001 | 5.03E-001 | 7.84E-001 | 4.13E-001 | 3.78E-001 | 8.87E-001 | 9.44E-001 | 8.60E-001 | 9.10E-002 | 7.89E-001 |
| 1844 | 6 | 2 | *Yersinia enterocolitica wap bl6 streptococcus gordonii Pseudomonas aeruginosa fdr440 Pseudomonas aeruginosa streptococcus pneumoniae d39 Helicobacter pylori kx1 npgec* | 1.00E+000 | 0 | 1.00E+000 | 3.31E-001 | 9.08E-001 | 5.03E-001 | 7.84E-001 | 1.36E-001 | 1.00E+000 | 8.87E-001 | 7.13E-001 | 1.73E-001 | 4.74E-001 | 1.00E+000 |
| 1822 | 6 | 2 | *Pseudomonas aeruginosa fdr1234 Pseudomonas aeruginosa fdr875 Pseudomonas aeruginosa fdr440 Staphylococcus aureus Bifidobacterium bifidum Helicobacter pylori kx2 mgep* | 1.00E+000 | 0 | 1.00E+000 | 3.31E-001 | 9.08E-001 | 5.03E-001 | 7.84E-001 | 2.32E-002 | 3.78E-001 | 1.00E+000 | 9.44E-001 | 3.21E-002 | 1.00E+000 | 1.00E+000 |
| 1866 | 6 | 2 | *Helicobacter pylori kx2 npgec Ehrlichia chaffeensis arkansa Ehrlichia chaffeensis wakulla Mycobacterium tuberculosis Helicobacter pylori kx1 npgec Aggregatibacter actinomycetemcomitans* | 1.00E+000 | 0 | 1.00E+000 | 9.08E-001 | 3.31E-001 | 1.00E+000 | 4.39E-001 | 4.13E-001 | 1.00E+000 | 8.87E-001 | 7.13E-001 | 8.60E-001 | 4.74E-001 | 3.82E-001 |
| 1858 | 6 | 2 | *Helicobacter pylori kx2 npgec Burkholderia pseudomallei streptococcus pyogenes Porphyromonas gingivalis streptococcus pneumoniae d39 Helicobacter pylori kx1 npgec* | 1.00E+000 | 0 | 1.00E+000 | 6.69E-001 | 6.69E-001 | 5.03E-001 | 7.84E-001 | 1.36E-001 | 1.00E+000 | 8.87E-001 | 7.13E-001 | 8.60E-001 | 4.74E-001 | 7.89E-001 |
| 1874 | 6 | 2 | *Helicobacter pylori kx2 npgec Lactobacillus acidophilus Bacillus anthracis Pseudomonas aeruginosa fdr440 Bifidobacterium bifidum Helicobacter pylori kx1 npgec* | 1.00E+000 | 0 | 1.00E+000 | 9.08E-001 | 3.31E-001 | 1.73E-001 | 9.54E-001 | 4.13E-001 | 4.88E-002 | 8.87E-001 | 7.13E-001 | 8.60E-001 | 1.00E+000 | 7.89E-001 |
| 1868 | 6 | 2 | *Helicobacter pylori kx2 npgec Staphylococcus aureus Bifidobacterium bifidum streptococcus pyogenes streptococcus pneumoniae d39 Helicobacter pylori kx1 npgec* | 1.00E+000 | 0 | 1.00E+000 | 9.08E-001 | 3.31E-001 | 3.21E-002 | 9.95E-001 | 1.36E-001 | 3.78E-001 | 1.00E+000 | 7.13E-001 | 5.03E-001 | 1.00E+000 | 1.00E+000 |
| 1856 | 6 | 2 | *Yersinia enterocolitica wap bl6 Yersinia enterocolitica p60 bc Anaplasma phagocytophilum Porphyromonas gingivalis Helicobacter pylori kx2 mgep Aggregatibacter actinomycetemcomitans* | 1.00E+000 | 2 | 1.20E-001 | 6.69E-001 | 6.69E-001 | 1.00E+000 | 1.13E-001 | 4.13E-001 | 1.00E+000 | 5.61E-001 | 3.61E-001 | 1.00E+000 | 9.10E-002 | 7.89E-001 |
| 1850 | 6 | 2 | *Helicobacter pylori kx1 mgep Ehrlichia chaffeensis arkansa Burkholderia pseudomallei Pseudomonas aeruginosa fdr440 Helicobacter pylori kx2 mgep Helicobacter pylori kx1 npgec* | 1.00E+000 | 0 | 1.00E+000 | 9.08E-001 | 3.31E-001 | 1.00E+000 | 1.13E-001 | 1.36E-001 | 1.00E+000 | 8.87E-001 | 3.61E-001 | 8.60E-001 | 1.00E+000 | 3.82E-001 |
| 1864 | 6 | 2 | *Yersinia enterocolitica wap bl6 Helicobacter pylori kx2 npgec Pseudomonas aeruginosa fdr440 Staphylococcus aureus streptococcus pyogenes Aeromonas cavia* | 1.00E+000 | 0 | 1.00E+000 | 9.08E-001 | 3.31E-001 | 5.03E-001 | 7.84E-001 | 4.13E-001 | 1.00E+000 | 8.87E-001 | 3.61E-001 | 5.03E-001 | 1.00E+000 | 1.00E+000 |
| 1872 | 6 | 2 | *Burkholderia pseudomallei Lactobacillus acidophilus Pseudomonas aeruginosa fdr875 Staphylococcus aureus Aeromonas cavia Aggregatibacter actinomycetemcomitans* | 1.00E+000 | 0 | 1.00E+000 | 3.31E-001 | 9.08E-001 | 5.03E-001 | 7.84E-001 | 4.13E-001 | 3.78E-001 | 8.87E-001 | 9.44E-001 | 5.03E-001 | 4.74E-001 | 7.89E-001 |
| 1848 | 6 | 2 | *Helicobacter pylori kx2 npgec Eubacterium rectale Ehrlichia chaffeensis wakulla Helicobacter pylori kx2 mgep Aeromonas cavia Helicobacter pylori kx1 npgec* | 1.00E+000 | 0 | 1.00E+000 | 1.00E+000 | 1.03E-002 | 8.60E-001 | 4.39E-001 | 4.13E-001 | 1.00E+000 | 1.00E+000 | 1.64E-002 | 1.00E+000 | 1.00E+000 | 7.89E-001 |
| 1830 | 6 | 2 | *Helicobacter pylori kx2 npgec Ehrlichia chaffeensis arkansa Ehrlichia chaffeensis wakulla Brucella ovis Helicobacter pylori kx1 npgec Escherichia coli* | 1.00E+000 | 4 | 6.63E-002 | 9.90E-001 | 9.18E-002 | 1.00E+000 | 1.13E-001 | 7.55E-001 | 1.00E+000 | 5.61E-001 | 3.61E-001 | 1.00E+000 | 1.00E+000 | 1.01E-001 |
| 1846 | 6 | 2 | *Lactobacillus acidophilus Bifidobacterium bifidum streptococcus pyogenes Mycobacterium tuberculosis Helicobacter pylori kx1 npgec Escherichia coli* | 1.00E+000 | 0 | 1.00E+000 | 9.08E-001 | 3.31E-001 | 1.73E-001 | 9.95E-001 | 9.56E-001 | 4.88E-002 | 5.61E-001 | 7.13E-001 | 8.60E-001 | 1.00E+000 | 1.00E+000 |
| 1810 | 7 | 2 | *Yersinia enterocolitica wap bl6 Helicobacter pylori kx2 npgec Burkholderia pseudomallei Pseudomonas aeruginosa fdr875 Staphylococcus aureus Helicobacter pylori kx1 npgec Escherichia coli* | 1.00E+000 | 0 | 1.00E+000 | 5.00E-001 | 7.95E-001 | 9.03E-001 | 3.40E-001 | 2.36E-001 | 1.00E+000 | 3.12E-001 | 1.92E-001 | 6.02E-001 | 1.00E+000 | 8.42E-001 |
| 1816 | 7 | 2 | *Helicobacter pylori kx2 npgec Burkholderia pseudomallei Lactobacillus acidophilus Bifidobacterium bifidum Pseudomonas aeruginosa Brucella ovis Helicobacter pylori kx1 npgec* | 1.00E+000 | 0 | 1.00E+000 | 9.53E-001 | 2.05E-001 | 6.02E-001 | 6.88E-001 | 8.39E-001 | 6.71E-002 | 6.60E-001 | 8.03E-001 | 9.03E-001 | 1.00E+000 | 4.72E-001 |
| 1814 | 7 | 2 | *Ehrlichia chaffeensis arkansa Pseudomonas aeruginosa fdr440 Pseudomonas aeruginosa Helicobacter pylori kx2 mgep streptococcus pneumoniae d39 Helicobacter pylori kx1 npgec Escherichia coli* | 1.00E+000 | 0 | 1.00E+000 | 5.00E-001 | 7.95E-001 | 9.03E-001 | 3.40E-001 | 2.36E-001 | 1.00E+000 | 9.25E-001 | 4.88E-001 | 2.56E-001 | 1.00E+000 | 8.42E-001 |
| 1812 | 7 | 2 | *Burkholderia pseudomallei Pseudomonas aeruginosa fdr440 Anaplasma phagocytophilum Mycobacterium tuberculosis Aeromonas cavia Helicobacter pylori kx1 npgec Escherichia coli* | 1.00E+000 | 0 | 1.00E+000 | 2.05E-001 | 9.53E-001 | 1.00E+000 | 3.40E-001 | 8.39E-001 | 1.00E+000 | 3.12E-001 | 4.88E-001 | 6.02E-001 | 1.00E+000 | 4.72E-001 |
| 1768 | 8 | 2 | *Helicobacter pylori kx2 npgec Burkholderia pseudomallei Lactobacillus acidophilus Aeromonas cavia Helicobacter pylori kx1 npgec Pseudomonas aeruginosa fdr1 Helicobacter pylori kx2 mgep Escherichia coli* | 1.00E+000 | 1 | 3.67E-001 | 8.81E-001 | 3.48E-001 | 9.33E-001 | 2.57E-001 | 3.51E-001 | 4.79E-001 | 7.43E-001 | 9.08E-002 | 9.33E-001 | 1.00E+000 | 8.82E-001 |
| 1732 | 8 | 2 | *Burkholderia pseudomallei Lactobacillus acidophilus Bifidobacterium bifidum Helicobacter pylori kx1 npgec Ehrlichia chaffeensis liberty Pseudomonas aeruginosa fdr1 Helicobacter pylori kx2 mgep Escherichia coli* | 1.00E+000 | 0 | 1.00E+000 | 8.81E-001 | 3.48E-001 | 6.88E-001 | 5.89E-001 | 6.64E-001 | 8.78E-002 | 7.43E-001 | 6.05E-001 | 9.33E-001 | 1.00E+000 | 5.57E-001 |
| 1772 | 8 | 2 | *Helicobacter pylori kx2 npgec Ehrlichia chaffeensis arkansa Burkholderia pseudomallei Ehrlichia chaffeensis wakulla Porphyromonas gingivalis Helicobacter pylori kx2 mgep Helicobacter pylori kx1 npgec Ehrlichia chaffeensis liberty* | 1.00E+000 | 0 | 1.00E+000 | 9.78E-001 | 1.19E-001 | 1.00E+000 | 4.96E-002 | 3.51E-001 | 1.00E+000 | 9.50E-001 | 6.05E-001 | 1.00E+000 | 5.86E-001 | 4.99E-002 |
| 1760 | 8 | 2 | *Yersinia enterocolitica wap bl6 Helicobacter pylori kx2 npgec Helicobacter pylori kx1 mgep Burkholderia pseudomallei Helicobacter pylori kx1 npgec Ehrlichia chaffeensis liberty Ehrlichia chaffeensis wakulla Helicobacter pylori kx2 mgep* | 1.00E+000 | 0 | 1.00E+000 | 9.98E-001 | 2.24E-002 | 1.00E+000 | 4.96E-002 | 3.51E-001 | 1.00E+000 | 7.43E-001 | 9.08E-002 | 1.00E+000 | 1.00E+000 | 2.19E-001 |
| 1762 | 8 | 2 | *Yersinia enterocolitica wap bl6 Yersinia enterocolitica p60 bc Ehrlichia chaffeensis wakulla Bifidobacterium bifidum Porphyromonas gingivalis Mycobacterium tuberculosis streptococcus pneumoniae d39 Aggregatibacter actinomycetemcomitans* | 1.00E+000 | 0 | 1.00E+000 | 6.52E-001 | 6.52E-001 | 6.88E-001 | 8.54E-001 | 6.64E-001 | 4.79E-001 | 4.11E-001 | 8.68E-001 | 6.88E-001 | 1.58E-001 | 8.82E-001 |
| 1734 | 8 | 2 | *Helicobacter pylori kx2 npgec Listeria monocytogenes Porphyromonas gingivalis Helicobacter pylori kx1 npgec Aggregatibacter actinomycetemcomitans Mycobacterium tuberculosis Helicobacter pylori kx2 mgep Escherichia coli* | 1.00E+000 | 0 | 1.00E+000 | 3.48E-001 | 8.81E-001 | 9.33E-001 | 5.89E-001 | 1.21E-001 | 4.79E-001 | 7.43E-001 | 9.08E-002 | 9.33E-001 | 1.58E-001 | 1.00E+000 |
| 1758 | 8 | 2 | *Yersinia enterocolitica wap bl6 Helicobacter pylori kx2 npgec Lactobacillus acidophilus Bifidobacterium bifidum Helicobacter pylori kx1 npgec Mycobacterium tuberculosis Helicobacter pylori kx2 mgep Escherichia coli* | 1.00E+000 | 0 | 1.00E+000 | 9.78E-001 | 1.19E-001 | 6.88E-001 | 8.54E-001 | 6.64E-001 | 8.78E-002 | 4.11E-001 | 9.08E-002 | 9.33E-001 | 1.00E+000 | 1.00E+000 |
| 1766 | 8 | 2 | *Yersinia enterocolitica wap bl6 Helicobacter pylori kx2 npgec Staphylococcus aureus Listeria monocytogenes Helicobacter pylori kx1 npgec Ehrlichia chaffeensis liberty Ehrlichia chaffeensis arkansa Escherichia coli* | 1.00E+000 | 0 | 1.00E+000 | 8.81E-001 | 3.48E-001 | 6.88E-001 | 5.89E-001 | 6.64E-001 | 4.79E-001 | 7.43E-001 | 9.08E-002 | 9.33E-001 | 1.00E+000 | 5.57E-001 |
| 1770 | 8 | 2 | *Yersinia enterocolitica wap bl6 Helicobacter pylori kx2 npgec Helicobacter pylori kx1 mgep Aeromonas cavia Helicobacter pylori kx1 npgec Ehrlichia chaffeensis liberty streptococcus pyogenes Helicobacter pylori kx2 mgep* | 1.00E+000 | 0 | 1.00E+000 | 1.00E+000 | 1.72E-003 | 9.33E-001 | 2.57E-001 | 3.51E-001 | 1.00E+000 | 9.50E-001 | 1.64E-002 | 1.00E+000 | 1.00E+000 | 8.82E-001 |
| 1740 | 8 | 2 | *streptococcus gordonii Pseudomonas aeruginosa fdr440 Porphyromonas gingivalis Mycobacterium tuberculosis Fusobacterium nucleatum Aeromonas cavia Escherichia coli Aggregatibacter actinomycetemcomitans* | 1.00E+000 | 0 | 1.00E+000 | 2.24E-002 | 9.98E-001 | 9.33E-001 | 5.89E-001 | 1.21E-001 | 1.00E+000 | 7.43E-001 | 8.68E-001 | 6.88E-001 | 6.25E-004 | 1.00E+000 |
| 1742 | 8 | 2 | *Burkholderia pseudomallei Yersinia enterocolitica p60 bl6 Yersinia enterocolitica p60 bc Pseudomonas aeruginosa fdr875 Porphyromonas gingivalis Aggregatibacter actinomycetemcomitans Pseudomonas aeruginosa fdr1 Pseudomonas aeruginosa fdr440* | 1.00E+000 | 0 | 1.00E+000 | 1.19E-001 | 9.78E-001 | 1.00E+000 | 4.96E-002 | 1.21E-001 | 1.00E+000 | 4.11E-001 | 8.68E-001 | 3.45E-001 | 1.58E-001 | 8.82E-001 |
| 1756 | 8 | 2 | *Helicobacter pylori kx2 npgec Pseudomonas aeruginosa fdr1234 Lactobacillus acidophilus Pseudomonas aeruginosa fdr440 Staphylococcus aureus Pseudomonas aeruginosa Helicobacter pylori kx2 mgep Helicobacter pylori kx1 npgec* | 1.00E+000 | 0 | 1.00E+000 | 6.52E-001 | 6.52E-001 | 6.88E-001 | 5.89E-001 | 2.47E-002 | 4.79E-001 | 1.00E+000 | 6.05E-001 | 1.07E-001 | 1.00E+000 | 1.00E+000 |
| 1736 | 8 | 2 | *Helicobacter pylori kx2 npgec Ehrlichia chaffeensis arkansa Pseudomonas aeruginosa fdr1 Pseudomonas aeruginosa fdr440 Mycobacterium tuberculosis Helicobacter pylori kx1 npgec Escherichia coli Ehrlichia chaffeensis liberty* | 1.00E+000 | 0 | 1.00E+000 | 6.52E-001 | 6.52E-001 | 1.00E+000 | 2.57E-001 | 3.51E-001 | 1.00E+000 | 7.43E-001 | 6.05E-001 | 3.45E-001 | 1.00E+000 | 5.57E-001 |
| 1744 | 8 | 2 | *Burkholderia pseudomallei Staphylococcus aureus Listeria monocytogenes Pseudomonas aeruginosa Aeromonas cavia Helicobacter pylori kx1 npgec Ehrlichia chaffeensis liberty Pseudomonas aeruginosa fdr440* | 1.00E+000 | 0 | 1.00E+000 | 3.48E-001 | 8.81E-001 | 6.88E-001 | 5.89E-001 | 6.64E-001 | 4.79E-001 | 9.50E-001 | 6.05E-001 | 3.45E-001 | 1.00E+000 | 5.57E-001 |
| 1674 | 9 | 2 | *Yersinia enterocolitica wap bl6 Helicobacter pylori kx2 npgec Burkholderia pseudomallei Lactobacillus acidophilus Porphyromonas gingivalis Aeromonas cavia Helicobacter pylori kx1 npgec Ehrlichia chaffeensis arkansa Escherichia coli* | 1.00E+000 | 0 | 1.00E+000 | 9.35E-001 | 2.27E-001 | 9.55E-001 | 1.90E-001 | 7.60E-001 | 5.25E-001 | 5.09E-001 | 1.56E-001 | 1.00E+000 | 6.34E-001 | 6.33E-001 |
| 1678 | 9 | 2 | *Yersinia enterocolitica wap bl6 Helicobacter pylori kx2 npgec Burkholderia pseudomallei Yersinia enterocolitica p60 bl6 Yersinia enterocolitica p60 bc Porphyromonas gingivalis streptococcus pneumoniae d39 Helicobacter pylori kx1 npgec Pseudomonas aeruginosa fdr1* | 1.00E+000 | 0 | 1.00E+000 | 7.73E-001 | 5.00E-001 | 9.55E-001 | 1.90E-001 | 2.02E-001 | 1.00E+000 | 2.16E-001 | 1.56E-001 | 7.60E-001 | 6.34E-001 | 9.14E-001 |
| 1698 | 9 | 2 | *Yersinia enterocolitica wap bl6 Burkholderia pseudomallei Yersinia enterocolitica p60 bl6 Yersinia enterocolitica p60 bc Pseudomonas aeruginosa streptococcus pneumoniae d39 Helicobacter pylori kx1 npgec Ehrlichia chaffeensis liberty Mycobacterium tuberculosis* | 1.00E+000 | 0 | 1.00E+000 | 7.73E-001 | 5.00E-001 | 9.55E-001 | 4.91E-001 | 9.37E-001 | 1.00E+000 | 5.79E-002 | 4.04E-001 | 4.36E-001 | 1.00E+000 | 6.33E-001 |
| 1718 | 9 | 2 | *Helicobacter pylori kx2 npgec Staphylococcus aureus Pseudomonas aeruginosa Porphyromonas gingivalis Aeromonas cavia streptococcus pneumoniae d39 Helicobacter pylori kx1 npgec Ehrlichia chaffeensis liberty streptococcus gordonii* | 1.00E+000 | 0 | 1.00E+000 | 5.00E-001 | 7.73E-001 | 4.36E-001 | 7.84E-001 | 5.58E-002 | 1.00E+000 | 1.00E+000 | 7.06E-001 | 4.36E-001 | 1.96E-001 | 9.14E-001 |
| 1714 | 9 | 2 | *Burkholderia pseudomallei Pseudomonas aeruginosa fdr1234 Lactobacillus acidophilus Pseudomonas aeruginosa fdr875 Bifidobacterium bifidum Porphyromonas gingivalis Aggregatibacter actinomycetemcomitans Pseudomonas aeruginosa fdr440 Helicobacter pylori kx2 mgep* | 1.00E+000 | 0 | 1.00E+000 | 2.27E-001 | 9.35E-001 | 7.60E-001 | 4.91E-001 | 5.58E-002 | 1.11E-001 | 9.68E-001 | 9.89E-001 | 4.36E-001 | 1.96E-001 | 9.14E-001 |
| 1730 | 9 | 2 | *Helicobacter pylori kx2 npgec Listeria monocytogenes Porphyromonas gingivalis Helicobacter pylori kx1 npgec Bacillus anthracis Pseudomonas aeruginosa fdr1 Pseudomonas aeruginosa fdr440 Mycobacterium tuberculosis Escherichia coli* | 1.00E+000 | 0 | 1.00E+000 | 6.50E-002 | 9.90E-001 | 7.60E-001 | 7.84E-001 | 2.02E-001 | 5.25E-001 | 5.09E-001 | 4.04E-001 | 4.36E-001 | 6.34E-001 | 9.14E-001 |
| 1720 | 9 | 2 | *Helicobacter pylori kx2 npgec Helicobacter pylori kx1 mgep Pseudomonas aeruginosa streptococcus pneumoniae d39 Helicobacter pylori kx1 npgec Pseudomonas aeruginosa fdr440 Mycobacterium tuberculosis Helicobacter pylori kx2 mgep Escherichia coli* | 1.00E+000 | 0 | 1.00E+000 | 5.00E-001 | 7.73E-001 | 9.55E-001 | 4.91E-001 | 5.58E-002 | 1.00E+000 | 8.10E-001 | 1.56E-001 | 1.63E-001 | 1.00E+000 | 1.00E+000 |
| 1654 | 10 | 2 | *Helicobacter pylori kx2 npgec Yersinia enterocolitica p60 bl6 Lactobacillus acidophilus Staphylococcus aureus Bifidobacterium bifidum Pseudomonas aeruginosa Helicobacter pylori kx1 npgec Ehrlichia chaffeensis wakulla Helicobacter pylori kx2 mgep Escherichia coli* | 1.00E+000 | 0 | 1.00E+000 | 9.67E-001 | 1.39E-001 | 5.24E-001 | 7.04E-001 | 5.85E-001 | 1.36E-001 | 8.63E-001 | 2.39E-001 | 8.19E-001 | 1.00E+000 | 9.37E-001 |
| 1614 | 10 | 2 | *Helicobacter pylori kx2 npgec Lactobacillus acidophilus Bifidobacterium bifidum Pseudomonas aeruginosa Aeromonas cavia streptococcus pneumoniae d39 Helicobacter pylori kx1 npgec Ehrlichia chaffeensis liberty Helicobacter pylori kx2 mgep Escherichia coli* | 1.00E+000 | 1 | 3.07E-001 | 9.67E-001 | 1.39E-001 | 5.24E-001 | 7.04E-001 | 5.85E-001 | 1.36E-001 | 9.80E-001 | 2.39E-001 | 8.19E-001 | 1.00E+000 | 9.37E-001 |
| 1656 | 10 | 2 | *Helicobacter pylori kx2 npgec Burkholderia pseudomallei Lactobacillus acidophilus Staphylococcus aureus Aeromonas cavia Helicobacter pylori kx1 npgec Ehrlichia chaffeensis arkansa Ehrlichia chaffeensis wakulla Helicobacter pylori kx2 mgep Escherichia coli* | 1.00E+000 | 0 | 1.00E+000 | 9.67E-001 | 1.39E-001 | 8.19E-001 | 4.00E-001 | 5.85E-001 | 5.68E-001 | 8.63E-001 | 2.39E-001 | 9.70E-001 | 1.00E+000 | 3.62E-001 |
| 1664 | 10 | 2 | *Yersinia enterocolitica wap bl6 Helicobacter pylori kx2 npgec Yersinia enterocolitica p60 bc Lactobacillus acidophilus Anaplasma phagocytophilum Bifidobacterium bifidum Porphyromonas gingivalis streptococcus pneumoniae d39 Helicobacter pylori kx1 npgec Aggregatibacter actinomycetemcomitans* | 1.00E+000 | 0 | 1.00E+000 | 8.61E-001 | 3.59E-001 | 5.24E-001 | 7.04E-001 | 3.00E-001 | 1.36E-001 | 8.63E-001 | 5.13E-001 | 9.70E-001 | 2.36E-001 | 9.37E-001 |
| 1670 | 10 | 2 | *Yersinia enterocolitica wap bl6 Helicobacter pylori kx2 npgec Yersinia enterocolitica p60 bl6 Yersinia enterocolitica p60 bc Lactobacillus acidophilus Staphylococcus aureus Porphyromonas gingivalis Helicobacter pylori kx1 npgec Ehrlichia chaffeensis liberty Ehrlichia chaffeensis wakulla* | 1.00E+000 | 0 | 1.00E+000 | 9.96E-001 | 3.35E-002 | 8.19E-001 | 4.00E-001 | 5.85E-001 | 5.68E-001 | 6.00E-001 | 2.39E-001 | 9.70E-001 | 6.79E-001 | 7.01E-001 |
| 1658 | 10 | 2 | *Yersinia enterocolitica wap bl6 Helicobacter pylori kx2 npgec Burkholderia pseudomallei Yersinia enterocolitica p60 bl6 Yersinia enterocolitica p60 bc Bifidobacterium bifidum Porphyromonas gingivalis Helicobacter pylori kx1 npgec streptococcus pyogenes Escherichia coli* | 1.00E+000 | 0 | 1.00E+000 | 9.67E-001 | 1.39E-001 | 8.19E-001 | 4.00E-001 | 8.35E-001 | 5.68E-001 | 9.62E-002 | 7.41E-002 | 1.00E+000 | 6.79E-001 | 9.37E-001 |
| 1666 | 10 | 2 | *Helicobacter pylori kx2 npgec Burkholderia pseudomallei Lactobacillus acidophilus Staphylococcus aureus Bifidobacterium bifidum Pseudomonas aeruginosa streptococcus pneumoniae d39 Helicobacter pylori kx1 npgec Mycobacterium tuberculosis Helicobacter pylori kx2 mgep* | 1.00E+000 | 0 | 1.00E+000 | 6.41E-001 | 6.41E-001 | 2.29E-001 | 9.80E-001 | 3.00E-001 | 1.36E-001 | 8.63E-001 | 7.88E-001 | 2.29E-001 | 1.00E+000 | 9.37E-001 |
| 1624 | 10 | 2 | *Helicobacter pylori kx2 npgec Staphylococcus aureus Porphyromonas gingivalis streptococcus pneumoniae d39 Helicobacter pylori kx1 npgec Ehrlichia chaffeensis liberty Aggregatibacter actinomycetemcomitans streptococcus gordonii Helicobacter pylori kx2 mgep Escherichia coli* | 1.00E+000 | 0 | 1.00E+000 | 3.59E-001 | 8.61E-001 | 5.24E-001 | 7.04E-001 | 3.05E-003 | 1.00E+000 | 9.80E-001 | 5.13E-001 | 8.19E-001 | 3.62E-002 | 9.37E-001 |
| 1672 | 10 | 2 | *Helicobacter pylori kx2 npgec Burkholderia pseudomallei Lactobacillus acidophilus Porphyromonas gingivalis Aeromonas cavia streptococcus pneumoniae d39 Helicobacter pylori kx1 npgec Pseudomonas aeruginosa fdr1 Pseudomonas aeruginosa fdr440 Escherichia coli* | 1.00E+000 | 0 | 1.00E+000 | 3.59E-001 | 8.61E-001 | 8.19E-001 | 4.00E-001 | 1.05E-001 | 5.68E-001 | 8.63E-001 | 5.13E-001 | 5.24E-001 | 6.79E-001 | 9.37E-001 |
| 1632 | 10 | 2 | *Helicobacter pylori kx2 npgec Burkholderia pseudomallei Lactobacillus acidophilus Pseudomonas aeruginosa streptococcus pneumoniae d39 Helicobacter pylori kx1 npgec Aggregatibacter actinomycetemcomitans streptococcus gordonii Pseudomonas aeruginosa fdr440 Escherichia coli* | 1.00E+000 | 0 | 1.00E+000 | 1.39E-001 | 9.67E-001 | 5.24E-001 | 7.04E-001 | 1.05E-001 | 5.68E-001 | 8.63E-001 | 7.88E-001 | 5.24E-001 | 2.36E-001 | 9.37E-001 |
| 1668 | 10 | 2 | *Helicobacter pylori kx2 npgec Pseudomonas aeruginosa fdr875 Staphylococcus aureus Pseudomonas aeruginosa Porphyromonas gingivalis streptococcus pneumoniae d39 Helicobacter pylori kx1 npgec Pseudomonas aeruginosa fdr1 Pseudomonas aeruginosa fdr440 Helicobacter pylori kx2 mgep* | 1.00E+000 | 0 | 1.00E+000 | 1.39E-001 | 9.67E-001 | 8.19E-001 | 4.00E-001 | 2.08E-004 | 1.00E+000 | 1.00E+000 | 7.88E-001 | 1.09E-002 | 6.79E-001 | 1.00E+000 |
| 1620 | 10 | 2 | *Helicobacter pylori kx2 npgec Helicobacter pylori kx1 mgep Burkholderia pseudomallei Pseudomonas aeruginosa Aeromonas cavia Helicobacter pylori kx1 npgec Ehrlichia chaffeensis liberty streptococcus pyogenes Helicobacter pylori kx2 mgep Escherichia coli* | 1.00E+000 | 0 | 1.00E+000 | 9.67E-001 | 1.39E-001 | 9.70E-001 | 1.37E-001 | 5.85E-001 | 1.00E+000 | 8.63E-001 | 7.41E-002 | 9.70E-001 | 1.00E+000 | 7.01E-001 |
| 1576 | 11 | 2 | *Burkholderia pseudomallei Lactobacillus acidophilus Listeria monocytogenes Bifidobacterium bifidum Porphyromonas gingivalis Aeromonas cavia Ehrlichia chaffeensis liberty Pseudomonas aeruginosa fdr1 Pseudomonas aeruginosa fdr440 Mycobacterium tuberculosis Escherichia coli* | 1.00E+000 | 0 | 1.00E+000 | 2.42E-001 | 9.21E-001 | 6.07E-001 | 8.54E-001 | 8.91E-001 | 1.44E-002 | 6.82E-001 | 8.52E-001 | 6.07E-001 | 7.19E-001 | 7.60E-001 |
| 1578 | 11 | 2 | *Yersinia enterocolitica wap bl6 Helicobacter pylori kx2 npgec Burkholderia pseudomallei Lactobacillus acidophilus Bifidobacterium bifidum streptococcus pneumoniae d39 Helicobacter pylori kx1 npgec Ehrlichia chaffeensis liberty Aggregatibacter actinomycetemcomitans Pseudomonas aeruginosa fdr440 Escherichia coli* | 1.00E+000 | 0 | 1.00E+000 | 7.58E-001 | 5.00E-001 | 6.07E-001 | 6.18E-001 | 4.07E-001 | 1.63E-001 | 6.82E-001 | 6.16E-001 | 8.66E-001 | 7.19E-001 | 7.60E-001 |
| 1518 | 12 | 2 | *Helicobacter pylori kx2 npgec Burkholderia pseudomallei Lactobacillus acidophilus Bifidobacterium bifidum Pseudomonas aeruginosa Porphyromonas gingivalis Fusobacterium nucleatum Aeromonas cavia streptococcus pneumoniae d39 Helicobacter pylori kx1 npgec Aggregatibacter actinomycetemcomitans streptococcus gordonii* | 1.00E+000 | 0 | 1.00E+000 | 3.67E-001 | 8.47E-001 | 3.82E-001 | 7.93E-001 | 8.84E-002 | 1.92E-001 | 9.92E-001 | 9.01E-001 | 9.03E-001 | 4.42E-003 | 9.68E-001 |
| 1436 | 14 | 2 | *Yersinia enterocolitica wap bl6 Yersinia enterocolitica wap bc Burkholderia pseudomallei Yersinia enterocolitica p60 bl6 Yersinia enterocolitica p60 bc Lactobacillus acidophilus Anaplasma phagocytophilum Staphylococcus aureus Bifidobacterium bifidum Porphyromonas gingivalis Aeromonas cavia Helicobacter pylori kx1 npgec Aggregatibacter actinomycetemcomitans Mycobacterium tuberculosis* | 1.00E+000 | 0 | 1.00E+000 | 8.37E-001 | 3.72E-001 | 8.05E-001 | 6.47E-001 | 8.93E-001 | 2.54E-001 | 1.39E-001 | 3.63E-001 | 9.52E-001 | 4.07E-001 | 8.87E-001 |
| 208 | 6 | 36 | *Helicobacter pylori kx2 npgec Lactobacillus acidophilus Bifidobacterium bifidum Mycobacterium tuberculosis streptococcus pneumoniae d39 Helicobacter pylori kx1 npgec* | 1.03E-001 | 1 | 5.61E-001 | 9.08E-001 | 3.31E-001 | 1.73E-001 | 9.95E-001 | 4.13E-001 | 4.88E-002 | 8.87E-001 | 7.13E-001 | 5.03E-001 | 1.00E+000 | 1.00E+000 |
| 210 | 6 | 36 | *Helicobacter pylori kx2 npgec Pseudomonas aeruginosa fdr440 Bifidobacterium bifidum Mycobacterium tuberculosis Helicobacter pylori kx1 npgec Escherichia coli* | 1.03E-001 | 0 | 1.00E+000 | 6.69E-001 | 6.69E-001 | 8.60E-001 | 7.84E-001 | 4.13E-001 | 3.78E-001 | 5.61E-001 | 3.61E-001 | 5.03E-001 | 1.00E+000 | 1.00E+000 |
| 798 | 8 | 8 | *Helicobacter pylori kx2 npgec Burkholderia pseudomallei Lactobacillus acidophilus Pseudomonas aeruginosa fdr440 Pseudomonas aeruginosa Helicobacter pylori kx1 npgec Escherichia coli Ehrlichia chaffeensis liberty* | 1.04E-001 | 0 | 1.00E+000 | 6.52E-001 | 6.52E-001 | 9.33E-001 | 2.57E-001 | 6.64E-001 | 4.79E-001 | 7.43E-001 | 6.05E-001 | 6.88E-001 | 1.00E+000 | 5.57E-001 |
| 800 | 8 | 8 | *Yersinia enterocolitica wap bl6 Helicobacter pylori kx2 npgec Burkholderia pseudomallei Lactobacillus acidophilus Bifidobacterium bifidum Porphyromonas gingivalis Pseudomonas aeruginosa fdr1 Mycobacterium tuberculosis* | 1.04E-001 | 0 | 1.00E+000 | 6.52E-001 | 6.52E-001 | 6.88E-001 | 8.54E-001 | 6.64E-001 | 8.78E-002 | 4.11E-001 | 8.68E-001 | 6.88E-001 | 5.86E-001 | 8.82E-001 |
| 796 | 8 | 8 | *Helicobacter pylori kx2 npgec Lactobacillus acidophilus Bifidobacterium bifidum Pseudomonas aeruginosa streptococcus pneumoniae d39 Helicobacter pylori kx1 npgec Helicobacter pylori kx2 mgep Escherichia coli* | 1.04E-001 | 2 | 2.16E-001 | 8.81E-001 | 3.48E-001 | 3.45E-001 | 8.54E-001 | 3.51E-001 | 8.78E-002 | 9.50E-001 | 2.94E-001 | 6.88E-001 | 1.00E+000 | 1.00E+000 |
| 810 | 8 | 8 | *Helicobacter pylori kx2 npgec Burkholderia pseudomallei Pseudomonas aeruginosa fdr1234 Lactobacillus acidophilus Bifidobacterium bifidum Porphyromonas gingivalis Helicobacter pylori kx1 npgec Aggregatibacter actinomycetemcomitans* | 1.04E-001 | 6 | 4.44E-007 | 6.52E-001 | 6.52E-001 | 6.88E-001 | 5.89E-001 | 1.21E-001 | 8.78E-002 | 9.50E-001 | 8.68E-001 | 9.33E-001 | 1.58E-001 | 8.82E-001 |
| 806 | 8 | 8 | *Helicobacter pylori kx2 npgec Burkholderia pseudomallei streptococcus gordonii Lactobacillus acidophilus Bifidobacterium bifidum Mycobacterium tuberculosis Fusobacterium nucleatum Aeromonas cavia* | 1.04E-001 | 0 | 1.00E+000 | 6.52E-001 | 6.52E-001 | 3.45E-001 | 9.69E-001 | 6.64E-001 | 8.78E-002 | 7.43E-001 | 8.68E-001 | 9.33E-001 | 1.58E-001 | 8.82E-001 |
| 812 | 8 | 8 | *Yersinia enterocolitica wap bl6 Helicobacter pylori kx2 npgec Burkholderia pseudomallei Yersinia enterocolitica p60 bl6 Staphylococcus aureus Porphyromonas gingivalis Helicobacter pylori kx1 npgec Helicobacter pylori kx2 mgep* | 1.04E-001 | 8 | 1.25E-003 | 8.81E-001 | 3.48E-001 | 9.33E-001 | 2.57E-001 | 1.21E-001 | 1.00E+000 | 4.11E-001 | 9.08E-002 | 9.33E-001 | 5.86E-001 | 8.82E-001 |
| 794 | 8 | 8 | *Yersinia enterocolitica wap bl6 Lactobacillus acidophilus Staphylococcus aureus Bifidobacterium bifidum Pseudomonas aeruginosa Porphyromonas gingivalis Ehrlichia chaffeensis liberty Ehrlichia chaffeensis arkansa* | 1.04E-001 | 0 | 1.00E+000 | 8.81E-001 | 3.48E-001 | 3.45E-001 | 8.54E-001 | 8.98E-001 | 8.78E-002 | 9.50E-001 | 9.81E-001 | 6.88E-001 | 5.86E-001 | 5.57E-001 |
| 808 | 8 | 8 | *Helicobacter pylori kx2 npgec streptococcus pneumoniae d39 Helicobacter pylori kx1 npgec Ehrlichia chaffeensis liberty Aggregatibacter actinomycetemcomitans Pseudomonas aeruginosa fdr1 Pseudomonas aeruginosa fdr440 Escherichia coli* | 1.04E-001 | 0 | 1.00E+000 | 3.48E-001 | 8.81E-001 | 9.33E-001 | 2.57E-001 | 2.47E-002 | 1.00E+000 | 9.50E-001 | 6.05E-001 | 3.45E-001 | 5.86E-001 | 8.82E-001 |
| 802 | 8 | 8 | *Burkholderia pseudomallei Porphyromonas gingivalis Aeromonas cavia streptococcus pneumoniae d39 Helicobacter pylori kx1 npgec Ehrlichia chaffeensis liberty Pseudomonas aeruginosa fdr440 Escherichia coli* | 1.04E-001 | 0 | 1.00E+000 | 3.48E-001 | 8.81E-001 | 9.33E-001 | 2.57E-001 | 3.51E-001 | 1.00E+000 | 7.43E-001 | 6.05E-001 | 6.88E-001 | 5.86E-001 | 5.57E-001 |
| 430 | 8 | 15 | *Yersinia enterocolitica wap bl6 Helicobacter pylori kx2 npgec Yersinia enterocolitica p60 bc Staphylococcus aureus Porphyromonas gingivalis Aeromonas cavia Helicobacter pylori kx1 npgec Escherichia coli* | 1.09E-002 | 3 | 1.38E-001 | 8.81E-001 | 3.48E-001 | 9.33E-001 | 2.57E-001 | 3.51E-001 | 1.00E+000 | 4.11E-001 | 1.64E-002 | 9.33E-001 | 5.86E-001 | 1.00E+000 |
| 424 | 8 | 15 | *Yersinia enterocolitica wap bl6 Burkholderia pseudomallei Lactobacillus acidophilus Staphylococcus aureus Bifidobacterium bifidum Porphyromonas gingivalis Helicobacter pylori kx1 npgec Ehrlichia chaffeensis wakulla* | 1.09E-002 | 9 | 3.21E-004 | 8.81E-001 | 3.48E-001 | 3.45E-001 | 8.54E-001 | 6.64E-001 | 8.78E-002 | 7.43E-001 | 8.68E-001 | 9.33E-001 | 5.86E-001 | 5.57E-001 |
| 420 | 8 | 15 | *Helicobacter pylori kx2 npgec Lactobacillus acidophilus Pseudomonas aeruginosa Porphyromonas gingivalis Aeromonas cavia Helicobacter pylori kx1 npgec Aggregatibacter actinomycetemcomitans Escherichia coli* | 1.09E-002 | 0 | 1.00E+000 | 6.52E-001 | 6.52E-001 | 9.33E-001 | 2.57E-001 | 3.51E-001 | 4.79E-001 | 9.50E-001 | 2.94E-001 | 9.33E-001 | 1.58E-001 | 1.00E+000 |
| 72 | 5 | 81 | *Burkholderia pseudomallei Yersinia enterocolitica p60 bl6 Lactobacillus acidophilus Bifidobacterium bifidum Helicobacter pylori kx1 npgec* | 1.10E-001 | 33 | 8.36E-010 | 9.76E-001 | 1.72E-001 | 3.93E-001 | 8.69E-001 | 9.23E-001 | 3.31E-002 | 4.46E-001 | 5.96E-001 | 1.00E+000 | 1.00E+000 | 7.21E-001 |
| 492 | 7 | 15 | *Yersinia enterocolitica wap bl6 Helicobacter pylori kx2 npgec Ehrlichia chaffeensis arkansa Ehrlichia chaffeensis wakulla Staphylococcus aureus Bifidobacterium bifidum Helicobacter pylori kx1 npgec* | 1.16E-001 | 7 | 9.32E-006 | 9.96E-001 | 4.65E-002 | 6.02E-001 | 6.88E-001 | 5.46E-001 | 4.30E-001 | 9.25E-001 | 4.88E-001 | 9.03E-001 | 1.00E+000 | 4.72E-001 |
| 12 | 3 | 283 | *Helicobacter pylori kx2 npgec Pseudomonas aeruginosa Helicobacter pylori kx1 npgec* | 1.17E-001 | 102 | < 1.67E-026 | 8.84E-001 | 5.00E-001 | 1.00E+000 | 3.54E-001 | 3.21E-001 | 1.00E+000 | 1.00E+000 | 2.87E-001 | 6.08E-001 | 1.00E+000 | 1.00E+000 |
| 226 | 6 | 34 | *Pseudomonas aeruginosa fdr440 Staphylococcus aureus Porphyromonas gingivalis Aeromonas cavia streptococcus pneumoniae d39 Helicobacter pylori kx1 npgec* | 1.18E-001 | 3 | 3.27E-001 | 3.31E-001 | 9.08E-001 | 5.03E-001 | 7.84E-001 | 2.32E-002 | 1.00E+000 | 1.00E+000 | 7.13E-001 | 1.73E-001 | 4.74E-001 | 1.00E+000 |
| 1030 | 12 | 4 | *Yersinia enterocolitica wap bl6 Helicobacter pylori kx2 npgec Yersinia enterocolitica p60 bc Lactobacillus acidophilus Staphylococcus aureus Bifidobacterium bifidum Pseudomonas aeruginosa Porphyromonas gingivalis Aeromonas cavia Helicobacter pylori kx1 npgec Ehrlichia chaffeensis liberty Aggregatibacter actinomycetemcomitans* | 1.19E-002 | 0 | 1.00E+000 | 9.57E-001 | 1.53E-001 | 6.82E-001 | 5.31E-001 | 5.15E-001 | 1.92E-001 | 9.33E-001 | 4.34E-001 | 9.03E-001 | 3.20E-001 | 9.68E-001 |
| 242 | 6 | 32 | *Yersinia enterocolitica wap bl6 Helicobacter pylori kx2 npgec Burkholderia pseudomallei Lactobacillus acidophilus Bifidobacterium bifidum Mycobacterium tuberculosis* | 1.33E-001 | 0 | 1.00E+000 | 9.08E-001 | 3.31E-001 | 5.03E-001 | 9.54E-001 | 9.56E-001 | 4.88E-002 | 2.16E-001 | 7.13E-001 | 8.60E-001 | 1.00E+000 | 7.89E-001 |
| 534 | 7 | 14 | *Yersinia enterocolitica wap bl6 Helicobacter pylori kx2 npgec Burkholderia pseudomallei Ehrlichia chaffeensis wakulla Pseudomonas aeruginosa fdr1 Lactobacillus acidophilus Bifidobacterium bifidum* | 1.36E-001 | 0 | 1.00E+000 | 9.53E-001 | 2.05E-001 | 6.02E-001 | 6.88E-001 | 8.39E-001 | 6.71E-002 | 6.60E-001 | 8.03E-001 | 9.03E-001 | 1.00E+000 | 4.72E-001 |
| 532 | 7 | 14 | *Yersinia enterocolitica wap bl6 Burkholderia pseudomallei Yersinia enterocolitica p60 bl6 Ehrlichia chaffeensis wakulla Lactobacillus acidophilus Bifidobacterium bifidum Aggregatibacter actinomycetemcomitans* | 1.36E-001 | 7 | 5.18E-004 | 9.53E-001 | 2.05E-001 | 6.02E-001 | 6.88E-001 | 9.76E-001 | 6.71E-002 | 3.12E-001 | 8.03E-001 | 1.00E+000 | 5.32E-001 | 4.72E-001 |
| 530 | 7 | 14 | *Helicobacter pylori kx2 npgec Lactobacillus acidophilus Porphyromonas gingivalis Helicobacter pylori kx2 mgep Aeromonas cavia Helicobacter pylori kx1 npgec Aggregatibacter actinomycetemcomitans* | 1.36E-001 | 0 | 1.00E+000 | 9.53E-001 | 2.05E-001 | 9.03E-001 | 3.40E-001 | 6.08E-002 | 4.30E-001 | 1.00E+000 | 1.92E-001 | 1.00E+000 | 1.23E-001 | 1.00E+000 |
| 528 | 7 | 14 | *Yersinia enterocolitica wap bl6 Helicobacter pylori kx2 npgec Yersinia enterocolitica p60 bc Ehrlichia chaffeensis wakulla Porphyromonas gingivalis Helicobacter pylori kx1 npgec Aggregatibacter actinomycetemcomitans* | 1.36E-001 | 3 | 1.18E-001 | 9.53E-001 | 2.05E-001 | 1.00E+000 | 7.55E-002 | 2.36E-001 | 1.00E+000 | 6.60E-001 | 1.92E-001 | 1.00E+000 | 1.23E-001 | 8.42E-001 |
| 248 | 6 | 31 | *Yersinia enterocolitica wap bl6 Helicobacter pylori kx2 npgec Ehrlichia chaffeensis wakulla Lactobacillus acidophilus Aeromonas cavia Helicobacter pylori kx1 npgec* | 1.43E-001 | 12 | 8.36E-004 | 1.00E+000 | 1.03E-002 | 8.60E-001 | 4.39E-001 | 7.55E-001 | 3.78E-001 | 8.87E-001 | 1.08E-001 | 1.00E+000 | 1.00E+000 | 7.89E-001 |
| 466 | 8 | 14 | *Helicobacter pylori kx2 npgec Burkholderia pseudomallei Staphylococcus aureus Bifidobacterium bifidum Pseudomonas aeruginosa streptococcus pneumoniae d39 Helicobacter pylori kx1 npgec Aggregatibacter actinomycetemcomitans* | 1.49E-002 | 0 | 1.00E+000 | 3.48E-001 | 8.81E-001 | 3.45E-001 | 8.54E-001 | 1.21E-001 | 4.79E-001 | 9.50E-001 | 8.68E-001 | 3.45E-001 | 5.86E-001 | 8.82E-001 |
| 1244 | 12 | 3 | *Yersinia enterocolitica wap bl6 Helicobacter pylori kx2 npgec Burkholderia pseudomallei Yersinia enterocolitica p60 bl6 Yersinia enterocolitica p60 bc Lactobacillus acidophilus Bifidobacterium bifidum Porphyromonas gingivalis streptococcus pneumoniae d39 Helicobacter pylori kx1 npgec Aggregatibacter actinomycetemcomitans Ehrlichia chaffeensis wakulla* | 1.53E-001 | 0 | 1.00E+000 | 9.57E-001 | 1.53E-001 | 6.82E-001 | 5.31E-001 | 5.15E-001 | 1.92E-001 | 4.69E-001 | 4.34E-001 | 9.87E-001 | 3.20E-001 | 8.10E-001 |
| 1200 | 12 | 3 | *Yersinia enterocolitica wap bl6 Helicobacter pylori kx2 npgec Staphylococcus aureus Listeria monocytogenes Porphyromonas gingivalis Aeromonas cavia Helicobacter pylori kx1 npgec Aggregatibacter actinomycetemcomitans Pseudomonas aeruginosa fdr1 Pseudomonas aeruginosa fdr440 Mycobacterium tuberculosis Escherichia coli* | 1.53E-001 | 0 | 1.00E+000 | 1.53E-001 | 9.57E-001 | 9.03E-001 | 5.31E-001 | 8.84E-002 | 6.46E-001 | 7.54E-001 | 1.93E-001 | 3.82E-001 | 3.20E-001 | 1.00E+000 |
| 1240 | 12 | 3 | *Yersinia enterocolitica wap bl6 Helicobacter pylori kx2 npgec Burkholderia pseudomallei Yersinia enterocolitica p60 bl6 Pseudomonas aeruginosa fdr1234 Lactobacillus acidophilus Bifidobacterium bifidum Aeromonas cavia Helicobacter pylori kx1 npgec Aggregatibacter actinomycetemcomitans Ehrlichia chaffeensis arkansa Pseudomonas aeruginosa fdr1* | 1.53E-001 | 0 | 1.00E+000 | 9.57E-001 | 1.53E-001 | 9.03E-001 | 2.46E-001 | 5.15E-001 | 1.92E-001 | 7.54E-001 | 4.34E-001 | 9.03E-001 | 7.55E-001 | 8.10E-001 |
| 578 | 7 | 13 | *Yersinia enterocolitica wap bl6 Helicobacter pylori kx2 npgec Ehrlichia chaffeensis arkansa Burkholderia pseudomallei Yersinia enterocolitica p60 bl6 Pseudomonas aeruginosa fdr1 Helicobacter pylori kx1 npgec* | 1.58E-001 | 9 | 1.37E-008 | 9.53E-001 | 2.05E-001 | 1.00E+000 | 7.55E-002 | 5.46E-001 | 1.00E+000 | 3.12E-001 | 1.92E-001 | 9.03E-001 | 1.00E+000 | 4.72E-001 |
| 572 | 7 | 13 | *Helicobacter pylori kx2 npgec Ehrlichia chaffeensis arkansa Burkholderia pseudomallei Staphylococcus aureus Porphyromonas gingivalis Helicobacter pylori kx1 npgec Ehrlichia chaffeensis liberty* | 1.58E-001 | 11 | 1.77E-007 | 7.95E-001 | 5.00E-001 | 9.03E-001 | 3.40E-001 | 2.36E-001 | 1.00E+000 | 9.25E-001 | 8.03E-001 | 9.03E-001 | 5.32E-001 | 1.55E-001 |
| 574 | 7 | 13 | *Yersinia enterocolitica wap bl6 Lactobacillus acidophilus Bifidobacterium bifidum Porphyromonas gingivalis Mycobacterium tuberculosis Helicobacter pylori kx1 npgec Escherichia coli* | 1.58E-001 | 0 | 1.00E+000 | 7.95E-001 | 5.00E-001 | 6.02E-001 | 9.12E-001 | 8.39E-001 | 6.71E-002 | 3.12E-001 | 4.88E-001 | 9.03E-001 | 5.32E-001 | 1.00E+000 |
| 576 | 7 | 13 | *Yersinia enterocolitica wap bl6 Pseudomonas aeruginosa Porphyromonas gingivalis Mycobacterium tuberculosis Helicobacter pylori kx1 npgec Escherichia coli Aggregatibacter actinomycetemcomitans* | 1.58E-001 | 1 | 3.07E-001 | 2.05E-001 | 9.53E-001 | 1.00E+000 | 3.40E-001 | 5.46E-001 | 1.00E+000 | 3.12E-001 | 4.88E-001 | 6.02E-001 | 1.23E-001 | 1.00E+000 |
| 570 | 7 | 13 | *Yersinia enterocolitica wap bl6 Helicobacter pylori kx2 npgec Lactobacillus acidophilus Pseudomonas aeruginosa Aeromonas cavia streptococcus pneumoniae d39 Helicobacter pylori kx1 npgec* | 1.58E-001 | 2 | 3.17E-001 | 9.53E-001 | 2.05E-001 | 6.02E-001 | 6.88E-001 | 5.46E-001 | 4.30E-001 | 9.25E-001 | 1.92E-001 | 6.02E-001 | 1.00E+000 | 1.00E+000 |
| 834 | 10 | 6 | *Yersinia enterocolitica wap bl6 Helicobacter pylori kx2 npgec Staphylococcus aureus Pseudomonas aeruginosa Porphyromonas gingivalis Aeromonas cavia streptococcus pneumoniae d39 Helicobacter pylori kx1 npgec Aggregatibacter actinomycetemcomitans Helicobacter pylori kx2 mgep* | 1.65E-002 | 0 | 1.00E+000 | 6.41E-001 | 6.41E-001 | 8.19E-001 | 4.00E-001 | 2.33E-002 | 1.00E+000 | 9.80E-001 | 2.39E-001 | 5.24E-001 | 2.36E-001 | 1.00E+000 |
| 526 | 9 | 11 | *Helicobacter pylori kx2 npgec Staphylococcus aureus Pseudomonas aeruginosa Aeromonas cavia Helicobacter pylori kx1 npgec Pseudomonas aeruginosa fdr440 Mycobacterium tuberculosis Helicobacter pylori kx2 mgep Escherichia coli* | 1.70E-003 | 0 | 1.00E+000 | 5.00E-001 | 7.73E-001 | 9.55E-001 | 4.91E-001 | 2.02E-001 | 1.00E+000 | 8.10E-001 | 1.56E-001 | 1.63E-001 | 1.00E+000 | 1.00E+000 |
| 522 | 9 | 11 | *Yersinia enterocolitica wap bl6 Yersinia enterocolitica wap bc Burkholderia pseudomallei Yersinia enterocolitica p60 bl6 Yersinia enterocolitica p60 bc Staphylococcus aureus Porphyromonas gingivalis Aeromonas cavia Mycobacterium tuberculosis* | 1.70E-003 | 0 | 1.00E+000 | 7.73E-001 | 5.00E-001 | 9.55E-001 | 4.91E-001 | 9.37E-001 | 1.00E+000 | 9.22E-003 | 1.56E-001 | 7.60E-001 | 6.34E-001 | 9.14E-001 |
| 888 | 8 | 7 | *Helicobacter pylori kx2 npgec Yersinia enterocolitica p60 bc Lactobacillus acidophilus Bifidobacterium bifidum Pseudomonas aeruginosa Helicobacter pylori kx2 mgep Aeromonas cavia Helicobacter pylori kx1 npgec* | 1.71E-001 | 0 | 1.00E+000 | 9.98E-001 | 2.24E-002 | 6.88E-001 | 5.89E-001 | 6.64E-001 | 8.78E-002 | 9.50E-001 | 9.08E-002 | 9.33E-001 | 1.00E+000 | 1.00E+000 |
| 890 | 8 | 7 | *Helicobacter pylori kx2 npgec Burkholderia pseudomallei Staphylococcus aureus Pseudomonas aeruginosa Aeromonas cavia streptococcus pneumoniae d39 Helicobacter pylori kx1 npgec Ehrlichia chaffeensis wakulla* | 1.71E-001 | 4 | 1.17E-002 | 6.52E-001 | 6.52E-001 | 6.88E-001 | 5.89E-001 | 3.51E-001 | 1.00E+000 | 9.50E-001 | 6.05E-001 | 3.45E-001 | 1.00E+000 | 5.57E-001 |
| 910 | 8 | 7 | *Helicobacter pylori kx2 npgec Lactobacillus acidophilus Pseudomonas aeruginosa fdr440 Porphyromonas gingivalis Mycobacterium tuberculosis Helicobacter pylori kx2 mgep Helicobacter pylori kx1 npgec Aggregatibacter actinomycetemcomitans* | 1.71E-001 | 0 | 1.00E+000 | 6.52E-001 | 6.52E-001 | 9.33E-001 | 5.89E-001 | 2.47E-002 | 4.79E-001 | 9.50E-001 | 6.05E-001 | 6.88E-001 | 1.58E-001 | 1.00E+000 |
| 892 | 8 | 7 | *Helicobacter pylori kx2 npgec Yersinia enterocolitica p60 bc Pseudomonas aeruginosa fdr440 Porphyromonas gingivalis Mycobacterium tuberculosis streptococcus pneumoniae d39 Helicobacter pylori kx1 npgec Escherichia coli* | 1.71E-001 | 1 | 5.19E-001 | 3.48E-001 | 8.81E-001 | 9.33E-001 | 5.89E-001 | 1.21E-001 | 1.00E+000 | 4.11E-001 | 2.94E-001 | 3.45E-001 | 5.86E-001 | 1.00E+000 |
| 22 | 4 | 175 | *Lactobacillus acidophilus Staphylococcus aureus Bifidobacterium bifidum Helicobacter pylori kx1 npgec* | 1.73E-001 | 81 | < 1.67E-026 | 9.47E-001 | 3.03E-001 | 4.86E-002 | 9.96E-001 | 4.95E-001 | 2.02E-002 | 1.00E+000 | 8.43E-001 | 7.19E-001 | 1.00E+000 | 1.00E+000 |
| 274 | 6 | 28 | *Yersinia enterocolitica wap bc Lactobacillus acidophilus Bifidobacterium bifidum Porphyromonas gingivalis Helicobacter pylori kx1 npgec Aggregatibacter actinomycetemcomitans* | 1.75E-001 | 7 | 3.87E-008 | 9.08E-001 | 3.31E-001 | 5.03E-001 | 7.84E-001 | 4.13E-001 | 4.88E-002 | 8.87E-001 | 7.13E-001 | 1.00E+000 | 9.10E-002 | 1.00E+000 |
| 26 | 4 | 165 | *Staphylococcus aureus Porphyromonas gingivalis Aeromonas cavia Aggregatibacter actinomycetemcomitans* | 1.77E-001 | 47 | 7.80E-006 | 3.03E-001 | 9.47E-001 | 7.19E-001 | 6.80E-001 | 1.46E-001 | 1.00E+000 | 1.00E+000 | 8.43E-001 | 7.19E-001 | 3.91E-002 | 1.00E+000 |
| 234 | 7 | 28 | *Helicobacter pylori kx2 npgec Burkholderia pseudomallei Lactobacillus acidophilus Mycobacterium tuberculosis Helicobacter pylori kx1 npgec Escherichia coli Aggregatibacter actinomycetemcomitans* | 1.78E-002 | 0 | 1.00E+000 | 5.00E-001 | 7.95E-001 | 9.03E-001 | 6.88E-001 | 5.46E-001 | 4.30E-001 | 3.12E-001 | 4.88E-001 | 9.03E-001 | 5.32E-001 | 8.42E-001 |
| 232 | 7 | 28 | *Yersinia enterocolitica wap bc Helicobacter pylori kx2 npgec Yersinia enterocolitica p60 bl6 Yersinia enterocolitica p60 bc Staphylococcus aureus Porphyromonas gingivalis Aeromonas cavia* | 1.78E-002 | 0 | 1.00E+000 | 9.53E-001 | 2.05E-001 | 9.03E-001 | 3.40E-001 | 5.46E-001 | 1.00E+000 | 3.12E-001 | 4.43E-002 | 9.03E-001 | 5.32E-001 | 1.00E+000 |
| 628 | 7 | 12 | *Burkholderia pseudomallei Pseudomonas aeruginosa fdr440 Staphylococcus aureus Porphyromonas gingivalis Mycobacterium tuberculosis Helicobacter pylori kx1 npgec Ehrlichia chaffeensis liberty* | 1.85E-001 | 0 | 1.00E+000 | 2.05E-001 | 9.53E-001 | 9.03E-001 | 6.88E-001 | 2.36E-001 | 1.00E+000 | 6.60E-001 | 9.67E-001 | 2.56E-001 | 5.32E-001 | 4.72E-001 |
| 630 | 7 | 12 | *Helicobacter pylori kx2 npgec Pseudomonas aeruginosa Porphyromonas gingivalis Mycobacterium tuberculosis streptococcus pneumoniae d39 Helicobacter pylori kx1 npgec Ehrlichia chaffeensis liberty* | 1.85E-001 | 1 | 5.19E-001 | 5.00E-001 | 7.95E-001 | 9.03E-001 | 6.88E-001 | 2.36E-001 | 1.00E+000 | 9.25E-001 | 8.03E-001 | 2.56E-001 | 5.32E-001 | 8.42E-001 |
| 278 | 6 | 27 | *Yersinia enterocolitica wap bl6 Helicobacter pylori kx2 npgec Porphyromonas gingivalis Helicobacter pylori kx2 mgep Aeromonas cavia Helicobacter pylori kx1 npgec* | 1.87E-001 | 14 | 1.98E-003 | 9.90E-001 | 9.18E-002 | 1.00E+000 | 1.13E-001 | 1.36E-001 | 1.00E+000 | 8.87E-001 | 1.64E-002 | 1.00E+000 | 4.74E-001 | 1.00E+000 |
| 956 | 13 | 4 | *Yersinia enterocolitica wap bc Burkholderia pseudomallei Yersinia enterocolitica p60 bl6 Yersinia enterocolitica p60 bc Lactobacillus acidophilus Anaplasma phagocytophilum Staphylococcus aureus Bifidobacterium bifidum Porphyromonas gingivalis Aeromonas cavia Aggregatibacter actinomycetemcomitans Ehrlichia chaffeensis wakulla Mycobacterium tuberculosis* | 1.89E-003 | 0 | 1.00E+000 | 7.47E-001 | 5.00E-001 | 7.49E-001 | 7.23E-001 | 9.57E-001 | 2.22E-001 | 2.77E-001 | 7.85E-001 | 9.31E-001 | 3.63E-001 | 5.79E-001 |
| 746 | 9 | 8 | *Yersinia enterocolitica wap bl6 Burkholderia pseudomallei Yersinia enterocolitica p60 bl6 Yersinia enterocolitica p60 bc Pseudomonas aeruginosa fdr1234 Lactobacillus acidophilus Bifidobacterium bifidum Aeromonas cavia Pseudomonas aeruginosa fdr1* | 1.90E-002 | 0 | 1.00E+000 | 9.35E-001 | 2.27E-001 | 7.60E-001 | 4.91E-001 | 9.37E-001 | 1.11E-001 | 2.16E-001 | 4.04E-001 | 7.60E-001 | 1.00E+000 | 9.14E-001 |
| 84 | 6 | 61 | *Burkholderia pseudomallei Lactobacillus acidophilus Bifidobacterium bifidum Porphyromonas gingivalis Helicobacter pylori kx1 npgec Escherichia coli* | 1.91E-002 | 6 | 9.99E-002 | 6.69E-001 | 6.69E-001 | 5.03E-001 | 7.84E-001 | 7.55E-001 | 4.88E-002 | 5.61E-001 | 7.13E-001 | 1.00E+000 | 4.74E-001 | 7.89E-001 |
| 300 | 6 | 26 | *Helicobacter pylori kx2 npgec Yersinia enterocolitica p60 bl6 Yersinia enterocolitica p60 bc Mycobacterium tuberculosis Aeromonas cavia Helicobacter pylori kx1 npgec* | 1.99E-001 | 0 | 1.00E+000 | 9.90E-001 | 9.18E-002 | 1.00E+000 | 4.39E-001 | 7.55E-001 | 1.00E+000 | 2.16E-001 | 1.64E-002 | 8.60E-001 | 1.00E+000 | 1.00E+000 |
| 296 | 6 | 26 | *Yersinia enterocolitica wap bl6 Helicobacter pylori kx2 npgec Yersinia enterocolitica p60 bl6 Yersinia enterocolitica p60 bc streptococcus pneumoniae d39 Helicobacter pylori kx1 npgec* | 1.99E-001 | 20 | 1.28E-003 | 9.90E-001 | 9.18E-002 | 8.60E-001 | 4.39E-001 | 4.13E-001 | 1.00E+000 | 2.16E-001 | 1.64E-002 | 8.60E-001 | 1.00E+000 | 1.00E+000 |
| 294 | 6 | 26 | *Helicobacter pylori kx2 npgec Burkholderia pseudomallei Pseudomonas aeruginosa fdr440 Mycobacterium tuberculosis streptococcus pneumoniae d39 Helicobacter pylori kx1 npgec* | 1.99E-001 | 0 | 1.00E+000 | 3.31E-001 | 9.08E-001 | 8.60E-001 | 7.84E-001 | 1.36E-001 | 1.00E+000 | 5.61E-001 | 7.13E-001 | 1.73E-001 | 1.00E+000 | 7.89E-001 |
| 292 | 6 | 26 | *Helicobacter pylori kx2 npgec Burkholderia pseudomallei Pseudomonas aeruginosa Helicobacter pylori kx2 mgep Helicobacter pylori kx1 npgec Escherichia coli* | 1.99E-001 | 3 | 2.68E-002 | 6.69E-001 | 6.69E-001 | 1.00E+000 | 1.13E-001 | 4.13E-001 | 1.00E+000 | 5.61E-001 | 1.08E-001 | 8.60E-001 | 1.00E+000 | 7.89E-001 |
| 102 | 5 | 64 | *Helicobacter pylori kx2 npgec Bifidobacterium bifidum Pseudomonas aeruginosa streptococcus pneumoniae d39 Helicobacter pylori kx1 npgec* | 2.02E-001 | 8 | 3.50E-002 | 8.28E-001 | 5.00E-001 | 3.93E-001 | 8.69E-001 | 2.75E-001 | 3.23E-001 | 1.00E+000 | 5.96E-001 | 3.93E-001 | 1.00E+000 | 1.00E+000 |
| 104 | 5 | 64 | *Yersinia enterocolitica p60 bl6 Yersinia enterocolitica p60 bc Staphylococcus aureus Helicobacter pylori kx1 npgec Aggregatibacter actinomycetemcomitans* | 2.02E-001 | 33 | 1.01E-008 | 8.28E-001 | 5.00E-001 | 8.00E-001 | 5.54E-001 | 2.75E-001 | 1.00E+000 | 4.46E-001 | 2.35E-001 | 8.00E-001 | 4.10E-001 | 1.00E+000 |
| 1156 | 10 | 4 | *Yersinia enterocolitica wap bl6 Helicobacter pylori kx2 npgec Porphyromonas gingivalis Aeromonas cavia streptococcus pneumoniae d39 Helicobacter pylori kx1 npgec Ehrlichia chaffeensis liberty Aggregatibacter actinomycetemcomitans Pseudomonas aeruginosa fdr440 Escherichia coli* | 2.03E-001 | 0 | 1.00E+000 | 6.41E-001 | 6.41E-001 | 9.70E-001 | 1.37E-001 | 1.05E-001 | 1.00E+000 | 8.63E-001 | 2.39E-001 | 8.19E-001 | 2.36E-001 | 9.37E-001 |
| 1166 | 10 | 4 | *Yersinia enterocolitica wap bl6 Yersinia enterocolitica p60 bc Lactobacillus acidophilus Anaplasma phagocytophilum Bifidobacterium bifidum Porphyromonas gingivalis Aeromonas cavia Aggregatibacter actinomycetemcomitans Mycobacterium tuberculosis Escherichia coli* | 2.03E-001 | 0 | 1.00E+000 | 6.41E-001 | 6.41E-001 | 8.19E-001 | 7.04E-001 | 9.62E-001 | 1.36E-001 | 2.96E-001 | 5.13E-001 | 9.70E-001 | 2.36E-001 | 9.37E-001 |
| 1126 | 10 | 4 | *Yersinia enterocolitica wap bl6 Helicobacter pylori kx2 npgec Yersinia enterocolitica p60 bc Lactobacillus acidophilus Bifidobacterium bifidum Pseudomonas aeruginosa streptococcus pneumoniae d39 Helicobacter pylori kx1 npgec Aggregatibacter actinomycetemcomitans Helicobacter pylori kx2 mgep* | 2.03E-001 | 0 | 1.00E+000 | 9.67E-001 | 1.39E-001 | 5.24E-001 | 7.04E-001 | 3.00E-001 | 1.36E-001 | 8.63E-001 | 2.39E-001 | 8.19E-001 | 6.79E-001 | 1.00E+000 |
| 1170 | 10 | 4 | *Helicobacter pylori kx2 npgec Burkholderia pseudomallei Lactobacillus acidophilus Bifidobacterium bifidum Pseudomonas aeruginosa streptococcus pneumoniae d39 Helicobacter pylori kx1 npgec Aggregatibacter actinomycetemcomitans Pseudomonas aeruginosa fdr440 Escherichia coli* | 2.03E-001 | 0 | 1.00E+000 | 3.59E-001 | 8.61E-001 | 5.24E-001 | 7.04E-001 | 3.00E-001 | 1.36E-001 | 8.63E-001 | 7.88E-001 | 5.24E-001 | 6.79E-001 | 9.37E-001 |
| 34 | 4 | 152 | *Yersinia enterocolitica wap bl6 Yersinia enterocolitica p60 bc Lactobacillus acidophilus Bifidobacterium bifidum* | 2.08E-001 | 86 | < 1.67E-026 | 1.00E+000 | 5.35E-002 | 2.77E-001 | 9.37E-001 | 1.00E+000 | 2.02E-002 | 3.20E-001 | 4.51E-001 | 1.00E+000 | 1.00E+000 | 1.00E+000 |
| 116 | 5 | 62 | *Helicobacter pylori kx2 npgec Burkholderia pseudomallei streptococcus pneumoniae d39 Helicobacter pylori kx1 npgec Aggregatibacter actinomycetemcomitans* | 2.13E-001 | 2 | 2.49E-001 | 5.00E-001 | 8.28E-001 | 8.00E-001 | 5.54E-001 | 6.08E-002 | 1.00E+000 | 8.32E-001 | 5.96E-001 | 8.00E-001 | 4.10E-001 | 7.21E-001 |
| 330 | 6 | 25 | *Helicobacter pylori kx2 npgec Staphylococcus aureus Bifidobacterium bifidum Mycobacterium tuberculosis Aeromonas cavia Aggregatibacter actinomycetemcomitans* | 2.13E-001 | 0 | 1.00E+000 | 6.69E-001 | 6.69E-001 | 5.03E-001 | 9.54E-001 | 4.13E-001 | 3.78E-001 | 8.87E-001 | 7.13E-001 | 5.03E-001 | 4.74E-001 | 1.00E+000 |
| 322 | 6 | 25 | *Helicobacter pylori kx2 npgec Yersinia enterocolitica p60 bc Porphyromonas gingivalis streptococcus pneumoniae d39 Helicobacter pylori kx1 npgec Aggregatibacter actinomycetemcomitans* | 2.13E-001 | 6 | 1.83E-001 | 6.69E-001 | 6.69E-001 | 8.60E-001 | 4.39E-001 | 2.32E-002 | 1.00E+000 | 8.87E-001 | 3.61E-001 | 8.60E-001 | 9.10E-002 | 1.00E+000 |
| 320 | 6 | 25 | *Yersinia enterocolitica wap bl6 Burkholderia pseudomallei Yersinia enterocolitica p60 bl6 Yersinia enterocolitica p60 bc Pseudomonas aeruginosa fdr1 Porphyromonas gingivalis* | 2.13E-001 | 14 | 3.77E-007 | 6.69E-001 | 6.69E-001 | 1.00E+000 | 1.13E-001 | 7.55E-001 | 1.00E+000 | 4.58E-002 | 3.61E-001 | 8.60E-001 | 4.74E-001 | 7.89E-001 |
| 1072 | 9 | 5 | *Yersinia enterocolitica wap bl6 Helicobacter pylori kx2 npgec Yersinia enterocolitica p60 bl6 Lactobacillus acidophilus Bifidobacterium bifidum Porphyromonas gingivalis Helicobacter pylori kx1 npgec Ehrlichia chaffeensis arkansa Ehrlichia chaffeensis wakulla* | 2.13E-001 | 0 | 1.00E+000 | 9.99E-001 | 1.02E-002 | 7.60E-001 | 4.91E-001 | 7.60E-001 | 1.11E-001 | 8.10E-001 | 4.04E-001 | 1.00E+000 | 6.34E-001 | 6.33E-001 |
| 1060 | 9 | 5 | *Yersinia enterocolitica wap bc Helicobacter pylori kx2 npgec Yersinia enterocolitica p60 bl6 Yersinia enterocolitica p60 bc Staphylococcus aureus Porphyromonas gingivalis Aeromonas cavia streptococcus pneumoniae d39 Helicobacter pylori kx1 npgec* | 2.13E-001 | 0 | 1.00E+000 | 9.35E-001 | 2.27E-001 | 7.60E-001 | 4.91E-001 | 2.02E-001 | 1.00E+000 | 5.09E-001 | 3.83E-002 | 7.60E-001 | 6.34E-001 | 1.00E+000 |
| 1070 | 9 | 5 | *Helicobacter pylori kx2 npgec Lactobacillus acidophilus Staphylococcus aureus Bifidobacterium bifidum Pseudomonas aeruginosa Helicobacter pylori kx1 npgec Ehrlichia chaffeensis arkansa Ehrlichia chaffeensis wakulla Helicobacter pylori kx2 mgep* | 2.13E-001 | 0 | 1.00E+000 | 9.90E-001 | 6.50E-002 | 4.36E-001 | 7.84E-001 | 4.71E-001 | 1.11E-001 | 1.00E+000 | 7.06E-001 | 7.60E-001 | 1.00E+000 | 6.33E-001 |
| 690 | 7 | 11 | *Pseudomonas aeruginosa fdr440 Mycobacterium tuberculosis Aeromonas cavia Helicobacter pylori kx1 npgec Escherichia coli Ehrlichia chaffeensis liberty Aggregatibacter actinomycetemcomitans* | 2.17E-001 | 0 | 1.00E+000 | 5.00E-001 | 7.95E-001 | 1.00E+000 | 3.40E-001 | 5.46E-001 | 1.00E+000 | 6.60E-001 | 4.88E-001 | 6.02E-001 | 5.32E-001 | 8.42E-001 |
| 694 | 7 | 11 | *Helicobacter pylori kx2 npgec Ehrlichia chaffeensis arkansa Lactobacillus acidophilus Bifidobacterium bifidum Helicobacter pylori kx2 mgep Aeromonas cavia Helicobacter pylori kx1 npgec* | 2.17E-001 | 0 | 1.00E+000 | 1.00E+000 | 4.31E-003 | 6.02E-001 | 6.88E-001 | 5.46E-001 | 6.71E-002 | 1.00E+000 | 1.92E-001 | 1.00E+000 | 1.00E+000 | 8.42E-001 |
| 692 | 7 | 11 | *Helicobacter pylori kx2 npgec Burkholderia pseudomallei Pseudomonas aeruginosa fdr440 Aeromonas cavia streptococcus pneumoniae d39 Helicobacter pylori kx1 npgec Aggregatibacter actinomycetemcomitans* | 2.17E-001 | 0 | 1.00E+000 | 5.00E-001 | 7.95E-001 | 9.03E-001 | 3.40E-001 | 6.08E-002 | 1.00E+000 | 9.25E-001 | 4.88E-001 | 6.02E-001 | 5.32E-001 | 8.42E-001 |
| 688 | 7 | 11 | *Helicobacter pylori kx2 npgec Burkholderia pseudomallei Ehrlichia chaffeensis wakulla Lactobacillus acidophilus Aeromonas cavia Helicobacter pylori kx1 npgec Aggregatibacter actinomycetemcomitans* | 2.17E-001 | 0 | 1.00E+000 | 9.53E-001 | 2.05E-001 | 9.03E-001 | 3.40E-001 | 5.46E-001 | 4.30E-001 | 9.25E-001 | 4.88E-001 | 1.00E+000 | 5.32E-001 | 4.72E-001 |
| 90 | 6 | 58 | *Helicobacter pylori kx2 npgec Burkholderia pseudomallei Lactobacillus acidophilus Pseudomonas aeruginosa Porphyromonas gingivalis Helicobacter pylori kx1 npgec* | 2.26E-002 | 5 | 1.29E-002 | 6.69E-001 | 6.69E-001 | 8.60E-001 | 4.39E-001 | 4.13E-001 | 3.78E-001 | 8.87E-001 | 7.13E-001 | 8.60E-001 | 4.74E-001 | 7.89E-001 |
| 92 | 6 | 58 | *Helicobacter pylori kx2 npgec Porphyromonas gingivalis Mycobacterium tuberculosis Aeromonas cavia Helicobacter pylori kx1 npgec Aggregatibacter actinomycetemcomitans* | 2.26E-002 | 3 | 6.01E-001 | 6.69E-001 | 6.69E-001 | 1.00E+000 | 4.39E-001 | 1.36E-001 | 1.00E+000 | 8.87E-001 | 3.61E-001 | 8.60E-001 | 9.10E-002 | 1.00E+000 |
| 352 | 6 | 24 | *Burkholderia pseudomallei Lactobacillus acidophilus Pseudomonas aeruginosa fdr440 Pseudomonas aeruginosa Mycobacterium tuberculosis Escherichia coli* | 2.29E-001 | 1 | 6.96E-001 | 9.18E-002 | 9.90E-001 | 8.60E-001 | 7.84E-001 | 9.56E-001 | 3.78E-001 | 2.16E-001 | 9.44E-001 | 1.73E-001 | 1.00E+000 | 7.89E-001 |
| 354 | 6 | 24 | *Helicobacter pylori kx2 npgec Pseudomonas aeruginosa fdr1 Porphyromonas gingivalis Mycobacterium tuberculosis Helicobacter pylori kx1 npgec Escherichia coli* | 2.29E-001 | 1 | 8.99E-001 | 3.31E-001 | 9.08E-001 | 1.00E+000 | 4.39E-001 | 1.36E-001 | 1.00E+000 | 5.61E-001 | 3.61E-001 | 5.03E-001 | 4.74E-001 | 1.00E+000 |
| 356 | 6 | 24 | *Helicobacter pylori kx2 npgec Ehrlichia chaffeensis arkansa Ehrlichia chaffeensis wakulla Aeromonas cavia Helicobacter pylori kx1 npgec Ehrlichia chaffeensis liberty* | 2.29E-001 | 9 | 6.88E-006 | 1.00E+000 | 1.03E-002 | 1.00E+000 | 1.13E-001 | 7.55E-001 | 1.00E+000 | 1.00E+000 | 3.61E-001 | 1.00E+000 | 1.00E+000 | 1.01E-001 |
| 130 | 5 | 59 | *Helicobacter pylori kx2 npgec Lactobacillus acidophilus Staphylococcus aureus Mycobacterium tuberculosis Helicobacter pylori kx1 npgec* | 2.32E-001 | 0 | 1.00E+000 | 8.28E-001 | 5.00E-001 | 3.93E-001 | 9.82E-001 | 2.75E-001 | 3.23E-001 | 8.32E-001 | 5.96E-001 | 3.93E-001 | 1.00E+000 | 1.00E+000 |
| 370 | 6 | 23 | *Helicobacter pylori kx2 npgec Burkholderia pseudomallei Porphyromonas gingivalis Mycobacterium tuberculosis Aeromonas cavia streptococcus pneumoniae d39* | 2.44E-001 | 0 | 1.00E+000 | 3.31E-001 | 9.08E-001 | 8.60E-001 | 7.84E-001 | 4.13E-001 | 1.00E+000 | 5.61E-001 | 7.13E-001 | 5.03E-001 | 4.74E-001 | 7.89E-001 |
| 368 | 6 | 23 | *Helicobacter pylori kx2 npgec Lactobacillus acidophilus Porphyromonas gingivalis Mycobacterium tuberculosis streptococcus pneumoniae d39 Escherichia coli* | 2.44E-001 | 0 | 1.00E+000 | 3.31E-001 | 9.08E-001 | 5.03E-001 | 9.54E-001 | 4.13E-001 | 3.78E-001 | 5.61E-001 | 7.13E-001 | 5.03E-001 | 4.74E-001 | 1.00E+000 |
| 136 | 5 | 56 | *Burkholderia pseudomallei Lactobacillus acidophilus Bifidobacterium bifidum Helicobacter pylori kx1 npgec Ehrlichia chaffeensis liberty* | 2.55E-001 | 28 | 8.58E-011 | 9.76E-001 | 1.72E-001 | 3.93E-001 | 8.69E-001 | 9.23E-001 | 3.31E-002 | 8.32E-001 | 9.05E-001 | 1.00E+000 | 1.00E+000 | 2.88E-001 |
| 138 | 5 | 56 | *Helicobacter pylori kx2 npgec Burkholderia pseudomallei Aeromonas cavia Helicobacter pylori kx1 npgec Escherichia coli* | 2.55E-001 | 14 | 1.00E-005 | 8.28E-001 | 5.00E-001 | 1.00E+000 | 1.68E-001 | 6.41E-001 | 1.00E+000 | 4.46E-001 | 4.69E-002 | 1.00E+000 | 1.00E+000 | 7.21E-001 |
| 750 | 7 | 10 | *Helicobacter pylori kx2 npgec Burkholderia pseudomallei Porphyromonas gingivalis Helicobacter pylori kx2 mgep Aeromonas cavia Helicobacter pylori kx1 npgec Ehrlichia chaffeensis liberty* | 2.56E-001 | 5 | 3.60E-003 | 9.53E-001 | 2.05E-001 | 1.00E+000 | 7.55E-002 | 2.36E-001 | 1.00E+000 | 9.25E-001 | 1.92E-001 | 1.00E+000 | 5.32E-001 | 4.72E-001 |
| 382 | 6 | 22 | *Helicobacter pylori kx2 npgec Lactobacillus acidophilus Pseudomonas aeruginosa fdr440 Staphylococcus aureus Pseudomonas aeruginosa Helicobacter pylori kx1 npgec* | 2.60E-001 | 0 | 1.00E+000 | 6.69E-001 | 6.69E-001 | 5.03E-001 | 7.84E-001 | 1.36E-001 | 3.78E-001 | 1.00E+000 | 7.13E-001 | 1.73E-001 | 1.00E+000 | 1.00E+000 |
| 552 | 8 | 12 | *Yersinia enterocolitica wap bl6 Yersinia enterocolitica p60 bl6 Yersinia enterocolitica p60 bc Porphyromonas gingivalis Mycobacterium tuberculosis Aeromonas cavia Escherichia coli Aggregatibacter actinomycetemcomitans* | 2.72E-002 | 0 | 1.00E+000 | 6.52E-001 | 6.52E-001 | 1.00E+000 | 2.57E-001 | 8.98E-001 | 1.00E+000 | 3.09E-002 | 9.08E-002 | 9.33E-001 | 1.58E-001 | 1.00E+000 |
| 550 | 8 | 12 | *Burkholderia pseudomallei Lactobacillus acidophilus Staphylococcus aureus Pseudomonas aeruginosa Porphyromonas gingivalis Mycobacterium tuberculosis Helicobacter pylori kx1 npgec Aggregatibacter actinomycetemcomitans* | 2.72E-002 | 0 | 1.00E+000 | 1.19E-001 | 9.78E-001 | 6.88E-001 | 8.54E-001 | 3.51E-001 | 4.79E-001 | 7.43E-001 | 9.81E-001 | 3.45E-001 | 1.58E-001 | 8.82E-001 |
| 262 | 7 | 25 | *Helicobacter pylori kx2 npgec Pseudomonas aeruginosa fdr1 Staphylococcus aureus Porphyromonas gingivalis Aeromonas cavia Helicobacter pylori kx1 npgec Aggregatibacter actinomycetemcomitans* | 2.73E-002 | 9 | 6.88E-006 | 5.00E-001 | 7.95E-001 | 9.03E-001 | 3.40E-001 | 8.14E-003 | 1.00E+000 | 1.00E+000 | 4.88E-001 | 6.02E-001 | 1.23E-001 | 1.00E+000 |
| 258 | 7 | 25 | *Yersinia enterocolitica wap bl6 Helicobacter pylori kx2 npgec Yersinia enterocolitica p60 bl6 Lactobacillus acidophilus Bifidobacterium bifidum Pseudomonas aeruginosa Helicobacter pylori kx1 npgec* | 2.73E-002 | 0 | 1.00E+000 | 9.96E-001 | 4.65E-002 | 6.02E-001 | 6.88E-001 | 8.39E-001 | 6.71E-002 | 6.60E-001 | 1.92E-001 | 9.03E-001 | 1.00E+000 | 1.00E+000 |
| 150 | 5 | 53 | *Helicobacter pylori kx2 npgec Mycobacterium tuberculosis Helicobacter pylori kx2 mgep Helicobacter pylori kx1 npgec Escherichia coli* | 2.74E-001 | 18 | 3.38E-004 | 8.28E-001 | 5.00E-001 | 1.00E+000 | 5.54E-001 | 2.75E-001 | 1.00E+000 | 4.46E-001 | 4.69E-002 | 8.00E-001 | 1.00E+000 | 1.00E+000 |
| 400 | 6 | 21 | *Helicobacter pylori kx2 npgec Lactobacillus acidophilus Pseudomonas aeruginosa streptococcus pneumoniae d39 Helicobacter pylori kx1 npgec Ehrlichia chaffeensis liberty* | 2.78E-001 | 10 | 6.10E-004 | 9.08E-001 | 3.31E-001 | 5.03E-001 | 7.84E-001 | 4.13E-001 | 3.78E-001 | 1.00E+000 | 7.13E-001 | 5.03E-001 | 1.00E+000 | 7.89E-001 |
| 402 | 6 | 21 | *Helicobacter pylori kx2 npgec Yersinia enterocolitica p60 bc Staphylococcus aureus Pseudomonas aeruginosa Porphyromonas gingivalis Aeromonas cavia* | 2.78E-001 | 2 | 2.49E-001 | 6.69E-001 | 6.69E-001 | 8.60E-001 | 4.39E-001 | 4.13E-001 | 1.00E+000 | 8.87E-001 | 3.61E-001 | 5.03E-001 | 4.74E-001 | 1.00E+000 |
| 404 | 6 | 21 | *Helicobacter pylori kx2 npgec Burkholderia pseudomallei Lactobacillus acidophilus Pseudomonas aeruginosa fdr440 Helicobacter pylori kx2 mgep Helicobacter pylori kx1 npgec* | 2.78E-001 | 0 | 1.00E+000 | 9.08E-001 | 3.31E-001 | 8.60E-001 | 4.39E-001 | 1.36E-001 | 3.78E-001 | 8.87E-001 | 3.61E-001 | 8.60E-001 | 1.00E+000 | 7.89E-001 |
| 406 | 6 | 21 | *Helicobacter pylori kx2 npgec streptococcus gordonii Staphylococcus aureus streptococcus pneumoniae d39 Helicobacter pylori kx1 npgec Aggregatibacter actinomycetemcomitans* | 2.78E-001 | 0 | 1.00E+000 | 3.31E-001 | 9.08E-001 | 1.73E-001 | 9.54E-001 | 1.53E-003 | 1.00E+000 | 1.00E+000 | 7.13E-001 | 5.03E-001 | 9.10E-002 | 1.00E+000 |
| 1050 | 8 | 6 | *Yersinia enterocolitica wap bl6 Yersinia enterocolitica p60 bl6 Lactobacillus acidophilus Bifidobacterium bifidum Porphyromonas gingivalis Aeromonas cavia Helicobacter pylori kx1 npgec Ehrlichia chaffeensis liberty* | 2.81E-001 | 0 | 1.00E+000 | 9.98E-001 | 2.24E-002 | 6.88E-001 | 5.89E-001 | 8.98E-001 | 8.78E-002 | 7.43E-001 | 2.94E-001 | 1.00E+000 | 5.86E-001 | 8.82E-001 |
| 1032 | 8 | 6 | *Helicobacter pylori kx2 npgec Burkholderia pseudomallei Lactobacillus acidophilus Bifidobacterium bifidum Aeromonas cavia streptococcus pneumoniae d39 Helicobacter pylori kx1 npgec Helicobacter pylori kx2 mgep* | 2.81E-001 | 2 | 1.83E-001 | 9.78E-001 | 1.19E-001 | 3.45E-001 | 8.54E-001 | 3.51E-001 | 8.78E-002 | 9.50E-001 | 2.94E-001 | 9.33E-001 | 1.00E+000 | 8.82E-001 |
| 1038 | 8 | 6 | *Yersinia enterocolitica wap bl6 Helicobacter pylori kx2 npgec Burkholderia pseudomallei Lactobacillus acidophilus Bifidobacterium bifidum Fusobacterium nucleatum streptococcus pneumoniae d39 streptococcus gordonii* | 2.81E-001 | 0 | 1.00E+000 | 6.52E-001 | 6.52E-001 | 1.07E-001 | 9.69E-001 | 3.51E-001 | 8.78E-002 | 7.43E-001 | 8.68E-001 | 9.33E-001 | 1.58E-001 | 8.82E-001 |
| 1028 | 8 | 6 | *Helicobacter pylori kx2 npgec Lactobacillus acidophilus Staphylococcus aureus Bifidobacterium bifidum streptococcus pneumoniae d39 Helicobacter pylori kx1 npgec Ehrlichia chaffeensis liberty Helicobacter pylori kx2 mgep* | 2.81E-001 | 1 | 4.73E-001 | 9.78E-001 | 1.19E-001 | 1.07E-001 | 9.69E-001 | 1.21E-001 | 8.78E-002 | 1.00E+000 | 6.05E-001 | 6.88E-001 | 1.00E+000 | 8.82E-001 |
| 1044 | 8 | 6 | *Yersinia enterocolitica wap bl6 Helicobacter pylori kx2 npgec Porphyromonas gingivalis Aeromonas cavia Helicobacter pylori kx1 npgec Aggregatibacter actinomycetemcomitans streptococcus gordonii Escherichia coli* | 2.81E-001 | 0 | 1.00E+000 | 6.52E-001 | 6.52E-001 | 9.33E-001 | 2.57E-001 | 1.21E-001 | 1.00E+000 | 7.43E-001 | 9.08E-002 | 1.00E+000 | 1.76E-002 | 1.00E+000 |
| 1046 | 8 | 6 | *Yersinia enterocolitica wap bl6 Burkholderia pseudomallei Staphylococcus aureus Pseudomonas aeruginosa Porphyromonas gingivalis Aeromonas cavia streptococcus pneumoniae d39 Mycobacterium tuberculosis* | 2.81E-001 | 0 | 1.00E+000 | 1.19E-001 | 9.78E-001 | 6.88E-001 | 8.54E-001 | 6.64E-001 | 1.00E+000 | 4.11E-001 | 8.68E-001 | 1.07E-001 | 5.86E-001 | 8.82E-001 |
| 44 | 2 | 260 | *Burkholderia pseudomallei Escherichia coli* | 2.93E-001 | 87 | 3.17E-005 | 2.44E-001 | 1.00E+000 | 1.00E+000 | 5.05E-001 | 1.00E+000 | 1.00E+000 | 7.67E-002 | 5.92E-001 | 1.00E+000 | 1.00E+000 | 3.87E-001 |
| 432 | 6 | 20 | *Helicobacter pylori kx2 npgec Bifidobacterium bifidum Porphyromonas gingivalis Mycobacterium tuberculosis Helicobacter pylori kx2 mgep Helicobacter pylori kx1 npgec* | 2.98E-001 | 5 | 1.29E-002 | 9.08E-001 | 3.31E-001 | 8.60E-001 | 7.84E-001 | 1.36E-001 | 3.78E-001 | 8.87E-001 | 3.61E-001 | 8.60E-001 | 4.74E-001 | 1.00E+000 |
| 426 | 6 | 20 | *Helicobacter pylori kx2 npgec Lactobacillus acidophilus Helicobacter pylori kx2 mgep Aeromonas cavia Helicobacter pylori kx1 npgec Escherichia coli* | 2.98E-001 | 9 | 7.71E-004 | 9.90E-001 | 9.18E-002 | 8.60E-001 | 4.39E-001 | 4.13E-001 | 3.78E-001 | 8.87E-001 | 1.64E-002 | 1.00E+000 | 1.00E+000 | 1.00E+000 |
| 428 | 6 | 20 | *Yersinia enterocolitica wap bl6 Burkholderia pseudomallei Yersinia enterocolitica p60 bc Porphyromonas gingivalis Aeromonas cavia streptococcus pneumoniae d39* | 2.98E-001 | 10 | 6.57E-003 | 6.69E-001 | 6.69E-001 | 8.60E-001 | 4.39E-001 | 7.55E-001 | 1.00E+000 | 2.16E-001 | 3.61E-001 | 8.60E-001 | 4.74E-001 | 7.89E-001 |
| 440 | 6 | 20 | *Yersinia enterocolitica wap bl6 Porphyromonas gingivalis Mycobacterium tuberculosis Aeromonas cavia streptococcus pneumoniae d39 Aggregatibacter actinomycetemcomitans* | 2.98E-001 | 0 | 1.00E+000 | 3.31E-001 | 9.08E-001 | 8.60E-001 | 7.84E-001 | 4.13E-001 | 1.00E+000 | 5.61E-001 | 7.13E-001 | 5.03E-001 | 9.10E-002 | 1.00E+000 |
| 816 | 7 | 9 | *Yersinia enterocolitica wap bl6 Helicobacter pylori kx2 npgec Yersinia enterocolitica p60 bl6 Bifidobacterium bifidum Helicobacter pylori kx1 npgec Escherichia coli Ehrlichia chaffeensis liberty* | 3.03E-001 | 0 | 1.00E+000 | 9.96E-001 | 4.65E-002 | 9.03E-001 | 3.40E-001 | 8.39E-001 | 4.30E-001 | 3.12E-001 | 4.43E-002 | 1.00E+000 | 1.00E+000 | 8.42E-001 |
| 818 | 7 | 9 | *Helicobacter pylori kx2 npgec Yersinia enterocolitica p60 bc Ehrlichia chaffeensis wakulla Lactobacillus acidophilus Staphylococcus aureus Helicobacter pylori kx1 npgec Ehrlichia chaffeensis liberty* | 3.03E-001 | 10 | 6.18E-006 | 9.96E-001 | 4.65E-002 | 6.02E-001 | 6.88E-001 | 5.46E-001 | 4.30E-001 | 9.25E-001 | 4.88E-001 | 9.03E-001 | 1.00E+000 | 4.72E-001 |
| 826 | 7 | 9 | *Pseudomonas aeruginosa fdr1 Lactobacillus acidophilus Pseudomonas aeruginosa fdr440 Porphyromonas gingivalis Mycobacterium tuberculosis Escherichia coli Aggregatibacter actinomycetemcomitans* | 3.03E-001 | 0 | 1.00E+000 | 4.65E-002 | 9.96E-001 | 9.03E-001 | 6.88E-001 | 2.36E-001 | 4.30E-001 | 6.60E-001 | 9.67E-001 | 2.56E-001 | 1.23E-001 | 1.00E+000 |
| 828 | 7 | 9 | *Burkholderia pseudomallei Lactobacillus acidophilus Listeria monocytogenes Bifidobacterium bifidum Mycobacterium tuberculosis Escherichia coli Aggregatibacter actinomycetemcomitans* | 3.03E-001 | 0 | 1.00E+000 | 2.05E-001 | 9.53E-001 | 2.56E-001 | 9.86E-001 | 9.76E-001 | 3.05E-003 | 3.12E-001 | 8.03E-001 | 9.03E-001 | 5.32E-001 | 8.42E-001 |
| 814 | 7 | 9 | *Burkholderia pseudomallei Pseudomonas aeruginosa fdr1 Lactobacillus acidophilus Bifidobacterium bifidum Helicobacter pylori kx2 mgep Helicobacter pylori kx1 npgec Escherichia coli* | 3.03E-001 | 2 | 2.49E-001 | 7.95E-001 | 5.00E-001 | 6.02E-001 | 6.88E-001 | 5.46E-001 | 6.71E-002 | 6.60E-001 | 4.88E-001 | 9.03E-001 | 1.00E+000 | 8.42E-001 |
| 62 | 4 | 111 | *Yersinia enterocolitica wap bl6 Helicobacter pylori kx2 npgec Burkholderia pseudomallei Porphyromonas gingivalis* | 3.05E-001 | 28 | 3.07E-005 | 6.97E-001 | 6.97E-001 | 1.00E+000 | 2.45E-001 | 4.95E-001 | 1.00E+000 | 3.20E-001 | 4.51E-001 | 1.00E+000 | 3.41E-001 | 6.34E-001 |
| 60 | 4 | 111 | *Pseudomonas aeruginosa Porphyromonas gingivalis Aeromonas cavia Helicobacter pylori kx1 npgec* | 3.05E-001 | 19 | 2.09E-004 | 6.97E-001 | 6.97E-001 | 1.00E+000 | 2.45E-001 | 4.95E-001 | 1.00E+000 | 1.00E+000 | 4.51E-001 | 7.19E-001 | 3.41E-001 | 1.00E+000 |
| 166 | 5 | 49 | *Burkholderia pseudomallei Staphylococcus aureus Mycobacterium tuberculosis Aeromonas cavia Helicobacter pylori kx1 npgec* | 3.06E-001 | 1 | 6.96E-001 | 5.00E-001 | 8.28E-001 | 8.00E-001 | 8.69E-001 | 6.41E-001 | 1.00E+000 | 4.46E-001 | 5.96E-001 | 3.93E-001 | 1.00E+000 | 7.21E-001 |
| 164 | 5 | 49 | *Burkholderia pseudomallei Lactobacillus acidophilus streptococcus pneumoniae d39 Helicobacter pylori kx1 npgec Escherichia coli* | 3.06E-001 | 4 | 2.85E-001 | 5.00E-001 | 8.28E-001 | 3.93E-001 | 8.69E-001 | 6.41E-001 | 3.23E-001 | 4.46E-001 | 5.96E-001 | 8.00E-001 | 1.00E+000 | 7.21E-001 |
| 52 | 4 | 118 | *Burkholderia pseudomallei Lactobacillus acidophilus Bifidobacterium bifidum streptococcus pneumoniae d39* | 3.10E-001 | 46 | 1.59E-007 | 6.97E-001 | 6.97E-001 | 4.86E-002 | 9.96E-001 | 8.66E-001 | 2.02E-002 | 7.55E-001 | 1.00E+000 | 7.19E-001 | 1.00E+000 | 6.34E-001 |
| 312 | 8 | 19 | *Yersinia enterocolitica p60 bc Lactobacillus acidophilus Staphylococcus aureus Bifidobacterium bifidum Porphyromonas gingivalis Aeromonas cavia Aggregatibacter actinomycetemcomitans Ehrlichia chaffeensis wakulla* | 3.17E-003 | 2 | 2.16E-001 | 8.81E-001 | 3.48E-001 | 3.45E-001 | 8.54E-001 | 6.64E-001 | 8.78E-002 | 9.50E-001 | 8.68E-001 | 9.33E-001 | 1.58E-001 | 8.82E-001 |
| 316 | 8 | 19 | *Helicobacter pylori kx2 npgec Porphyromonas gingivalis streptococcus pneumoniae d39 Helicobacter pylori kx1 npgec Aggregatibacter actinomycetemcomitans Pseudomonas aeruginosa fdr440 Helicobacter pylori kx2 mgep Escherichia coli* | 3.17E-003 | 0 | 1.00E+000 | 3.48E-001 | 8.81E-001 | 9.33E-001 | 2.57E-001 | 2.63E-003 | 1.00E+000 | 9.50E-001 | 2.94E-001 | 6.88E-001 | 1.58E-001 | 1.00E+000 |
| 458 | 6 | 19 | *Burkholderia pseudomallei Lactobacillus acidophilus Bifidobacterium bifidum Pseudomonas aeruginosa Aeromonas cavia Ehrlichia chaffeensis liberty* | 3.19E-001 | 6 | 4.44E-003 | 9.08E-001 | 3.31E-001 | 5.03E-001 | 7.84E-001 | 1.00E+000 | 4.88E-002 | 8.87E-001 | 9.44E-001 | 8.60E-001 | 1.00E+000 | 3.82E-001 |
| 462 | 6 | 19 | *Yersinia enterocolitica wap bl6 Yersinia enterocolitica p60 bc Staphylococcus aureus Helicobacter pylori kx2 mgep Aeromonas cavia Helicobacter pylori kx1 npgec* | 3.19E-001 | 13 | 1.39E-004 | 9.90E-001 | 9.18E-002 | 8.60E-001 | 4.39E-001 | 4.13E-001 | 1.00E+000 | 5.61E-001 | 1.64E-002 | 8.60E-001 | 1.00E+000 | 1.00E+000 |
| 464 | 6 | 19 | *Helicobacter pylori kx2 npgec Burkholderia pseudomallei streptococcus gordonii Staphylococcus aureus Bifidobacterium bifidum Helicobacter pylori kx1 npgec* | 3.19E-001 | 0 | 1.00E+000 | 6.69E-001 | 6.69E-001 | 1.73E-001 | 9.54E-001 | 1.36E-001 | 3.78E-001 | 8.87E-001 | 7.13E-001 | 8.60E-001 | 4.74E-001 | 7.89E-001 |
| 180 | 5 | 47 | *Helicobacter pylori kx2 npgec Lactobacillus acidophilus Staphylococcus aureus Pseudomonas aeruginosa Aggregatibacter actinomycetemcomitans* | 3.21E-001 | 4 | 1.06E-001 | 5.00E-001 | 8.28E-001 | 3.93E-001 | 8.69E-001 | 2.75E-001 | 3.23E-001 | 1.00E+000 | 9.05E-001 | 3.93E-001 | 4.10E-001 | 1.00E+000 |
| 1278 | 11 | 3 | *Yersinia enterocolitica wap bl6 Helicobacter pylori kx2 npgec Lactobacillus acidophilus Staphylococcus aureus Bifidobacterium bifidum Pseudomonas aeruginosa streptococcus pneumoniae d39 Helicobacter pylori kx1 npgec Pseudomonas aeruginosa fdr440 Mycobacterium tuberculosis Escherichia coli* | 3.28E-001 | 0 | 1.00E+000 | 5.00E-001 | 7.58E-001 | 3.03E-001 | 9.64E-001 | 4.07E-001 | 1.63E-001 | 6.82E-001 | 6.16E-001 | 1.00E-001 | 1.00E+000 | 1.00E+000 |
| 1292 | 11 | 3 | *Yersinia enterocolitica wap bl6 Helicobacter pylori kx2 npgec Burkholderia pseudomallei Yersinia enterocolitica p60 bl6 Yersinia enterocolitica p60 bc Lactobacillus acidophilus Bifidobacterium bifidum Porphyromonas gingivalis Aeromonas cavia Ehrlichia chaffeensis wakulla Pseudomonas aeruginosa fdr1* | 3.28E-001 | 0 | 1.00E+000 | 9.84E-001 | 7.95E-002 | 8.66E-001 | 3.18E-001 | 8.91E-001 | 1.63E-001 | 3.82E-001 | 3.33E-001 | 9.80E-001 | 7.19E-001 | 7.60E-001 |
| 1282 | 11 | 3 | *Helicobacter pylori kx2 npgec Staphylococcus aureus Porphyromonas gingivalis Aeromonas cavia streptococcus pneumoniae d39 Helicobacter pylori kx1 npgec Ehrlichia chaffeensis liberty Pseudomonas aeruginosa fdr1 Pseudomonas aeruginosa fdr440 Mycobacterium tuberculosis Escherichia coli* | 3.28E-001 | 0 | 1.00E+000 | 2.42E-001 | 9.21E-001 | 8.66E-001 | 6.18E-001 | 4.87E-002 | 1.00E+000 | 9.03E-001 | 6.16E-001 | 1.00E-001 | 7.19E-001 | 9.55E-001 |
| 1280 | 11 | 3 | *Helicobacter pylori kx2 npgec Burkholderia pseudomallei Lactobacillus acidophilus Staphylococcus aureus Bifidobacterium bifidum Porphyromonas gingivalis Helicobacter pylori kx1 npgec Ehrlichia chaffeensis liberty Pseudomonas aeruginosa fdr440 Mycobacterium tuberculosis Escherichia coli* | 3.28E-001 | 0 | 1.00E+000 | 5.00E-001 | 7.58E-001 | 6.07E-001 | 8.54E-001 | 4.07E-001 | 1.63E-001 | 6.82E-001 | 8.52E-001 | 6.07E-001 | 7.19E-001 | 7.60E-001 |
| 1290 | 11 | 3 | *Helicobacter pylori kx2 npgec Lactobacillus acidophilus Staphylococcus aureus Bifidobacterium bifidum Pseudomonas aeruginosa Porphyromonas gingivalis Aeromonas cavia streptococcus pneumoniae d39 Helicobacter pylori kx1 npgec Mycobacterium tuberculosis Escherichia coli* | 3.28E-001 | 0 | 1.00E+000 | 5.00E-001 | 7.58E-001 | 3.03E-001 | 9.64E-001 | 4.07E-001 | 1.63E-001 | 9.03E-001 | 6.16E-001 | 3.03E-001 | 7.19E-001 | 1.00E+000 |
| 188 | 5 | 46 | *Yersinia enterocolitica wap bl6 Staphylococcus aureus Porphyromonas gingivalis streptococcus pneumoniae d39 Helicobacter pylori kx1 npgec* | 3.32E-001 | 26 | 1.84E-004 | 5.00E-001 | 8.28E-001 | 3.93E-001 | 8.69E-001 | 6.08E-002 | 1.00E+000 | 8.32E-001 | 5.96E-001 | 3.93E-001 | 4.10E-001 | 1.00E+000 |
| 488 | 6 | 18 | *Yersinia enterocolitica wap bl6 Burkholderia pseudomallei Lactobacillus acidophilus Bifidobacterium bifidum Pseudomonas aeruginosa Escherichia coli* | 3.42E-001 | 1 | 4.73E-001 | 6.69E-001 | 6.69E-001 | 5.03E-001 | 7.84E-001 | 1.00E+000 | 4.88E-002 | 2.16E-001 | 7.13E-001 | 8.60E-001 | 1.00E+000 | 7.89E-001 |
| 490 | 6 | 18 | *Burkholderia pseudomallei Pseudomonas aeruginosa fdr1 Lactobacillus acidophilus Porphyromonas gingivalis Aeromonas cavia Aggregatibacter actinomycetemcomitans* | 3.42E-001 | 0 | 1.00E+000 | 3.31E-001 | 9.08E-001 | 8.60E-001 | 4.39E-001 | 4.13E-001 | 3.78E-001 | 8.87E-001 | 9.44E-001 | 8.60E-001 | 9.10E-002 | 7.89E-001 |
| 206 | 5 | 44 | *Burkholderia pseudomallei streptococcus gordonii Lactobacillus acidophilus Bifidobacterium bifidum Fusobacterium nucleatum* | 3.50E-001 | 13 | 5.52E-003 | 5.00E-001 | 8.28E-001 | 1.03E-001 | 9.82E-001 | 6.41E-001 | 3.31E-002 | 8.32E-001 | 1.00E+000 | 1.00E+000 | 6.29E-002 | 7.21E-001 |
| 202 | 5 | 44 | *Burkholderia pseudomallei Pseudomonas aeruginosa Aeromonas cavia Helicobacter pylori kx1 npgec Aggregatibacter actinomycetemcomitans* | 3.50E-001 | 0 | 1.00E+000 | 5.00E-001 | 8.28E-001 | 1.00E+000 | 1.68E-001 | 6.41E-001 | 1.00E+000 | 8.32E-001 | 5.96E-001 | 8.00E-001 | 4.10E-001 | 7.21E-001 |
| 204 | 5 | 44 | *Burkholderia pseudomallei Listeria monocytogenes Bifidobacterium bifidum Mycobacterium tuberculosis Escherichia coli* | 3.50E-001 | 3 | 2.53E-001 | 1.72E-001 | 9.76E-001 | 3.93E-001 | 9.82E-001 | 1.00E+000 | 3.31E-002 | 1.31E-001 | 5.96E-001 | 8.00E-001 | 1.00E+000 | 7.21E-001 |
| 230 | 8 | 25 | *Yersinia enterocolitica wap bl6 Helicobacter pylori kx2 npgec Burkholderia pseudomallei Yersinia enterocolitica p60 bc Lactobacillus acidophilus Bifidobacterium bifidum Helicobacter pylori kx1 npgec Helicobacter pylori kx2 mgep* | 3.56E-004 | 6 | 3.67E-002 | 9.98E-001 | 2.24E-002 | 6.88E-001 | 5.89E-001 | 6.64E-001 | 8.78E-002 | 4.11E-001 | 9.08E-002 | 1.00E+000 | 1.00E+000 | 8.82E-001 |
| 906 | 7 | 8 | *Helicobacter pylori kx2 npgec Lactobacillus acidophilus Porphyromonas gingivalis Mycobacterium tuberculosis Aeromonas cavia Helicobacter pylori kx1 npgec Ehrlichia chaffeensis liberty* | 3.58E-001 | 0 | 1.00E+000 | 9.53E-001 | 2.05E-001 | 9.03E-001 | 6.88E-001 | 5.46E-001 | 4.30E-001 | 9.25E-001 | 4.88E-001 | 9.03E-001 | 5.32E-001 | 8.42E-001 |
| 916 | 7 | 8 | *Yersinia enterocolitica wap bl6 Helicobacter pylori kx2 npgec Yersinia enterocolitica p60 bl6 Yersinia enterocolitica p60 bc Pseudomonas aeruginosa fdr1234 Staphylococcus aureus Helicobacter pylori kx1 npgec* | 3.58E-001 | 2 | 1.50E-001 | 9.53E-001 | 2.05E-001 | 9.03E-001 | 3.40E-001 | 2.36E-001 | 1.00E+000 | 3.12E-001 | 4.43E-002 | 6.02E-001 | 1.00E+000 | 1.00E+000 |
| 918 | 7 | 8 | *Yersinia enterocolitica wap bl6 Helicobacter pylori kx2 npgec Ehrlichia chaffeensis wakulla Pseudomonas aeruginosa fdr1 Porphyromonas gingivalis Aeromonas cavia Helicobacter pylori kx1 npgec* | 3.58E-001 | 2 | 2.16E-001 | 9.53E-001 | 2.05E-001 | 1.00E+000 | 7.55E-002 | 2.36E-001 | 1.00E+000 | 9.25E-001 | 1.92E-001 | 9.03E-001 | 5.32E-001 | 8.42E-001 |
| 904 | 7 | 8 | *Burkholderia pseudomallei Pseudomonas aeruginosa fdr1 Lactobacillus acidophilus Pseudomonas aeruginosa fdr440 Aeromonas cavia Escherichia coli Ehrlichia chaffeensis liberty* | 3.58E-001 | 0 | 1.00E+000 | 5.00E-001 | 7.95E-001 | 9.03E-001 | 3.40E-001 | 8.39E-001 | 4.30E-001 | 6.60E-001 | 8.03E-001 | 6.02E-001 | 1.00E+000 | 4.72E-001 |
| 886 | 7 | 8 | *Yersinia enterocolitica wap bl6 Helicobacter pylori kx2 npgec Bifidobacterium bifidum Porphyromonas gingivalis Helicobacter pylori kx2 mgep Helicobacter pylori kx1 npgec Escherichia coli* | 3.58E-001 | 2 | 2.83E-001 | 9.53E-001 | 2.05E-001 | 9.03E-001 | 3.40E-001 | 2.36E-001 | 4.30E-001 | 6.60E-001 | 4.43E-002 | 1.00E+000 | 5.32E-001 | 1.00E+000 |
| 894 | 7 | 8 | *Burkholderia pseudomallei Lactobacillus acidophilus Pseudomonas aeruginosa fdr440 Staphylococcus aureus Bifidobacterium bifidum streptococcus pneumoniae d39 Helicobacter pylori kx1 npgec* | 3.58E-001 | 0 | 1.00E+000 | 5.00E-001 | 7.95E-001 | 6.35E-002 | 9.86E-001 | 2.36E-001 | 6.71E-002 | 9.25E-001 | 9.67E-001 | 2.56E-001 | 1.00E+000 | 8.42E-001 |
| 902 | 7 | 8 | *Burkholderia pseudomallei streptococcus gordonii Lactobacillus acidophilus Bifidobacterium bifidum Fusobacterium nucleatum Helicobacter pylori kx1 npgec Escherichia coli* | 3.58E-001 | 0 | 1.00E+000 | 5.00E-001 | 7.95E-001 | 2.56E-001 | 9.12E-001 | 5.46E-001 | 6.71E-002 | 6.60E-001 | 8.03E-001 | 1.00E+000 | 1.23E-001 | 8.42E-001 |
| 896 | 7 | 8 | *Yersinia enterocolitica wap bl6 Helicobacter pylori kx2 npgec Pseudomonas aeruginosa fdr1 Pseudomonas aeruginosa fdr440 Pseudomonas aeruginosa Mycobacterium tuberculosis Helicobacter pylori kx1 npgec* | 3.58E-001 | 1 | 3.67E-001 | 5.00E-001 | 7.95E-001 | 1.00E+000 | 3.40E-001 | 2.36E-001 | 1.00E+000 | 6.60E-001 | 4.88E-001 | 6.35E-002 | 1.00E+000 | 1.00E+000 |
| 912 | 7 | 8 | *Helicobacter pylori kx2 npgec Lactobacillus acidophilus Pseudomonas aeruginosa fdr440 Bifidobacterium bifidum Helicobacter pylori kx2 mgep Helicobacter pylori kx1 npgec Ehrlichia chaffeensis liberty* | 3.58E-001 | 0 | 1.00E+000 | 9.96E-001 | 4.65E-002 | 6.02E-001 | 6.88E-001 | 2.36E-001 | 6.71E-002 | 1.00E+000 | 4.88E-001 | 9.03E-001 | 1.00E+000 | 8.42E-001 |
| 900 | 7 | 8 | *Pseudomonas aeruginosa fdr440 Staphylococcus aureus Listeria monocytogenes Pseudomonas aeruginosa Porphyromonas gingivalis streptococcus pneumoniae d39 Helicobacter pylori kx1 npgec* | 3.58E-001 | 0 | 1.00E+000 | 4.65E-002 | 9.96E-001 | 2.56E-001 | 9.12E-001 | 6.08E-002 | 4.30E-001 | 1.00E+000 | 8.03E-001 | 6.35E-002 | 5.32E-001 | 1.00E+000 |
| 212 | 5 | 43 | *Helicobacter pylori kx2 npgec Staphylococcus aureus Helicobacter pylori kx2 mgep Helicobacter pylori kx1 npgec Aggregatibacter actinomycetemcomitans* | 3.60E-001 | 18 | 2.48E-004 | 8.28E-001 | 5.00E-001 | 8.00E-001 | 5.54E-001 | 5.13E-003 | 1.00E+000 | 1.00E+000 | 2.35E-001 | 8.00E-001 | 4.10E-001 | 1.00E+000 |
| 280 | 7 | 23 | *Yersinia enterocolitica wap bl6 Burkholderia pseudomallei Yersinia enterocolitica p60 bc Lactobacillus acidophilus Bifidobacterium bifidum Porphyromonas gingivalis Escherichia coli* | 3.66E-002 | 2 | 2.83E-001 | 7.95E-001 | 5.00E-001 | 6.02E-001 | 6.88E-001 | 9.76E-001 | 6.71E-002 | 8.83E-002 | 4.88E-001 | 1.00E+000 | 5.32E-001 | 8.42E-001 |
| 506 | 6 | 17 | *Helicobacter pylori kx2 npgec Lactobacillus acidophilus Pseudomonas aeruginosa fdr440 streptococcus pneumoniae d39 Helicobacter pylori kx1 npgec Aggregatibacter actinomycetemcomitans* | 3.67E-001 | 0 | 1.00E+000 | 6.69E-001 | 6.69E-001 | 5.03E-001 | 7.84E-001 | 2.32E-002 | 3.78E-001 | 1.00E+000 | 7.13E-001 | 5.03E-001 | 4.74E-001 | 1.00E+000 |
| 498 | 6 | 17 | *Yersinia enterocolitica wap bl6 Helicobacter pylori kx2 npgec Burkholderia pseudomallei Pseudomonas aeruginosa Mycobacterium tuberculosis Helicobacter pylori kx1 npgec* | 3.67E-001 | 0 | 1.00E+000 | 6.69E-001 | 6.69E-001 | 1.00E+000 | 4.39E-001 | 7.55E-001 | 1.00E+000 | 2.16E-001 | 3.61E-001 | 5.03E-001 | 1.00E+000 | 7.89E-001 |
| 500 | 6 | 17 | *Helicobacter pylori kx2 npgec Lactobacillus acidophilus Pseudomonas aeruginosa Mycobacterium tuberculosis Helicobacter pylori kx2 mgep Helicobacter pylori kx1 npgec* | 3.67E-001 | 2 | 1.20E-001 | 9.08E-001 | 3.31E-001 | 8.60E-001 | 7.84E-001 | 4.13E-001 | 3.78E-001 | 8.87E-001 | 3.61E-001 | 5.03E-001 | 1.00E+000 | 1.00E+000 |
| 502 | 6 | 17 | *Burkholderia pseudomallei Pseudomonas aeruginosa fdr440 Staphylococcus aureus Pseudomonas aeruginosa Porphyromonas gingivalis Aeromonas cavia* | 3.67E-001 | 4 | 3.06E-003 | 9.18E-002 | 9.90E-001 | 8.60E-001 | 4.39E-001 | 4.13E-001 | 1.00E+000 | 8.87E-001 | 9.44E-001 | 1.73E-001 | 4.74E-001 | 7.89E-001 |
| 504 | 6 | 17 | *Lactobacillus acidophilus Staphylococcus aureus Mycobacterium tuberculosis Aeromonas cavia Helicobacter pylori kx1 npgec Escherichia coli* | 3.67E-001 | 0 | 1.00E+000 | 6.69E-001 | 6.69E-001 | 5.03E-001 | 9.54E-001 | 7.55E-001 | 3.78E-001 | 5.61E-001 | 3.61E-001 | 5.03E-001 | 1.00E+000 | 1.00E+000 |
| 112 | 6 | 52 | *Helicobacter pylori kx2 npgec Pseudomonas aeruginosa Porphyromonas gingivalis streptococcus pneumoniae d39 Helicobacter pylori kx1 npgec Escherichia coli* | 3.69E-002 | 4 | 3.47E-001 | 3.31E-001 | 9.08E-001 | 8.60E-001 | 4.39E-001 | 1.36E-001 | 1.00E+000 | 8.87E-001 | 3.61E-001 | 5.03E-001 | 4.74E-001 | 1.00E+000 |
| 606 | 8 | 11 | *Lactobacillus acidophilus Staphylococcus aureus Porphyromonas gingivalis Aeromonas cavia streptococcus pneumoniae d39 Helicobacter pylori kx1 npgec Aggregatibacter actinomycetemcomitans Mycobacterium tuberculosis* | 3.71E-002 | 0 | 1.00E+000 | 3.48E-001 | 8.81E-001 | 3.45E-001 | 9.69E-001 | 1.21E-001 | 4.79E-001 | 9.50E-001 | 8.68E-001 | 3.45E-001 | 1.58E-001 | 1.00E+000 |
| 602 | 8 | 11 | *Yersinia enterocolitica wap bl6 Yersinia enterocolitica p60 bl6 Lactobacillus acidophilus Staphylococcus aureus Bifidobacterium bifidum Porphyromonas gingivalis streptococcus pneumoniae d39 Helicobacter pylori kx1 npgec* | 3.71E-002 | 6 | 4.91E-002 | 8.81E-001 | 3.48E-001 | 1.07E-001 | 9.69E-001 | 3.51E-001 | 8.78E-002 | 7.43E-001 | 6.05E-001 | 6.88E-001 | 5.86E-001 | 1.00E+000 |
| 214 | 5 | 42 | *Yersinia enterocolitica wap bl6 Burkholderia pseudomallei Staphylococcus aureus Pseudomonas aeruginosa Helicobacter pylori kx1 npgec* | 3.71E-001 | 13 | 1.49E-003 | 5.00E-001 | 8.28E-001 | 8.00E-001 | 5.54E-001 | 6.41E-001 | 1.00E+000 | 4.46E-001 | 5.96E-001 | 3.93E-001 | 1.00E+000 | 7.21E-001 |
| 216 | 5 | 42 | *Burkholderia pseudomallei Staphylococcus aureus Bifidobacterium bifidum Porphyromonas gingivalis Mycobacterium tuberculosis* | 3.71E-001 | 3 | 6.01E-001 | 1.72E-001 | 9.76E-001 | 3.93E-001 | 9.82E-001 | 6.41E-001 | 3.23E-001 | 4.46E-001 | 1.00E+000 | 3.93E-001 | 4.10E-001 | 7.21E-001 |
| 68 | 4 | 108 | *Helicobacter pylori kx2 npgec Helicobacter pylori kx2 mgep Aeromonas cavia Helicobacter pylori kx1 npgec* | 3.76E-001 | 65 | 1.82E-012 | 1.00E+000 | 5.35E-002 | 1.00E+000 | 2.45E-001 | 1.46E-001 | 1.00E+000 | 1.00E+000 | 1.22E-002 | 1.00E+000 | 1.00E+000 | 1.00E+000 |
| 220 | 5 | 41 | *Burkholderia pseudomallei Bifidobacterium bifidum Helicobacter pylori kx2 mgep Helicobacter pylori kx1 npgec Escherichia coli* | 3.82E-001 | 9 | 2.55E-003 | 8.28E-001 | 5.00E-001 | 8.00E-001 | 5.54E-001 | 6.41E-001 | 3.23E-001 | 4.46E-001 | 2.35E-001 | 1.00E+000 | 1.00E+000 | 7.21E-001 |
| 222 | 5 | 41 | *Burkholderia pseudomallei Lactobacillus acidophilus Bifidobacterium bifidum Helicobacter pylori kx2 mgep Aeromonas cavia* | 3.82E-001 | 12 | 6.05E-005 | 9.76E-001 | 1.72E-001 | 3.93E-001 | 8.69E-001 | 9.23E-001 | 3.31E-002 | 8.32E-001 | 5.96E-001 | 1.00E+000 | 1.00E+000 | 7.21E-001 |
| 596 | 9 | 10 | *Yersinia enterocolitica wap bl6 Helicobacter pylori kx2 npgec Yersinia enterocolitica p60 bl6 Yersinia enterocolitica p60 bc Lactobacillus acidophilus Bifidobacterium bifidum Aeromonas cavia Ehrlichia chaffeensis wakulla Mycobacterium tuberculosis* | 3.83E-003 | 0 | 1.00E+000 | 9.99E-001 | 1.02E-002 | 7.60E-001 | 7.84E-001 | 9.93E-001 | 1.11E-001 | 2.16E-001 | 1.56E-001 | 9.55E-001 | 1.00E+000 | 9.14E-001 |
| 540 | 6 | 16 | *Yersinia enterocolitica wap bl6 Helicobacter pylori kx2 npgec Burkholderia pseudomallei Yersinia enterocolitica p60 bl6 Porphyromonas gingivalis Escherichia coli* | 3.93E-001 | 4 | 1.63E-002 | 6.69E-001 | 6.69E-001 | 1.00E+000 | 1.13E-001 | 7.55E-001 | 1.00E+000 | 4.58E-002 | 1.08E-001 | 1.00E+000 | 4.74E-001 | 7.89E-001 |
| 536 | 6 | 16 | *Yersinia enterocolitica wap bl6 Helicobacter pylori kx2 npgec Helicobacter pylori kx1 npgec Escherichia coli Ehrlichia chaffeensis liberty Aggregatibacter actinomycetemcomitans* | 3.93E-001 | 5 | 3.17E-002 | 9.08E-001 | 3.31E-001 | 1.00E+000 | 1.13E-001 | 4.13E-001 | 1.00E+000 | 5.61E-001 | 1.08E-001 | 1.00E+000 | 4.74E-001 | 7.89E-001 |
| 542 | 6 | 16 | *Yersinia enterocolitica wap bl6 Helicobacter pylori kx2 npgec Burkholderia pseudomallei Helicobacter pylori kx2 mgep Helicobacter pylori kx1 npgec Aggregatibacter actinomycetemcomitans* | 3.93E-001 | 0 | 1.00E+000 | 9.08E-001 | 3.31E-001 | 1.00E+000 | 1.13E-001 | 1.36E-001 | 1.00E+000 | 5.61E-001 | 1.08E-001 | 1.00E+000 | 4.74E-001 | 7.89E-001 |
| 554 | 6 | 16 | *Helicobacter pylori kx2 npgec Burkholderia pseudomallei Pseudomonas aeruginosa fdr1 Bifidobacterium bifidum Helicobacter pylori kx1 npgec Aggregatibacter actinomycetemcomitans* | 3.93E-001 | 0 | 1.00E+000 | 6.69E-001 | 6.69E-001 | 8.60E-001 | 4.39E-001 | 1.36E-001 | 3.78E-001 | 8.87E-001 | 7.13E-001 | 8.60E-001 | 4.74E-001 | 7.89E-001 |
| 546 | 6 | 16 | *Helicobacter pylori kx2 npgec Pseudomonas aeruginosa fdr440 Staphylococcus aureus Listeria monocytogenes Helicobacter pylori kx1 npgec Aggregatibacter actinomycetemcomitans* | 3.93E-001 | 0 | 1.00E+000 | 3.31E-001 | 9.08E-001 | 5.03E-001 | 7.84E-001 | 2.32E-002 | 3.78E-001 | 1.00E+000 | 3.61E-001 | 5.03E-001 | 4.74E-001 | 1.00E+000 |
| 228 | 5 | 40 | *Yersinia enterocolitica wap bl6 Helicobacter pylori kx2 npgec Bifidobacterium bifidum Helicobacter pylori kx1 npgec Escherichia coli* | 3.93E-001 | 7 | 1.33E-001 | 9.76E-001 | 1.72E-001 | 8.00E-001 | 5.54E-001 | 6.41E-001 | 3.23E-001 | 4.46E-001 | 4.69E-002 | 1.00E+000 | 1.00E+000 | 1.00E+000 |
| 70 | 4 | 103 | *Helicobacter pylori kx2 npgec Pseudomonas aeruginosa fdr440 Porphyromonas gingivalis Helicobacter pylori kx1 npgec* | 4.01E-001 | 23 | 5.70E-003 | 6.97E-001 | 6.97E-001 | 1.00E+000 | 2.45E-001 | 1.63E-002 | 1.00E+000 | 1.00E+000 | 4.51E-001 | 7.19E-001 | 3.41E-001 | 1.00E+000 |
| 236 | 5 | 39 | *Helicobacter pylori kx2 npgec streptococcus pneumoniae d39 Helicobacter pylori kx1 npgec Escherichia coli Ehrlichia chaffeensis liberty* | 4.06E-001 | 21 | 1.62E-003 | 8.28E-001 | 5.00E-001 | 8.00E-001 | 5.54E-001 | 2.75E-001 | 1.00E+000 | 8.32E-001 | 2.35E-001 | 8.00E-001 | 1.00E+000 | 7.21E-001 |
| 246 | 5 | 38 | *Yersinia enterocolitica wap bl6 Burkholderia pseudomallei Bifidobacterium bifidum streptococcus pneumoniae d39 Helicobacter pylori kx1 npgec* | 4.20E-001 | 17 | 1.11E-003 | 8.28E-001 | 5.00E-001 | 3.93E-001 | 8.69E-001 | 6.41E-001 | 3.23E-001 | 4.46E-001 | 5.96E-001 | 8.00E-001 | 1.00E+000 | 7.21E-001 |
| 304 | 7 | 22 | *Yersinia enterocolitica wap bl6 Helicobacter pylori kx2 npgec Lactobacillus acidophilus Staphylococcus aureus Aeromonas cavia Helicobacter pylori kx1 npgec Ehrlichia chaffeensis liberty* | 4.21E-002 | 6 | 2.36E-003 | 9.96E-001 | 4.65E-002 | 6.02E-001 | 6.88E-001 | 5.46E-001 | 4.30E-001 | 9.25E-001 | 1.92E-001 | 9.03E-001 | 1.00E+000 | 8.42E-001 |
| 580 | 6 | 15 | *Yersinia enterocolitica wap bl6 Burkholderia pseudomallei Pseudomonas aeruginosa Porphyromonas gingivalis Helicobacter pylori kx1 npgec Ehrlichia chaffeensis liberty* | 4.23E-001 | 4 | 7.97E-003 | 6.69E-001 | 6.69E-001 | 1.00E+000 | 1.13E-001 | 7.55E-001 | 1.00E+000 | 5.61E-001 | 7.13E-001 | 8.60E-001 | 4.74E-001 | 3.82E-001 |
| 594 | 6 | 15 | *Helicobacter pylori kx2 npgec Pseudomonas aeruginosa Helicobacter pylori kx2 mgep Aeromonas cavia Helicobacter pylori kx1 npgec Ehrlichia chaffeensis liberty* | 4.23E-001 | 6 | 4.44E-003 | 9.90E-001 | 9.18E-002 | 1.00E+000 | 1.13E-001 | 4.13E-001 | 1.00E+000 | 1.00E+000 | 1.08E-001 | 8.60E-001 | 1.00E+000 | 7.89E-001 |
| 586 | 6 | 15 | *Helicobacter pylori kx2 npgec Ehrlichia chaffeensis wakulla Lactobacillus acidophilus Bifidobacterium bifidum streptococcus pneumoniae d39 Helicobacter pylori kx1 npgec* | 4.23E-001 | 7 | 4.43E-002 | 9.90E-001 | 9.18E-002 | 1.73E-001 | 9.54E-001 | 4.13E-001 | 4.88E-002 | 1.00E+000 | 7.13E-001 | 8.60E-001 | 1.00E+000 | 7.89E-001 |
| 590 | 6 | 15 | *Helicobacter pylori kx2 npgec Burkholderia pseudomallei Staphylococcus aureus Helicobacter pylori kx2 mgep streptococcus pneumoniae d39 Helicobacter pylori kx1 npgec* | 4.23E-001 | 6 | 2.26E-001 | 6.69E-001 | 6.69E-001 | 5.03E-001 | 7.84E-001 | 2.32E-002 | 1.00E+000 | 8.87E-001 | 3.61E-001 | 5.03E-001 | 1.00E+000 | 7.89E-001 |
| 584 | 6 | 15 | *Helicobacter pylori kx2 npgec Ehrlichia chaffeensis arkansa Burkholderia pseudomallei Staphylococcus aureus Bifidobacterium bifidum Pseudomonas aeruginosa* | 4.23E-001 | 0 | 1.00E+000 | 6.69E-001 | 6.69E-001 | 5.03E-001 | 7.84E-001 | 7.55E-001 | 3.78E-001 | 8.87E-001 | 9.44E-001 | 5.03E-001 | 1.00E+000 | 3.82E-001 |
| 592 | 6 | 15 | *Helicobacter pylori kx2 npgec Porphyromonas gingivalis Helicobacter pylori kx2 mgep streptococcus pneumoniae d39 Helicobacter pylori kx1 npgec Ehrlichia chaffeensis liberty* | 4.23E-001 | 11 | 7.28E-003 | 9.08E-001 | 3.31E-001 | 8.60E-001 | 4.39E-001 | 2.32E-002 | 1.00E+000 | 1.00E+000 | 3.61E-001 | 8.60E-001 | 4.74E-001 | 7.89E-001 |
| 74 | 4 | 99 | *Burkholderia pseudomallei Lactobacillus acidophilus Pseudomonas aeruginosa fdr440 Bifidobacterium bifidum* | 4.24E-001 | 5 | 6.20E-002 | 6.97E-001 | 6.97E-001 | 2.77E-001 | 9.37E-001 | 8.66E-001 | 2.02E-002 | 7.55E-001 | 1.00E+000 | 7.19E-001 | 1.00E+000 | 6.34E-001 |
| 1020 | 7 | 7 | *Burkholderia pseudomallei Lactobacillus acidophilus Pseudomonas aeruginosa fdr440 Staphylococcus aureus Bifidobacterium bifidum Helicobacter pylori kx2 mgep Helicobacter pylori kx1 npgec* | 4.26E-001 | 0 | 1.00E+000 | 7.95E-001 | 5.00E-001 | 2.56E-001 | 9.12E-001 | 2.36E-001 | 6.71E-002 | 9.25E-001 | 8.03E-001 | 6.02E-001 | 1.00E+000 | 8.42E-001 |
| 992 | 7 | 7 | *Yersinia enterocolitica wap bl6 Helicobacter pylori kx2 npgec Helicobacter pylori kx1 mgep Burkholderia pseudomallei Yersinia enterocolitica p60 bl6 Helicobacter pylori kx2 mgep Helicobacter pylori kx1 npgec* | 4.26E-001 | 0 | 1.00E+000 | 9.96E-001 | 4.65E-002 | 1.00E+000 | 7.55E-002 | 2.36E-001 | 1.00E+000 | 3.12E-001 | 5.25E-003 | 1.00E+000 | 1.00E+000 | 8.42E-001 |
| 1000 | 7 | 7 | *Burkholderia pseudomallei Yersinia enterocolitica p60 bl6 Pseudomonas aeruginosa fdr440 Listeria monocytogenes Mycobacterium tuberculosis Helicobacter pylori kx1 npgec Escherichia coli* | 4.26E-001 | 0 | 1.00E+000 | 2.05E-001 | 9.53E-001 | 9.03E-001 | 6.88E-001 | 8.39E-001 | 4.30E-001 | 8.83E-002 | 1.92E-001 | 6.02E-001 | 1.00E+000 | 8.42E-001 |
| 1012 | 7 | 7 | *Yersinia enterocolitica wap bl6 Bifidobacterium bifidum Porphyromonas gingivalis Mycobacterium tuberculosis streptococcus pneumoniae d39 Helicobacter pylori kx1 npgec Aggregatibacter actinomycetemcomitans* | 4.26E-001 | 0 | 1.00E+000 | 5.00E-001 | 7.95E-001 | 6.02E-001 | 9.12E-001 | 2.36E-001 | 4.30E-001 | 6.60E-001 | 8.03E-001 | 6.02E-001 | 1.23E-001 | 1.00E+000 |
| 1002 | 7 | 7 | *Helicobacter pylori kx2 npgec Pseudomonas aeruginosa fdr1 Staphylococcus aureus Porphyromonas gingivalis Helicobacter pylori kx2 mgep Helicobacter pylori kx1 npgec Escherichia coli* | 4.26E-001 | 2 | 2.49E-001 | 5.00E-001 | 7.95E-001 | 9.03E-001 | 3.40E-001 | 8.14E-003 | 1.00E+000 | 9.25E-001 | 1.92E-001 | 6.02E-001 | 5.32E-001 | 1.00E+000 |
| 990 | 7 | 7 | *Ehrlichia chaffeensis arkansa Burkholderia pseudomallei Lactobacillus acidophilus Bifidobacterium bifidum Helicobacter pylori kx2 mgep Helicobacter pylori kx1 npgec Aggregatibacter actinomycetemcomitans* | 4.26E-001 | 5 | 2.83E-005 | 9.53E-001 | 2.05E-001 | 6.02E-001 | 6.88E-001 | 5.46E-001 | 6.71E-002 | 9.25E-001 | 8.03E-001 | 1.00E+000 | 5.32E-001 | 4.72E-001 |
| 1006 | 7 | 7 | *Burkholderia pseudomallei Lactobacillus acidophilus Porphyromonas gingivalis Mycobacterium tuberculosis Aeromonas cavia streptococcus pneumoniae d39 Escherichia coli* | 4.26E-001 | 0 | 1.00E+000 | 2.05E-001 | 9.53E-001 | 6.02E-001 | 9.12E-001 | 8.39E-001 | 4.30E-001 | 3.12E-001 | 8.03E-001 | 6.02E-001 | 5.32E-001 | 8.42E-001 |
| 996 | 7 | 7 | *Yersinia enterocolitica wap bl6 Helicobacter pylori kx2 npgec Yersinia enterocolitica p60 bc Staphylococcus aureus streptococcus pneumoniae d39 Helicobacter pylori kx1 npgec Ehrlichia chaffeensis liberty* | 4.26E-001 | 4 | 1.21E-001 | 9.53E-001 | 2.05E-001 | 6.02E-001 | 6.88E-001 | 2.36E-001 | 1.00E+000 | 6.60E-001 | 1.92E-001 | 6.02E-001 | 1.00E+000 | 8.42E-001 |
| 1014 | 7 | 7 | *Burkholderia pseudomallei Lactobacillus acidophilus Pseudomonas aeruginosa fdr440 Bifidobacterium bifidum Pseudomonas aeruginosa Mycobacterium tuberculosis Aggregatibacter actinomycetemcomitans* | 4.26E-001 | 0 | 1.00E+000 | 2.05E-001 | 9.53E-001 | 6.02E-001 | 9.12E-001 | 8.39E-001 | 6.71E-002 | 6.60E-001 | 1.00E+000 | 2.56E-001 | 5.32E-001 | 8.42E-001 |
| 1004 | 7 | 7 | *Burkholderia pseudomallei Pseudomonas aeruginosa fdr1 Lactobacillus acidophilus Staphylococcus aureus Bifidobacterium bifidum Mycobacterium tuberculosis Aeromonas cavia* | 4.26E-001 | 0 | 1.00E+000 | 5.00E-001 | 7.95E-001 | 2.56E-001 | 9.86E-001 | 8.39E-001 | 6.71E-002 | 6.60E-001 | 9.67E-001 | 2.56E-001 | 1.00E+000 | 8.42E-001 |
| 994 | 7 | 7 | *Yersinia enterocolitica wap bl6 Helicobacter pylori kx2 npgec Ehrlichia chaffeensis wakulla Pseudomonas aeruginosa Porphyromonas gingivalis Aeromonas cavia Helicobacter pylori kx1 npgec* | 4.26E-001 | 1 | 4.73E-001 | 9.53E-001 | 2.05E-001 | 1.00E+000 | 7.55E-002 | 5.46E-001 | 1.00E+000 | 9.25E-001 | 1.92E-001 | 9.03E-001 | 5.32E-001 | 8.42E-001 |
| 988 | 7 | 7 | *Helicobacter pylori kx2 npgec Ehrlichia chaffeensis arkansa Staphylococcus aureus Porphyromonas gingivalis streptococcus pneumoniae d39 Helicobacter pylori kx1 npgec Ehrlichia chaffeensis liberty* | 4.26E-001 | 0 | 1.00E+000 | 7.95E-001 | 5.00E-001 | 6.02E-001 | 6.88E-001 | 6.08E-002 | 1.00E+000 | 1.00E+000 | 8.03E-001 | 6.02E-001 | 5.32E-001 | 4.72E-001 |
| 1008 | 7 | 7 | *Burkholderia pseudomallei streptococcus gordonii Lactobacillus acidophilus Pseudomonas aeruginosa Mycobacterium tuberculosis streptococcus pneumoniae d39 Helicobacter pylori kx1 npgec* | 4.26E-001 | 0 | 1.00E+000 | 2.05E-001 | 9.53E-001 | 2.56E-001 | 9.86E-001 | 5.46E-001 | 4.30E-001 | 6.60E-001 | 9.67E-001 | 2.56E-001 | 5.32E-001 | 8.42E-001 |
| 1018 | 7 | 7 | *Helicobacter pylori kx2 npgec Yersinia enterocolitica p60 bl6 Lactobacillus acidophilus Bifidobacterium bifidum Helicobacter pylori kx2 mgep streptococcus pneumoniae d39 Helicobacter pylori kx1 npgec* | 4.26E-001 | 0 | 1.00E+000 | 9.96E-001 | 4.65E-002 | 2.56E-001 | 9.12E-001 | 2.36E-001 | 6.71E-002 | 9.25E-001 | 1.92E-001 | 9.03E-001 | 1.00E+000 | 1.00E+000 |
| 998 | 7 | 7 | *Helicobacter pylori kx2 npgec Lactobacillus acidophilus Pseudomonas aeruginosa fdr440 Mycobacterium tuberculosis streptococcus pneumoniae d39 Helicobacter pylori kx1 npgec Ehrlichia chaffeensis liberty* | 4.26E-001 | 0 | 1.00E+000 | 7.95E-001 | 5.00E-001 | 6.02E-001 | 9.12E-001 | 2.36E-001 | 4.30E-001 | 9.25E-001 | 8.03E-001 | 2.56E-001 | 1.00E+000 | 8.42E-001 |
| 1010 | 7 | 7 | *Yersinia enterocolitica p60 bc Pseudomonas aeruginosa Porphyromonas gingivalis Mycobacterium tuberculosis Aeromonas cavia Helicobacter pylori kx1 npgec Aggregatibacter actinomycetemcomitans* | 4.26E-001 | 0 | 1.00E+000 | 5.00E-001 | 7.95E-001 | 1.00E+000 | 3.40E-001 | 5.46E-001 | 1.00E+000 | 6.60E-001 | 4.88E-001 | 6.02E-001 | 1.23E-001 | 1.00E+000 |
| 1016 | 7 | 7 | *Helicobacter pylori kx2 npgec streptococcus gordonii Pseudomonas aeruginosa fdr440 Staphylococcus aureus Porphyromonas gingivalis streptococcus pneumoniae d39 Helicobacter pylori kx1 npgec* | 4.26E-001 | 0 | 1.00E+000 | 2.05E-001 | 9.53E-001 | 2.56E-001 | 9.12E-001 | 4.24E-004 | 1.00E+000 | 1.00E+000 | 8.03E-001 | 2.56E-001 | 1.23E-001 | 1.00E+000 |
| 820 | 9 | 7 | *Helicobacter pylori kx2 npgec Burkholderia pseudomallei Yersinia enterocolitica p60 bc Lactobacillus acidophilus Bifidobacterium bifidum Porphyromonas gingivalis streptococcus pneumoniae d39 Helicobacter pylori kx1 npgec Escherichia coli* | 4.26E-002 | 0 | 1.00E+000 | 7.73E-001 | 5.00E-001 | 4.36E-001 | 7.84E-001 | 4.71E-001 | 1.11E-001 | 5.09E-001 | 4.04E-001 | 9.55E-001 | 6.34E-001 | 9.14E-001 |
| 76 | 4 | 98 | *Yersinia enterocolitica wap bl6 Staphylococcus aureus Bifidobacterium bifidum Aeromonas cavia* | 4.28E-001 | 37 | 1.01E-007 | 9.47E-001 | 3.03E-001 | 2.77E-001 | 9.37E-001 | 8.66E-001 | 2.65E-001 | 7.55E-001 | 4.51E-001 | 7.19E-001 | 1.00E+000 | 1.00E+000 |
| 78 | 4 | 97 | *Yersinia enterocolitica wap bl6 Helicobacter pylori kx2 npgec Aeromonas cavia Aggregatibacter actinomycetemcomitans* | 4.39E-001 | 24 | 3.28E-006 | 9.47E-001 | 3.03E-001 | 1.00E+000 | 2.45E-001 | 4.95E-001 | 1.00E+000 | 7.55E-001 | 1.22E-001 | 1.00E+000 | 3.41E-001 | 1.00E+000 |
| 80 | 4 | 97 | *Helicobacter pylori kx2 npgec Burkholderia pseudomallei Porphyromonas gingivalis Mycobacterium tuberculosis* | 4.39E-001 | 10 | 7.80E-004 | 3.03E-001 | 9.47E-001 | 1.00E+000 | 6.80E-001 | 4.95E-001 | 1.00E+000 | 3.20E-001 | 8.43E-001 | 7.19E-001 | 3.41E-001 | 6.34E-001 |
| 254 | 5 | 36 | *Burkholderia pseudomallei Staphylococcus aureus Pseudomonas aeruginosa Porphyromonas gingivalis streptococcus pneumoniae d39* | 4.43E-001 | 14 | 6.06E-002 | 2.39E-002 | 1.00E+000 | 3.93E-001 | 8.69E-001 | 2.75E-001 | 1.00E+000 | 8.32E-001 | 1.00E+000 | 1.03E-001 | 4.10E-001 | 7.21E-001 |
| 256 | 5 | 36 | *Yersinia enterocolitica wap bl6 Staphylococcus aureus Mycobacterium tuberculosis Helicobacter pylori kx1 npgec Aggregatibacter actinomycetemcomitans* | 4.43E-001 | 2 | 6.56E-001 | 5.00E-001 | 8.28E-001 | 8.00E-001 | 8.69E-001 | 2.75E-001 | 1.00E+000 | 4.46E-001 | 5.96E-001 | 3.93E-001 | 4.10E-001 | 1.00E+000 |
| 1246 | 9 | 4 | *Helicobacter pylori kx2 npgec Pseudomonas aeruginosa fdr875 Porphyromonas gingivalis streptococcus pneumoniae d39 Helicobacter pylori kx1 npgec Aggregatibacter actinomycetemcomitans Pseudomonas aeruginosa fdr1 Pseudomonas aeruginosa fdr440 Helicobacter pylori kx2 mgep* | 4.46E-001 | 0 | 1.00E+000 | 2.27E-001 | 9.35E-001 | 9.55E-001 | 1.90E-001 | 2.57E-005 | 1.00E+000 | 1.00E+000 | 7.06E-001 | 1.63E-001 | 1.96E-001 | 1.00E+000 |
| 1220 | 9 | 4 | *Yersinia enterocolitica wap bl6 Burkholderia pseudomallei Yersinia enterocolitica p60 bl6 Staphylococcus aureus Porphyromonas gingivalis Aeromonas cavia Helicobacter pylori kx1 npgec Aggregatibacter actinomycetemcomitans streptococcus gordonii* | 4.46E-001 | 1 | 3.67E-001 | 5.00E-001 | 7.73E-001 | 7.60E-001 | 4.91E-001 | 2.02E-001 | 1.00E+000 | 5.09E-001 | 4.04E-001 | 9.55E-001 | 2.59E-002 | 9.14E-001 |
| 1186 | 9 | 4 | *Yersinia enterocolitica wap bl6 Burkholderia pseudomallei Yersinia enterocolitica p60 bc Lactobacillus acidophilus Bifidobacterium bifidum Pseudomonas aeruginosa Helicobacter pylori kx1 npgec Pseudomonas aeruginosa fdr440 Escherichia coli* | 4.46E-001 | 0 | 1.00E+000 | 7.73E-001 | 5.00E-001 | 7.60E-001 | 4.91E-001 | 9.37E-001 | 1.11E-001 | 2.16E-001 | 4.04E-001 | 7.60E-001 | 1.00E+000 | 9.14E-001 |
| 1248 | 9 | 4 | *Helicobacter pylori kx2 npgec Lactobacillus acidophilus Staphylococcus aureus Bifidobacterium bifidum Pseudomonas aeruginosa Aeromonas cavia Helicobacter pylori kx1 npgec Pseudomonas aeruginosa fdr440 Mycobacterium tuberculosis* | 4.46E-001 | 0 | 1.00E+000 | 7.73E-001 | 5.00E-001 | 4.36E-001 | 9.42E-001 | 4.71E-001 | 1.11E-001 | 9.68E-001 | 7.06E-001 | 1.63E-001 | 1.00E+000 | 1.00E+000 |
| 36 | 5 | 114 | *Helicobacter pylori kx2 npgec Burkholderia pseudomallei Lactobacillus acidophilus Bifidobacterium bifidum Aggregatibacter actinomycetemcomitans* | 4.48E-002 | 12 | 5.43E-009 | 8.28E-001 | 5.00E-001 | 3.93E-001 | 8.69E-001 | 6.41E-001 | 3.31E-002 | 8.32E-001 | 9.05E-001 | 1.00E+000 | 4.10E-001 | 7.21E-001 |
| 156 | 7 | 37 | *Yersinia enterocolitica wap bl6 Yersinia enterocolitica wap bc Burkholderia pseudomallei Yersinia enterocolitica p60 bl6 Yersinia enterocolitica p60 bc Lactobacillus acidophilus Bifidobacterium bifidum* | 4.52E-003 | 0 | 1.00E+000 | 9.96E-001 | 4.65E-002 | 6.02E-001 | 6.88E-001 | 1.00E+000 | 6.71E-002 | 1.38E-002 | 1.92E-001 | 1.00E+000 | 1.00E+000 | 8.42E-001 |
| 1138 | 8 | 5 | *Staphylococcus aureus Listeria monocytogenes Porphyromonas gingivalis streptococcus pneumoniae d39 Helicobacter pylori kx1 npgec Ehrlichia chaffeensis liberty Pseudomonas aeruginosa fdr1 Pseudomonas aeruginosa fdr440* | 4.53E-001 | 0 | 1.00E+000 | 1.19E-001 | 9.78E-001 | 3.45E-001 | 8.54E-001 | 2.47E-002 | 4.79E-001 | 1.00E+000 | 8.68E-001 | 1.07E-001 | 5.86E-001 | 8.82E-001 |
| 1168 | 8 | 5 | *Helicobacter pylori kx2 npgec Pseudomonas aeruginosa fdr440 Listeria monocytogenes Porphyromonas gingivalis Aeromonas cavia Helicobacter pylori kx1 npgec Ehrlichia chaffeensis liberty Aggregatibacter actinomycetemcomitans* | 4.53E-001 | 0 | 1.00E+000 | 6.52E-001 | 6.52E-001 | 9.33E-001 | 2.57E-001 | 1.21E-001 | 4.79E-001 | 1.00E+000 | 2.94E-001 | 9.33E-001 | 1.58E-001 | 8.82E-001 |
| 1136 | 8 | 5 | *Helicobacter pylori kx2 npgec Burkholderia pseudomallei Lactobacillus acidophilus Staphylococcus aureus Listeria monocytogenes Helicobacter pylori kx1 npgec Ehrlichia chaffeensis liberty Escherichia coli* | 4.53E-001 | 0 | 1.00E+000 | 6.52E-001 | 6.52E-001 | 3.45E-001 | 8.54E-001 | 6.64E-001 | 8.78E-002 | 7.43E-001 | 2.94E-001 | 9.33E-001 | 1.00E+000 | 5.57E-001 |
| 1150 | 8 | 5 | *Yersinia enterocolitica wap bl6 Burkholderia pseudomallei Pseudomonas aeruginosa fdr1234 Lactobacillus acidophilus Staphylococcus aureus Bifidobacterium bifidum Porphyromonas gingivalis Aeromonas cavia* | 4.53E-001 | 0 | 1.00E+000 | 6.52E-001 | 6.52E-001 | 3.45E-001 | 8.54E-001 | 6.64E-001 | 8.78E-002 | 7.43E-001 | 8.68E-001 | 6.88E-001 | 5.86E-001 | 8.82E-001 |
| 1164 | 8 | 5 | *Yersinia enterocolitica wap bl6 Helicobacter pylori kx2 npgec Pseudomonas aeruginosa fdr1234 Porphyromonas gingivalis Aeromonas cavia Helicobacter pylori kx1 npgec Aggregatibacter actinomycetemcomitans Pseudomonas aeruginosa fdr1* | 4.53E-001 | 1 | 1.67E-001 | 6.52E-001 | 6.52E-001 | 1.00E+000 | 4.96E-002 | 2.47E-002 | 1.00E+000 | 9.50E-001 | 2.94E-001 | 6.88E-001 | 1.58E-001 | 1.00E+000 |
| 1132 | 8 | 5 | *Yersinia enterocolitica wap bl6 Burkholderia pseudomallei Lactobacillus acidophilus Staphylococcus aureus Pseudomonas aeruginosa Porphyromonas gingivalis Ehrlichia chaffeensis liberty Pseudomonas aeruginosa fdr440* | 4.53E-001 | 0 | 1.00E+000 | 3.48E-001 | 8.81E-001 | 6.88E-001 | 5.89E-001 | 6.64E-001 | 4.79E-001 | 7.43E-001 | 9.81E-001 | 3.45E-001 | 5.86E-001 | 5.57E-001 |
| 1134 | 8 | 5 | *Helicobacter pylori kx2 npgec Burkholderia pseudomallei Staphylococcus aureus Pseudomonas aeruginosa streptococcus pneumoniae d39 Helicobacter pylori kx1 npgec Ehrlichia chaffeensis liberty Mycobacterium tuberculosis* | 4.53E-001 | 0 | 1.00E+000 | 3.48E-001 | 8.81E-001 | 6.88E-001 | 8.54E-001 | 3.51E-001 | 1.00E+000 | 7.43E-001 | 8.68E-001 | 1.07E-001 | 1.00E+000 | 5.57E-001 |
| 638 | 6 | 14 | *Burkholderia pseudomallei Pseudomonas aeruginosa Mycobacterium tuberculosis streptococcus pneumoniae d39 Escherichia coli Aggregatibacter actinomycetemcomitans* | 4.54E-001 | 1 | 6.35E-001 | 1.03E-002 | 1.00E+000 | 8.60E-001 | 7.84E-001 | 7.55E-001 | 1.00E+000 | 2.16E-001 | 9.44E-001 | 1.73E-001 | 4.74E-001 | 7.89E-001 |
| 632 | 6 | 14 | *Yersinia enterocolitica wap bl6 Helicobacter pylori kx2 npgec Yersinia enterocolitica p60 bl6 Yersinia enterocolitica p60 bc Pseudomonas aeruginosa Aggregatibacter actinomycetemcomitans* | 4.54E-001 | 1 | 6.35E-001 | 9.08E-001 | 3.31E-001 | 1.00E+000 | 1.13E-001 | 7.55E-001 | 1.00E+000 | 2.16E-001 | 1.08E-001 | 8.60E-001 | 4.74E-001 | 1.00E+000 |
| 626 | 6 | 14 | *Yersinia enterocolitica wap bl6 Burkholderia pseudomallei Lactobacillus acidophilus Porphyromonas gingivalis Mycobacterium tuberculosis streptococcus pneumoniae d39* | 4.54E-001 | 0 | 1.00E+000 | 3.31E-001 | 9.08E-001 | 5.03E-001 | 9.54E-001 | 7.55E-001 | 3.78E-001 | 2.16E-001 | 9.44E-001 | 5.03E-001 | 4.74E-001 | 7.89E-001 |
| 620 | 6 | 14 | *Burkholderia pseudomallei Pseudomonas aeruginosa fdr1 Lactobacillus acidophilus Bifidobacterium bifidum Porphyromonas gingivalis Ehrlichia chaffeensis liberty* | 4.54E-001 | 2 | 2.16E-002 | 6.69E-001 | 6.69E-001 | 5.03E-001 | 7.84E-001 | 7.55E-001 | 4.88E-002 | 8.87E-001 | 1.00E+000 | 8.60E-001 | 4.74E-001 | 3.82E-001 |
| 622 | 6 | 14 | *Burkholderia pseudomallei Ehrlichia chaffeensis wakulla Lactobacillus acidophilus Bifidobacterium bifidum Helicobacter pylori kx2 mgep Helicobacter pylori kx1 npgec* | 4.54E-001 | 4 | 2.86E-002 | 9.90E-001 | 9.18E-002 | 5.03E-001 | 7.84E-001 | 7.55E-001 | 4.88E-002 | 8.87E-001 | 7.13E-001 | 1.00E+000 | 1.00E+000 | 3.82E-001 |
| 624 | 6 | 14 | *Yersinia enterocolitica wap bl6 Helicobacter pylori kx2 npgec Yersinia enterocolitica p60 bc Pseudomonas aeruginosa Helicobacter pylori kx1 npgec Escherichia coli* | 4.54E-001 | 5 | 5.33E-002 | 9.08E-001 | 3.31E-001 | 1.00E+000 | 1.13E-001 | 7.55E-001 | 1.00E+000 | 2.16E-001 | 1.64E-002 | 8.60E-001 | 1.00E+000 | 1.00E+000 |
| 636 | 6 | 14 | *Yersinia enterocolitica wap bl6 Helicobacter pylori kx2 npgec Staphylococcus aureus Porphyromonas gingivalis Fusobacterium nucleatum Helicobacter pylori kx1 npgec* | 4.54E-001 | 0 | 1.00E+000 | 6.69E-001 | 6.69E-001 | 8.60E-001 | 4.39E-001 | 2.32E-002 | 1.00E+000 | 8.87E-001 | 3.61E-001 | 8.60E-001 | 9.10E-002 | 1.00E+000 |
| 260 | 5 | 35 | *Helicobacter pylori kx2 npgec Burkholderia pseudomallei Lactobacillus acidophilus Listeria monocytogenes Helicobacter pylori kx1 npgec* | 4.57E-001 | 0 | 1.00E+000 | 8.28E-001 | 5.00E-001 | 3.93E-001 | 8.69E-001 | 6.41E-001 | 3.31E-002 | 8.32E-001 | 2.35E-001 | 1.00E+000 | 1.00E+000 | 7.21E-001 |
| 88 | 4 | 90 | *Lactobacillus acidophilus Bifidobacterium bifidum Mycobacterium tuberculosis Aeromonas cavia* | 4.76E-001 | 1 | 9.84E-001 | 9.47E-001 | 3.03E-001 | 2.77E-001 | 9.96E-001 | 1.00E+000 | 2.02E-002 | 7.55E-001 | 8.43E-001 | 7.19E-001 | 1.00E+000 | 1.00E+000 |
| 96 | 4 | 85 | *Yersinia enterocolitica p60 bc Lactobacillus acidophilus Staphylococcus aureus Porphyromonas gingivalis* | 4.76E-001 | 32 | 1.10E-003 | 6.97E-001 | 6.97E-001 | 2.77E-001 | 9.37E-001 | 4.95E-001 | 2.65E-001 | 7.55E-001 | 8.43E-001 | 7.19E-001 | 3.41E-001 | 1.00E+000 |
| 342 | 7 | 21 | *Yersinia enterocolitica wap bl6 Helicobacter pylori kx2 npgec Burkholderia pseudomallei Yersinia enterocolitica p60 bl6 Yersinia enterocolitica p60 bc Helicobacter pylori kx1 npgec Ehrlichia chaffeensis liberty* | 4.83E-002 | 18 | 1.61E-010 | 9.96E-001 | 4.65E-002 | 1.00E+000 | 7.55E-002 | 8.39E-001 | 1.00E+000 | 8.83E-002 | 4.43E-002 | 1.00E+000 | 1.00E+000 | 4.72E-001 |
| 346 | 7 | 21 | *Helicobacter pylori kx2 npgec Lactobacillus acidophilus Bifidobacterium bifidum Pseudomonas aeruginosa Porphyromonas gingivalis Mycobacterium tuberculosis Helicobacter pylori kx1 npgec* | 4.83E-002 | 2 | 4.07E-002 | 7.95E-001 | 5.00E-001 | 6.02E-001 | 9.12E-001 | 5.46E-001 | 6.71E-002 | 9.25E-001 | 8.03E-001 | 6.02E-001 | 5.32E-001 | 1.00E+000 |
| 338 | 7 | 21 | *Burkholderia pseudomallei Staphylococcus aureus Bifidobacterium bifidum Porphyromonas gingivalis Helicobacter pylori kx1 npgec Escherichia coli Aggregatibacter actinomycetemcomitans* | 4.83E-002 | 0 | 1.00E+000 | 2.05E-001 | 9.53E-001 | 6.02E-001 | 6.88E-001 | 2.36E-001 | 4.30E-001 | 6.60E-001 | 8.03E-001 | 9.03E-001 | 1.23E-001 | 8.42E-001 |
| 344 | 7 | 21 | *Helicobacter pylori kx2 npgec Lactobacillus acidophilus Staphylococcus aureus Bifidobacterium bifidum Pseudomonas aeruginosa Helicobacter pylori kx2 mgep Helicobacter pylori kx1 npgec* | 4.83E-002 | 3 | 5.03E-002 | 9.53E-001 | 2.05E-001 | 2.56E-001 | 9.12E-001 | 2.36E-001 | 6.71E-002 | 1.00E+000 | 4.88E-001 | 6.02E-001 | 1.00E+000 | 1.00E+000 |
| 336 | 7 | 21 | *Yersinia enterocolitica wap bl6 Helicobacter pylori kx2 npgec Staphylococcus aureus Pseudomonas aeruginosa Porphyromonas gingivalis Aeromonas cavia Escherichia coli* | 4.83E-002 | 0 | 1.00E+000 | 5.00E-001 | 7.95E-001 | 9.03E-001 | 3.40E-001 | 5.46E-001 | 1.00E+000 | 6.60E-001 | 1.92E-001 | 6.02E-001 | 5.32E-001 | 1.00E+000 |
| 276 | 5 | 33 | *Pseudomonas aeruginosa Mycobacterium tuberculosis Aeromonas cavia streptococcus pneumoniae d39 Helicobacter pylori kx1 npgec* | 4.83E-001 | 4 | 1.88E-001 | 5.00E-001 | 8.28E-001 | 8.00E-001 | 8.69E-001 | 6.41E-001 | 1.00E+000 | 8.32E-001 | 5.96E-001 | 1.03E-001 | 1.00E+000 | 1.00E+000 |
| 686 | 6 | 13 | *Helicobacter pylori kx2 npgec Burkholderia pseudomallei Staphylococcus aureus Pseudomonas aeruginosa Fusobacterium nucleatum streptococcus pneumoniae d39* | 4.87E-001 | 0 | 1.00E+000 | 9.18E-002 | 9.90E-001 | 5.03E-001 | 7.84E-001 | 1.36E-001 | 1.00E+000 | 8.87E-001 | 9.44E-001 | 1.73E-001 | 4.74E-001 | 7.89E-001 |
| 682 | 6 | 13 | *Yersinia enterocolitica p60 bc Lactobacillus acidophilus Bifidobacterium bifidum Pseudomonas aeruginosa Mycobacterium tuberculosis Helicobacter pylori kx1 npgec* | 4.87E-001 | 0 | 1.00E+000 | 9.08E-001 | 3.31E-001 | 5.03E-001 | 9.54E-001 | 9.56E-001 | 4.88E-002 | 5.61E-001 | 7.13E-001 | 5.03E-001 | 1.00E+000 | 1.00E+000 |
| 680 | 6 | 13 | *Helicobacter pylori kx2 npgec Burkholderia pseudomallei Yersinia enterocolitica p60 bc Bifidobacterium bifidum Mycobacterium tuberculosis streptococcus pneumoniae d39* | 4.87E-001 | 0 | 1.00E+000 | 6.69E-001 | 6.69E-001 | 5.03E-001 | 9.54E-001 | 7.55E-001 | 3.78E-001 | 2.16E-001 | 7.13E-001 | 5.03E-001 | 1.00E+000 | 7.89E-001 |
| 286 | 5 | 32 | *Pseudomonas aeruginosa Porphyromonas gingivalis Helicobacter pylori kx2 mgep Aeromonas cavia Aggregatibacter actinomycetemcomitans* | 4.97E-001 | 1 | 5.19E-001 | 5.00E-001 | 8.28E-001 | 1.00E+000 | 1.68E-001 | 2.75E-001 | 1.00E+000 | 1.00E+000 | 5.96E-001 | 8.00E-001 | 6.29E-002 | 1.00E+000 |
| 284 | 5 | 32 | *Burkholderia pseudomallei Porphyromonas gingivalis Helicobacter pylori kx2 mgep Helicobacter pylori kx1 npgec Aggregatibacter actinomycetemcomitans* | 4.97E-001 | 10 | 6.18E-006 | 5.00E-001 | 8.28E-001 | 1.00E+000 | 1.68E-001 | 6.08E-002 | 1.00E+000 | 8.32E-001 | 5.96E-001 | 1.00E+000 | 6.29E-002 | 7.21E-001 |
| 302 | 5 | 31 | *Yersinia enterocolitica wap bl6 Yersinia enterocolitica p60 bl6 Yersinia enterocolitica p60 bc Staphylococcus aureus Pseudomonas aeruginosa* | 5.08E-001 | 14 | 4.48E-003 | 8.28E-001 | 5.00E-001 | 8.00E-001 | 5.54E-001 | 9.23E-001 | 1.00E+000 | 1.31E-001 | 2.35E-001 | 3.93E-001 | 1.00E+000 | 1.00E+000 |
| 666 | 8 | 10 | *Burkholderia pseudomallei Lactobacillus acidophilus Staphylococcus aureus Bifidobacterium bifidum Pseudomonas aeruginosa Helicobacter pylori kx1 npgec Mycobacterium tuberculosis Escherichia coli* | 5.10E-002 | 0 | 1.00E+000 | 3.48E-001 | 8.81E-001 | 3.45E-001 | 9.69E-001 | 8.98E-001 | 8.78E-002 | 4.11E-001 | 8.68E-001 | 3.45E-001 | 1.00E+000 | 8.82E-001 |
| 114 | 4 | 78 | *Yersinia enterocolitica p60 bl6 Bifidobacterium bifidum Porphyromonas gingivalis Aggregatibacter actinomycetemcomitans* | 5.16E-001 | 8 | 3.20E-001 | 6.97E-001 | 6.97E-001 | 7.19E-001 | 6.80E-001 | 4.95E-001 | 2.65E-001 | 7.55E-001 | 8.43E-001 | 1.00E+000 | 3.91E-002 | 1.00E+000 |
| 1110 | 7 | 6 | *Yersinia enterocolitica wap bl6 Helicobacter pylori kx2 npgec Pseudomonas aeruginosa fdr1 Pseudomonas aeruginosa Helicobacter pylori kx2 mgep Helicobacter pylori kx1 npgec Ehrlichia chaffeensis liberty* | 5.18E-001 | 0 | 1.00E+000 | 9.53E-001 | 2.05E-001 | 1.00E+000 | 7.55E-002 | 2.36E-001 | 1.00E+000 | 9.25E-001 | 1.92E-001 | 6.02E-001 | 1.00E+000 | 8.42E-001 |
| 1090 | 7 | 6 | *Yersinia enterocolitica wap bl6 Staphylococcus aureus Pseudomonas aeruginosa Porphyromonas gingivalis Mycobacterium tuberculosis Helicobacter pylori kx1 npgec Ehrlichia chaffeensis liberty* | 5.18E-001 | 0 | 1.00E+000 | 5.00E-001 | 7.95E-001 | 9.03E-001 | 6.88E-001 | 5.46E-001 | 1.00E+000 | 6.60E-001 | 8.03E-001 | 2.56E-001 | 5.32E-001 | 8.42E-001 |
| 1108 | 7 | 6 | *Yersinia enterocolitica wap bl6 Burkholderia pseudomallei Pseudomonas aeruginosa fdr440 Pseudomonas aeruginosa Porphyromonas gingivalis Aeromonas cavia Escherichia coli* | 5.18E-001 | 0 | 1.00E+000 | 2.05E-001 | 9.53E-001 | 1.00E+000 | 7.55E-002 | 8.39E-001 | 1.00E+000 | 3.12E-001 | 4.88E-001 | 6.02E-001 | 5.32E-001 | 8.42E-001 |
| 1106 | 7 | 6 | *Yersinia enterocolitica wap bl6 Helicobacter pylori kx2 npgec Lactobacillus acidophilus Pseudomonas aeruginosa Mycobacterium tuberculosis Aeromonas cavia Escherichia coli* | 5.18E-001 | 0 | 1.00E+000 | 7.95E-001 | 5.00E-001 | 9.03E-001 | 6.88E-001 | 9.76E-001 | 4.30E-001 | 3.12E-001 | 1.92E-001 | 6.02E-001 | 1.00E+000 | 1.00E+000 |
| 1116 | 7 | 6 | *Burkholderia pseudomallei Pseudomonas aeruginosa fdr440 Listeria monocytogenes Pseudomonas aeruginosa Porphyromonas gingivalis Mycobacterium tuberculosis Helicobacter pylori kx1 npgec* | 5.18E-001 | 0 | 1.00E+000 | 4.65E-002 | 9.96E-001 | 9.03E-001 | 6.88E-001 | 5.46E-001 | 4.30E-001 | 6.60E-001 | 8.03E-001 | 2.56E-001 | 5.32E-001 | 8.42E-001 |
| 1086 | 7 | 6 | *Yersinia enterocolitica wap bl6 Helicobacter pylori kx2 npgec Yersinia enterocolitica p60 bl6 Pseudomonas aeruginosa Porphyromonas gingivalis streptococcus pneumoniae d39 Helicobacter pylori kx1 npgec* | 5.18E-001 | 0 | 1.00E+000 | 7.95E-001 | 5.00E-001 | 9.03E-001 | 3.40E-001 | 2.36E-001 | 1.00E+000 | 6.60E-001 | 1.92E-001 | 6.02E-001 | 5.32E-001 | 1.00E+000 |
| 1102 | 7 | 6 | *Helicobacter pylori kx2 npgec Burkholderia pseudomallei streptococcus gordonii Staphylococcus aureus Pseudomonas aeruginosa Helicobacter pylori kx1 npgec Escherichia coli* | 5.18E-001 | 0 | 1.00E+000 | 2.05E-001 | 9.53E-001 | 6.02E-001 | 6.88E-001 | 2.36E-001 | 1.00E+000 | 6.60E-001 | 4.88E-001 | 6.02E-001 | 5.32E-001 | 8.42E-001 |
| 1082 | 7 | 6 | *Yersinia enterocolitica wap bl6 Yersinia enterocolitica p60 bc Staphylococcus aureus Pseudomonas aeruginosa Porphyromonas gingivalis Escherichia coli Aggregatibacter actinomycetemcomitans* | 5.18E-001 | 0 | 1.00E+000 | 2.05E-001 | 9.53E-001 | 9.03E-001 | 3.40E-001 | 5.46E-001 | 1.00E+000 | 3.12E-001 | 4.88E-001 | 6.02E-001 | 1.23E-001 | 1.00E+000 |
| 1120 | 7 | 6 | *Helicobacter pylori kx2 npgec Pseudomonas aeruginosa fdr1234 Staphylococcus aureus Pseudomonas aeruginosa Porphyromonas gingivalis Helicobacter pylori kx1 npgec Aggregatibacter actinomycetemcomitans* | 5.18E-001 | 0 | 1.00E+000 | 2.05E-001 | 9.53E-001 | 9.03E-001 | 3.40E-001 | 8.14E-003 | 1.00E+000 | 1.00E+000 | 8.03E-001 | 2.56E-001 | 1.23E-001 | 1.00E+000 |
| 1094 | 7 | 6 | *Yersinia enterocolitica wap bl6 Yersinia enterocolitica p60 bl6 Yersinia enterocolitica p60 bc Pseudomonas aeruginosa Porphyromonas gingivalis Aeromonas cavia Ehrlichia chaffeensis liberty* | 5.18E-001 | 0 | 1.00E+000 | 9.53E-001 | 2.05E-001 | 1.00E+000 | 7.55E-002 | 9.76E-001 | 1.00E+000 | 3.12E-001 | 1.92E-001 | 9.03E-001 | 5.32E-001 | 8.42E-001 |
| 1100 | 7 | 6 | *Yersinia enterocolitica wap bl6 Yersinia enterocolitica p60 bl6 Yersinia enterocolitica p60 bc Lactobacillus acidophilus Listeria monocytogenes Bifidobacterium bifidum Aeromonas cavia* | 5.18E-001 | 2 | 2.16E-002 | 9.96E-001 | 4.65E-002 | 2.56E-001 | 9.12E-001 | 1.00E+000 | 3.05E-003 | 3.12E-001 | 4.43E-002 | 1.00E+000 | 1.00E+000 | 1.00E+000 |
| 722 | 6 | 12 | *Yersinia enterocolitica p60 bc Lactobacillus acidophilus Pseudomonas aeruginosa fdr440 Bifidobacterium bifidum Mycobacterium tuberculosis Escherichia coli* | 5.23E-001 | 0 | 1.00E+000 | 6.69E-001 | 6.69E-001 | 5.03E-001 | 9.54E-001 | 9.56E-001 | 4.88E-002 | 2.16E-001 | 7.13E-001 | 5.03E-001 | 1.00E+000 | 1.00E+000 |
| 716 | 6 | 12 | *Yersinia enterocolitica wap bl6 Helicobacter pylori kx2 npgec Yersinia enterocolitica p60 bc Pseudomonas aeruginosa fdr1 Helicobacter pylori kx1 npgec Escherichia coli* | 5.23E-001 | 5 | 1.87E-001 | 9.08E-001 | 3.31E-001 | 1.00E+000 | 1.13E-001 | 4.13E-001 | 1.00E+000 | 2.16E-001 | 1.64E-002 | 8.60E-001 | 1.00E+000 | 1.00E+000 |
| 734 | 6 | 12 | *Helicobacter pylori kx2 npgec Lactobacillus acidophilus Fusobacterium nucleatum streptococcus pneumoniae d39 Helicobacter pylori kx1 npgec Aggregatibacter actinomycetemcomitans* | 5.23E-001 | 0 | 1.00E+000 | 6.69E-001 | 6.69E-001 | 5.03E-001 | 7.84E-001 | 2.32E-002 | 3.78E-001 | 1.00E+000 | 7.13E-001 | 8.60E-001 | 9.10E-002 | 1.00E+000 |
| 724 | 6 | 12 | *Yersinia enterocolitica p60 bl6 Yersinia enterocolitica p60 bc Staphylococcus aureus Porphyromonas gingivalis Mycobacterium tuberculosis streptococcus pneumoniae d39* | 5.23E-001 | 0 | 1.00E+000 | 3.31E-001 | 9.08E-001 | 5.03E-001 | 9.54E-001 | 4.13E-001 | 1.00E+000 | 2.16E-001 | 7.13E-001 | 1.73E-001 | 4.74E-001 | 1.00E+000 |
| 736 | 6 | 12 | *Burkholderia pseudomallei Lactobacillus acidophilus Bifidobacterium bifidum Pseudomonas aeruginosa Fusobacterium nucleatum Aeromonas cavia* | 5.23E-001 | 0 | 1.00E+000 | 6.69E-001 | 6.69E-001 | 5.03E-001 | 7.84E-001 | 9.56E-001 | 4.88E-002 | 8.87E-001 | 9.44E-001 | 8.60E-001 | 4.74E-001 | 7.89E-001 |
| 730 | 6 | 12 | *Burkholderia pseudomallei Pseudomonas aeruginosa fdr440 Listeria monocytogenes Mycobacterium tuberculosis Escherichia coli Ehrlichia chaffeensis liberty* | 5.23E-001 | 1 | 7.23E-001 | 9.18E-002 | 9.90E-001 | 8.60E-001 | 7.84E-001 | 9.56E-001 | 3.78E-001 | 2.16E-001 | 7.13E-001 | 5.03E-001 | 1.00E+000 | 3.82E-001 |
| 738 | 6 | 12 | *streptococcus gordonii Porphyromonas gingivalis Mycobacterium tuberculosis Fusobacterium nucleatum Aeromonas cavia streptococcus pneumoniae d39* | 5.23E-001 | 0 | 1.00E+000 | 9.18E-002 | 9.90E-001 | 5.03E-001 | 9.54E-001 | 1.36E-001 | 1.00E+000 | 8.87E-001 | 9.44E-001 | 5.03E-001 | 6.57E-003 | 1.00E+000 |
| 326 | 5 | 30 | *Burkholderia pseudomallei streptococcus gordonii Pseudomonas aeruginosa streptococcus pneumoniae d39 Helicobacter pylori kx1 npgec* | 5.23E-001 | 0 | 1.00E+000 | 1.72E-001 | 9.76E-001 | 3.93E-001 | 8.69E-001 | 2.75E-001 | 1.00E+000 | 8.32E-001 | 9.05E-001 | 3.93E-001 | 4.10E-001 | 7.21E-001 |
| 14 | 3 | 253 | *Burkholderia pseudomallei Lactobacillus acidophilus Porphyromonas gingivalis* | 5.23E-001 | 85 | < 1.67E-026 | 5.00E-001 | 8.84E-001 | 6.08E-001 | 8.08E-001 | 7.74E-001 | 2.04E-001 | 6.46E-001 | 1.00E+000 | 1.00E+000 | 2.65E-001 | 5.25E-001 |
| 16 | 3 | 237 | *Burkholderia pseudomallei Bifidobacterium bifidum Aeromonas cavia* | 5.35E-001 | 74 | < 1.67E-026 | 8.84E-001 | 5.00E-001 | 6.08E-001 | 8.08E-001 | 1.00E+000 | 2.04E-001 | 6.46E-001 | 7.45E-001 | 1.00E+000 | 1.00E+000 | 5.25E-001 |
| 18 | 3 | 237 | *Helicobacter pylori kx2 npgec Helicobacter pylori kx1 npgec Escherichia coli* | 5.35E-001 | 115 | 3.83E-011 | 8.84E-001 | 5.00E-001 | 1.00E+000 | 3.54E-001 | 3.21E-001 | 1.00E+000 | 6.46E-001 | 3.96E-002 | 1.00E+000 | 1.00E+000 | 1.00E+000 |
| 140 | 6 | 46 | *Helicobacter pylori kx2 npgec Burkholderia pseudomallei Staphylococcus aureus Bifidobacterium bifidum Pseudomonas aeruginosa Aeromonas cavia* | 5.43E-002 | 5 | 2.39E-003 | 6.69E-001 | 6.69E-001 | 5.03E-001 | 7.84E-001 | 7.55E-001 | 3.78E-001 | 8.87E-001 | 7.13E-001 | 5.03E-001 | 1.00E+000 | 7.89E-001 |
| 118 | 4 | 77 | *Helicobacter pylori kx2 npgec Helicobacter pylori kx1 mgep Helicobacter pylori kx2 mgep Helicobacter pylori kx1 npgec* | 5.46E-001 | 98 | < 1.67E-026 | 1.00E+000 | 5.35E-002 | 1.00E+000 | 2.45E-001 | 1.63E-002 | 1.00E+000 | 1.00E+000 | 1.22E-002 | 1.00E+000 | 1.00E+000 | 1.00E+000 |
| 120 | 4 | 76 | *Lactobacillus acidophilus Porphyromonas gingivalis Mycobacterium tuberculosis Aggregatibacter actinomycetemcomitans* | 5.46E-001 | 8 | 6.08E-002 | 3.03E-001 | 9.47E-001 | 7.19E-001 | 9.37E-001 | 4.95E-001 | 2.65E-001 | 7.55E-001 | 1.00E+000 | 7.19E-001 | 3.91E-002 | 1.00E+000 |
| 364 | 7 | 20 | *Helicobacter pylori kx2 npgec Burkholderia pseudomallei Pseudomonas aeruginosa fdr1 Bifidobacterium bifidum Porphyromonas gingivalis Helicobacter pylori kx2 mgep Helicobacter pylori kx1 npgec* | 5.60E-002 | 3 | 6.47E-002 | 7.95E-001 | 5.00E-001 | 9.03E-001 | 3.40E-001 | 6.08E-002 | 4.30E-001 | 9.25E-001 | 4.88E-001 | 9.03E-001 | 5.32E-001 | 8.42E-001 |
| 360 | 7 | 20 | *Yersinia enterocolitica wap bl6 Helicobacter pylori kx2 npgec Ehrlichia chaffeensis arkansa Lactobacillus acidophilus Bifidobacterium bifidum Porphyromonas gingivalis Helicobacter pylori kx1 npgec* | 5.60E-002 | 0 | 1.00E+000 | 9.96E-001 | 4.65E-002 | 6.02E-001 | 6.88E-001 | 5.46E-001 | 6.71E-002 | 9.25E-001 | 4.88E-001 | 1.00E+000 | 5.32E-001 | 8.42E-001 |
| 778 | 6 | 11 | *Yersinia enterocolitica wap bl6 Yersinia enterocolitica p60 bl6 Yersinia enterocolitica p60 bc Porphyromonas gingivalis Helicobacter pylori kx2 mgep Aggregatibacter actinomycetemcomitans* | 5.62E-001 | 5 | 6.20E-002 | 9.08E-001 | 3.31E-001 | 1.00E+000 | 1.13E-001 | 4.13E-001 | 1.00E+000 | 2.16E-001 | 1.08E-001 | 1.00E+000 | 9.10E-002 | 1.00E+000 |
| 776 | 6 | 11 | *Helicobacter pylori kx2 npgec Burkholderia pseudomallei Pseudomonas aeruginosa fdr440 Helicobacter pylori kx2 mgep Aeromonas cavia Helicobacter pylori kx1 npgec* | 5.62E-001 | 0 | 1.00E+000 | 9.08E-001 | 3.31E-001 | 1.00E+000 | 1.13E-001 | 1.36E-001 | 1.00E+000 | 8.87E-001 | 1.08E-001 | 8.60E-001 | 1.00E+000 | 7.89E-001 |
| 774 | 6 | 11 | *Helicobacter pylori kx2 npgec Burkholderia pseudomallei streptococcus gordonii Lactobacillus acidophilus Porphyromonas gingivalis Helicobacter pylori kx1 npgec* | 5.62E-001 | 0 | 1.00E+000 | 6.69E-001 | 6.69E-001 | 5.03E-001 | 7.84E-001 | 1.36E-001 | 3.78E-001 | 8.87E-001 | 7.13E-001 | 1.00E+000 | 9.10E-002 | 7.89E-001 |
| 378 | 5 | 27 | *Staphylococcus aureus Listeria monocytogenes Porphyromonas gingivalis Mycobacterium tuberculosis Helicobacter pylori kx1 npgec* | 5.67E-001 | 0 | 1.00E+000 | 1.72E-001 | 9.76E-001 | 3.93E-001 | 9.82E-001 | 2.75E-001 | 3.23E-001 | 8.32E-001 | 5.96E-001 | 3.93E-001 | 4.10E-001 | 1.00E+000 |
| 1398 | 10 | 3 | *Yersinia enterocolitica wap bl6 Helicobacter pylori kx2 npgec Burkholderia pseudomallei Lactobacillus acidophilus Bifidobacterium bifidum Aeromonas cavia Helicobacter pylori kx1 npgec Ehrlichia chaffeensis wakulla Pseudomonas aeruginosa fdr1 Mycobacterium tuberculosis* | 5.72E-001 | 0 | 1.00E+000 | 9.67E-001 | 1.39E-001 | 8.19E-001 | 7.04E-001 | 8.35E-001 | 1.36E-001 | 6.00E-001 | 5.13E-001 | 8.19E-001 | 1.00E+000 | 7.01E-001 |
| 1374 | 10 | 3 | *Helicobacter pylori kx2 npgec Yersinia enterocolitica p60 bl6 Yersinia enterocolitica p60 bc Eubacterium rectale Lactobacillus acidophilus Staphylococcus aureus Bifidobacterium bifidum Porphyromonas gingivalis Aeromonas cavia Aggregatibacter actinomycetemcomitans* | 5.72E-001 | 0 | 1.00E+000 | 9.67E-001 | 1.39E-001 | 2.29E-001 | 9.04E-001 | 5.85E-001 | 1.36E-001 | 8.63E-001 | 2.39E-001 | 9.70E-001 | 2.36E-001 | 1.00E+000 |
| 1352 | 10 | 3 | *Helicobacter pylori kx2 npgec Burkholderia pseudomallei Lactobacillus acidophilus Staphylococcus aureus Bifidobacterium bifidum Porphyromonas gingivalis Aeromonas cavia Helicobacter pylori kx1 npgec Ehrlichia chaffeensis liberty Ehrlichia chaffeensis wakulla* | 5.72E-001 | 2 | 1.20E-001 | 9.67E-001 | 1.39E-001 | 5.24E-001 | 7.04E-001 | 5.85E-001 | 1.36E-001 | 9.80E-001 | 7.88E-001 | 9.70E-001 | 6.79E-001 | 3.62E-001 |
| 1344 | 10 | 3 | *Yersinia enterocolitica wap bl6 Helicobacter pylori kx2 npgec Burkholderia pseudomallei Yersinia enterocolitica p60 bl6 Yersinia enterocolitica p60 bc Lactobacillus acidophilus Bifidobacterium bifidum Helicobacter pylori kx1 npgec Helicobacter pylori kx2 mgep Escherichia coli* | 5.72E-001 | 3 | 3.76E-002 | 9.96E-001 | 3.35E-002 | 8.19E-001 | 4.00E-001 | 8.35E-001 | 1.36E-001 | 9.62E-002 | 1.44E-002 | 1.00E+000 | 1.00E+000 | 9.37E-001 |
| 1358 | 10 | 3 | *Yersinia enterocolitica wap bl6 Helicobacter pylori kx2 npgec Burkholderia pseudomallei Yersinia enterocolitica p60 bl6 Lactobacillus acidophilus Bifidobacterium bifidum Pseudomonas aeruginosa Helicobacter pylori kx1 npgec Ehrlichia chaffeensis liberty Mycobacterium tuberculosis* | 5.72E-001 | 0 | 1.00E+000 | 9.67E-001 | 1.39E-001 | 8.19E-001 | 7.04E-001 | 9.62E-001 | 1.36E-001 | 2.96E-001 | 5.13E-001 | 8.19E-001 | 1.00E+000 | 7.01E-001 |
| 1346 | 10 | 3 | *Yersinia enterocolitica wap bl6 Helicobacter pylori kx2 npgec Burkholderia pseudomallei Yersinia enterocolitica p60 bc Porphyromonas gingivalis Helicobacter pylori kx1 npgec Ehrlichia chaffeensis liberty Pseudomonas aeruginosa fdr1 Helicobacter pylori kx2 mgep Escherichia coli* | 5.72E-001 | 0 | 1.00E+000 | 8.61E-001 | 3.59E-001 | 1.00E+000 | 2.04E-002 | 3.00E-001 | 1.00E+000 | 2.96E-001 | 7.41E-002 | 9.70E-001 | 6.79E-001 | 7.01E-001 |
| 1392 | 10 | 3 | *Helicobacter pylori kx2 npgec Staphylococcus aureus Bifidobacterium bifidum Pseudomonas aeruginosa Aeromonas cavia streptococcus pneumoniae d39 Helicobacter pylori kx1 npgec Ehrlichia chaffeensis liberty Ehrlichia chaffeensis arkansa Ehrlichia chaffeensis wakulla* | 5.72E-001 | 0 | 1.00E+000 | 9.67E-001 | 1.39E-001 | 5.24E-001 | 7.04E-001 | 5.85E-001 | 5.68E-001 | 1.00E+000 | 7.88E-001 | 5.24E-001 | 1.00E+000 | 3.62E-001 |
| 1394 | 10 | 3 | *Yersinia enterocolitica wap bl6 Helicobacter pylori kx2 npgec Staphylococcus aureus Bifidobacterium bifidum Porphyromonas gingivalis Fusobacterium nucleatum streptococcus pneumoniae d39 Helicobacter pylori kx1 npgec Ehrlichia chaffeensis wakulla streptococcus gordonii* | 5.72E-001 | 0 | 1.00E+000 | 6.41E-001 | 6.41E-001 | 2.29E-001 | 9.04E-001 | 2.33E-002 | 5.68E-001 | 9.80E-001 | 7.88E-001 | 8.19E-001 | 3.62E-002 | 9.37E-001 |
| 1376 | 10 | 3 | *Burkholderia pseudomallei Lactobacillus acidophilus Staphylococcus aureus Pseudomonas aeruginosa Porphyromonas gingivalis streptococcus pneumoniae d39 Aggregatibacter actinomycetemcomitans Pseudomonas aeruginosa fdr440 Mycobacterium tuberculosis Escherichia coli* | 5.72E-001 | 0 | 1.00E+000 | 4.43E-003 | 1.00E+000 | 5.24E-001 | 9.04E-001 | 3.00E-001 | 5.68E-001 | 6.00E-001 | 9.94E-001 | 6.43E-002 | 2.36E-001 | 9.37E-001 |
| 1340 | 10 | 3 | *Burkholderia pseudomallei Lactobacillus acidophilus Bifidobacterium bifidum Pseudomonas aeruginosa Helicobacter pylori kx1 npgec Ehrlichia chaffeensis liberty Aggregatibacter actinomycetemcomitans Pseudomonas aeruginosa fdr1 Pseudomonas aeruginosa fdr440 Escherichia coli* | 5.72E-001 | 0 | 1.00E+000 | 3.59E-001 | 8.61E-001 | 8.19E-001 | 4.00E-001 | 5.85E-001 | 1.36E-001 | 8.63E-001 | 9.46E-001 | 5.24E-001 | 6.79E-001 | 7.01E-001 |
| 32 | 3 | 204 | *Burkholderia pseudomallei Bifidobacterium bifidum Mycobacterium tuberculosis* | 5.74E-001 | 19 | 5.20E-002 | 5.00E-001 | 8.84E-001 | 6.08E-001 | 9.81E-001 | 1.00E+000 | 2.04E-001 | 1.92E-001 | 1.00E+000 | 6.08E-001 | 1.00E+000 | 5.25E-001 |
| 24 | 3 | 221 | *Helicobacter pylori kx2 npgec Lactobacillus acidophilus Aeromonas cavia* | 5.77E-001 | 69 | < 1.67E-026 | 1.00E+000 | 1.16E-001 | 6.08E-001 | 8.08E-001 | 7.74E-001 | 2.04E-001 | 1.00E+000 | 2.87E-001 | 1.00E+000 | 1.00E+000 | 1.00E+000 |
| 386 | 5 | 26 | *Burkholderia pseudomallei Pseudomonas aeruginosa fdr440 Porphyromonas gingivalis Mycobacterium tuberculosis Aggregatibacter actinomycetemcomitans* | 5.82E-001 | 0 | 1.00E+000 | 2.39E-002 | 1.00E+000 | 1.00E+000 | 5.54E-001 | 2.75E-001 | 1.00E+000 | 4.46E-001 | 1.00E+000 | 3.93E-001 | 6.29E-002 | 7.21E-001 |
| 384 | 5 | 26 | *Burkholderia pseudomallei Lactobacillus acidophilus Staphylococcus aureus Mycobacterium tuberculosis streptococcus pneumoniae d39* | 5.82E-001 | 0 | 1.00E+000 | 1.72E-001 | 9.76E-001 | 1.03E-001 | 9.99E-001 | 6.41E-001 | 3.23E-001 | 4.46E-001 | 1.00E+000 | 1.03E-001 | 1.00E+000 | 7.21E-001 |
| 38 | 5 | 108 | *Helicobacter pylori kx2 npgec Burkholderia pseudomallei Lactobacillus acidophilus Helicobacter pylori kx2 mgep Helicobacter pylori kx1 npgec* | 5.84E-002 | 24 | 1.61E-007 | 9.76E-001 | 1.72E-001 | 8.00E-001 | 5.54E-001 | 2.75E-001 | 3.23E-001 | 8.32E-001 | 2.35E-001 | 1.00E+000 | 1.00E+000 | 7.21E-001 |
| 410 | 5 | 25 | *Burkholderia pseudomallei Lactobacillus acidophilus Porphyromonas gingivalis Helicobacter pylori kx2 mgep Escherichia coli* | 5.98E-001 | 6 | 3.14E-002 | 5.00E-001 | 8.28E-001 | 8.00E-001 | 5.54E-001 | 6.41E-001 | 3.23E-001 | 4.46E-001 | 5.96E-001 | 1.00E+000 | 4.10E-001 | 7.21E-001 |
| 416 | 5 | 25 | *Yersinia enterocolitica p60 bl6 Pseudomonas aeruginosa Porphyromonas gingivalis Aeromonas cavia Helicobacter pylori kx1 npgec* | 5.98E-001 | 4 | 3.06E-003 | 8.28E-001 | 5.00E-001 | 1.00E+000 | 1.68E-001 | 6.41E-001 | 1.00E+000 | 8.32E-001 | 2.35E-001 | 8.00E-001 | 4.10E-001 | 1.00E+000 |
| 414 | 5 | 25 | *Helicobacter pylori kx2 npgec Pseudomonas aeruginosa fdr1 Lactobacillus acidophilus Staphylococcus aureus Helicobacter pylori kx1 npgec* | 5.98E-001 | 11 | 2.23E-004 | 8.28E-001 | 5.00E-001 | 3.93E-001 | 8.69E-001 | 6.08E-002 | 3.23E-001 | 1.00E+000 | 5.96E-001 | 3.93E-001 | 1.00E+000 | 1.00E+000 |
| 412 | 5 | 25 | *Yersinia enterocolitica wap bl6 Helicobacter pylori kx2 npgec Pseudomonas aeruginosa fdr440 Staphylococcus aureus Helicobacter pylori kx1 npgec* | 5.98E-001 | 5 | 4.30E-001 | 8.28E-001 | 5.00E-001 | 8.00E-001 | 5.54E-001 | 6.08E-002 | 1.00E+000 | 8.32E-001 | 2.35E-001 | 3.93E-001 | 1.00E+000 | 1.00E+000 |
| 984 | 10 | 5 | *Helicobacter pylori kx2 npgec Pseudomonas aeruginosa fdr1 Pseudomonas aeruginosa fdr440 Staphylococcus aureus Porphyromonas gingivalis Helicobacter pylori kx2 mgep Aeromonas cavia Helicobacter pylori kx1 npgec Ehrlichia chaffeensis liberty Aggregatibacter actinomycetemcomitans* | 5.99E-002 | 0 | 1.00E+000 | 6.41E-001 | 6.41E-001 | 9.70E-001 | 1.37E-001 | 3.05E-003 | 1.00E+000 | 1.00E+000 | 5.13E-001 | 5.24E-001 | 2.36E-001 | 9.37E-001 |
| 962 | 10 | 5 | *Yersinia enterocolitica wap bl6 Helicobacter pylori kx2 npgec Burkholderia pseudomallei Lactobacillus acidophilus Staphylococcus aureus Aeromonas cavia Helicobacter pylori kx1 npgec Ehrlichia chaffeensis liberty Helicobacter pylori kx2 mgep Escherichia coli* | 5.99E-002 | 0 | 1.00E+000 | 9.67E-001 | 1.39E-001 | 8.19E-001 | 4.00E-001 | 5.85E-001 | 5.68E-001 | 6.00E-001 | 7.41E-002 | 9.70E-001 | 1.00E+000 | 7.01E-001 |
| 874 | 6 | 10 | *Burkholderia pseudomallei Pseudomonas aeruginosa fdr1 Lactobacillus acidophilus Porphyromonas gingivalis Helicobacter pylori kx2 mgep Aggregatibacter actinomycetemcomitans* | 6.04E-001 | 0 | 1.00E+000 | 3.31E-001 | 9.08E-001 | 8.60E-001 | 4.39E-001 | 1.36E-001 | 3.78E-001 | 8.87E-001 | 9.44E-001 | 8.60E-001 | 9.10E-002 | 7.89E-001 |
| 868 | 6 | 10 | *streptococcus gordonii Staphylococcus aureus Pseudomonas aeruginosa Porphyromonas gingivalis streptococcus pneumoniae d39 Helicobacter pylori kx1 npgec* | 6.04E-001 | 0 | 1.00E+000 | 9.18E-002 | 9.90E-001 | 1.73E-001 | 9.54E-001 | 2.32E-002 | 1.00E+000 | 1.00E+000 | 9.44E-001 | 1.73E-001 | 9.10E-002 | 1.00E+000 |
| 860 | 6 | 10 | *Burkholderia pseudomallei Porphyromonas gingivalis Fusobacterium nucleatum Aeromonas cavia streptococcus pneumoniae d39 Aggregatibacter actinomycetemcomitans* | 6.04E-001 | 3 | 1.38E-001 | 9.18E-002 | 9.90E-001 | 8.60E-001 | 4.39E-001 | 1.36E-001 | 1.00E+000 | 8.87E-001 | 9.44E-001 | 8.60E-001 | 6.57E-003 | 7.89E-001 |
| 832 | 6 | 10 | *Helicobacter pylori kx2 npgec Ehrlichia chaffeensis wakulla Lactobacillus acidophilus Staphylococcus aureus Helicobacter pylori kx1 npgec Escherichia coli* | 6.04E-001 | 15 | 5.52E-009 | 9.08E-001 | 3.31E-001 | 5.03E-001 | 7.84E-001 | 4.13E-001 | 3.78E-001 | 8.87E-001 | 3.61E-001 | 8.60E-001 | 1.00E+000 | 7.89E-001 |
| 842 | 6 | 10 | *Yersinia enterocolitica wap bl6 Burkholderia pseudomallei Pseudomonas aeruginosa Mycobacterium tuberculosis streptococcus pneumoniae d39 Escherichia coli* | 6.04E-001 | 0 | 1.00E+000 | 9.18E-002 | 9.90E-001 | 8.60E-001 | 7.84E-001 | 9.56E-001 | 1.00E+000 | 4.58E-002 | 7.13E-001 | 1.73E-001 | 1.00E+000 | 7.89E-001 |
| 858 | 6 | 10 | *Helicobacter pylori kx2 npgec Staphylococcus aureus Bifidobacterium bifidum Fusobacterium nucleatum Aeromonas cavia Aggregatibacter actinomycetemcomitans* | 6.04E-001 | 0 | 1.00E+000 | 6.69E-001 | 6.69E-001 | 5.03E-001 | 7.84E-001 | 1.36E-001 | 3.78E-001 | 1.00E+000 | 7.13E-001 | 8.60E-001 | 9.10E-002 | 1.00E+000 |
| 836 | 6 | 10 | *Helicobacter pylori kx2 npgec Yersinia enterocolitica p60 bl6 Lactobacillus acidophilus Pseudomonas aeruginosa fdr440 Bifidobacterium bifidum Helicobacter pylori kx1 npgec* | 6.04E-001 | 0 | 1.00E+000 | 9.90E-001 | 9.18E-002 | 5.03E-001 | 7.84E-001 | 4.13E-001 | 4.88E-002 | 8.87E-001 | 3.61E-001 | 8.60E-001 | 1.00E+000 | 1.00E+000 |
| 852 | 6 | 10 | *Yersinia enterocolitica wap bc Yersinia enterocolitica p60 bc Ehrlichia chaffeensis wakulla Lactobacillus acidophilus Bifidobacterium bifidum Helicobacter pylori kx1 npgec* | 6.04E-001 | 0 | 1.00E+000 | 1.00E+000 | 1.03E-002 | 5.03E-001 | 7.84E-001 | 9.56E-001 | 4.88E-002 | 5.61E-001 | 3.61E-001 | 1.00E+000 | 1.00E+000 | 7.89E-001 |
| 1076 | 11 | 4 | *Yersinia enterocolitica wap bl6 Helicobacter pylori kx2 npgec Burkholderia pseudomallei Yersinia enterocolitica p60 bl6 Lactobacillus acidophilus Bifidobacterium bifidum Pseudomonas aeruginosa Porphyromonas gingivalis Helicobacter pylori kx1 npgec Aggregatibacter actinomycetemcomitans Pseudomonas aeruginosa fdr1* | 6.10E-002 | 0 | 1.00E+000 | 7.58E-001 | 5.00E-001 | 8.66E-001 | 3.18E-001 | 4.07E-001 | 1.63E-001 | 6.82E-001 | 6.16E-001 | 8.66E-001 | 2.77E-001 | 9.55E-001 |
| 1078 | 11 | 4 | *Helicobacter pylori kx2 npgec Burkholderia pseudomallei Lactobacillus acidophilus Staphylococcus aureus Pseudomonas aeruginosa Aeromonas cavia Helicobacter pylori kx1 npgec Aggregatibacter actinomycetemcomitans Pseudomonas aeruginosa fdr440 Mycobacterium tuberculosis Escherichia coli* | 6.10E-002 | 0 | 1.00E+000 | 2.42E-001 | 9.21E-001 | 8.66E-001 | 6.18E-001 | 4.07E-001 | 6.08E-001 | 6.82E-001 | 6.16E-001 | 3.03E-001 | 7.19E-001 | 9.55E-001 |
| 438 | 5 | 24 | *Helicobacter pylori kx2 npgec streptococcus gordonii Lactobacillus acidophilus Bifidobacterium bifidum streptococcus pneumoniae d39* | 6.16E-001 | 0 | 1.00E+000 | 8.28E-001 | 5.00E-001 | 1.26E-002 | 9.99E-001 | 2.75E-001 | 3.31E-002 | 1.00E+000 | 9.05E-001 | 8.00E-001 | 4.10E-001 | 1.00E+000 |
| 436 | 5 | 24 | *Lactobacillus acidophilus Pseudomonas aeruginosa fdr440 Staphylococcus aureus Bifidobacterium bifidum Mycobacterium tuberculosis* | 6.16E-001 | 0 | 1.00E+000 | 5.00E-001 | 8.28E-001 | 1.03E-001 | 9.99E-001 | 6.41E-001 | 3.31E-002 | 8.32E-001 | 1.00E+000 | 1.03E-001 | 1.00E+000 | 1.00E+000 |
| 434 | 5 | 24 | *Burkholderia pseudomallei Bifidobacterium bifidum Mycobacterium tuberculosis streptococcus pneumoniae d39 Escherichia coli* | 6.16E-001 | 0 | 1.00E+000 | 1.72E-001 | 9.76E-001 | 3.93E-001 | 9.82E-001 | 9.23E-001 | 3.23E-001 | 1.31E-001 | 9.05E-001 | 3.93E-001 | 1.00E+000 | 7.21E-001 |
| 442 | 5 | 24 | *Helicobacter pylori kx2 npgec Burkholderia pseudomallei Fusobacterium nucleatum Aeromonas cavia Helicobacter pylori kx1 npgec* | 6.16E-001 | 0 | 1.00E+000 | 8.28E-001 | 5.00E-001 | 1.00E+000 | 1.68E-001 | 2.75E-001 | 1.00E+000 | 8.32E-001 | 2.35E-001 | 1.00E+000 | 4.10E-001 | 7.21E-001 |
| 454 | 5 | 23 | *Burkholderia pseudomallei Staphylococcus aureus Aeromonas cavia streptococcus pneumoniae d39 Escherichia coli* | 6.31E-001 | 5 | 1.57E-001 | 1.72E-001 | 9.76E-001 | 3.93E-001 | 8.69E-001 | 6.41E-001 | 1.00E+000 | 4.46E-001 | 5.96E-001 | 3.93E-001 | 1.00E+000 | 7.21E-001 |
| 456 | 5 | 23 | *Burkholderia pseudomallei Lactobacillus acidophilus Pseudomonas aeruginosa fdr440 Bifidobacterium bifidum Ehrlichia chaffeensis liberty* | 6.31E-001 | 3 | 1.59E-001 | 8.28E-001 | 5.00E-001 | 3.93E-001 | 8.69E-001 | 9.23E-001 | 3.31E-002 | 8.32E-001 | 1.00E+000 | 8.00E-001 | 1.00E+000 | 2.88E-001 |
| 160 | 4 | 64 | *Helicobacter pylori kx2 npgec Lactobacillus acidophilus Pseudomonas aeruginosa Escherichia coli* | 6.31E-001 | 19 | 7.33E-006 | 6.97E-001 | 6.97E-001 | 7.19E-001 | 6.80E-001 | 8.66E-001 | 2.65E-001 | 7.55E-001 | 4.51E-001 | 7.19E-001 | 1.00E+000 | 1.00E+000 |
| 162 | 4 | 64 | *Helicobacter pylori kx2 npgec Ehrlichia chaffeensis arkansa Burkholderia pseudomallei Helicobacter pylori kx1 npgec* | 6.31E-001 | 35 | < 1.67E-026 | 9.47E-001 | 3.03E-001 | 1.00E+000 | 2.45E-001 | 4.95E-001 | 1.00E+000 | 7.55E-001 | 4.51E-001 | 1.00E+000 | 1.00E+000 | 1.96E-001 |
| 380 | 7 | 19 | *Helicobacter pylori kx2 npgec Helicobacter pylori kx1 mgep Burkholderia pseudomallei Lactobacillus acidophilus Bifidobacterium bifidum Helicobacter pylori kx2 mgep Helicobacter pylori kx1 npgec* | 6.44E-002 | 0 | 1.00E+000 | 9.96E-001 | 4.65E-002 | 6.02E-001 | 6.88E-001 | 2.36E-001 | 6.71E-002 | 9.25E-001 | 1.92E-001 | 1.00E+000 | 1.00E+000 | 8.42E-001 |
| 168 | 4 | 61 | *Staphylococcus aureus Porphyromonas gingivalis Helicobacter pylori kx1 npgec Ehrlichia chaffeensis liberty* | 6.48E-001 | 40 | 9.60E-007 | 6.97E-001 | 6.97E-001 | 7.19E-001 | 6.80E-001 | 1.46E-001 | 1.00E+000 | 1.00E+000 | 8.43E-001 | 7.19E-001 | 3.41E-001 | 6.34E-001 |
| 934 | 6 | 9 | *Helicobacter pylori kx2 npgec Ehrlichia chaffeensis wakulla Helicobacter pylori kx2 mgep Aeromonas cavia Helicobacter pylori kx1 npgec Ehrlichia chaffeensis liberty* | 6.48E-001 | 7 | 2.55E-002 | 1.00E+000 | 1.03E-002 | 1.00E+000 | 1.13E-001 | 4.13E-001 | 1.00E+000 | 1.00E+000 | 1.08E-001 | 1.00E+000 | 1.00E+000 | 3.82E-001 |
| 932 | 6 | 9 | *Helicobacter pylori kx2 npgec Burkholderia pseudomallei Ehrlichia chaffeensis wakulla Porphyromonas gingivalis Helicobacter pylori kx2 mgep Helicobacter pylori kx1 npgec* | 6.48E-001 | 4 | 3.64E-002 | 9.08E-001 | 3.31E-001 | 1.00E+000 | 1.13E-001 | 1.36E-001 | 1.00E+000 | 8.87E-001 | 3.61E-001 | 1.00E+000 | 4.74E-001 | 3.82E-001 |
| 946 | 6 | 9 | *Helicobacter pylori kx2 npgec Helicobacter pylori kx1 mgep Mycobacterium tuberculosis Helicobacter pylori kx2 mgep Helicobacter pylori kx1 npgec Escherichia coli* | 6.48E-001 | 11 | 1.74E-005 | 9.08E-001 | 3.31E-001 | 1.00E+000 | 4.39E-001 | 1.36E-001 | 1.00E+000 | 5.61E-001 | 1.64E-002 | 8.60E-001 | 1.00E+000 | 1.00E+000 |
| 930 | 6 | 9 | *Yersinia enterocolitica wap bl6 Burkholderia pseudomallei Yersinia enterocolitica p60 bl6 Yersinia enterocolitica p60 bc Anaplasma phagocytophilum Porphyromonas gingivalis* | 6.48E-001 | 0 | 1.00E+000 | 6.69E-001 | 6.69E-001 | 1.00E+000 | 1.13E-001 | 9.56E-001 | 1.00E+000 | 4.58E-002 | 3.61E-001 | 1.00E+000 | 4.74E-001 | 3.82E-001 |
| 938 | 6 | 9 | *Yersinia enterocolitica wap bl6 Helicobacter pylori kx2 npgec Yersinia enterocolitica p60 bl6 streptococcus pneumoniae d39 Helicobacter pylori kx1 npgec Ehrlichia chaffeensis liberty* | 6.48E-001 | 7 | 1.08E-002 | 9.90E-001 | 9.18E-002 | 8.60E-001 | 4.39E-001 | 4.13E-001 | 1.00E+000 | 5.61E-001 | 1.08E-001 | 8.60E-001 | 1.00E+000 | 7.89E-001 |
| 486 | 5 | 22 | *Burkholderia pseudomallei Lactobacillus acidophilus Pseudomonas aeruginosa Mycobacterium tuberculosis Aeromonas cavia* | 6.49E-001 | 0 | 1.00E+000 | 5.00E-001 | 8.28E-001 | 8.00E-001 | 8.69E-001 | 1.00E+000 | 3.23E-001 | 4.46E-001 | 9.05E-001 | 3.93E-001 | 1.00E+000 | 7.21E-001 |
| 480 | 5 | 22 | *Yersinia enterocolitica p60 bc Lactobacillus acidophilus Staphylococcus aureus Bifidobacterium bifidum Pseudomonas aeruginosa* | 6.49E-001 | 4 | 5.53E-002 | 8.28E-001 | 5.00E-001 | 1.03E-001 | 9.82E-001 | 9.23E-001 | 3.31E-002 | 8.32E-001 | 9.05E-001 | 3.93E-001 | 1.00E+000 | 1.00E+000 |
| 484 | 5 | 22 | *Lactobacillus acidophilus Bifidobacterium bifidum Porphyromonas gingivalis Fusobacterium nucleatum Aeromonas cavia* | 6.49E-001 | 6 | 3.14E-002 | 8.28E-001 | 5.00E-001 | 3.93E-001 | 8.69E-001 | 6.41E-001 | 3.31E-002 | 1.00E+000 | 9.05E-001 | 1.00E+000 | 6.29E-002 | 1.00E+000 |
| 478 | 5 | 22 | *Pseudomonas aeruginosa fdr440 Pseudomonas aeruginosa Helicobacter pylori kx1 npgec Ehrlichia chaffeensis liberty Aggregatibacter actinomycetemcomitans* | 6.49E-001 | 4 | 6.63E-002 | 5.00E-001 | 8.28E-001 | 1.00E+000 | 1.68E-001 | 2.75E-001 | 1.00E+000 | 1.00E+000 | 9.05E-001 | 3.93E-001 | 4.10E-001 | 7.21E-001 |
| 482 | 5 | 22 | *Yersinia enterocolitica wap bl6 Yersinia enterocolitica p60 bc Mycobacterium tuberculosis streptococcus pneumoniae d39 Helicobacter pylori kx1 npgec* | 6.49E-001 | 9 | 1.29E-001 | 8.28E-001 | 5.00E-001 | 8.00E-001 | 8.69E-001 | 6.41E-001 | 1.00E+000 | 1.31E-001 | 2.35E-001 | 3.93E-001 | 1.00E+000 | 1.00E+000 |
| 178 | 4 | 60 | *Yersinia enterocolitica wap bl6 Yersinia enterocolitica p60 bc Porphyromonas gingivalis Mycobacterium tuberculosis* | 6.56E-001 | 15 | 1.37E-001 | 6.97E-001 | 6.97E-001 | 1.00E+000 | 6.80E-001 | 8.66E-001 | 1.00E+000 | 6.34E-002 | 4.51E-001 | 7.19E-001 | 3.41E-001 | 1.00E+000 |
| 176 | 4 | 60 | *Burkholderia pseudomallei Staphylococcus aureus Helicobacter pylori kx2 mgep Helicobacter pylori kx1 npgec* | 6.56E-001 | 33 | 7.96E-007 | 6.97E-001 | 6.97E-001 | 7.19E-001 | 6.80E-001 | 1.46E-001 | 1.00E+000 | 7.55E-001 | 4.51E-001 | 7.19E-001 | 1.00E+000 | 6.34E-001 |
| 174 | 4 | 60 | *Burkholderia pseudomallei Pseudomonas aeruginosa fdr1 Lactobacillus acidophilus Bifidobacterium bifidum* | 6.56E-001 | 18 | 1.23E-006 | 6.97E-001 | 6.97E-001 | 2.77E-001 | 9.37E-001 | 8.66E-001 | 2.02E-002 | 7.55E-001 | 1.00E+000 | 7.19E-001 | 1.00E+000 | 6.34E-001 |
| 42 | 3 | 176 | *Lactobacillus acidophilus Bifidobacterium bifidum Pseudomonas aeruginosa* | 6.68E-001 | 40 | 1.38E-009 | 8.84E-001 | 5.00E-001 | 1.63E-001 | 9.81E-001 | 1.00E+000 | 1.03E-002 | 1.00E+000 | 1.00E+000 | 6.08E-001 | 1.00E+000 | 1.00E+000 |
| 194 | 4 | 57 | *Lactobacillus acidophilus Pseudomonas aeruginosa fdr440 Porphyromonas gingivalis Escherichia coli* | 6.74E-001 | 3 | 6.21E-001 | 3.03E-001 | 9.47E-001 | 7.19E-001 | 6.80E-001 | 4.95E-001 | 2.65E-001 | 7.55E-001 | 8.43E-001 | 7.19E-001 | 3.41E-001 | 1.00E+000 |
| 50 | 5 | 98 | *Yersinia enterocolitica wap bl6 Yersinia enterocolitica p60 bl6 Yersinia enterocolitica p60 bc Porphyromonas gingivalis Aeromonas cavia* | 6.78E-002 | 37 | 1.48E-006 | 9.76E-001 | 1.72E-001 | 1.00E+000 | 1.68E-001 | 9.23E-001 | 1.00E+000 | 1.31E-001 | 4.69E-002 | 1.00E+000 | 4.10E-001 | 1.00E+000 |
| 1258 | 7 | 5 | *Lactobacillus acidophilus Pseudomonas aeruginosa fdr440 Listeria monocytogenes Bifidobacterium bifidum Mycobacterium tuberculosis streptococcus pneumoniae d39 Escherichia coli* | 6.80E-001 | 0 | 1.00E+000 | 2.05E-001 | 9.53E-001 | 6.35E-002 | 9.99E-001 | 8.39E-001 | 3.05E-003 | 6.60E-001 | 8.03E-001 | 2.56E-001 | 1.00E+000 | 1.00E+000 |
| 1260 | 7 | 5 | *Yersinia enterocolitica wap bl6 Pseudomonas aeruginosa fdr440 Listeria monocytogenes Porphyromonas gingivalis Mycobacterium tuberculosis Helicobacter pylori kx1 npgec Ehrlichia chaffeensis liberty* | 6.80E-001 | 0 | 1.00E+000 | 5.00E-001 | 7.95E-001 | 9.03E-001 | 6.88E-001 | 5.46E-001 | 4.30E-001 | 6.60E-001 | 4.88E-001 | 6.02E-001 | 5.32E-001 | 8.42E-001 |
| 1274 | 7 | 5 | *Ehrlichia chaffeensis arkansa Burkholderia pseudomallei Ehrlichia chaffeensis wakulla Bifidobacterium bifidum Helicobacter pylori kx2 mgep Helicobacter pylori kx1 npgec Ehrlichia chaffeensis liberty* | 6.80E-001 | 0 | 1.00E+000 | 9.96E-001 | 4.65E-002 | 9.03E-001 | 3.40E-001 | 8.39E-001 | 4.30E-001 | 9.25E-001 | 8.03E-001 | 1.00E+000 | 1.00E+000 | 2.81E-002 |
| 1272 | 7 | 5 | *Yersinia enterocolitica wap bl6 Helicobacter pylori kx2 npgec Helicobacter pylori kx1 mgep Porphyromonas gingivalis Helicobacter pylori kx2 mgep Aeromonas cavia Helicobacter pylori kx1 npgec* | 6.80E-001 | 3 | 3.27E-001 | 9.96E-001 | 4.65E-002 | 1.00E+000 | 7.55E-002 | 6.08E-002 | 1.00E+000 | 9.25E-001 | 5.25E-003 | 1.00E+000 | 5.32E-001 | 1.00E+000 |
| 1264 | 7 | 5 | *Yersinia enterocolitica wap bl6 Helicobacter pylori kx2 npgec Pseudomonas aeruginosa fdr1 Porphyromonas gingivalis Helicobacter pylori kx2 mgep streptococcus pneumoniae d39 Helicobacter pylori kx1 npgec* | 6.80E-001 | 4 | 2.19E-002 | 7.95E-001 | 5.00E-001 | 9.03E-001 | 3.40E-001 | 8.14E-003 | 1.00E+000 | 9.25E-001 | 1.92E-001 | 6.02E-001 | 5.32E-001 | 1.00E+000 |
| 1252 | 7 | 5 | *Helicobacter pylori kx2 npgec Burkholderia pseudomallei Helicobacter pylori kx2 mgep streptococcus pneumoniae d39 Helicobacter pylori kx1 npgec Ehrlichia chaffeensis liberty Aggregatibacter actinomycetemcomitans* | 6.80E-001 | 0 | 1.00E+000 | 7.95E-001 | 5.00E-001 | 9.03E-001 | 3.40E-001 | 6.08E-002 | 1.00E+000 | 9.25E-001 | 4.88E-001 | 9.03E-001 | 5.32E-001 | 4.72E-001 |
| 1256 | 7 | 5 | *Burkholderia pseudomallei Pseudomonas aeruginosa fdr1 Lactobacillus acidophilus Bifidobacterium bifidum Mycobacterium tuberculosis streptococcus pneumoniae d39 Helicobacter pylori kx1 npgec* | 6.80E-001 | 0 | 1.00E+000 | 5.00E-001 | 7.95E-001 | 2.56E-001 | 9.86E-001 | 5.46E-001 | 6.71E-002 | 6.60E-001 | 9.67E-001 | 2.56E-001 | 1.00E+000 | 8.42E-001 |
| 200 | 4 | 56 | *Lactobacillus acidophilus Bifidobacterium bifidum Aeromonas cavia Escherichia coli* | 6.82E-001 | 19 | 2.76E-003 | 9.47E-001 | 3.03E-001 | 2.77E-001 | 9.37E-001 | 1.00E+000 | 2.02E-002 | 7.55E-001 | 4.51E-001 | 1.00E+000 | 1.00E+000 | 1.00E+000 |
| 508 | 5 | 20 | *Yersinia enterocolitica wap bl6 Staphylococcus aureus streptococcus pneumoniae d39 Escherichia coli Aggregatibacter actinomycetemcomitans* | 6.85E-001 | 0 | 1.00E+000 | 1.72E-001 | 9.76E-001 | 3.93E-001 | 8.69E-001 | 2.75E-001 | 1.00E+000 | 4.46E-001 | 5.96E-001 | 3.93E-001 | 4.10E-001 | 1.00E+000 |
| 516 | 5 | 20 | *Helicobacter pylori kx2 npgec Lactobacillus acidophilus Bifidobacterium bifidum Mycobacterium tuberculosis Ehrlichia chaffeensis liberty* | 6.85E-001 | 13 | 2.86E-011 | 9.76E-001 | 1.72E-001 | 3.93E-001 | 9.82E-001 | 9.23E-001 | 3.31E-002 | 8.32E-001 | 9.05E-001 | 8.00E-001 | 1.00E+000 | 7.21E-001 |
| 1296 | 8 | 4 | *Burkholderia pseudomallei Pseudomonas aeruginosa fdr1 Lactobacillus acidophilus Pseudomonas aeruginosa fdr440 Bifidobacterium bifidum Pseudomonas aeruginosa Helicobacter pylori kx2 mgep Helicobacter pylori kx1 npgec* | 6.86E-001 | 0 | 1.00E+000 | 6.52E-001 | 6.52E-001 | 6.88E-001 | 5.89E-001 | 3.51E-001 | 8.78E-002 | 9.50E-001 | 8.68E-001 | 3.45E-001 | 1.00E+000 | 8.82E-001 |
| 1322 | 8 | 4 | *Helicobacter pylori kx2 npgec Lactobacillus acidophilus Pseudomonas aeruginosa fdr440 Staphylococcus aureus Pseudomonas aeruginosa Helicobacter pylori kx2 mgep Helicobacter pylori kx1 npgec Escherichia coli* | 6.86E-001 | 0 | 1.00E+000 | 6.52E-001 | 6.52E-001 | 6.88E-001 | 5.89E-001 | 1.21E-001 | 4.79E-001 | 9.50E-001 | 2.94E-001 | 3.45E-001 | 1.00E+000 | 1.00E+000 |
| 1336 | 8 | 4 | *Yersinia enterocolitica wap bl6 Helicobacter pylori kx2 npgec Staphylococcus aureus Aeromonas cavia Helicobacter pylori kx1 npgec Ehrlichia chaffeensis wakulla Pseudomonas aeruginosa fdr1 Helicobacter pylori kx2 mgep* | 6.86E-001 | 0 | 1.00E+000 | 9.78E-001 | 1.19E-001 | 9.33E-001 | 2.57E-001 | 1.21E-001 | 1.00E+000 | 9.50E-001 | 9.08E-002 | 6.88E-001 | 1.00E+000 | 8.82E-001 |
| 1310 | 8 | 4 | *Helicobacter pylori kx2 npgec Lactobacillus acidophilus Staphylococcus aureus Porphyromonas gingivalis streptococcus pneumoniae d39 Helicobacter pylori kx1 npgec streptococcus gordonii Escherichia coli* | 6.86E-001 | 0 | 1.00E+000 | 3.48E-001 | 8.81E-001 | 1.07E-001 | 9.69E-001 | 2.47E-002 | 4.79E-001 | 9.50E-001 | 6.05E-001 | 6.88E-001 | 1.58E-001 | 1.00E+000 |
| 1330 | 8 | 4 | *Helicobacter pylori kx2 npgec Helicobacter pylori kx1 mgep Lactobacillus acidophilus Listeria monocytogenes Bifidobacterium bifidum Helicobacter pylori kx1 npgec Helicobacter pylori kx2 mgep Escherichia coli* | 6.86E-001 | 0 | 1.00E+000 | 9.78E-001 | 1.19E-001 | 3.45E-001 | 8.54E-001 | 3.51E-001 | 4.88E-003 | 9.50E-001 | 1.64E-002 | 1.00E+000 | 1.00E+000 | 1.00E+000 |
| 1326 | 8 | 4 | *Yersinia enterocolitica wap bl6 Helicobacter pylori kx2 npgec Burkholderia pseudomallei Lactobacillus acidophilus Staphylococcus aureus Helicobacter pylori kx1 npgec Ehrlichia chaffeensis liberty streptococcus pyogenes* | 6.86E-001 | 4 | 5.84E-005 | 9.78E-001 | 1.19E-001 | 3.45E-001 | 8.54E-001 | 6.64E-001 | 4.79E-001 | 7.43E-001 | 6.05E-001 | 9.33E-001 | 1.00E+000 | 5.57E-001 |
| 1302 | 8 | 4 | *Helicobacter pylori kx2 npgec Burkholderia pseudomallei Lactobacillus acidophilus Listeria monocytogenes Porphyromonas gingivalis Helicobacter pylori kx2 mgep Helicobacter pylori kx1 npgec Escherichia coli* | 6.86E-001 | 0 | 1.00E+000 | 6.52E-001 | 6.52E-001 | 6.88E-001 | 5.89E-001 | 3.51E-001 | 8.78E-002 | 7.43E-001 | 9.08E-002 | 1.00E+000 | 5.86E-001 | 8.82E-001 |
| 1318 | 8 | 4 | *Burkholderia pseudomallei Lactobacillus acidophilus Bifidobacterium bifidum Fusobacterium nucleatum streptococcus pneumoniae d39 Aggregatibacter actinomycetemcomitans streptococcus gordonii Mycobacterium tuberculosis* | 6.86E-001 | 0 | 1.00E+000 | 1.19E-001 | 9.78E-001 | 1.07E-001 | 9.96E-001 | 3.51E-001 | 8.78E-002 | 7.43E-001 | 1.00E+000 | 6.88E-001 | 1.76E-002 | 8.82E-001 |
| 1298 | 8 | 4 | *Helicobacter pylori kx2 npgec Lactobacillus acidophilus Porphyromonas gingivalis streptococcus pneumoniae d39 Helicobacter pylori kx1 npgec Ehrlichia chaffeensis liberty Pseudomonas aeruginosa fdr440 Escherichia coli* | 6.86E-001 | 0 | 1.00E+000 | 6.52E-001 | 6.52E-001 | 6.88E-001 | 5.89E-001 | 1.21E-001 | 4.79E-001 | 9.50E-001 | 6.05E-001 | 6.88E-001 | 5.86E-001 | 8.82E-001 |
| 1332 | 8 | 4 | *Helicobacter pylori kx2 npgec Helicobacter pylori kx1 mgep Ehrlichia chaffeensis arkansa Lactobacillus acidophilus Bifidobacterium bifidum Helicobacter pylori kx2 mgep Helicobacter pylori kx1 npgec Escherichia coli* | 6.86E-001 | 0 | 1.00E+000 | 9.98E-001 | 2.24E-002 | 6.88E-001 | 5.89E-001 | 3.51E-001 | 8.78E-002 | 9.50E-001 | 9.08E-002 | 1.00E+000 | 1.00E+000 | 8.82E-001 |
| 1316 | 8 | 4 | *Yersinia enterocolitica wap bl6 Burkholderia pseudomallei Lactobacillus acidophilus Bifidobacterium bifidum Pseudomonas aeruginosa Aggregatibacter actinomycetemcomitans Mycobacterium tuberculosis Escherichia coli* | 6.86E-001 | 0 | 1.00E+000 | 3.48E-001 | 8.81E-001 | 6.88E-001 | 8.54E-001 | 9.87E-001 | 8.78E-002 | 1.46E-001 | 8.68E-001 | 6.88E-001 | 5.86E-001 | 8.82E-001 |
| 1324 | 8 | 4 | *Helicobacter pylori kx2 npgec Burkholderia pseudomallei Pseudomonas aeruginosa fdr1 Pseudomonas aeruginosa fdr440 Staphylococcus aureus Mycobacterium tuberculosis Helicobacter pylori kx2 mgep Helicobacter pylori kx1 npgec* | 6.86E-001 | 0 | 1.00E+000 | 3.48E-001 | 8.81E-001 | 9.33E-001 | 5.89E-001 | 2.47E-002 | 1.00E+000 | 7.43E-001 | 6.05E-001 | 1.07E-001 | 1.00E+000 | 8.82E-001 |
| 1300 | 8 | 4 | *Helicobacter pylori kx2 npgec Staphylococcus aureus Listeria monocytogenes Aeromonas cavia streptococcus pneumoniae d39 Helicobacter pylori kx1 npgec Pseudomonas aeruginosa fdr440 Escherichia coli* | 6.86E-001 | 0 | 1.00E+000 | 3.48E-001 | 8.81E-001 | 3.45E-001 | 8.54E-001 | 1.21E-001 | 4.79E-001 | 9.50E-001 | 9.08E-002 | 3.45E-001 | 1.00E+000 | 1.00E+000 |
| 1334 | 8 | 4 | *Helicobacter pylori kx2 npgec Burkholderia pseudomallei Lactobacillus acidophilus Listeria monocytogenes Bifidobacterium bifidum Porphyromonas gingivalis Helicobacter pylori kx1 npgec Ehrlichia chaffeensis liberty* | 6.86E-001 | 0 | 1.00E+000 | 8.81E-001 | 3.48E-001 | 3.45E-001 | 8.54E-001 | 6.64E-001 | 4.88E-003 | 9.50E-001 | 6.05E-001 | 1.00E+000 | 5.86E-001 | 5.57E-001 |
| 1022 | 6 | 8 | *Ehrlichia chaffeensis arkansa Burkholderia pseudomallei Lactobacillus acidophilus Staphylococcus aureus Bifidobacterium bifidum Aggregatibacter actinomycetemcomitans* | 6.97E-001 | 0 | 1.00E+000 | 6.69E-001 | 6.69E-001 | 1.73E-001 | 9.54E-001 | 7.55E-001 | 4.88E-002 | 8.87E-001 | 1.00E+000 | 8.60E-001 | 4.74E-001 | 3.82E-001 |
| 1034 | 6 | 8 | *Yersinia enterocolitica wap bl6 Helicobacter pylori kx2 npgec Burkholderia pseudomallei Pseudomonas aeruginosa fdr440 streptococcus pneumoniae d39 Helicobacter pylori kx1 npgec* | 6.97E-001 | 2 | 6.56E-001 | 6.69E-001 | 6.69E-001 | 8.60E-001 | 4.39E-001 | 1.36E-001 | 1.00E+000 | 5.61E-001 | 3.61E-001 | 5.03E-001 | 1.00E+000 | 7.89E-001 |
| 1036 | 6 | 8 | *Burkholderia pseudomallei Pseudomonas aeruginosa fdr1 Lactobacillus acidophilus Staphylococcus aureus Helicobacter pylori kx1 npgec Escherichia coli* | 6.97E-001 | 2 | 3.50E-001 | 3.31E-001 | 9.08E-001 | 5.03E-001 | 7.84E-001 | 4.13E-001 | 3.78E-001 | 5.61E-001 | 7.13E-001 | 5.03E-001 | 1.00E+000 | 7.89E-001 |
| 1024 | 6 | 8 | *Pseudomonas aeruginosa fdr440 Pseudomonas aeruginosa Helicobacter pylori kx2 mgep Helicobacter pylori kx1 npgec Escherichia coli Ehrlichia chaffeensis liberty* | 6.97E-001 | 4 | 5.13E-003 | 6.69E-001 | 6.69E-001 | 1.00E+000 | 1.13E-001 | 4.13E-001 | 1.00E+000 | 8.87E-001 | 3.61E-001 | 5.03E-001 | 1.00E+000 | 7.89E-001 |
| 1042 | 6 | 8 | *Helicobacter pylori kx2 npgec Ehrlichia chaffeensis wakulla Bifidobacterium bifidum Porphyromonas gingivalis Mycobacterium tuberculosis Helicobacter pylori kx1 npgec* | 6.97E-001 | 0 | 1.00E+000 | 9.08E-001 | 3.31E-001 | 8.60E-001 | 7.84E-001 | 4.13E-001 | 3.78E-001 | 8.87E-001 | 7.13E-001 | 8.60E-001 | 4.74E-001 | 7.89E-001 |
| 560 | 5 | 19 | *Yersinia enterocolitica p60 bl6 Staphylococcus aureus Bifidobacterium bifidum Helicobacter pylori kx1 npgec Ehrlichia chaffeensis liberty* | 7.02E-001 | 7 | 3.87E-008 | 9.76E-001 | 1.72E-001 | 3.93E-001 | 8.69E-001 | 6.41E-001 | 3.23E-001 | 8.32E-001 | 5.96E-001 | 8.00E-001 | 1.00E+000 | 7.21E-001 |
| 558 | 5 | 19 | *Yersinia enterocolitica wap bl6 Burkholderia pseudomallei Yersinia enterocolitica p60 bl6 Pseudomonas aeruginosa Aeromonas cavia* | 7.02E-001 | 1 | 5.61E-001 | 8.28E-001 | 5.00E-001 | 1.00E+000 | 1.68E-001 | 1.00E+000 | 1.00E+000 | 1.31E-001 | 2.35E-001 | 8.00E-001 | 1.00E+000 | 7.21E-001 |
| 556 | 5 | 19 | *Helicobacter pylori kx2 npgec Helicobacter pylori kx1 mgep Staphylococcus aureus Helicobacter pylori kx2 mgep Helicobacter pylori kx1 npgec* | 7.02E-001 | 16 | 1.77E-003 | 9.76E-001 | 1.72E-001 | 8.00E-001 | 5.54E-001 | 5.13E-003 | 1.00E+000 | 1.00E+000 | 4.69E-002 | 8.00E-001 | 1.00E+000 | 1.00E+000 |
| 728 | 8 | 9 | *Helicobacter pylori kx2 npgec Burkholderia pseudomallei Porphyromonas gingivalis streptococcus pneumoniae d39 Helicobacter pylori kx1 npgec Pseudomonas aeruginosa fdr1 Pseudomonas aeruginosa fdr440 Escherichia coli* | 7.06E-002 | 0 | 1.00E+000 | 1.19E-001 | 9.78E-001 | 9.33E-001 | 2.57E-001 | 2.47E-002 | 1.00E+000 | 7.43E-001 | 6.05E-001 | 3.45E-001 | 5.86E-001 | 8.82E-001 |
| 742 | 8 | 9 | *Helicobacter pylori kx2 npgec Lactobacillus acidophilus Bifidobacterium bifidum Pseudomonas aeruginosa streptococcus pneumoniae d39 Helicobacter pylori kx1 npgec Aggregatibacter actinomycetemcomitans Mycobacterium tuberculosis* | 7.06E-002 | 0 | 1.00E+000 | 6.52E-001 | 6.52E-001 | 3.45E-001 | 9.69E-001 | 3.51E-001 | 8.78E-002 | 9.50E-001 | 8.68E-001 | 3.45E-001 | 5.86E-001 | 1.00E+000 |
| 732 | 8 | 9 | *Yersinia enterocolitica wap bl6 Helicobacter pylori kx2 npgec Burkholderia pseudomallei Porphyromonas gingivalis Aeromonas cavia Helicobacter pylori kx1 npgec Ehrlichia chaffeensis liberty Pseudomonas aeruginosa fdr1* | 7.06E-002 | 5 | 9.20E-005 | 8.81E-001 | 3.48E-001 | 1.00E+000 | 4.96E-002 | 3.51E-001 | 1.00E+000 | 7.43E-001 | 2.94E-001 | 9.33E-001 | 5.86E-001 | 5.57E-001 |
| 740 | 8 | 9 | *Yersinia enterocolitica wap bl6 Burkholderia pseudomallei Staphylococcus aureus Porphyromonas gingivalis Aeromonas cavia Aggregatibacter actinomycetemcomitans Mycobacterium tuberculosis Escherichia coli* | 7.06E-002 | 0 | 1.00E+000 | 1.19E-001 | 9.78E-001 | 9.33E-001 | 5.89E-001 | 6.64E-001 | 1.00E+000 | 1.46E-001 | 6.05E-001 | 6.88E-001 | 1.58E-001 | 8.82E-001 |
| 726 | 8 | 9 | *Yersinia enterocolitica wap bl6 Burkholderia pseudomallei Yersinia enterocolitica p60 bl6 Lactobacillus acidophilus Staphylococcus aureus Bifidobacterium bifidum Pseudomonas aeruginosa Porphyromonas gingivalis* | 7.06E-002 | 0 | 1.00E+000 | 6.52E-001 | 6.52E-001 | 3.45E-001 | 8.54E-001 | 8.98E-001 | 8.78E-002 | 4.11E-001 | 8.68E-001 | 6.88E-001 | 5.86E-001 | 8.82E-001 |
| 56 | 3 | 153 | *Helicobacter pylori kx2 npgec Yersinia enterocolitica p60 bc Helicobacter pylori kx1 npgec* | 7.19E-001 | 141 | < 1.67E-026 | 1.00E+000 | 1.16E-001 | 1.00E+000 | 3.54E-001 | 3.21E-001 | 1.00E+000 | 6.46E-001 | 3.96E-002 | 1.00E+000 | 1.00E+000 | 1.00E+000 |
| 588 | 5 | 18 | *Helicobacter pylori kx2 npgec Burkholderia pseudomallei Ehrlichia chaffeensis wakulla Bifidobacterium bifidum Ehrlichia chaffeensis liberty* | 7.21E-001 | 11 | 1.28E-004 | 9.76E-001 | 1.72E-001 | 8.00E-001 | 5.54E-001 | 9.23E-001 | 3.23E-001 | 8.32E-001 | 9.05E-001 | 1.00E+000 | 1.00E+000 | 5.72E-002 |
| 598 | 5 | 18 | *Lactobacillus acidophilus Pseudomonas aeruginosa fdr440 Bifidobacterium bifidum Pseudomonas aeruginosa Aeromonas cavia* | 7.21E-001 | 1 | 8.75E-002 | 8.28E-001 | 5.00E-001 | 3.93E-001 | 8.69E-001 | 9.23E-001 | 3.31E-002 | 1.00E+000 | 9.05E-001 | 3.93E-001 | 1.00E+000 | 1.00E+000 |
| 224 | 4 | 51 | *Burkholderia pseudomallei Bifidobacterium bifidum Pseudomonas aeruginosa Mycobacterium tuberculosis* | 7.22E-001 | 2 | 5.59E-001 | 3.03E-001 | 9.47E-001 | 7.19E-001 | 9.37E-001 | 1.00E+000 | 2.65E-001 | 3.20E-001 | 1.00E+000 | 2.77E-001 | 1.00E+000 | 6.34E-001 |
| 58 | 3 | 149 | *Helicobacter pylori kx2 npgec Staphylococcus aureus streptococcus pneumoniae d39* | 7.25E-001 | 62 | 1.69E-002 | 5.00E-001 | 8.84E-001 | 1.63E-001 | 9.81E-001 | 4.88E-002 | 1.00E+000 | 1.00E+000 | 7.45E-001 | 1.63E-001 | 1.00E+000 | 1.00E+000 |
| 64 | 3 | 145 | *Lactobacillus acidophilus Mycobacterium tuberculosis Escherichia coli* | 7.27E-001 | 8 | 9.65E-001 | 5.00E-001 | 8.84E-001 | 6.08E-001 | 9.81E-001 | 1.00E+000 | 2.04E-001 | 1.92E-001 | 7.45E-001 | 6.08E-001 | 1.00E+000 | 1.00E+000 |
| 610 | 5 | 17 | *Burkholderia pseudomallei Lactobacillus acidophilus Anaplasma phagocytophilum Bifidobacterium bifidum Porphyromonas gingivalis* | 7.39E-001 | 13 | 7.31E-012 | 5.00E-001 | 8.28E-001 | 3.93E-001 | 8.69E-001 | 9.23E-001 | 3.31E-002 | 8.32E-001 | 1.00E+000 | 1.00E+000 | 4.10E-001 | 2.88E-001 |
| 614 | 5 | 17 | *Burkholderia pseudomallei Yersinia enterocolitica p60 bc Staphylococcus aureus Aeromonas cavia Ehrlichia chaffeensis liberty* | 7.39E-001 | 23 | 4.18E-012 | 8.28E-001 | 5.00E-001 | 8.00E-001 | 5.54E-001 | 9.23E-001 | 1.00E+000 | 4.46E-001 | 5.96E-001 | 8.00E-001 | 1.00E+000 | 2.88E-001 |
| 612 | 5 | 17 | *Helicobacter pylori kx2 npgec Ehrlichia chaffeensis wakulla Porphyromonas gingivalis Helicobacter pylori kx1 npgec Ehrlichia chaffeensis liberty* | 7.39E-001 | 22 | 3.59E-005 | 9.76E-001 | 1.72E-001 | 1.00E+000 | 1.68E-001 | 2.75E-001 | 1.00E+000 | 1.00E+000 | 5.96E-001 | 1.00E+000 | 4.10E-001 | 2.88E-001 |
| 616 | 5 | 17 | *Pseudomonas aeruginosa fdr1 Pseudomonas aeruginosa fdr440 Aeromonas cavia Helicobacter pylori kx1 npgec Escherichia coli* | 7.39E-001 | 1 | 6.67E-001 | 5.00E-001 | 8.28E-001 | 1.00E+000 | 1.68E-001 | 2.75E-001 | 1.00E+000 | 8.32E-001 | 2.35E-001 | 3.93E-001 | 1.00E+000 | 1.00E+000 |
| 618 | 5 | 17 | *Lactobacillus acidophilus Pseudomonas aeruginosa fdr440 Staphylococcus aureus Escherichia coli Aggregatibacter actinomycetemcomitans* | 7.39E-001 | 0 | 1.00E+000 | 1.72E-001 | 9.76E-001 | 3.93E-001 | 8.69E-001 | 2.75E-001 | 3.23E-001 | 8.32E-001 | 9.05E-001 | 3.93E-001 | 4.10E-001 | 1.00E+000 |
| 398 | 7 | 18 | *Helicobacter pylori kx2 npgec Lactobacillus acidophilus Bifidobacterium bifidum Porphyromonas gingivalis Helicobacter pylori kx1 npgec Ehrlichia chaffeensis liberty Aggregatibacter actinomycetemcomitans* | 7.40E-002 | 7 | 3.87E-008 | 9.53E-001 | 2.05E-001 | 6.02E-001 | 6.88E-001 | 2.36E-001 | 6.71E-002 | 1.00E+000 | 8.03E-001 | 1.00E+000 | 1.23E-001 | 8.42E-001 |
| 396 | 7 | 18 | *Helicobacter pylori kx2 npgec Staphylococcus aureus Aeromonas cavia streptococcus pneumoniae d39 Helicobacter pylori kx1 npgec Ehrlichia chaffeensis liberty Aggregatibacter actinomycetemcomitans* | 7.40E-002 | 2 | 2.83E-001 | 7.95E-001 | 5.00E-001 | 6.02E-001 | 6.88E-001 | 6.08E-002 | 1.00E+000 | 1.00E+000 | 4.88E-001 | 6.02E-001 | 5.32E-001 | 8.42E-001 |
| 54 | 3 | 157 | *Helicobacter pylori kx2 npgec Helicobacter pylori kx1 npgec Ehrlichia chaffeensis liberty* | 7.44E-001 | 165 | < 1.67E-026 | 1.00E+000 | 1.16E-001 | 1.00E+000 | 3.54E-001 | 3.21E-001 | 1.00E+000 | 1.00E+000 | 2.87E-001 | 1.00E+000 | 1.00E+000 | 5.25E-001 |
| 244 | 4 | 48 | *Burkholderia pseudomallei Pseudomonas aeruginosa fdr440 Staphylococcus aureus Helicobacter pylori kx1 npgec* | 7.46E-001 | 14 | 2.21E-002 | 3.03E-001 | 9.47E-001 | 7.19E-001 | 6.80E-001 | 1.46E-001 | 1.00E+000 | 7.55E-001 | 8.43E-001 | 2.77E-001 | 1.00E+000 | 6.34E-001 |
| 240 | 4 | 48 | *Lactobacillus acidophilus Bifidobacterium bifidum streptococcus pneumoniae d39 Aggregatibacter actinomycetemcomitans* | 7.46E-001 | 13 | 4.64E-001 | 6.97E-001 | 6.97E-001 | 4.86E-002 | 9.96E-001 | 4.95E-001 | 2.02E-002 | 1.00E+000 | 1.00E+000 | 7.19E-001 | 3.41E-001 | 1.00E+000 |
| 1084 | 6 | 7 | *Yersinia enterocolitica wap bl6 Burkholderia pseudomallei Yersinia enterocolitica p60 bl6 Pseudomonas aeruginosa fdr440 Pseudomonas aeruginosa Helicobacter pylori kx1 npgec* | 7.47E-001 | 0 | 1.00E+000 | 6.69E-001 | 6.69E-001 | 1.00E+000 | 1.13E-001 | 7.55E-001 | 1.00E+000 | 2.16E-001 | 3.61E-001 | 5.03E-001 | 1.00E+000 | 7.89E-001 |
| 1122 | 6 | 7 | *Helicobacter pylori kx2 npgec Burkholderia pseudomallei Pseudomonas aeruginosa fdr440 Bifidobacterium bifidum Aeromonas cavia Ehrlichia chaffeensis liberty* | 7.47E-001 | 0 | 1.00E+000 | 9.08E-001 | 3.31E-001 | 8.60E-001 | 4.39E-001 | 7.55E-001 | 3.78E-001 | 8.87E-001 | 7.13E-001 | 8.60E-001 | 1.00E+000 | 3.82E-001 |
| 1112 | 6 | 7 | *Helicobacter pylori kx2 npgec Mycobacterium tuberculosis Helicobacter pylori kx2 mgep Helicobacter pylori kx1 npgec Ehrlichia chaffeensis liberty Aggregatibacter actinomycetemcomitans* | 7.47E-001 | 10 | 2.45E-005 | 9.08E-001 | 3.31E-001 | 1.00E+000 | 4.39E-001 | 1.36E-001 | 1.00E+000 | 8.87E-001 | 3.61E-001 | 8.60E-001 | 4.74E-001 | 7.89E-001 |
| 1098 | 6 | 7 | *Helicobacter pylori kx2 npgec Burkholderia pseudomallei Ehrlichia chaffeensis wakulla streptococcus pyogenes Aeromonas cavia Helicobacter pylori kx1 npgec* | 7.47E-001 | 4 | 7.58E-004 | 9.90E-001 | 9.18E-002 | 8.60E-001 | 4.39E-001 | 7.55E-001 | 1.00E+000 | 8.87E-001 | 3.61E-001 | 1.00E+000 | 1.00E+000 | 3.82E-001 |
| 1080 | 6 | 7 | *Helicobacter pylori kx2 npgec Ehrlichia chaffeensis arkansa Burkholderia pseudomallei Bifidobacterium bifidum streptococcus pneumoniae d39 Helicobacter pylori kx1 npgec* | 7.47E-001 | 0 | 1.00E+000 | 9.08E-001 | 3.31E-001 | 5.03E-001 | 7.84E-001 | 4.13E-001 | 3.78E-001 | 8.87E-001 | 7.13E-001 | 8.60E-001 | 1.00E+000 | 3.82E-001 |
| 1092 | 6 | 7 | *Yersinia enterocolitica wap bl6 Helicobacter pylori kx2 npgec Yersinia enterocolitica p60 bc streptococcus gordonii streptococcus pneumoniae d39 Helicobacter pylori kx1 npgec* | 7.47E-001 | 5 | 3.94E-001 | 9.08E-001 | 3.31E-001 | 5.03E-001 | 7.84E-001 | 1.36E-001 | 1.00E+000 | 5.61E-001 | 1.08E-001 | 8.60E-001 | 4.74E-001 | 1.00E+000 |
| 1114 | 6 | 7 | *Helicobacter pylori kx2 npgec Ehrlichia chaffeensis arkansa Burkholderia pseudomallei Staphylococcus aureus Bifidobacterium bifidum streptococcus pyogenes* | 7.47E-001 | 4 | 7.58E-004 | 9.08E-001 | 3.31E-001 | 1.73E-001 | 9.54E-001 | 7.55E-001 | 3.78E-001 | 8.87E-001 | 9.44E-001 | 8.60E-001 | 1.00E+000 | 3.82E-001 |
| 1096 | 6 | 7 | *Helicobacter pylori kx2 npgec Burkholderia pseudomallei Ehrlichia chaffeensis wakulla Staphylococcus aureus Mycobacterium tuberculosis Aeromonas cavia* | 7.47E-001 | 0 | 1.00E+000 | 6.69E-001 | 6.69E-001 | 8.60E-001 | 7.84E-001 | 7.55E-001 | 1.00E+000 | 5.61E-001 | 7.13E-001 | 5.03E-001 | 1.00E+000 | 3.82E-001 |
| 1104 | 6 | 7 | *Yersinia enterocolitica wap bl6 Yersinia enterocolitica p60 bc streptococcus gordonii Porphyromonas gingivalis Mycobacterium tuberculosis Aeromonas cavia* | 7.47E-001 | 0 | 1.00E+000 | 6.69E-001 | 6.69E-001 | 8.60E-001 | 7.84E-001 | 7.55E-001 | 1.00E+000 | 2.16E-001 | 3.61E-001 | 8.60E-001 | 9.10E-002 | 1.00E+000 |
| 1118 | 6 | 7 | *Helicobacter pylori kx2 npgec Lactobacillus acidophilus Mycobacterium tuberculosis Helicobacter pylori kx2 mgep Aeromonas cavia Helicobacter pylori kx1 npgec* | 7.47E-001 | 0 | 1.00E+000 | 9.90E-001 | 9.18E-002 | 8.60E-001 | 7.84E-001 | 4.13E-001 | 3.78E-001 | 8.87E-001 | 1.08E-001 | 8.60E-001 | 1.00E+000 | 1.00E+000 |
| 1124 | 6 | 7 | *Helicobacter pylori kx2 npgec Pseudomonas aeruginosa fdr1 Pseudomonas aeruginosa fdr1234 Helicobacter pylori kx2 mgep Aeromonas cavia Helicobacter pylori kx1 npgec* | 7.47E-001 | 2 | 6.40E-002 | 9.08E-001 | 3.31E-001 | 1.00E+000 | 1.13E-001 | 2.32E-002 | 1.00E+000 | 1.00E+000 | 1.08E-001 | 5.03E-001 | 1.00E+000 | 1.00E+000 |
| 660 | 5 | 16 | *Burkholderia pseudomallei Lactobacillus acidophilus Staphylococcus aureus Helicobacter pylori kx2 mgep Aggregatibacter actinomycetemcomitans* | 7.59E-001 | 2 | 2.83E-001 | 5.00E-001 | 8.28E-001 | 3.93E-001 | 8.69E-001 | 2.75E-001 | 3.23E-001 | 8.32E-001 | 9.05E-001 | 8.00E-001 | 4.10E-001 | 7.21E-001 |
| 658 | 5 | 16 | *Burkholderia pseudomallei Yersinia enterocolitica p60 bc Pseudomonas aeruginosa Helicobacter pylori kx1 npgec Aggregatibacter actinomycetemcomitans* | 7.59E-001 | 0 | 1.00E+000 | 5.00E-001 | 8.28E-001 | 1.00E+000 | 1.68E-001 | 6.41E-001 | 1.00E+000 | 4.46E-001 | 5.96E-001 | 8.00E-001 | 4.10E-001 | 7.21E-001 |
| 674 | 5 | 16 | *Helicobacter pylori kx2 npgec streptococcus gordonii Pseudomonas aeruginosa Aeromonas cavia Helicobacter pylori kx1 npgec* | 7.59E-001 | 0 | 1.00E+000 | 8.28E-001 | 5.00E-001 | 8.00E-001 | 5.54E-001 | 2.75E-001 | 1.00E+000 | 1.00E+000 | 2.35E-001 | 8.00E-001 | 4.10E-001 | 1.00E+000 |
| 668 | 5 | 16 | *Lactobacillus acidophilus Pseudomonas aeruginosa fdr440 Porphyromonas gingivalis Helicobacter pylori kx1 npgec Ehrlichia chaffeensis liberty* | 7.59E-001 | 0 | 1.00E+000 | 8.28E-001 | 5.00E-001 | 8.00E-001 | 5.54E-001 | 2.75E-001 | 3.23E-001 | 1.00E+000 | 9.05E-001 | 8.00E-001 | 4.10E-001 | 7.21E-001 |
| 676 | 5 | 16 | *Pseudomonas aeruginosa fdr1 Pseudomonas aeruginosa fdr1234 Staphylococcus aureus Aeromonas cavia Helicobacter pylori kx1 npgec* | 7.59E-001 | 13 | 1.04E-007 | 5.00E-001 | 8.28E-001 | 8.00E-001 | 5.54E-001 | 6.08E-002 | 1.00E+000 | 1.00E+000 | 5.96E-001 | 1.03E-001 | 1.00E+000 | 1.00E+000 |
| 250 | 4 | 46 | *Helicobacter pylori kx2 npgec Staphylococcus aureus Pseudomonas aeruginosa Ehrlichia chaffeensis liberty* | 7.60E-001 | 24 | 5.84E-008 | 6.97E-001 | 6.97E-001 | 7.19E-001 | 6.80E-001 | 4.95E-001 | 1.00E+000 | 1.00E+000 | 8.43E-001 | 2.77E-001 | 1.00E+000 | 6.34E-001 |
| 706 | 5 | 15 | *Lactobacillus acidophilus Pseudomonas aeruginosa fdr440 Pseudomonas aeruginosa Porphyromonas gingivalis streptococcus pneumoniae d39* | 7.77E-001 | 1 | 4.73E-001 | 1.72E-001 | 9.76E-001 | 3.93E-001 | 8.69E-001 | 2.75E-001 | 3.23E-001 | 1.00E+000 | 1.00E+000 | 1.03E-001 | 4.10E-001 | 1.00E+000 |
| 708 | 5 | 15 | *Pseudomonas aeruginosa fdr440 Listeria monocytogenes Mycobacterium tuberculosis streptococcus pneumoniae d39 Helicobacter pylori kx1 npgec* | 7.77E-001 | 4 | 5.84E-005 | 1.72E-001 | 9.76E-001 | 3.93E-001 | 9.82E-001 | 2.75E-001 | 3.23E-001 | 8.32E-001 | 5.96E-001 | 1.03E-001 | 1.00E+000 | 1.00E+000 |
| 710 | 5 | 15 | *Helicobacter pylori kx2 npgec Pseudomonas aeruginosa fdr1 Listeria monocytogenes Porphyromonas gingivalis Helicobacter pylori kx1 npgec* | 7.77E-001 | 5 | 1.50E-003 | 5.00E-001 | 8.28E-001 | 8.00E-001 | 5.54E-001 | 6.08E-002 | 3.23E-001 | 1.00E+000 | 2.35E-001 | 8.00E-001 | 4.10E-001 | 1.00E+000 |
| 270 | 4 | 43 | *Yersinia enterocolitica wap bl6 Yersinia enterocolitica p60 bl6 Yersinia enterocolitica p60 bc Ehrlichia chaffeensis wakulla* | 7.83E-001 | 79 | 1.01E-011 | 1.00E+000 | 5.35E-002 | 1.00E+000 | 2.45E-001 | 1.00E+000 | 1.00E+000 | 6.34E-002 | 1.22E-001 | 1.00E+000 | 1.00E+000 | 6.34E-001 |
| 266 | 4 | 43 | *Yersinia enterocolitica wap bl6 Helicobacter pylori kx2 npgec Porphyromonas gingivalis Ehrlichia chaffeensis liberty* | 7.83E-001 | 48 | 5.88E-013 | 9.47E-001 | 3.03E-001 | 1.00E+000 | 2.45E-001 | 4.95E-001 | 1.00E+000 | 7.55E-001 | 4.51E-001 | 1.00E+000 | 3.41E-001 | 6.34E-001 |
| 268 | 4 | 43 | *Helicobacter pylori kx2 npgec Burkholderia pseudomallei Ehrlichia chaffeensis wakulla Staphylococcus aureus* | 7.83E-001 | 25 | 7.02E-009 | 6.97E-001 | 6.97E-001 | 7.19E-001 | 6.80E-001 | 4.95E-001 | 1.00E+000 | 7.55E-001 | 8.43E-001 | 7.19E-001 | 1.00E+000 | 1.96E-001 |
| 82 | 3 | 124 | *Helicobacter pylori kx2 npgec Ehrlichia chaffeensis wakulla Helicobacter pylori kx1 npgec* | 7.84E-001 | 126 | < 1.67E-026 | 1.00E+000 | 1.16E-001 | 1.00E+000 | 3.54E-001 | 3.21E-001 | 1.00E+000 | 1.00E+000 | 2.87E-001 | 1.00E+000 | 1.00E+000 | 5.25E-001 |
| 1448 | 9 | 3 | *Yersinia enterocolitica wap bl6 Helicobacter pylori kx2 npgec Burkholderia pseudomallei Yersinia enterocolitica p60 bc Staphylococcus aureus Listeria monocytogenes Aeromonas cavia Helicobacter pylori kx1 npgec Ehrlichia chaffeensis liberty* | 7.88E-001 | 0 | 1.00E+000 | 9.35E-001 | 2.27E-001 | 7.60E-001 | 4.91E-001 | 7.60E-001 | 5.25E-001 | 5.09E-001 | 3.83E-002 | 9.55E-001 | 1.00E+000 | 6.33E-001 |
| 1438 | 9 | 3 | *Yersinia enterocolitica wap bl6 Burkholderia pseudomallei Lactobacillus acidophilus Bifidobacterium bifidum Aeromonas cavia streptococcus pneumoniae d39 Helicobacter pylori kx1 npgec Ehrlichia chaffeensis liberty Ehrlichia chaffeensis arkansa* | 7.88E-001 | 0 | 1.00E+000 | 9.90E-001 | 6.50E-002 | 4.36E-001 | 7.84E-001 | 9.37E-001 | 1.11E-001 | 8.10E-001 | 7.06E-001 | 9.55E-001 | 1.00E+000 | 2.88E-001 |
| 1460 | 9 | 3 | *Yersinia enterocolitica wap bl6 Helicobacter pylori kx2 npgec Burkholderia pseudomallei Lactobacillus acidophilus Bifidobacterium bifidum Porphyromonas gingivalis Aggregatibacter actinomycetemcomitans Ehrlichia chaffeensis wakulla Pseudomonas aeruginosa fdr1* | 7.88E-001 | 0 | 1.00E+000 | 7.73E-001 | 5.00E-001 | 7.60E-001 | 4.91E-001 | 4.71E-001 | 1.11E-001 | 8.10E-001 | 9.15E-001 | 9.55E-001 | 1.96E-001 | 6.33E-001 |
| 1446 | 9 | 3 | *Yersinia enterocolitica wap bl6 Yersinia enterocolitica wap bc Burkholderia pseudomallei Yersinia enterocolitica p60 bl6 Yersinia enterocolitica p60 bc Porphyromonas gingivalis Aeromonas cavia streptococcus pneumoniae d39 Mycobacterium tuberculosis* | 7.88E-001 | 0 | 1.00E+000 | 7.73E-001 | 5.00E-001 | 9.55E-001 | 4.91E-001 | 9.37E-001 | 1.00E+000 | 9.22E-003 | 1.56E-001 | 7.60E-001 | 6.34E-001 | 9.14E-001 |
| 1450 | 9 | 3 | *Yersinia enterocolitica wap bl6 Helicobacter pylori kx2 npgec Burkholderia pseudomallei Porphyromonas gingivalis streptococcus pneumoniae d39 Helicobacter pylori kx1 npgec Aggregatibacter actinomycetemcomitans Mycobacterium tuberculosis Escherichia coli* | 7.88E-001 | 0 | 1.00E+000 | 2.27E-001 | 9.35E-001 | 9.55E-001 | 4.91E-001 | 2.02E-001 | 1.00E+000 | 2.16E-001 | 4.04E-001 | 7.60E-001 | 1.96E-001 | 9.14E-001 |
| 1442 | 9 | 3 | *Helicobacter pylori kx2 npgec Lactobacillus acidophilus Bifidobacterium bifidum Pseudomonas aeruginosa Porphyromonas gingivalis streptococcus pneumoniae d39 Helicobacter pylori kx1 npgec Ehrlichia chaffeensis liberty Mycobacterium tuberculosis* | 7.88E-001 | 0 | 1.00E+000 | 7.73E-001 | 5.00E-001 | 4.36E-001 | 9.42E-001 | 4.71E-001 | 1.11E-001 | 9.68E-001 | 9.15E-001 | 4.36E-001 | 6.34E-001 | 9.14E-001 |
| 1452 | 9 | 3 | *Yersinia enterocolitica wap bl6 Helicobacter pylori kx2 npgec Yersinia enterocolitica p60 bc Staphylococcus aureus Porphyromonas gingivalis Fusobacterium nucleatum Helicobacter pylori kx1 npgec Aggregatibacter actinomycetemcomitans Escherichia coli* | 7.88E-001 | 0 | 1.00E+000 | 5.00E-001 | 7.73E-001 | 9.55E-001 | 1.90E-001 | 5.58E-002 | 1.00E+000 | 5.09E-001 | 1.56E-001 | 9.55E-001 | 2.59E-002 | 1.00E+000 |
| 1454 | 9 | 3 | *Yersinia enterocolitica wap bl6 Helicobacter pylori kx2 npgec Yersinia enterocolitica p60 bc Lactobacillus acidophilus Staphylococcus aureus Bifidobacterium bifidum Helicobacter pylori kx1 npgec Ehrlichia chaffeensis liberty Mycobacterium tuberculosis* | 7.88E-001 | 0 | 1.00E+000 | 9.90E-001 | 6.50E-002 | 4.36E-001 | 9.42E-001 | 7.60E-001 | 1.11E-001 | 5.09E-001 | 4.04E-001 | 7.60E-001 | 1.00E+000 | 9.14E-001 |
| 1462 | 9 | 3 | *Helicobacter pylori kx2 npgec Lactobacillus acidophilus Porphyromonas gingivalis Aeromonas cavia streptococcus pneumoniae d39 Helicobacter pylori kx1 npgec Aggregatibacter actinomycetemcomitans Pseudomonas aeruginosa fdr440 Mycobacterium tuberculosis* | 7.88E-001 | 0 | 1.00E+000 | 5.00E-001 | 7.73E-001 | 7.60E-001 | 7.84E-001 | 5.58E-002 | 5.25E-001 | 9.68E-001 | 7.06E-001 | 4.36E-001 | 1.96E-001 | 1.00E+000 |
| 1456 | 9 | 3 | *Yersinia enterocolitica wap bl6 Lactobacillus acidophilus Staphylococcus aureus Pseudomonas aeruginosa Aeromonas cavia streptococcus pneumoniae d39 Aggregatibacter actinomycetemcomitans Pseudomonas aeruginosa fdr440 Escherichia coli* | 7.88E-001 | 0 | 1.00E+000 | 2.27E-001 | 9.35E-001 | 4.36E-001 | 7.84E-001 | 4.71E-001 | 5.25E-001 | 8.10E-001 | 7.06E-001 | 1.63E-001 | 6.34E-001 | 1.00E+000 |
| 1444 | 9 | 3 | *Helicobacter pylori kx2 npgec Staphylococcus aureus Listeria monocytogenes Pseudomonas aeruginosa streptococcus pneumoniae d39 Helicobacter pylori kx1 npgec Ehrlichia chaffeensis liberty Pseudomonas aeruginosa fdr440 Escherichia coli* | 7.88E-001 | 1 | 2.40E-001 | 2.27E-001 | 9.35E-001 | 4.36E-001 | 7.84E-001 | 2.02E-001 | 5.25E-001 | 9.68E-001 | 4.04E-001 | 1.63E-001 | 1.00E+000 | 9.14E-001 |
| 172 | 6 | 40 | *Helicobacter pylori kx2 npgec Lactobacillus acidophilus Porphyromonas gingivalis Aeromonas cavia streptococcus pneumoniae d39 Helicobacter pylori kx1 npgec* | 7.95E-002 | 4 | 2.07E-001 | 9.08E-001 | 3.31E-001 | 5.03E-001 | 7.84E-001 | 1.36E-001 | 3.78E-001 | 1.00E+000 | 3.61E-001 | 8.60E-001 | 4.74E-001 | 1.00E+000 |
| 758 | 5 | 14 | *Helicobacter pylori kx2 npgec Burkholderia pseudomallei Lactobacillus acidophilus Bifidobacterium bifidum streptococcus pyogenes* | 7.97E-001 | 18 | 1.17E-011 | 9.76E-001 | 1.72E-001 | 1.03E-001 | 9.82E-001 | 9.23E-001 | 3.31E-002 | 8.32E-001 | 9.05E-001 | 1.00E+000 | 1.00E+000 | 7.21E-001 |
| 752 | 5 | 14 | *Helicobacter pylori kx2 npgec Listeria monocytogenes Pseudomonas aeruginosa Helicobacter pylori kx1 npgec Escherichia coli* | 7.97E-001 | 6 | 4.44E-003 | 5.00E-001 | 8.28E-001 | 8.00E-001 | 5.54E-001 | 6.41E-001 | 3.23E-001 | 8.32E-001 | 4.69E-002 | 8.00E-001 | 1.00E+000 | 1.00E+000 |
| 756 | 5 | 14 | *Helicobacter pylori kx2 npgec Helicobacter pylori kx1 mgep Burkholderia pseudomallei Pseudomonas aeruginosa Helicobacter pylori kx1 npgec* | 7.97E-001 | 0 | 1.00E+000 | 8.28E-001 | 5.00E-001 | 1.00E+000 | 1.68E-001 | 2.75E-001 | 1.00E+000 | 8.32E-001 | 2.35E-001 | 8.00E-001 | 1.00E+000 | 7.21E-001 |
| 754 | 5 | 14 | *Yersinia enterocolitica wap bl6 Yersinia enterocolitica p60 bc Lactobacillus acidophilus Bifidobacterium bifidum Fusobacterium nucleatum* | 7.97E-001 | 7 | 5.00E-002 | 9.76E-001 | 1.72E-001 | 3.93E-001 | 8.69E-001 | 9.23E-001 | 3.31E-002 | 4.46E-001 | 5.96E-001 | 1.00E+000 | 4.10E-001 | 1.00E+000 |
| 748 | 5 | 14 | *Bifidobacterium bifidum Helicobacter pylori kx2 mgep Helicobacter pylori kx1 npgec Escherichia coli Ehrlichia chaffeensis liberty* | 7.97E-001 | 3 | 9.86E-002 | 9.76E-001 | 1.72E-001 | 8.00E-001 | 5.54E-001 | 6.41E-001 | 3.23E-001 | 8.32E-001 | 2.35E-001 | 1.00E+000 | 1.00E+000 | 7.21E-001 |
| 1190 | 6 | 6 | *Helicobacter pylori kx2 npgec Burkholderia pseudomallei Listeria monocytogenes streptococcus pneumoniae d39 Helicobacter pylori kx1 npgec Escherichia coli* | 8.00E-001 | 0 | 1.00E+000 | 3.31E-001 | 9.08E-001 | 5.03E-001 | 7.84E-001 | 4.13E-001 | 3.78E-001 | 5.61E-001 | 1.08E-001 | 8.60E-001 | 1.00E+000 | 7.89E-001 |
| 1202 | 6 | 6 | *Helicobacter pylori kx1 mgep Burkholderia pseudomallei Pseudomonas aeruginosa fdr440 Helicobacter pylori kx2 mgep Helicobacter pylori kx1 npgec Escherichia coli* | 8.00E-001 | 0 | 1.00E+000 | 6.69E-001 | 6.69E-001 | 1.00E+000 | 1.13E-001 | 1.36E-001 | 1.00E+000 | 5.61E-001 | 1.08E-001 | 8.60E-001 | 1.00E+000 | 7.89E-001 |
| 1232 | 6 | 6 | *Yersinia enterocolitica wap bl6 Burkholderia pseudomallei Yersinia enterocolitica p60 bl6 Yersinia enterocolitica p60 bc Ehrlichia chaffeensis wakulla Ehrlichia chaffeensis liberty* | 8.00E-001 | 8 | 3.38E-009 | 9.90E-001 | 9.18E-002 | 1.00E+000 | 1.13E-001 | 1.00E+000 | 1.00E+000 | 4.58E-002 | 3.61E-001 | 1.00E+000 | 1.00E+000 | 1.01E-001 |
| 1228 | 6 | 6 | *Yersinia enterocolitica p60 bc Pseudomonas aeruginosa fdr440 Pseudomonas aeruginosa streptococcus pneumoniae d39 Helicobacter pylori kx1 npgec Ehrlichia chaffeensis liberty* | 8.00E-001 | 2 | 9.05E-002 | 6.69E-001 | 6.69E-001 | 8.60E-001 | 4.39E-001 | 4.13E-001 | 1.00E+000 | 8.87E-001 | 7.13E-001 | 1.73E-001 | 1.00E+000 | 7.89E-001 |
| 1242 | 6 | 6 | *Yersinia enterocolitica wap bl6 Helicobacter pylori kx2 npgec Yersinia enterocolitica p60 bl6 Ehrlichia chaffeensis wakulla Helicobacter pylori kx1 npgec Escherichia coli* | 8.00E-001 | 11 | 2.02E-003 | 9.90E-001 | 9.18E-002 | 1.00E+000 | 1.13E-001 | 7.55E-001 | 1.00E+000 | 2.16E-001 | 1.64E-002 | 1.00E+000 | 1.00E+000 | 7.89E-001 |
| 1208 | 6 | 6 | *Helicobacter pylori kx2 npgec Helicobacter pylori kx1 mgep Burkholderia pseudomallei Helicobacter pylori kx2 mgep Helicobacter pylori kx1 npgec Aggregatibacter actinomycetemcomitans* | 8.00E-001 | 0 | 1.00E+000 | 9.08E-001 | 3.31E-001 | 1.00E+000 | 1.13E-001 | 2.32E-002 | 1.00E+000 | 8.87E-001 | 1.08E-001 | 1.00E+000 | 4.74E-001 | 7.89E-001 |
| 1198 | 6 | 6 | *Pseudomonas aeruginosa fdr440 Listeria monocytogenes Pseudomonas aeruginosa Mycobacterium tuberculosis Escherichia coli Aggregatibacter actinomycetemcomitans* | 8.00E-001 | 0 | 1.00E+000 | 1.03E-002 | 1.00E+000 | 8.60E-001 | 7.84E-001 | 7.55E-001 | 3.78E-001 | 5.61E-001 | 7.13E-001 | 1.73E-001 | 4.74E-001 | 1.00E+000 |
| 1236 | 6 | 6 | *Helicobacter pylori kx2 npgec Ehrlichia chaffeensis arkansa Burkholderia pseudomallei Lactobacillus acidophilus Bifidobacterium bifidum Mycobacterium tuberculosis* | 8.00E-001 | 6 | 2.88E-006 | 9.08E-001 | 3.31E-001 | 5.03E-001 | 9.54E-001 | 9.56E-001 | 4.88E-002 | 5.61E-001 | 9.44E-001 | 8.60E-001 | 1.00E+000 | 3.82E-001 |
| 1176 | 6 | 6 | *Helicobacter pylori kx2 npgec Ehrlichia chaffeensis arkansa Pseudomonas aeruginosa Aeromonas cavia Helicobacter pylori kx1 npgec Escherichia coli* | 8.00E-001 | 0 | 1.00E+000 | 9.08E-001 | 3.31E-001 | 1.00E+000 | 1.13E-001 | 7.55E-001 | 1.00E+000 | 8.87E-001 | 1.08E-001 | 8.60E-001 | 1.00E+000 | 7.89E-001 |
| 1184 | 6 | 6 | *Burkholderia pseudomallei Yersinia enterocolitica p60 bc Lactobacillus acidophilus Pseudomonas aeruginosa fdr440 Staphylococcus aureus Mycobacterium tuberculosis* | 8.00E-001 | 0 | 1.00E+000 | 3.31E-001 | 9.08E-001 | 5.03E-001 | 9.54E-001 | 7.55E-001 | 3.78E-001 | 2.16E-001 | 9.44E-001 | 1.73E-001 | 1.00E+000 | 7.89E-001 |
| 1206 | 6 | 6 | *Helicobacter pylori kx2 npgec Helicobacter pylori kx1 mgep Staphylococcus aureus Pseudomonas aeruginosa Porphyromonas gingivalis Helicobacter pylori kx1 npgec* | 8.00E-001 | 0 | 1.00E+000 | 6.69E-001 | 6.69E-001 | 8.60E-001 | 4.39E-001 | 2.32E-002 | 1.00E+000 | 1.00E+000 | 3.61E-001 | 5.03E-001 | 4.74E-001 | 1.00E+000 |
| 1222 | 6 | 6 | *Helicobacter pylori kx2 npgec Mycobacterium tuberculosis Fusobacterium nucleatum streptococcus pneumoniae d39 Helicobacter pylori kx1 npgec Escherichia coli* | 8.00E-001 | 0 | 1.00E+000 | 3.31E-001 | 9.08E-001 | 8.60E-001 | 7.84E-001 | 1.36E-001 | 1.00E+000 | 5.61E-001 | 3.61E-001 | 5.03E-001 | 4.74E-001 | 1.00E+000 |
| 1194 | 6 | 6 | *Yersinia enterocolitica wap bc Burkholderia pseudomallei Pseudomonas aeruginosa fdr440 Porphyromonas gingivalis Mycobacterium tuberculosis Helicobacter pylori kx1 npgec* | 8.00E-001 | 0 | 1.00E+000 | 3.31E-001 | 9.08E-001 | 1.00E+000 | 4.39E-001 | 4.13E-001 | 1.00E+000 | 2.16E-001 | 7.13E-001 | 5.03E-001 | 4.74E-001 | 7.89E-001 |
| 1192 | 6 | 6 | *Burkholderia pseudomallei Staphylococcus aureus Listeria monocytogenes streptococcus pneumoniae d39 Helicobacter pylori kx1 npgec Ehrlichia chaffeensis liberty* | 8.00E-001 | 0 | 1.00E+000 | 3.31E-001 | 9.08E-001 | 1.73E-001 | 9.54E-001 | 4.13E-001 | 3.78E-001 | 8.87E-001 | 7.13E-001 | 5.03E-001 | 1.00E+000 | 3.82E-001 |
| 1212 | 6 | 6 | *Helicobacter pylori kx2 npgec Ehrlichia chaffeensis arkansa Ehrlichia chaffeensis wakulla Mycobacterium tuberculosis Helicobacter pylori kx1 npgec Ehrlichia chaffeensis liberty* | 8.00E-001 | 0 | 1.00E+000 | 9.90E-001 | 9.18E-002 | 1.00E+000 | 4.39E-001 | 7.55E-001 | 1.00E+000 | 8.87E-001 | 7.13E-001 | 8.60E-001 | 1.00E+000 | 1.01E-001 |
| 1226 | 6 | 6 | *Helicobacter pylori kx2 npgec Burkholderia pseudomallei Pseudomonas aeruginosa fdr875 Pseudomonas aeruginosa Porphyromonas gingivalis Helicobacter pylori kx1 npgec* | 8.00E-001 | 0 | 1.00E+000 | 3.31E-001 | 9.08E-001 | 1.00E+000 | 1.13E-001 | 1.36E-001 | 1.00E+000 | 8.87E-001 | 7.13E-001 | 5.03E-001 | 4.74E-001 | 7.89E-001 |
| 288 | 4 | 40 | *Burkholderia pseudomallei Pseudomonas aeruginosa fdr1 Aeromonas cavia Helicobacter pylori kx1 npgec* | 8.04E-001 | 25 | 2.89E-012 | 6.97E-001 | 6.97E-001 | 1.00E+000 | 2.45E-001 | 4.95E-001 | 1.00E+000 | 7.55E-001 | 4.51E-001 | 7.19E-001 | 1.00E+000 | 6.34E-001 |
| 282 | 4 | 40 | *Ehrlichia chaffeensis arkansa Burkholderia pseudomallei Lactobacillus acidophilus Aeromonas cavia* | 8.04E-001 | 19 | 6.07E-014 | 9.47E-001 | 3.03E-001 | 7.19E-001 | 6.80E-001 | 1.00E+000 | 2.65E-001 | 7.55E-001 | 8.43E-001 | 1.00E+000 | 1.00E+000 | 1.96E-001 |
| 298 | 4 | 39 | *Pseudomonas aeruginosa fdr440 Listeria monocytogenes Helicobacter pylori kx1 npgec Escherichia coli* | 8.11E-001 | 9 | 1.21E-005 | 3.03E-001 | 9.47E-001 | 7.19E-001 | 6.80E-001 | 4.95E-001 | 2.65E-001 | 7.55E-001 | 1.22E-001 | 7.19E-001 | 1.00E+000 | 1.00E+000 |
| 290 | 4 | 39 | *Helicobacter pylori kx2 npgec Burkholderia pseudomallei Yersinia enterocolitica p60 bc Pseudomonas aeruginosa* | 8.11E-001 | 7 | 3.12E-001 | 6.97E-001 | 6.97E-001 | 1.00E+000 | 2.45E-001 | 8.66E-001 | 1.00E+000 | 3.20E-001 | 4.51E-001 | 7.19E-001 | 1.00E+000 | 6.34E-001 |
| 314 | 4 | 38 | *Yersinia enterocolitica wap bl6 Helicobacter pylori kx2 npgec Mycobacterium tuberculosis Escherichia coli* | 8.16E-001 | 10 | 1.10E-001 | 6.97E-001 | 6.97E-001 | 1.00E+000 | 6.80E-001 | 8.66E-001 | 1.00E+000 | 6.34E-002 | 1.22E-001 | 7.19E-001 | 1.00E+000 | 1.00E+000 |
| 318 | 4 | 38 | *Burkholderia pseudomallei Lactobacillus acidophilus Pseudomonas aeruginosa fdr440 Aeromonas cavia* | 8.16E-001 | 4 | 1.70E-001 | 6.97E-001 | 6.97E-001 | 7.19E-001 | 6.80E-001 | 8.66E-001 | 2.65E-001 | 7.55E-001 | 8.43E-001 | 7.19E-001 | 1.00E+000 | 6.34E-001 |
| 784 | 5 | 13 | *Lactobacillus acidophilus Pseudomonas aeruginosa fdr440 Bifidobacterium bifidum Porphyromonas gingivalis Helicobacter pylori kx2 mgep* | 8.16E-001 | 0 | 1.00E+000 | 8.28E-001 | 5.00E-001 | 3.93E-001 | 8.69E-001 | 2.75E-001 | 3.31E-002 | 1.00E+000 | 9.05E-001 | 8.00E-001 | 4.10E-001 | 1.00E+000 |
| 788 | 5 | 13 | *Pseudomonas aeruginosa fdr1 Pseudomonas aeruginosa fdr440 Porphyromonas gingivalis Mycobacterium tuberculosis Helicobacter pylori kx2 mgep* | 8.16E-001 | 1 | 8.08E-001 | 1.72E-001 | 9.76E-001 | 1.00E+000 | 5.54E-001 | 6.08E-002 | 1.00E+000 | 8.32E-001 | 9.05E-001 | 1.03E-001 | 4.10E-001 | 1.00E+000 |
| 782 | 5 | 13 | *Bifidobacterium bifidum Aeromonas cavia streptococcus pneumoniae d39 Helicobacter pylori kx1 npgec Ehrlichia chaffeensis liberty* | 8.16E-001 | 6 | 1.21E-001 | 9.76E-001 | 1.72E-001 | 3.93E-001 | 8.69E-001 | 6.41E-001 | 3.23E-001 | 1.00E+000 | 5.96E-001 | 8.00E-001 | 1.00E+000 | 7.21E-001 |
| 786 | 5 | 13 | *Yersinia enterocolitica wap bl6 Yersinia enterocolitica p60 bc Pseudomonas aeruginosa fdr440 Aeromonas cavia Helicobacter pylori kx1 npgec* | 8.16E-001 | 11 | 2.23E-004 | 9.76E-001 | 1.72E-001 | 1.00E+000 | 1.68E-001 | 6.41E-001 | 1.00E+000 | 4.46E-001 | 4.69E-002 | 8.00E-001 | 1.00E+000 | 1.00E+000 |
| 144 | 2 | 136 | *Ehrlichia chaffeensis wakulla Aeromonas cavia* | 8.19E-001 | 213 | < 1.67E-026 | 1.00E+000 | 2.44E-001 | 1.00E+000 | 5.05E-001 | 1.00E+000 | 1.00E+000 | 1.00E+000 | 5.92E-001 | 1.00E+000 | 1.00E+000 | 3.87E-001 |
| 332 | 4 | 37 | *Yersinia enterocolitica wap bl6 Lactobacillus acidophilus Pseudomonas aeruginosa Porphyromonas gingivalis* | 8.23E-001 | 18 | 1.79E-004 | 6.97E-001 | 6.97E-001 | 7.19E-001 | 6.80E-001 | 8.66E-001 | 2.65E-001 | 7.55E-001 | 8.43E-001 | 7.19E-001 | 3.41E-001 | 1.00E+000 |
| 348 | 4 | 36 | *Burkholderia pseudomallei Helicobacter pylori kx1 npgec Escherichia coli Ehrlichia chaffeensis liberty* | 8.31E-001 | 18 | 9.23E-007 | 6.97E-001 | 6.97E-001 | 1.00E+000 | 2.45E-001 | 8.66E-001 | 1.00E+000 | 3.20E-001 | 4.51E-001 | 1.00E+000 | 1.00E+000 | 1.96E-001 |
| 846 | 5 | 12 | *Yersinia enterocolitica wap bl6 Helicobacter pylori kx2 npgec Burkholderia pseudomallei Listeria monocytogenes Helicobacter pylori kx1 npgec* | 8.36E-001 | 0 | 1.00E+000 | 8.28E-001 | 5.00E-001 | 8.00E-001 | 5.54E-001 | 6.41E-001 | 3.23E-001 | 4.46E-001 | 4.69E-002 | 1.00E+000 | 1.00E+000 | 7.21E-001 |
| 866 | 5 | 12 | *Burkholderia pseudomallei Pseudomonas aeruginosa fdr1234 Lactobacillus acidophilus Pseudomonas aeruginosa fdr440 Mycobacterium tuberculosis* | 8.36E-001 | 0 | 1.00E+000 | 1.72E-001 | 9.76E-001 | 8.00E-001 | 8.69E-001 | 6.41E-001 | 3.23E-001 | 4.46E-001 | 1.00E+000 | 1.03E-001 | 1.00E+000 | 7.21E-001 |
| 838 | 5 | 12 | *Yersinia enterocolitica wap bl6 Yersinia enterocolitica p60 bl6 Yersinia enterocolitica p60 bc Pseudomonas aeruginosa Mycobacterium tuberculosis* | 8.36E-001 | 1 | 6.00E-001 | 8.28E-001 | 5.00E-001 | 1.00E+000 | 5.54E-001 | 1.00E+000 | 1.00E+000 | 1.84E-002 | 2.35E-001 | 3.93E-001 | 1.00E+000 | 1.00E+000 |
| 848 | 5 | 12 | *Yersinia enterocolitica wap bl6 Yersinia enterocolitica wap bc Yersinia enterocolitica p60 bc Porphyromonas gingivalis Escherichia coli* | 8.36E-001 | 0 | 1.00E+000 | 8.28E-001 | 5.00E-001 | 1.00E+000 | 1.68E-001 | 9.23E-001 | 1.00E+000 | 1.84E-002 | 4.69E-002 | 1.00E+000 | 4.10E-001 | 1.00E+000 |
| 864 | 5 | 12 | *Ehrlichia chaffeensis arkansa Burkholderia pseudomallei Ehrlichia chaffeensis wakulla Bifidobacterium bifidum Porphyromonas gingivalis* | 8.36E-001 | 2 | 4.07E-002 | 8.28E-001 | 5.00E-001 | 8.00E-001 | 5.54E-001 | 9.23E-001 | 3.23E-001 | 8.32E-001 | 1.00E+000 | 1.00E+000 | 4.10E-001 | 5.72E-002 |
| 840 | 5 | 12 | *Yersinia enterocolitica wap bl6 Lactobacillus acidophilus Staphylococcus aureus Pseudomonas aeruginosa Mycobacterium tuberculosis* | 8.36E-001 | 0 | 1.00E+000 | 5.00E-001 | 8.28E-001 | 3.93E-001 | 9.82E-001 | 9.23E-001 | 3.23E-001 | 4.46E-001 | 9.05E-001 | 1.03E-001 | 1.00E+000 | 1.00E+000 |
| 366 | 4 | 35 | *Pseudomonas aeruginosa fdr440 Porphyromonas gingivalis Escherichia coli Ehrlichia chaffeensis liberty* | 8.37E-001 | 16 | 1.55E-003 | 3.03E-001 | 9.47E-001 | 1.00E+000 | 2.45E-001 | 4.95E-001 | 1.00E+000 | 7.55E-001 | 8.43E-001 | 7.19E-001 | 3.41E-001 | 6.34E-001 |
| 362 | 4 | 35 | *Lactobacillus acidophilus Pseudomonas aeruginosa fdr440 Bifidobacterium bifidum Aggregatibacter actinomycetemcomitans* | 8.37E-001 | 0 | 1.00E+000 | 6.97E-001 | 6.97E-001 | 2.77E-001 | 9.37E-001 | 4.95E-001 | 2.02E-002 | 1.00E+000 | 1.00E+000 | 7.19E-001 | 3.41E-001 | 1.00E+000 |
| 374 | 4 | 34 | *Yersinia enterocolitica wap bc Bifidobacterium bifidum Porphyromonas gingivalis Mycobacterium tuberculosis* | 8.42E-001 | 9 | 2.32E-004 | 6.97E-001 | 6.97E-001 | 7.19E-001 | 9.37E-001 | 8.66E-001 | 2.65E-001 | 3.20E-001 | 8.43E-001 | 7.19E-001 | 3.41E-001 | 1.00E+000 |
| 376 | 4 | 34 | *Burkholderia pseudomallei Pseudomonas aeruginosa fdr1234 Lactobacillus acidophilus Bifidobacterium bifidum* | 8.42E-001 | 20 | 2.86E-015 | 6.97E-001 | 6.97E-001 | 2.77E-001 | 9.37E-001 | 8.66E-001 | 2.02E-002 | 7.55E-001 | 1.00E+000 | 7.19E-001 | 1.00E+000 | 6.34E-001 |
| 1408 | 7 | 4 | *Staphylococcus aureus Listeria monocytogenes Pseudomonas aeruginosa Mycobacterium tuberculosis Helicobacter pylori kx2 mgep Helicobacter pylori kx1 npgec Escherichia coli* | 8.49E-001 | 0 | 1.00E+000 | 2.05E-001 | 9.53E-001 | 6.02E-001 | 9.12E-001 | 5.46E-001 | 4.30E-001 | 6.60E-001 | 1.92E-001 | 2.56E-001 | 1.00E+000 | 1.00E+000 |
| 1430 | 7 | 4 | *Helicobacter pylori kx2 npgec Helicobacter pylori kx1 mgep Lactobacillus acidophilus streptococcus pyogenes Helicobacter pylori kx2 mgep Aeromonas cavia Helicobacter pylori kx1 npgec* | 8.49E-001 | 0 | 1.00E+000 | 1.00E+000 | 4.31E-003 | 6.02E-001 | 6.88E-001 | 2.36E-001 | 4.30E-001 | 1.00E+000 | 4.43E-002 | 1.00E+000 | 1.00E+000 | 1.00E+000 |
| 1424 | 7 | 4 | *Burkholderia pseudomallei Lactobacillus acidophilus Pseudomonas aeruginosa fdr875 Pseudomonas aeruginosa fdr440 Porphyromonas gingivalis Mycobacterium tuberculosis Helicobacter pylori kx2 mgep* | 8.49E-001 | 0 | 1.00E+000 | 2.05E-001 | 9.53E-001 | 9.03E-001 | 6.88E-001 | 2.36E-001 | 4.30E-001 | 6.60E-001 | 9.67E-001 | 2.56E-001 | 5.32E-001 | 8.42E-001 |
| 1400 | 7 | 4 | *Helicobacter pylori kx2 npgec Ehrlichia chaffeensis arkansa Pseudomonas aeruginosa streptococcus pneumoniae d39 Helicobacter pylori kx1 npgec Ehrlichia chaffeensis liberty Aggregatibacter actinomycetemcomitans* | 8.49E-001 | 1 | 8.75E-002 | 7.95E-001 | 5.00E-001 | 9.03E-001 | 3.40E-001 | 2.36E-001 | 1.00E+000 | 1.00E+000 | 8.03E-001 | 6.02E-001 | 5.32E-001 | 4.72E-001 |
| 1420 | 7 | 4 | *Burkholderia pseudomallei Pseudomonas aeruginosa fdr1 Pseudomonas aeruginosa fdr440 Porphyromonas gingivalis Helicobacter pylori kx2 mgep Aeromonas cavia Aggregatibacter actinomycetemcomitans* | 8.49E-001 | 0 | 1.00E+000 | 2.05E-001 | 9.53E-001 | 1.00E+000 | 7.55E-002 | 6.08E-002 | 1.00E+000 | 9.25E-001 | 8.03E-001 | 6.02E-001 | 1.23E-001 | 8.42E-001 |
| 1426 | 7 | 4 | *Yersinia enterocolitica wap bl6 Burkholderia pseudomallei Yersinia enterocolitica p60 bl6 Porphyromonas gingivalis Helicobacter pylori kx2 mgep Ehrlichia chaffeensis liberty Aggregatibacter actinomycetemcomitans* | 8.49E-001 | 0 | 1.00E+000 | 7.95E-001 | 5.00E-001 | 1.00E+000 | 7.55E-002 | 5.46E-001 | 1.00E+000 | 3.12E-001 | 4.88E-001 | 1.00E+000 | 1.23E-001 | 4.72E-001 |
| 1434 | 7 | 4 | *Helicobacter pylori kx2 npgec Ehrlichia chaffeensis arkansa Ehrlichia chaffeensis wakulla Staphylococcus aureus streptococcus pyogenes Porphyromonas gingivalis Helicobacter pylori kx1 npgec* | 8.49E-001 | 1 | 4.23E-001 | 9.53E-001 | 2.05E-001 | 6.02E-001 | 6.88E-001 | 2.36E-001 | 1.00E+000 | 1.00E+000 | 8.03E-001 | 9.03E-001 | 5.32E-001 | 4.72E-001 |
| 1418 | 7 | 4 | *Helicobacter pylori kx2 npgec Yersinia enterocolitica p60 bl6 Pseudomonas aeruginosa Porphyromonas gingivalis Mycobacterium tuberculosis Aeromonas cavia streptococcus pneumoniae d39* | 8.49E-001 | 0 | 1.00E+000 | 5.00E-001 | 7.95E-001 | 9.03E-001 | 6.88E-001 | 5.46E-001 | 1.00E+000 | 6.60E-001 | 4.88E-001 | 2.56E-001 | 5.32E-001 | 1.00E+000 |
| 1432 | 7 | 4 | *Helicobacter pylori kx2 npgec Lactobacillus acidophilus Pseudomonas aeruginosa fdr440 Porphyromonas gingivalis Helicobacter pylori kx2 mgep Aeromonas cavia Helicobacter pylori kx1 npgec* | 8.49E-001 | 0 | 1.00E+000 | 9.53E-001 | 2.05E-001 | 9.03E-001 | 3.40E-001 | 6.08E-002 | 4.30E-001 | 1.00E+000 | 1.92E-001 | 9.03E-001 | 5.32E-001 | 1.00E+000 |
| 1412 | 7 | 4 | *Yersinia enterocolitica wap bl6 Helicobacter pylori kx2 npgec Staphylococcus aureus Listeria monocytogenes Helicobacter pylori kx2 mgep Aeromonas cavia Helicobacter pylori kx1 npgec* | 8.49E-001 | 0 | 1.00E+000 | 9.53E-001 | 2.05E-001 | 6.02E-001 | 6.88E-001 | 2.36E-001 | 4.30E-001 | 9.25E-001 | 5.25E-003 | 9.03E-001 | 1.00E+000 | 1.00E+000 |
| 1422 | 7 | 4 | *Helicobacter pylori kx2 npgec Ehrlichia chaffeensis arkansa Ehrlichia chaffeensis wakulla streptococcus pyogenes Helicobacter pylori kx2 mgep Helicobacter pylori kx1 npgec Escherichia coli* | 8.49E-001 | 6 | 4.44E-004 | 9.96E-001 | 4.65E-002 | 9.03E-001 | 3.40E-001 | 5.46E-001 | 1.00E+000 | 9.25E-001 | 1.92E-001 | 1.00E+000 | 1.00E+000 | 4.72E-001 |
| 650 | 9 | 9 | *Yersinia enterocolitica wap bl6 Helicobacter pylori kx2 npgec Burkholderia pseudomallei Yersinia enterocolitica p60 bl6 Yersinia enterocolitica p60 bc Porphyromonas gingivalis Aeromonas cavia Helicobacter pylori kx1 npgec Ehrlichia chaffeensis wakulla* | 8.50E-003 | 0 | 1.00E+000 | 9.90E-001 | 6.50E-002 | 1.00E+000 | 3.21E-002 | 7.60E-001 | 1.00E+000 | 2.16E-001 | 3.83E-002 | 1.00E+000 | 6.34E-001 | 6.33E-001 |
| 648 | 9 | 9 | *Burkholderia pseudomallei Lactobacillus acidophilus Listeria monocytogenes Bifidobacterium bifidum Porphyromonas gingivalis Pseudomonas aeruginosa fdr1 Pseudomonas aeruginosa fdr440 Mycobacterium tuberculosis Escherichia coli* | 8.50E-003 | 0 | 1.00E+000 | 6.50E-002 | 9.90E-001 | 4.36E-001 | 9.42E-001 | 7.60E-001 | 7.32E-003 | 5.09E-001 | 9.15E-001 | 4.36E-001 | 6.34E-001 | 9.14E-001 |
| 654 | 9 | 9 | *Helicobacter pylori kx2 npgec Burkholderia pseudomallei Lactobacillus acidophilus Pseudomonas aeruginosa fdr440 Bifidobacterium bifidum Porphyromonas gingivalis Aeromonas cavia Helicobacter pylori kx1 npgec Escherichia coli* | 8.50E-003 | 0 | 1.00E+000 | 7.73E-001 | 5.00E-001 | 7.60E-001 | 4.91E-001 | 4.71E-001 | 1.11E-001 | 8.10E-001 | 4.04E-001 | 9.55E-001 | 6.34E-001 | 9.14E-001 |
| 170 | 3 | 81 | *Pseudomonas aeruginosa fdr440 streptococcus pneumoniae d39 Escherichia coli* | 8.51E-001 | 31 | 1.38E-001 | 1.16E-001 | 1.00E+000 | 6.08E-001 | 8.08E-001 | 3.21E-001 | 1.00E+000 | 6.46E-001 | 7.45E-001 | 1.63E-001 | 1.00E+000 | 1.00E+000 |
| 1356 | 6 | 5 | *Yersinia enterocolitica wap bc Yersinia enterocolitica p60 bc Staphylococcus aureus Porphyromonas gingivalis Helicobacter pylori kx1 npgec Ehrlichia chaffeensis liberty* | 8.54E-001 | 0 | 1.00E+000 | 9.08E-001 | 3.31E-001 | 8.60E-001 | 4.39E-001 | 4.13E-001 | 1.00E+000 | 5.61E-001 | 3.61E-001 | 8.60E-001 | 4.74E-001 | 7.89E-001 |
| 1390 | 6 | 5 | *Yersinia enterocolitica wap bc Burkholderia pseudomallei Bifidobacterium bifidum Porphyromonas gingivalis streptococcus pneumoniae d39 Aggregatibacter actinomycetemcomitans* | 8.54E-001 | 0 | 1.00E+000 | 3.31E-001 | 9.08E-001 | 5.03E-001 | 7.84E-001 | 4.13E-001 | 3.78E-001 | 5.61E-001 | 9.44E-001 | 8.60E-001 | 9.10E-002 | 7.89E-001 |
| 1380 | 6 | 5 | *Lactobacillus acidophilus Pseudomonas aeruginosa fdr440 Bifidobacterium bifidum Fusobacterium nucleatum streptococcus pneumoniae d39 Helicobacter pylori kx1 npgec* | 8.54E-001 | 0 | 1.00E+000 | 6.69E-001 | 6.69E-001 | 1.73E-001 | 9.54E-001 | 1.36E-001 | 4.88E-002 | 1.00E+000 | 9.44E-001 | 5.03E-001 | 4.74E-001 | 1.00E+000 |
| 1382 | 6 | 5 | *Helicobacter pylori kx2 npgec Burkholderia pseudomallei Bifidobacterium bifidum streptococcus pyogenes Aeromonas cavia Ehrlichia chaffeensis liberty* | 8.54E-001 | 2 | 7.65E-003 | 9.90E-001 | 9.18E-002 | 5.03E-001 | 7.84E-001 | 9.56E-001 | 3.78E-001 | 8.87E-001 | 7.13E-001 | 1.00E+000 | 1.00E+000 | 3.82E-001 |
| 1360 | 6 | 5 | *Yersinia enterocolitica wap bl6 Helicobacter pylori kx2 npgec Burkholderia pseudomallei Fusobacterium nucleatum Helicobacter pylori kx1 npgec Ehrlichia chaffeensis liberty* | 8.54E-001 | 0 | 1.00E+000 | 9.08E-001 | 3.31E-001 | 1.00E+000 | 1.13E-001 | 4.13E-001 | 1.00E+000 | 5.61E-001 | 3.61E-001 | 1.00E+000 | 4.74E-001 | 3.82E-001 |
| 1350 | 6 | 5 | *Helicobacter pylori kx2 npgec Yersinia enterocolitica p60 bc Helicobacter pylori kx2 mgep Helicobacter pylori kx1 npgec Escherichia coli Aggregatibacter actinomycetemcomitans* | 8.54E-001 | 2 | 1.83E-001 | 9.08E-001 | 3.31E-001 | 1.00E+000 | 1.13E-001 | 1.36E-001 | 1.00E+000 | 5.61E-001 | 1.64E-002 | 1.00E+000 | 4.74E-001 | 1.00E+000 |
| 182 | 6 | 39 | *Yersinia enterocolitica wap bl6 Yersinia enterocolitica p60 bc Pseudomonas aeruginosa Porphyromonas gingivalis Helicobacter pylori kx1 npgec Aggregatibacter actinomycetemcomitans* | 8.54E-002 | 6 | 5.61E-002 | 6.69E-001 | 6.69E-001 | 1.00E+000 | 1.13E-001 | 4.13E-001 | 1.00E+000 | 5.61E-001 | 3.61E-001 | 8.60E-001 | 9.10E-002 | 1.00E+000 |
| 392 | 4 | 32 | *Lactobacillus acidophilus Pseudomonas aeruginosa fdr440 Bifidobacterium bifidum streptococcus pneumoniae d39* | 8.56E-001 | 4 | 1.65E-003 | 6.97E-001 | 6.97E-001 | 4.86E-002 | 9.96E-001 | 4.95E-001 | 2.02E-002 | 1.00E+000 | 1.00E+000 | 2.77E-001 | 1.00E+000 | 1.00E+000 |
| 394 | 4 | 32 | *Lactobacillus acidophilus Mycobacterium tuberculosis Fusobacterium nucleatum streptococcus pneumoniae d39* | 8.56E-001 | 0 | 1.00E+000 | 3.03E-001 | 9.47E-001 | 2.77E-001 | 9.96E-001 | 4.95E-001 | 2.65E-001 | 7.55E-001 | 1.00E+000 | 2.77E-001 | 3.41E-001 | 1.00E+000 |
| 390 | 4 | 32 | *Yersinia enterocolitica wap bl6 Ehrlichia chaffeensis arkansa Burkholderia pseudomallei Ehrlichia chaffeensis liberty* | 8.56E-001 | 35 | 3.38E-023 | 9.47E-001 | 3.03E-001 | 1.00E+000 | 2.45E-001 | 1.00E+000 | 1.00E+000 | 3.20E-001 | 8.43E-001 | 1.00E+000 | 1.00E+000 | 2.59E-002 |
| 926 | 5 | 11 | *Burkholderia pseudomallei Yersinia enterocolitica p60 bc Mycobacterium tuberculosis Helicobacter pylori kx1 npgec Ehrlichia chaffeensis liberty* | 8.56E-001 | 0 | 1.00E+000 | 8.28E-001 | 5.00E-001 | 1.00E+000 | 5.54E-001 | 9.23E-001 | 1.00E+000 | 1.31E-001 | 5.96E-001 | 8.00E-001 | 1.00E+000 | 2.88E-001 |
| 920 | 5 | 11 | *Ehrlichia chaffeensis arkansa Burkholderia pseudomallei Helicobacter pylori kx2 mgep Helicobacter pylori kx1 npgec Escherichia coli* | 8.56E-001 | 0 | 1.00E+000 | 8.28E-001 | 5.00E-001 | 1.00E+000 | 1.68E-001 | 6.41E-001 | 1.00E+000 | 4.46E-001 | 2.35E-001 | 1.00E+000 | 1.00E+000 | 2.88E-001 |
| 922 | 5 | 11 | *Burkholderia pseudomallei Pseudomonas aeruginosa fdr1 Staphylococcus aureus Bifidobacterium bifidum Ehrlichia chaffeensis liberty* | 8.56E-001 | 0 | 1.00E+000 | 5.00E-001 | 8.28E-001 | 3.93E-001 | 8.69E-001 | 6.41E-001 | 3.23E-001 | 8.32E-001 | 1.00E+000 | 3.93E-001 | 1.00E+000 | 2.88E-001 |
| 928 | 5 | 11 | *Listeria monocytogenes Porphyromonas gingivalis streptococcus pneumoniae d39 Helicobacter pylori kx1 npgec Escherichia coli* | 8.56E-001 | 2 | 6.40E-002 | 1.72E-001 | 9.76E-001 | 3.93E-001 | 8.69E-001 | 2.75E-001 | 3.23E-001 | 8.32E-001 | 2.35E-001 | 8.00E-001 | 4.10E-001 | 1.00E+000 |
| 924 | 5 | 11 | *Burkholderia pseudomallei Lactobacillus acidophilus Listeria monocytogenes Bifidobacterium bifidum Pseudomonas aeruginosa* | 8.56E-001 | 0 | 1.00E+000 | 5.00E-001 | 8.28E-001 | 1.03E-001 | 9.82E-001 | 1.00E+000 | 8.71E-004 | 8.32E-001 | 9.05E-001 | 8.00E-001 | 1.00E+000 | 7.21E-001 |
| 184 | 3 | 78 | *Ehrlichia chaffeensis wakulla Lactobacillus acidophilus Bifidobacterium bifidum* | 8.61E-001 | 81 | < 1.67E-026 | 1.00E+000 | 1.16E-001 | 1.63E-001 | 9.81E-001 | 1.00E+000 | 1.03E-002 | 1.00E+000 | 1.00E+000 | 1.00E+000 | 1.00E+000 | 5.25E-001 |
| 444 | 7 | 17 | *Helicobacter pylori kx2 npgec Pseudomonas aeruginosa fdr1 Pseudomonas aeruginosa fdr440 Pseudomonas aeruginosa Porphyromonas gingivalis streptococcus pneumoniae d39 Helicobacter pylori kx1 npgec* | 8.61E-002 | 1 | 4.23E-001 | 2.05E-001 | 9.53E-001 | 9.03E-001 | 3.40E-001 | 8.14E-003 | 1.00E+000 | 1.00E+000 | 8.03E-001 | 6.35E-002 | 5.32E-001 | 1.00E+000 |
| 418 | 4 | 31 | *Helicobacter pylori kx2 npgec Ehrlichia chaffeensis wakulla Pseudomonas aeruginosa Helicobacter pylori kx1 npgec* | 8.62E-001 | 35 | 1.12E-011 | 9.47E-001 | 3.03E-001 | 1.00E+000 | 2.45E-001 | 4.95E-001 | 1.00E+000 | 1.00E+000 | 4.51E-001 | 7.19E-001 | 1.00E+000 | 6.34E-001 |
| 452 | 4 | 29 | *Helicobacter pylori kx2 npgec Ehrlichia chaffeensis arkansa Helicobacter pylori kx1 npgec Escherichia coli* | 8.74E-001 | 23 | 3.63E-006 | 9.47E-001 | 3.03E-001 | 1.00E+000 | 2.45E-001 | 4.95E-001 | 1.00E+000 | 7.55E-001 | 1.22E-001 | 1.00E+000 | 1.00E+000 | 6.34E-001 |
| 134 | 3 | 94 | *Porphyromonas gingivalis Escherichia coli Aggregatibacter actinomycetemcomitans* | 8.74E-001 | 27 | 9.43E-002 | 1.16E-001 | 1.00E+000 | 1.00E+000 | 3.54E-001 | 3.21E-001 | 1.00E+000 | 6.46E-001 | 7.45E-001 | 1.00E+000 | 2.02E-002 | 1.00E+000 |
| 978 | 5 | 10 | *Lactobacillus acidophilus Listeria monocytogenes Bifidobacterium bifidum Porphyromonas gingivalis Aeromonas cavia* | 8.76E-001 | 4 | 5.13E-003 | 8.28E-001 | 5.00E-001 | 1.03E-001 | 9.82E-001 | 9.23E-001 | 8.71E-004 | 1.00E+000 | 5.96E-001 | 1.00E+000 | 4.10E-001 | 1.00E+000 |
| 968 | 5 | 10 | *Burkholderia pseudomallei Listeria monocytogenes Porphyromonas gingivalis Aeromonas cavia Ehrlichia chaffeensis liberty* | 8.76E-001 | 1 | 4.23E-001 | 5.00E-001 | 8.28E-001 | 8.00E-001 | 5.54E-001 | 9.23E-001 | 3.23E-001 | 8.32E-001 | 5.96E-001 | 1.00E+000 | 4.10E-001 | 2.88E-001 |
| 982 | 5 | 10 | *streptococcus gordonii Staphylococcus aureus Mycobacterium tuberculosis Aeromonas cavia Helicobacter pylori kx1 npgec* | 8.76E-001 | 0 | 1.00E+000 | 5.00E-001 | 8.28E-001 | 3.93E-001 | 9.82E-001 | 2.75E-001 | 1.00E+000 | 8.32E-001 | 5.96E-001 | 3.93E-001 | 4.10E-001 | 1.00E+000 |
| 966 | 5 | 10 | *Helicobacter pylori kx2 npgec Yersinia enterocolitica p60 bl6 Staphylococcus aureus Pseudomonas aeruginosa Escherichia coli* | 8.76E-001 | 0 | 1.00E+000 | 5.00E-001 | 8.28E-001 | 8.00E-001 | 5.54E-001 | 6.41E-001 | 1.00E+000 | 4.46E-001 | 2.35E-001 | 3.93E-001 | 1.00E+000 | 1.00E+000 |
| 976 | 5 | 10 | *Helicobacter pylori kx2 npgec Ehrlichia chaffeensis arkansa Burkholderia pseudomallei Ehrlichia chaffeensis wakulla Escherichia coli* | 8.76E-001 | 0 | 1.00E+000 | 8.28E-001 | 5.00E-001 | 1.00E+000 | 1.68E-001 | 9.23E-001 | 1.00E+000 | 4.46E-001 | 5.96E-001 | 1.00E+000 | 1.00E+000 | 5.72E-002 |
| 970 | 5 | 10 | *Burkholderia pseudomallei Lactobacillus acidophilus Staphylococcus aureus Mycobacterium tuberculosis Helicobacter pylori kx2 mgep* | 8.76E-001 | 0 | 1.00E+000 | 5.00E-001 | 8.28E-001 | 3.93E-001 | 9.82E-001 | 6.41E-001 | 3.23E-001 | 4.46E-001 | 9.05E-001 | 3.93E-001 | 1.00E+000 | 7.21E-001 |
| 986 | 5 | 10 | *Yersinia enterocolitica wap bl6 Burkholderia pseudomallei Pseudomonas aeruginosa fdr1234 Porphyromonas gingivalis Aggregatibacter actinomycetemcomitans* | 8.76E-001 | 0 | 1.00E+000 | 1.72E-001 | 9.76E-001 | 1.00E+000 | 1.68E-001 | 2.75E-001 | 1.00E+000 | 4.46E-001 | 9.05E-001 | 8.00E-001 | 6.29E-002 | 7.21E-001 |
| 964 | 5 | 10 | *Yersinia enterocolitica wap bl6 Helicobacter pylori kx2 npgec Yersinia enterocolitica p60 bl6 Mycobacterium tuberculosis streptococcus pneumoniae d39* | 8.76E-001 | 0 | 1.00E+000 | 8.28E-001 | 5.00E-001 | 8.00E-001 | 8.69E-001 | 6.41E-001 | 1.00E+000 | 1.31E-001 | 2.35E-001 | 3.93E-001 | 1.00E+000 | 1.00E+000 |
| 974 | 5 | 10 | *Helicobacter pylori kx2 npgec Burkholderia pseudomallei streptococcus pyogenes Pseudomonas aeruginosa Helicobacter pylori kx1 npgec* | 8.76E-001 | 7 | 2.71E-003 | 8.28E-001 | 5.00E-001 | 8.00E-001 | 5.54E-001 | 6.41E-001 | 1.00E+000 | 8.32E-001 | 5.96E-001 | 8.00E-001 | 1.00E+000 | 7.21E-001 |
| 980 | 5 | 10 | *Pseudomonas aeruginosa fdr1 Pseudomonas aeruginosa fdr1234 Mycobacterium tuberculosis Aeromonas cavia Escherichia coli* | 8.76E-001 | 0 | 1.00E+000 | 1.72E-001 | 9.76E-001 | 1.00E+000 | 5.54E-001 | 6.41E-001 | 1.00E+000 | 4.46E-001 | 5.96E-001 | 1.03E-001 | 1.00E+000 | 1.00E+000 |
| 972 | 5 | 10 | *Helicobacter pylori kx2 npgec streptococcus gordonii streptococcus pneumoniae d39 Helicobacter pylori kx1 npgec Ehrlichia chaffeensis liberty* | 8.76E-001 | 5 | 3.94E-001 | 8.28E-001 | 5.00E-001 | 3.93E-001 | 8.69E-001 | 6.08E-002 | 1.00E+000 | 1.00E+000 | 5.96E-001 | 8.00E-001 | 4.10E-001 | 7.21E-001 |
| 108 | 3 | 105 | *Pseudomonas aeruginosa Helicobacter pylori kx2 mgep Helicobacter pylori kx1 npgec* | 8.76E-001 | 55 | 3.73E-012 | 8.84E-001 | 5.00E-001 | 1.00E+000 | 3.54E-001 | 3.21E-001 | 1.00E+000 | 1.00E+000 | 2.87E-001 | 6.08E-001 | 1.00E+000 | 1.00E+000 |
| 132 | 3 | 97 | *Porphyromonas gingivalis Mycobacterium tuberculosis streptococcus pneumoniae d39* | 8.81E-001 | 28 | 2.74E-001 | 1.16E-001 | 1.00E+000 | 6.08E-001 | 9.81E-001 | 3.21E-001 | 1.00E+000 | 6.46E-001 | 1.00E+000 | 1.63E-001 | 2.65E-001 | 1.00E+000 |
| 126 | 3 | 100 | *Yersinia enterocolitica wap bl6 Helicobacter pylori kx2 npgec Pseudomonas aeruginosa* | 8.82E-001 | 46 | 1.48E-008 | 8.84E-001 | 5.00E-001 | 1.00E+000 | 3.54E-001 | 7.74E-001 | 1.00E+000 | 6.46E-001 | 2.87E-001 | 6.08E-001 | 1.00E+000 | 1.00E+000 |
| 468 | 4 | 28 | *Ehrlichia chaffeensis arkansa Staphylococcus aureus Aeromonas cavia Helicobacter pylori kx1 npgec* | 8.82E-001 | 41 | 1.67E-026 | 9.47E-001 | 3.03E-001 | 7.19E-001 | 6.80E-001 | 4.95E-001 | 1.00E+000 | 1.00E+000 | 4.51E-001 | 7.19E-001 | 1.00E+000 | 6.34E-001 |
| 474 | 4 | 28 | *Helicobacter pylori kx1 mgep Helicobacter pylori kx2 mgep Aeromonas cavia Helicobacter pylori kx1 npgec* | 8.82E-001 | 19 | 1.73E-005 | 1.00E+000 | 5.35E-002 | 1.00E+000 | 2.45E-001 | 1.46E-001 | 1.00E+000 | 1.00E+000 | 1.22E-002 | 1.00E+000 | 1.00E+000 | 1.00E+000 |
| 472 | 4 | 28 | *Pseudomonas aeruginosa fdr440 Staphylococcus aureus Aeromonas cavia Ehrlichia chaffeensis liberty* | 8.82E-001 | 22 | 3.55E-008 | 6.97E-001 | 6.97E-001 | 7.19E-001 | 6.80E-001 | 4.95E-001 | 1.00E+000 | 1.00E+000 | 8.43E-001 | 2.77E-001 | 1.00E+000 | 6.34E-001 |
| 152 | 3 | 88 | *Helicobacter pylori kx2 npgec Helicobacter pylori kx2 mgep streptococcus pneumoniae d39* | 8.83E-001 | 57 | 2.29E-004 | 8.84E-001 | 5.00E-001 | 6.08E-001 | 8.08E-001 | 4.88E-002 | 1.00E+000 | 1.00E+000 | 2.87E-001 | 6.08E-001 | 1.00E+000 | 1.00E+000 |
| 142 | 3 | 91 | *Helicobacter pylori kx2 npgec Pseudomonas aeruginosa Mycobacterium tuberculosis* | 8.83E-001 | 21 | 3.90E-004 | 5.00E-001 | 8.84E-001 | 1.00E+000 | 8.08E-001 | 7.74E-001 | 1.00E+000 | 6.46E-001 | 7.45E-001 | 1.63E-001 | 1.00E+000 | 1.00E+000 |
| 6 | 4 | 238 | *Helicobacter pylori kx2 npgec Staphylococcus aureus Porphyromonas gingivalis Helicobacter pylori kx1 npgec* | 8.83E-002 | 79 | 5.44E-010 | 6.97E-001 | 6.97E-001 | 7.19E-001 | 6.80E-001 | 1.63E-002 | 1.00E+000 | 1.00E+000 | 4.51E-001 | 7.19E-001 | 3.41E-001 | 1.00E+000 |
| 158 | 3 | 86 | *Pseudomonas aeruginosa streptococcus pneumoniae d39 Aggregatibacter actinomycetemcomitans* | 8.91E-001 | 21 | 2.27E-001 | 1.16E-001 | 1.00E+000 | 6.08E-001 | 8.08E-001 | 3.21E-001 | 1.00E+000 | 1.00E+000 | 1.00E+000 | 1.63E-001 | 2.65E-001 | 1.00E+000 |
| 124 | 3 | 101 | *Helicobacter pylori kx2 npgec streptococcus gordonii Helicobacter pylori kx1 npgec* | 8.94E-001 | 24 | 1.70E-001 | 8.84E-001 | 5.00E-001 | 6.08E-001 | 8.08E-001 | 4.88E-002 | 1.00E+000 | 1.00E+000 | 2.87E-001 | 1.00E+000 | 2.65E-001 | 1.00E+000 |
| 122 | 3 | 101 | *Helicobacter pylori kx2 npgec Pseudomonas aeruginosa fdr1 Helicobacter pylori kx1 npgec* | 8.94E-001 | 96 | < 1.67E-026 | 8.84E-001 | 5.00E-001 | 1.00E+000 | 3.54E-001 | 4.88E-002 | 1.00E+000 | 1.00E+000 | 2.87E-001 | 6.08E-001 | 1.00E+000 | 1.00E+000 |
| 1056 | 5 | 9 | *Yersinia enterocolitica wap bl6 Burkholderia pseudomallei Ehrlichia chaffeensis wakulla Porphyromonas gingivalis Ehrlichia chaffeensis liberty* | 8.96E-001 | 1 | 7.89E-001 | 8.28E-001 | 5.00E-001 | 1.00E+000 | 1.68E-001 | 9.23E-001 | 1.00E+000 | 4.46E-001 | 9.05E-001 | 1.00E+000 | 4.10E-001 | 5.72E-002 |
| 1062 | 5 | 9 | *Yersinia enterocolitica wap bl6 Pseudomonas aeruginosa Fusobacterium nucleatum streptococcus pneumoniae d39 Helicobacter pylori kx1 npgec* | 8.96E-001 | 0 | 1.00E+000 | 5.00E-001 | 8.28E-001 | 8.00E-001 | 5.54E-001 | 2.75E-001 | 1.00E+000 | 8.32E-001 | 5.96E-001 | 3.93E-001 | 4.10E-001 | 1.00E+000 |
| 1064 | 5 | 9 | *Pseudomonas aeruginosa fdr1 Pseudomonas aeruginosa fdr1234 Lactobacillus acidophilus Pseudomonas aeruginosa fdr440 Bifidobacterium bifidum* | 8.96E-001 | 0 | 1.00E+000 | 5.00E-001 | 8.28E-001 | 3.93E-001 | 8.69E-001 | 2.75E-001 | 3.31E-002 | 1.00E+000 | 1.00E+000 | 1.03E-001 | 1.00E+000 | 1.00E+000 |
| 1054 | 5 | 9 | *Helicobacter pylori kx2 npgec Ehrlichia chaffeensis wakulla Helicobacter pylori kx2 mgep streptococcus pneumoniae d39 Helicobacter pylori kx1 npgec* | 8.96E-001 | 9 | 3.90E-002 | 9.76E-001 | 1.72E-001 | 8.00E-001 | 5.54E-001 | 6.08E-002 | 1.00E+000 | 1.00E+000 | 2.35E-001 | 8.00E-001 | 1.00E+000 | 7.21E-001 |
| 1058 | 5 | 9 | *Yersinia enterocolitica p60 bc Pseudomonas aeruginosa fdr1 Staphylococcus aureus Porphyromonas gingivalis Mycobacterium tuberculosis* | 8.96E-001 | 0 | 1.00E+000 | 1.72E-001 | 9.76E-001 | 8.00E-001 | 8.69E-001 | 2.75E-001 | 1.00E+000 | 4.46E-001 | 9.05E-001 | 1.03E-001 | 4.10E-001 | 1.00E+000 |
| 1052 | 5 | 9 | *Helicobacter pylori kx2 npgec Lactobacillus acidophilus Anaplasma phagocytophilum Bifidobacterium bifidum Helicobacter pylori kx1 npgec* | 8.96E-001 | 30 | 8.61E-013 | 9.76E-001 | 1.72E-001 | 3.93E-001 | 8.69E-001 | 6.41E-001 | 3.31E-002 | 1.00E+000 | 5.96E-001 | 1.00E+000 | 1.00E+000 | 7.21E-001 |
| 1066 | 5 | 9 | *Helicobacter pylori kx2 npgec Burkholderia pseudomallei streptococcus pyogenes Helicobacter pylori kx1 npgec Escherichia coli* | 8.96E-001 | 0 | 1.00E+000 | 8.28E-001 | 5.00E-001 | 8.00E-001 | 5.54E-001 | 6.41E-001 | 1.00E+000 | 4.46E-001 | 2.35E-001 | 1.00E+000 | 1.00E+000 | 7.21E-001 |
| 494 | 4 | 26 | *Yersinia enterocolitica p60 bl6 Lactobacillus acidophilus Helicobacter pylori kx1 npgec Escherichia coli* | 8.96E-001 | 8 | 6.92E-004 | 9.47E-001 | 3.03E-001 | 7.19E-001 | 6.80E-001 | 8.66E-001 | 2.65E-001 | 3.20E-001 | 1.22E-001 | 1.00E+000 | 1.00E+000 | 1.00E+000 |
| 512 | 4 | 25 | *Yersinia enterocolitica p60 bc Pseudomonas aeruginosa Helicobacter pylori kx1 npgec Ehrlichia chaffeensis liberty* | 9.02E-001 | 17 | 1.94E-006 | 9.47E-001 | 3.03E-001 | 1.00E+000 | 2.45E-001 | 8.66E-001 | 1.00E+000 | 7.55E-001 | 4.51E-001 | 7.19E-001 | 1.00E+000 | 6.34E-001 |
| 514 | 4 | 25 | *Yersinia enterocolitica wap bl6 Pseudomonas aeruginosa fdr440 Porphyromonas gingivalis Mycobacterium tuberculosis* | 9.02E-001 | 7 | 1.23E-001 | 3.03E-001 | 9.47E-001 | 1.00E+000 | 6.80E-001 | 4.95E-001 | 1.00E+000 | 3.20E-001 | 8.43E-001 | 2.77E-001 | 3.41E-001 | 1.00E+000 |
| 510 | 4 | 25 | *Pseudomonas aeruginosa fdr1 Helicobacter pylori kx2 mgep Helicobacter pylori kx1 npgec Aggregatibacter actinomycetemcomitans* | 9.02E-001 | 17 | 9.72E-004 | 6.97E-001 | 6.97E-001 | 1.00E+000 | 2.45E-001 | 1.63E-002 | 1.00E+000 | 1.00E+000 | 4.51E-001 | 7.19E-001 | 3.41E-001 | 1.00E+000 |
| 518 | 4 | 25 | *Yersinia enterocolitica wap bc Helicobacter pylori kx2 npgec Burkholderia pseudomallei Helicobacter pylori kx1 npgec* | 9.02E-001 | 15 | 3.85E-010 | 9.47E-001 | 3.03E-001 | 1.00E+000 | 2.45E-001 | 4.95E-001 | 1.00E+000 | 3.20E-001 | 1.22E-001 | 1.00E+000 | 1.00E+000 | 6.34E-001 |
| 196 | 3 | 76 | *Mycobacterium tuberculosis Aeromonas cavia Escherichia coli* | 9.03E-001 | 27 | 6.66E-003 | 5.00E-001 | 8.84E-001 | 1.00E+000 | 8.08E-001 | 1.00E+000 | 1.00E+000 | 1.92E-001 | 2.87E-001 | 6.08E-001 | 1.00E+000 | 1.00E+000 |
| 192 | 6 | 38 | *Helicobacter pylori kx2 npgec Lactobacillus acidophilus Bifidobacterium bifidum Helicobacter pylori kx2 mgep Helicobacter pylori kx1 npgec Aggregatibacter actinomycetemcomitans* | 9.06E-002 | 10 | 3.66E-006 | 9.90E-001 | 9.18E-002 | 5.03E-001 | 7.84E-001 | 1.36E-001 | 4.88E-002 | 1.00E+000 | 3.61E-001 | 1.00E+000 | 4.74E-001 | 1.00E+000 |
| 198 | 6 | 38 | *Helicobacter pylori kx2 npgec Pseudomonas aeruginosa fdr440 Staphylococcus aureus Mycobacterium tuberculosis Helicobacter pylori kx1 npgec Aggregatibacter actinomycetemcomitans* | 9.06E-002 | 0 | 1.00E+000 | 3.31E-001 | 9.08E-001 | 8.60E-001 | 7.84E-001 | 2.32E-002 | 1.00E+000 | 8.87E-001 | 7.13E-001 | 1.73E-001 | 4.74E-001 | 1.00E+000 |
| 538 | 4 | 24 | *Ehrlichia chaffeensis arkansa Pseudomonas aeruginosa Helicobacter pylori kx1 npgec Ehrlichia chaffeensis liberty* | 9.09E-001 | 34 | 4.26E-013 | 9.47E-001 | 3.03E-001 | 1.00E+000 | 2.45E-001 | 8.66E-001 | 1.00E+000 | 1.00E+000 | 8.43E-001 | 7.19E-001 | 1.00E+000 | 1.96E-001 |
| 1546 | 8 | 3 | *Helicobacter pylori kx2 npgec Helicobacter pylori kx1 mgep Ehrlichia chaffeensis arkansa Ehrlichia chaffeensis wakulla Lactobacillus acidophilus Bifidobacterium bifidum Helicobacter pylori kx2 mgep Helicobacter pylori kx1 npgec* | 9.13E-001 | 0 | 1.00E+000 | 1.00E+000 | 1.72E-003 | 6.88E-001 | 5.89E-001 | 3.51E-001 | 8.78E-002 | 1.00E+000 | 2.94E-001 | 1.00E+000 | 1.00E+000 | 5.57E-001 |
| 1506 | 8 | 3 | *Helicobacter pylori kx2 npgec Burkholderia pseudomallei streptococcus gordonii Lactobacillus acidophilus Staphylococcus aureus Bifidobacterium bifidum Helicobacter pylori kx2 mgep Helicobacter pylori kx1 npgec* | 9.13E-001 | 0 | 1.00E+000 | 8.81E-001 | 3.48E-001 | 1.07E-001 | 9.69E-001 | 1.21E-001 | 8.78E-002 | 9.50E-001 | 6.05E-001 | 9.33E-001 | 5.86E-001 | 8.82E-001 |
| 1498 | 8 | 3 | *Yersinia enterocolitica wap bl6 Helicobacter pylori kx2 npgec Yersinia enterocolitica p60 bl6 Porphyromonas gingivalis Aeromonas cavia Helicobacter pylori kx1 npgec Ehrlichia chaffeensis liberty Escherichia coli* | 9.13E-001 | 0 | 1.00E+000 | 9.78E-001 | 1.19E-001 | 1.00E+000 | 4.96E-002 | 6.64E-001 | 1.00E+000 | 4.11E-001 | 1.64E-002 | 1.00E+000 | 5.86E-001 | 8.82E-001 |
| 1560 | 8 | 3 | *Helicobacter pylori kx2 npgec Burkholderia pseudomallei Lactobacillus acidophilus Helicobacter pylori kx1 npgec Pseudomonas aeruginosa fdr440 Mycobacterium tuberculosis streptococcus pyogenes Helicobacter pylori kx2 mgep* | 9.13E-001 | 0 | 1.00E+000 | 8.81E-001 | 3.48E-001 | 6.88E-001 | 8.54E-001 | 3.51E-001 | 4.79E-001 | 7.43E-001 | 6.05E-001 | 6.88E-001 | 1.00E+000 | 8.82E-001 |
| 1530 | 8 | 3 | *Burkholderia pseudomallei Lactobacillus acidophilus Staphylococcus aureus Bifidobacterium bifidum streptococcus pyogenes Aeromonas cavia Helicobacter pylori kx1 npgec Escherichia coli* | 9.13E-001 | 0 | 1.00E+000 | 8.81E-001 | 3.48E-001 | 1.07E-001 | 9.69E-001 | 8.98E-001 | 8.78E-002 | 7.43E-001 | 6.05E-001 | 9.33E-001 | 1.00E+000 | 8.82E-001 |
| 1522 | 8 | 3 | *Yersinia enterocolitica wap bl6 Burkholderia pseudomallei Lactobacillus acidophilus Staphylococcus aureus Pseudomonas aeruginosa Aeromonas cavia Pseudomonas aeruginosa fdr440 Mycobacterium tuberculosis* | 9.13E-001 | 0 | 1.00E+000 | 3.48E-001 | 8.81E-001 | 6.88E-001 | 8.54E-001 | 8.98E-001 | 4.79E-001 | 4.11E-001 | 8.68E-001 | 1.07E-001 | 1.00E+000 | 8.82E-001 |
| 1568 | 8 | 3 | *Helicobacter pylori kx2 npgec Burkholderia pseudomallei Yersinia enterocolitica p60 bl6 Lactobacillus acidophilus Bifidobacterium bifidum Mycobacterium tuberculosis Helicobacter pylori kx2 mgep Helicobacter pylori kx1 npgec* | 9.13E-001 | 0 | 1.00E+000 | 9.78E-001 | 1.19E-001 | 6.88E-001 | 8.54E-001 | 6.64E-001 | 8.78E-002 | 4.11E-001 | 2.94E-001 | 9.33E-001 | 1.00E+000 | 8.82E-001 |
| 1496 | 8 | 3 | *Yersinia enterocolitica wap bl6 Helicobacter pylori kx2 npgec Staphylococcus aureus Porphyromonas gingivalis Aeromonas cavia Helicobacter pylori kx1 npgec Ehrlichia chaffeensis wakulla Helicobacter pylori kx2 mgep* | 9.13E-001 | 3 | 5.03E-002 | 9.78E-001 | 1.19E-001 | 9.33E-001 | 2.57E-001 | 1.21E-001 | 1.00E+000 | 9.50E-001 | 9.08E-002 | 9.33E-001 | 5.86E-001 | 8.82E-001 |
| 1508 | 8 | 3 | *Helicobacter pylori kx2 npgec Helicobacter pylori kx1 mgep Burkholderia pseudomallei Lactobacillus acidophilus Bifidobacterium bifidum streptococcus pneumoniae d39 Helicobacter pylori kx1 npgec Helicobacter pylori kx2 mgep* | 9.13E-001 | 0 | 1.00E+000 | 9.78E-001 | 1.19E-001 | 3.45E-001 | 8.54E-001 | 1.21E-001 | 8.78E-002 | 9.50E-001 | 2.94E-001 | 9.33E-001 | 1.00E+000 | 8.82E-001 |
| 1566 | 8 | 3 | *Burkholderia pseudomallei Lactobacillus acidophilus Staphylococcus aureus Bifidobacterium bifidum Helicobacter pylori kx1 npgec Aggregatibacter actinomycetemcomitans Pseudomonas aeruginosa fdr1 Helicobacter pylori kx2 mgep* | 9.13E-001 | 0 | 1.00E+000 | 6.52E-001 | 6.52E-001 | 3.45E-001 | 8.54E-001 | 1.21E-001 | 8.78E-002 | 9.50E-001 | 8.68E-001 | 6.88E-001 | 5.86E-001 | 8.82E-001 |
| 1562 | 8 | 3 | *Helicobacter pylori kx2 npgec Lactobacillus acidophilus Staphylococcus aureus Aeromonas cavia streptococcus pneumoniae d39 Helicobacter pylori kx1 npgec Ehrlichia chaffeensis liberty Mycobacterium tuberculosis* | 9.13E-001 | 0 | 1.00E+000 | 8.81E-001 | 3.48E-001 | 3.45E-001 | 9.69E-001 | 3.51E-001 | 4.79E-001 | 9.50E-001 | 6.05E-001 | 3.45E-001 | 1.00E+000 | 8.82E-001 |
| 1486 | 8 | 3 | *Ehrlichia chaffeensis arkansa Lactobacillus acidophilus Pseudomonas aeruginosa fdr440 Bifidobacterium bifidum Helicobacter pylori kx2 mgep Helicobacter pylori kx1 npgec Escherichia coli Ehrlichia chaffeensis liberty* | 9.13E-001 | 0 | 1.00E+000 | 9.78E-001 | 1.19E-001 | 6.88E-001 | 5.89E-001 | 6.64E-001 | 8.78E-002 | 9.50E-001 | 6.05E-001 | 9.33E-001 | 1.00E+000 | 5.57E-001 |
| 1572 | 8 | 3 | *Yersinia enterocolitica wap bl6 Helicobacter pylori kx2 npgec Porphyromonas gingivalis streptococcus pneumoniae d39 Helicobacter pylori kx1 npgec Aggregatibacter actinomycetemcomitans streptococcus gordonii Pseudomonas aeruginosa fdr440* | 9.13E-001 | 0 | 1.00E+000 | 3.48E-001 | 8.81E-001 | 6.88E-001 | 5.89E-001 | 2.63E-003 | 1.00E+000 | 9.50E-001 | 6.05E-001 | 6.88E-001 | 1.76E-002 | 1.00E+000 |
| 1534 | 8 | 3 | *Helicobacter pylori kx2 npgec Ehrlichia chaffeensis arkansa Burkholderia pseudomallei Lactobacillus acidophilus Bifidobacterium bifidum streptococcus pyogenes Helicobacter pylori kx2 mgep Helicobacter pylori kx1 npgec* | 9.13E-001 | 0 | 1.00E+000 | 9.98E-001 | 2.24E-002 | 3.45E-001 | 8.54E-001 | 6.64E-001 | 8.78E-002 | 9.50E-001 | 6.05E-001 | 1.00E+000 | 1.00E+000 | 5.57E-001 |
| 1504 | 8 | 3 | *Helicobacter pylori kx2 npgec Pseudomonas aeruginosa fdr1 Pseudomonas aeruginosa fdr440 Listeria monocytogenes Porphyromonas gingivalis Helicobacter pylori kx2 mgep Helicobacter pylori kx1 npgec Escherichia coli* | 9.13E-001 | 0 | 1.00E+000 | 3.48E-001 | 8.81E-001 | 9.33E-001 | 2.57E-001 | 2.47E-002 | 4.79E-001 | 9.50E-001 | 9.08E-002 | 6.88E-001 | 5.86E-001 | 1.00E+000 |
| 1526 | 8 | 3 | *Yersinia enterocolitica wap bl6 Helicobacter pylori kx2 npgec Pseudomonas aeruginosa fdr875 Staphylococcus aureus Porphyromonas gingivalis streptococcus pneumoniae d39 Helicobacter pylori kx1 npgec Aggregatibacter actinomycetemcomitans* | 9.13E-001 | 0 | 1.00E+000 | 3.48E-001 | 8.81E-001 | 6.88E-001 | 5.89E-001 | 2.63E-003 | 1.00E+000 | 9.50E-001 | 6.05E-001 | 3.45E-001 | 1.58E-001 | 1.00E+000 |
| 1542 | 8 | 3 | *Yersinia enterocolitica wap bl6 Burkholderia pseudomallei Yersinia enterocolitica p60 bl6 Yersinia enterocolitica p60 bc Pseudomonas aeruginosa fdr1234 Porphyromonas gingivalis Aeromonas cavia Ehrlichia chaffeensis wakulla* | 9.13E-001 | 0 | 1.00E+000 | 8.81E-001 | 3.48E-001 | 1.00E+000 | 4.96E-002 | 8.98E-001 | 1.00E+000 | 1.46E-001 | 2.94E-001 | 9.33E-001 | 5.86E-001 | 5.57E-001 |
| 1564 | 8 | 3 | *Helicobacter pylori kx2 npgec Yersinia enterocolitica p60 bl6 Bifidobacterium bifidum Pseudomonas aeruginosa Helicobacter pylori kx2 mgep streptococcus pneumoniae d39 Helicobacter pylori kx1 npgec Ehrlichia chaffeensis liberty* | 9.13E-001 | 0 | 1.00E+000 | 9.78E-001 | 1.19E-001 | 6.88E-001 | 5.89E-001 | 3.51E-001 | 4.79E-001 | 9.50E-001 | 2.94E-001 | 6.88E-001 | 1.00E+000 | 8.82E-001 |
| 1538 | 8 | 3 | *Yersinia enterocolitica wap bl6 Burkholderia pseudomallei Staphylococcus aureus Bifidobacterium bifidum Pseudomonas aeruginosa Aeromonas cavia Ehrlichia chaffeensis wakulla Pseudomonas aeruginosa fdr440* | 9.13E-001 | 0 | 1.00E+000 | 6.52E-001 | 6.52E-001 | 6.88E-001 | 5.89E-001 | 8.98E-001 | 4.79E-001 | 7.43E-001 | 8.68E-001 | 3.45E-001 | 1.00E+000 | 5.57E-001 |
| 568 | 4 | 23 | *Helicobacter pylori kx2 npgec Yersinia enterocolitica p60 bc Pseudomonas aeruginosa fdr1 Aeromonas cavia* | 9.14E-001 | 21 | 5.18E-009 | 9.47E-001 | 3.03E-001 | 1.00E+000 | 2.45E-001 | 4.95E-001 | 1.00E+000 | 7.55E-001 | 1.22E-001 | 7.19E-001 | 1.00E+000 | 1.00E+000 |
| 566 | 4 | 23 | *Staphylococcus aureus Bifidobacterium bifidum Porphyromonas gingivalis Helicobacter pylori kx2 mgep* | 9.14E-001 | 14 | 1.34E-004 | 6.97E-001 | 6.97E-001 | 2.77E-001 | 9.37E-001 | 1.46E-001 | 2.65E-001 | 1.00E+000 | 8.43E-001 | 7.19E-001 | 3.41E-001 | 1.00E+000 |
| 564 | 4 | 23 | *Burkholderia pseudomallei Yersinia enterocolitica p60 bc Porphyromonas gingivalis Helicobacter pylori kx2 mgep* | 9.14E-001 | 17 | 4.69E-004 | 6.97E-001 | 6.97E-001 | 1.00E+000 | 2.45E-001 | 4.95E-001 | 1.00E+000 | 3.20E-001 | 4.51E-001 | 1.00E+000 | 3.41E-001 | 6.34E-001 |
| 562 | 4 | 23 | *Burkholderia pseudomallei Staphylococcus aureus Ehrlichia chaffeensis liberty Aggregatibacter actinomycetemcomitans* | 9.14E-001 | 10 | 3.35E-003 | 3.03E-001 | 9.47E-001 | 7.19E-001 | 6.80E-001 | 4.95E-001 | 1.00E+000 | 7.55E-001 | 1.00E+000 | 7.19E-001 | 3.41E-001 | 1.96E-001 |
| 1152 | 5 | 8 | *Burkholderia pseudomallei streptococcus gordonii Pseudomonas aeruginosa Porphyromonas gingivalis Fusobacterium nucleatum* | 9.15E-001 | 0 | 1.00E+000 | 2.39E-002 | 1.00E+000 | 8.00E-001 | 5.54E-001 | 2.75E-001 | 1.00E+000 | 8.32E-001 | 1.00E+000 | 8.00E-001 | 3.35E-003 | 7.21E-001 |
| 1130 | 5 | 8 | *Helicobacter pylori kx2 npgec Yersinia enterocolitica p60 bc Staphylococcus aureus Pseudomonas aeruginosa Mycobacterium tuberculosis* | 9.15E-001 | 0 | 1.00E+000 | 5.00E-001 | 8.28E-001 | 8.00E-001 | 8.69E-001 | 6.41E-001 | 1.00E+000 | 4.46E-001 | 5.96E-001 | 1.03E-001 | 1.00E+000 | 1.00E+000 |
| 1144 | 5 | 8 | *Ehrlichia chaffeensis arkansa Burkholderia pseudomallei Ehrlichia chaffeensis wakulla streptococcus pyogenes Ehrlichia chaffeensis liberty* | 9.15E-001 | 12 | 1.93E-013 | 9.76E-001 | 1.72E-001 | 8.00E-001 | 5.54E-001 | 1.00E+000 | 1.00E+000 | 8.32E-001 | 1.00E+000 | 1.00E+000 | 1.00E+000 | 5.04E-003 |
| 1148 | 5 | 8 | *Burkholderia pseudomallei Pseudomonas aeruginosa fdr1234 Lactobacillus acidophilus Porphyromonas gingivalis Ehrlichia chaffeensis liberty* | 9.15E-001 | 9 | 5.06E-008 | 5.00E-001 | 8.28E-001 | 8.00E-001 | 5.54E-001 | 6.41E-001 | 3.23E-001 | 8.32E-001 | 1.00E+000 | 8.00E-001 | 4.10E-001 | 2.88E-001 |
| 1154 | 5 | 8 | *streptococcus gordonii Pseudomonas aeruginosa fdr440 Pseudomonas aeruginosa streptococcus pneumoniae d39 Helicobacter pylori kx1 npgec* | 9.15E-001 | 0 | 1.00E+000 | 1.72E-001 | 9.76E-001 | 3.93E-001 | 8.69E-001 | 6.08E-002 | 1.00E+000 | 1.00E+000 | 9.05E-001 | 1.03E-001 | 4.10E-001 | 1.00E+000 |
| 1128 | 5 | 8 | *Burkholderia pseudomallei Yersinia enterocolitica p60 bc Lactobacillus acidophilus Mycobacterium tuberculosis Helicobacter pylori kx2 mgep* | 9.15E-001 | 0 | 1.00E+000 | 8.28E-001 | 5.00E-001 | 8.00E-001 | 8.69E-001 | 9.23E-001 | 3.23E-001 | 1.31E-001 | 5.96E-001 | 8.00E-001 | 1.00E+000 | 7.21E-001 |
| 1142 | 5 | 8 | *streptococcus gordonii Bifidobacterium bifidum Porphyromonas gingivalis Fusobacterium nucleatum Aggregatibacter actinomycetemcomitans* | 9.15E-001 | 5 | 1.04E-001 | 1.72E-001 | 9.76E-001 | 3.93E-001 | 8.69E-001 | 6.08E-002 | 3.23E-001 | 1.00E+000 | 1.00E+000 | 1.00E+000 | 4.47E-005 | 1.00E+000 |
| 604 | 4 | 22 | *Yersinia enterocolitica wap bl6 Burkholderia pseudomallei Lactobacillus acidophilus Pseudomonas aeruginosa fdr440* | 9.20E-001 | 5 | 5.20E-003 | 6.97E-001 | 6.97E-001 | 7.19E-001 | 6.80E-001 | 8.66E-001 | 2.65E-001 | 3.20E-001 | 8.43E-001 | 7.19E-001 | 1.00E+000 | 6.34E-001 |
| 600 | 4 | 22 | *Lactobacillus acidophilus Staphylococcus aureus Escherichia coli Ehrlichia chaffeensis liberty* | 9.20E-001 | 7 | 2.19E-002 | 6.97E-001 | 6.97E-001 | 2.77E-001 | 9.37E-001 | 8.66E-001 | 2.65E-001 | 7.55E-001 | 8.43E-001 | 7.19E-001 | 1.00E+000 | 6.34E-001 |
| 634 | 4 | 21 | *Pseudomonas aeruginosa fdr1 Porphyromonas gingivalis Helicobacter pylori kx2 mgep Aeromonas cavia* | 9.26E-001 | 7 | 3.40E-002 | 6.97E-001 | 6.97E-001 | 1.00E+000 | 2.45E-001 | 1.46E-001 | 1.00E+000 | 1.00E+000 | 4.51E-001 | 7.19E-001 | 3.41E-001 | 1.00E+000 |
| 640 | 4 | 21 | *Helicobacter pylori kx2 npgec Ehrlichia chaffeensis arkansa Ehrlichia chaffeensis wakulla streptococcus pyogenes* | 9.26E-001 | 37 | 9.44E-012 | 1.00E+000 | 5.35E-002 | 7.19E-001 | 6.80E-001 | 8.66E-001 | 1.00E+000 | 1.00E+000 | 8.43E-001 | 1.00E+000 | 1.00E+000 | 1.96E-001 |
| 662 | 4 | 20 | *Burkholderia pseudomallei Yersinia enterocolitica p60 bc Pseudomonas aeruginosa fdr440 Porphyromonas gingivalis* | 9.31E-001 | 2 | 8.67E-001 | 3.03E-001 | 9.47E-001 | 1.00E+000 | 2.45E-001 | 4.95E-001 | 1.00E+000 | 3.20E-001 | 8.43E-001 | 7.19E-001 | 3.41E-001 | 6.34E-001 |
| 672 | 4 | 20 | *streptococcus gordonii streptococcus pneumoniae d39 Helicobacter pylori kx1 npgec Escherichia coli* | 9.31E-001 | 4 | 7.59E-001 | 3.03E-001 | 9.47E-001 | 2.77E-001 | 9.37E-001 | 1.46E-001 | 1.00E+000 | 7.55E-001 | 4.51E-001 | 7.19E-001 | 3.41E-001 | 1.00E+000 |
| 664 | 4 | 20 | *Yersinia enterocolitica wap bl6 Pseudomonas aeruginosa fdr440 Pseudomonas aeruginosa streptococcus pneumoniae d39* | 9.31E-001 | 9 | 3.50E-002 | 3.03E-001 | 9.47E-001 | 7.19E-001 | 6.80E-001 | 4.95E-001 | 1.00E+000 | 7.55E-001 | 8.43E-001 | 4.86E-002 | 1.00E+000 | 1.00E+000 |
| 656 | 4 | 20 | *Helicobacter pylori kx2 npgec Helicobacter pylori kx1 mgep Helicobacter pylori kx1 npgec Ehrlichia chaffeensis liberty* | 9.31E-001 | 33 | 1.98E-011 | 1.00E+000 | 5.35E-002 | 1.00E+000 | 2.45E-001 | 1.46E-001 | 1.00E+000 | 1.00E+000 | 1.22E-001 | 1.00E+000 | 1.00E+000 | 6.34E-001 |
| 670 | 4 | 20 | *Pseudomonas aeruginosa fdr1 Pseudomonas aeruginosa fdr440 Staphylococcus aureus Mycobacterium tuberculosis* | 9.31E-001 | 3 | 3.76E-001 | 5.35E-002 | 1.00E+000 | 7.19E-001 | 9.37E-001 | 1.46E-001 | 1.00E+000 | 7.55E-001 | 1.00E+000 | 2.95E-003 | 1.00E+000 | 1.00E+000 |
| 1250 | 5 | 7 | *Ehrlichia chaffeensis arkansa Burkholderia pseudomallei Lactobacillus acidophilus Pseudomonas aeruginosa fdr440 Bifidobacterium bifidum* | 9.33E-001 | 3 | 6.68E-004 | 8.28E-001 | 5.00E-001 | 3.93E-001 | 8.69E-001 | 9.23E-001 | 3.31E-002 | 8.32E-001 | 1.00E+000 | 8.00E-001 | 1.00E+000 | 2.88E-001 |
| 1270 | 5 | 7 | *Burkholderia pseudomallei Lactobacillus acidophilus Pseudomonas aeruginosa fdr875 Pseudomonas aeruginosa fdr440 Helicobacter pylori kx1 npgec* | 9.33E-001 | 0 | 1.00E+000 | 5.00E-001 | 8.28E-001 | 8.00E-001 | 5.54E-001 | 2.75E-001 | 3.23E-001 | 8.32E-001 | 9.05E-001 | 3.93E-001 | 1.00E+000 | 7.21E-001 |
| 1266 | 5 | 7 | *Helicobacter pylori kx2 npgec Bifidobacterium bifidum Fusobacterium nucleatum Helicobacter pylori kx2 mgep Helicobacter pylori kx1 npgec* | 9.33E-001 | 0 | 1.00E+000 | 9.76E-001 | 1.72E-001 | 8.00E-001 | 5.54E-001 | 6.08E-002 | 3.23E-001 | 1.00E+000 | 2.35E-001 | 1.00E+000 | 4.10E-001 | 1.00E+000 |
| 1268 | 5 | 7 | *Helicobacter pylori kx2 npgec streptococcus pyogenes Porphyromonas gingivalis Helicobacter pylori kx1 npgec Ehrlichia chaffeensis liberty* | 9.33E-001 | 35 | < 1.67E-026 | 9.76E-001 | 1.72E-001 | 8.00E-001 | 5.54E-001 | 2.75E-001 | 1.00E+000 | 1.00E+000 | 5.96E-001 | 1.00E+000 | 4.10E-001 | 7.21E-001 |
| 1254 | 5 | 7 | *Yersinia enterocolitica p60 bc Bifidobacterium bifidum Mycobacterium tuberculosis streptococcus pneumoniae d39 Escherichia coli* | 9.33E-001 | 0 | 1.00E+000 | 5.00E-001 | 8.28E-001 | 3.93E-001 | 9.82E-001 | 9.23E-001 | 3.23E-001 | 1.31E-001 | 5.96E-001 | 3.93E-001 | 1.00E+000 | 1.00E+000 |
| 1262 | 5 | 7 | *Ehrlichia chaffeensis arkansa Helicobacter pylori kx2 mgep Aeromonas cavia streptococcus pneumoniae d39 Helicobacter pylori kx1 npgec* | 9.33E-001 | 0 | 1.00E+000 | 9.76E-001 | 1.72E-001 | 8.00E-001 | 5.54E-001 | 2.75E-001 | 1.00E+000 | 1.00E+000 | 2.35E-001 | 8.00E-001 | 1.00E+000 | 7.21E-001 |
| 1536 | 6 | 4 | *Helicobacter pylori kx2 npgec Ehrlichia chaffeensis arkansa Eubacterium rectale Ehrlichia chaffeensis wakulla Helicobacter pylori kx1 npgec Ehrlichia chaffeensis liberty* | 9.34E-001 | 0 | 1.00E+000 | 1.00E+000 | 1.03E-002 | 8.60E-001 | 4.39E-001 | 7.55E-001 | 1.00E+000 | 1.00E+000 | 3.61E-001 | 1.00E+000 | 1.00E+000 | 1.01E-001 |
| 1554 | 6 | 4 | *Helicobacter pylori kx2 npgec Pseudomonas aeruginosa fdr875 Bifidobacterium bifidum Porphyromonas gingivalis Helicobacter pylori kx1 npgec Aggregatibacter actinomycetemcomitans* | 9.34E-001 | 4 | 5.84E-005 | 6.69E-001 | 6.69E-001 | 8.60E-001 | 4.39E-001 | 2.32E-002 | 3.78E-001 | 1.00E+000 | 7.13E-001 | 8.60E-001 | 9.10E-002 | 1.00E+000 |
| 1500 | 6 | 4 | *Helicobacter pylori kx2 npgec Burkholderia pseudomallei Yersinia enterocolitica p60 bl6 streptococcus gordonii Pseudomonas aeruginosa Helicobacter pylori kx1 npgec* | 9.34E-001 | 0 | 1.00E+000 | 6.69E-001 | 6.69E-001 | 8.60E-001 | 4.39E-001 | 4.13E-001 | 1.00E+000 | 5.61E-001 | 3.61E-001 | 8.60E-001 | 4.74E-001 | 7.89E-001 |
| 1548 | 6 | 4 | *Helicobacter pylori kx2 npgec Listeria monocytogenes Helicobacter pylori kx2 mgep streptococcus pneumoniae d39 Helicobacter pylori kx1 npgec Ehrlichia chaffeensis liberty* | 9.34E-001 | 2 | 6.40E-002 | 9.08E-001 | 3.31E-001 | 5.03E-001 | 7.84E-001 | 1.36E-001 | 3.78E-001 | 1.00E+000 | 1.08E-001 | 8.60E-001 | 1.00E+000 | 7.89E-001 |
| 1540 | 6 | 4 | *Yersinia enterocolitica wap bl6 Burkholderia pseudomallei Yersinia enterocolitica p60 bl6 Ehrlichia chaffeensis wakulla Porphyromonas gingivalis Helicobacter pylori kx2 mgep* | 9.34E-001 | 0 | 1.00E+000 | 9.08E-001 | 3.31E-001 | 1.00E+000 | 1.13E-001 | 7.55E-001 | 1.00E+000 | 2.16E-001 | 3.61E-001 | 1.00E+000 | 4.74E-001 | 3.82E-001 |
| 1520 | 6 | 4 | *streptococcus gordonii Lactobacillus acidophilus Mycobacterium tuberculosis Fusobacterium nucleatum Aeromonas cavia Escherichia coli* | 9.34E-001 | 0 | 1.00E+000 | 3.31E-001 | 9.08E-001 | 5.03E-001 | 9.54E-001 | 7.55E-001 | 3.78E-001 | 5.61E-001 | 7.13E-001 | 8.60E-001 | 9.10E-002 | 1.00E+000 |
| 1570 | 6 | 4 | *Helicobacter pylori kx2 npgec Pseudomonas aeruginosa fdr875 Pseudomonas aeruginosa fdr440 Staphylococcus aureus Helicobacter pylori kx1 npgec Escherichia coli* | 9.34E-001 | 0 | 1.00E+000 | 3.31E-001 | 9.08E-001 | 8.60E-001 | 4.39E-001 | 2.32E-002 | 1.00E+000 | 8.87E-001 | 3.61E-001 | 1.73E-001 | 1.00E+000 | 1.00E+000 |
| 1502 | 6 | 4 | *Helicobacter pylori kx1 mgep Burkholderia pseudomallei Helicobacter pylori kx2 mgep Aeromonas cavia streptococcus pneumoniae d39 Helicobacter pylori kx1 npgec* | 9.34E-001 | 0 | 1.00E+000 | 9.08E-001 | 3.31E-001 | 8.60E-001 | 4.39E-001 | 1.36E-001 | 1.00E+000 | 8.87E-001 | 1.08E-001 | 8.60E-001 | 1.00E+000 | 7.89E-001 |
| 1544 | 6 | 4 | *Helicobacter pylori kx2 npgec Ehrlichia chaffeensis arkansa Bifidobacterium bifidum Mycobacterium tuberculosis streptococcus pneumoniae d39 Helicobacter pylori kx1 npgec* | 9.34E-001 | 0 | 1.00E+000 | 9.08E-001 | 3.31E-001 | 5.03E-001 | 9.54E-001 | 4.13E-001 | 3.78E-001 | 8.87E-001 | 7.13E-001 | 5.03E-001 | 1.00E+000 | 7.89E-001 |
| 1514 | 6 | 4 | *Helicobacter pylori kx2 npgec Helicobacter pylori kx1 mgep Staphylococcus aureus Pseudomonas aeruginosa Helicobacter pylori kx1 npgec Ehrlichia chaffeensis liberty* | 9.34E-001 | 0 | 1.00E+000 | 9.08E-001 | 3.31E-001 | 8.60E-001 | 4.39E-001 | 1.36E-001 | 1.00E+000 | 1.00E+000 | 3.61E-001 | 5.03E-001 | 1.00E+000 | 7.89E-001 |
| 698 | 4 | 19 | *Helicobacter pylori kx1 mgep Helicobacter pylori kx2 mgep streptococcus pneumoniae d39 Helicobacter pylori kx1 npgec* | 9.36E-001 | 13 | 1.75E-003 | 9.47E-001 | 3.03E-001 | 7.19E-001 | 6.80E-001 | 1.63E-002 | 1.00E+000 | 1.00E+000 | 1.22E-001 | 7.19E-001 | 1.00E+000 | 1.00E+000 |
| 702 | 4 | 19 | *Helicobacter pylori kx2 npgec streptococcus gordonii Mycobacterium tuberculosis streptococcus pneumoniae d39* | 9.36E-001 | 3 | 7.66E-001 | 3.03E-001 | 9.47E-001 | 2.77E-001 | 9.96E-001 | 1.46E-001 | 1.00E+000 | 7.55E-001 | 8.43E-001 | 2.77E-001 | 3.41E-001 | 1.00E+000 |
| 696 | 4 | 19 | *Yersinia enterocolitica wap bl6 Ehrlichia chaffeensis wakulla Aeromonas cavia Ehrlichia chaffeensis liberty* | 9.36E-001 | 31 | 1.12E-008 | 1.00E+000 | 5.35E-002 | 1.00E+000 | 2.45E-001 | 1.00E+000 | 1.00E+000 | 7.55E-001 | 4.51E-001 | 1.00E+000 | 1.00E+000 | 1.96E-001 |
| 720 | 4 | 18 | *Ehrlichia chaffeensis wakulla Bifidobacterium bifidum Pseudomonas aeruginosa Aeromonas cavia* | 9.42E-001 | 8 | 2.43E-004 | 9.47E-001 | 3.03E-001 | 7.19E-001 | 6.80E-001 | 1.00E+000 | 2.65E-001 | 1.00E+000 | 8.43E-001 | 7.19E-001 | 1.00E+000 | 6.34E-001 |
| 744 | 4 | 18 | *Burkholderia pseudomallei Pseudomonas aeruginosa fdr1 Staphylococcus aureus Helicobacter pylori kx2 mgep* | 9.42E-001 | 13 | 3.20E-003 | 3.03E-001 | 9.47E-001 | 7.19E-001 | 6.80E-001 | 1.46E-001 | 1.00E+000 | 7.55E-001 | 8.43E-001 | 2.77E-001 | 1.00E+000 | 6.34E-001 |
| 714 | 4 | 18 | *Helicobacter pylori kx2 npgec Ehrlichia chaffeensis arkansa Helicobacter pylori kx1 npgec Aggregatibacter actinomycetemcomitans* | 9.42E-001 | 31 | 6.90E-012 | 9.47E-001 | 3.03E-001 | 1.00E+000 | 2.45E-001 | 1.46E-001 | 1.00E+000 | 1.00E+000 | 4.51E-001 | 1.00E+000 | 3.41E-001 | 6.34E-001 |
| 762 | 4 | 17 | *Yersinia enterocolitica wap bl6 Yersinia enterocolitica p60 bc Ehrlichia chaffeensis liberty Aggregatibacter actinomycetemcomitans* | 9.47E-001 | 21 | 8.03E-005 | 9.47E-001 | 3.03E-001 | 1.00E+000 | 2.45E-001 | 8.66E-001 | 1.00E+000 | 3.20E-001 | 4.51E-001 | 1.00E+000 | 3.41E-001 | 6.34E-001 |
| 764 | 4 | 17 | *Yersinia enterocolitica p60 bc Pseudomonas aeruginosa Porphyromonas gingivalis streptococcus pneumoniae d39* | 9.47E-001 | 7 | 3.12E-001 | 3.03E-001 | 9.47E-001 | 7.19E-001 | 6.80E-001 | 4.95E-001 | 1.00E+000 | 7.55E-001 | 8.43E-001 | 2.77E-001 | 3.41E-001 | 1.00E+000 |
| 766 | 4 | 17 | *Yersinia enterocolitica wap bl6 Mycobacterium tuberculosis Helicobacter pylori kx2 mgep Helicobacter pylori kx1 npgec* | 9.47E-001 | 11 | 5.96E-004 | 9.47E-001 | 3.03E-001 | 1.00E+000 | 6.80E-001 | 4.95E-001 | 1.00E+000 | 3.20E-001 | 1.22E-001 | 7.19E-001 | 1.00E+000 | 1.00E+000 |
| 760 | 4 | 17 | *Burkholderia pseudomallei Pseudomonas aeruginosa fdr1 Pseudomonas aeruginosa Helicobacter pylori kx1 npgec* | 9.47E-001 | 0 | 1.00E+000 | 3.03E-001 | 9.47E-001 | 1.00E+000 | 2.45E-001 | 4.95E-001 | 1.00E+000 | 7.55E-001 | 8.43E-001 | 2.77E-001 | 1.00E+000 | 6.34E-001 |
| 1354 | 5 | 6 | *Burkholderia pseudomallei Pseudomonas aeruginosa fdr1 Staphylococcus aureus streptococcus pneumoniae d39 Helicobacter pylori kx1 npgec* | 9.51E-001 | 0 | 1.00E+000 | 1.72E-001 | 9.76E-001 | 3.93E-001 | 8.69E-001 | 6.08E-002 | 1.00E+000 | 8.32E-001 | 9.05E-001 | 1.03E-001 | 1.00E+000 | 7.21E-001 |
| 1396 | 5 | 6 | *Helicobacter pylori kx2 npgec Ehrlichia chaffeensis arkansa Ehrlichia chaffeensis wakulla Bifidobacterium bifidum Aggregatibacter actinomycetemcomitans* | 9.51E-001 | 0 | 1.00E+000 | 9.76E-001 | 1.72E-001 | 8.00E-001 | 5.54E-001 | 6.41E-001 | 3.23E-001 | 1.00E+000 | 9.05E-001 | 1.00E+000 | 4.10E-001 | 2.88E-001 |
| 1386 | 5 | 6 | *Burkholderia pseudomallei Ehrlichia chaffeensis wakulla Pseudomonas aeruginosa fdr1234 Lactobacillus acidophilus Bifidobacterium bifidum* | 9.51E-001 | 0 | 1.00E+000 | 8.28E-001 | 5.00E-001 | 3.93E-001 | 8.69E-001 | 9.23E-001 | 3.31E-002 | 8.32E-001 | 1.00E+000 | 8.00E-001 | 1.00E+000 | 2.88E-001 |
| 1372 | 5 | 6 | *Yersinia enterocolitica wap bl6 streptococcus gordonii Mycobacterium tuberculosis Fusobacterium nucleatum streptococcus pneumoniae d39* | 9.51E-001 | 0 | 1.00E+000 | 1.72E-001 | 9.76E-001 | 3.93E-001 | 9.82E-001 | 2.75E-001 | 1.00E+000 | 4.46E-001 | 9.05E-001 | 3.93E-001 | 6.29E-002 | 1.00E+000 |
| 1388 | 5 | 6 | *Helicobacter pylori kx2 npgec streptococcus pyogenes streptococcus pneumoniae d39 Helicobacter pylori kx1 npgec Aggregatibacter actinomycetemcomitans* | 9.51E-001 | 5 | 8.17E-002 | 8.28E-001 | 5.00E-001 | 3.93E-001 | 8.69E-001 | 6.08E-002 | 1.00E+000 | 1.00E+000 | 5.96E-001 | 8.00E-001 | 4.10E-001 | 1.00E+000 |
| 1338 | 5 | 6 | *Ehrlichia chaffeensis arkansa Pseudomonas aeruginosa fdr1 Helicobacter pylori kx1 npgec Ehrlichia chaffeensis liberty Aggregatibacter actinomycetemcomitans* | 9.51E-001 | 12 | 1.25E-008 | 8.28E-001 | 5.00E-001 | 1.00E+000 | 1.68E-001 | 2.75E-001 | 1.00E+000 | 1.00E+000 | 9.05E-001 | 8.00E-001 | 4.10E-001 | 2.88E-001 |
| 1362 | 5 | 6 | *Yersinia enterocolitica wap bl6 Helicobacter pylori kx2 npgec Ehrlichia chaffeensis arkansa Mycobacterium tuberculosis Helicobacter pylori kx1 npgec* | 9.51E-001 | 0 | 1.00E+000 | 9.76E-001 | 1.72E-001 | 1.00E+000 | 5.54E-001 | 6.41E-001 | 1.00E+000 | 4.46E-001 | 2.35E-001 | 8.00E-001 | 1.00E+000 | 7.21E-001 |
| 1370 | 5 | 6 | *Helicobacter pylori kx2 npgec Ehrlichia chaffeensis wakulla Pseudomonas aeruginosa fdr1 Helicobacter pylori kx1 npgec Ehrlichia chaffeensis liberty* | 9.51E-001 | 9 | 3.84E-003 | 9.76E-001 | 1.72E-001 | 1.00E+000 | 1.68E-001 | 2.75E-001 | 1.00E+000 | 1.00E+000 | 5.96E-001 | 8.00E-001 | 1.00E+000 | 2.88E-001 |
| 1378 | 5 | 6 | *Helicobacter pylori kx1 mgep Ehrlichia chaffeensis arkansa Ehrlichia chaffeensis wakulla Helicobacter pylori kx1 npgec Ehrlichia chaffeensis liberty* | 9.51E-001 | 0 | 1.00E+000 | 1.00E+000 | 2.39E-002 | 1.00E+000 | 1.68E-001 | 6.41E-001 | 1.00E+000 | 1.00E+000 | 5.96E-001 | 1.00E+000 | 1.00E+000 | 5.72E-002 |
| 1384 | 5 | 6 | *Pseudomonas aeruginosa fdr1 Pseudomonas aeruginosa fdr1234 Pseudomonas aeruginosa fdr440 Staphylococcus aureus Porphyromonas gingivalis* | 9.51E-001 | 7 | 3.89E-002 | 2.39E-002 | 1.00E+000 | 8.00E-001 | 5.54E-001 | 5.13E-003 | 1.00E+000 | 1.00E+000 | 1.00E+000 | 1.26E-002 | 4.10E-001 | 1.00E+000 |
| 1348 | 5 | 6 | *Helicobacter pylori kx2 npgec Ehrlichia chaffeensis wakulla Helicobacter pylori kx1 npgec Ehrlichia chaffeensis liberty Aggregatibacter actinomycetemcomitans* | 9.51E-001 | 0 | 1.00E+000 | 9.76E-001 | 1.72E-001 | 1.00E+000 | 1.68E-001 | 2.75E-001 | 1.00E+000 | 1.00E+000 | 5.96E-001 | 1.00E+000 | 4.10E-001 | 2.88E-001 |
| 790 | 4 | 16 | *Bifidobacterium bifidum Porphyromonas gingivalis Escherichia coli Ehrlichia chaffeensis liberty* | 9.52E-001 | 5 | 9.26E-002 | 6.97E-001 | 6.97E-001 | 7.19E-001 | 6.80E-001 | 8.66E-001 | 2.65E-001 | 7.55E-001 | 8.43E-001 | 1.00E+000 | 3.41E-001 | 6.34E-001 |
| 804 | 4 | 16 | *Helicobacter pylori kx2 npgec Listeria monocytogenes Helicobacter pylori kx2 mgep Helicobacter pylori kx1 npgec* | 9.52E-001 | 28 | 4.23E-010 | 9.47E-001 | 3.03E-001 | 7.19E-001 | 6.80E-001 | 1.46E-001 | 2.65E-001 | 1.00E+000 | 1.22E-002 | 1.00E+000 | 1.00E+000 | 1.00E+000 |
| 792 | 4 | 16 | *Ehrlichia chaffeensis arkansa Lactobacillus acidophilus Bifidobacterium bifidum Escherichia coli* | 9.52E-001 | 1 | 8.75E-002 | 9.47E-001 | 3.03E-001 | 2.77E-001 | 9.37E-001 | 1.00E+000 | 2.02E-002 | 7.55E-001 | 8.43E-001 | 1.00E+000 | 1.00E+000 | 6.34E-001 |
| 272 | 3 | 57 | *Pseudomonas aeruginosa fdr440 Aeromonas cavia Aggregatibacter actinomycetemcomitans* | 9.57E-001 | 21 | 5.04E-004 | 5.00E-001 | 8.84E-001 | 1.00E+000 | 3.54E-001 | 3.21E-001 | 1.00E+000 | 1.00E+000 | 7.45E-001 | 6.08E-001 | 2.65E-001 | 1.00E+000 |
| 862 | 4 | 15 | *Ehrlichia chaffeensis arkansa Ehrlichia chaffeensis wakulla Escherichia coli Ehrlichia chaffeensis liberty* | 9.57E-001 | 32 | 8.19E-010 | 9.47E-001 | 3.03E-001 | 1.00E+000 | 2.45E-001 | 1.00E+000 | 1.00E+000 | 7.55E-001 | 8.43E-001 | 1.00E+000 | 1.00E+000 | 2.59E-002 |
| 856 | 4 | 15 | *Helicobacter pylori kx2 npgec streptococcus gordonii Helicobacter pylori kx2 mgep Helicobacter pylori kx1 npgec* | 9.57E-001 | 8 | 1.61E-001 | 9.47E-001 | 3.03E-001 | 7.19E-001 | 6.80E-001 | 1.63E-002 | 1.00E+000 | 1.00E+000 | 1.22E-001 | 1.00E+000 | 3.41E-001 | 1.00E+000 |
| 850 | 4 | 15 | *Ehrlichia chaffeensis arkansa streptococcus pyogenes Helicobacter pylori kx1 npgec Ehrlichia chaffeensis liberty* | 9.57E-001 | 45 | < 1.67E-026 | 1.00E+000 | 5.35E-002 | 7.19E-001 | 6.80E-001 | 8.66E-001 | 1.00E+000 | 1.00E+000 | 8.43E-001 | 1.00E+000 | 1.00E+000 | 1.96E-001 |
| 872 | 4 | 15 | *Yersinia enterocolitica p60 bl6 Staphylococcus aureus Pseudomonas aeruginosa Helicobacter pylori kx2 mgep* | 9.57E-001 | 3 | 2.28E-001 | 6.97E-001 | 6.97E-001 | 7.19E-001 | 6.80E-001 | 4.95E-001 | 1.00E+000 | 7.55E-001 | 4.51E-001 | 2.77E-001 | 1.00E+000 | 1.00E+000 |
| 870 | 4 | 15 | *Helicobacter pylori kx2 npgec Helicobacter pylori kx1 mgep Ehrlichia chaffeensis wakulla Helicobacter pylori kx1 npgec* | 9.57E-001 | 12 | 2.88E-004 | 1.00E+000 | 5.35E-002 | 1.00E+000 | 2.45E-001 | 1.46E-001 | 1.00E+000 | 1.00E+000 | 1.22E-001 | 1.00E+000 | 1.00E+000 | 6.34E-001 |
| 948 | 9 | 6 | *Helicobacter pylori kx2 npgec Burkholderia pseudomallei Yersinia enterocolitica p60 bc Staphylococcus aureus Bifidobacterium bifidum Porphyromonas gingivalis Aeromonas cavia Helicobacter pylori kx1 npgec Ehrlichia chaffeensis arkansa* | 9.62E-002 | 0 | 1.00E+000 | 9.35E-001 | 2.27E-001 | 7.60E-001 | 4.91E-001 | 4.71E-001 | 5.25E-001 | 8.10E-001 | 4.04E-001 | 9.55E-001 | 6.34E-001 | 6.33E-001 |
| 936 | 9 | 6 | *Helicobacter pylori kx2 npgec Burkholderia pseudomallei Lactobacillus acidophilus Bifidobacterium bifidum Porphyromonas gingivalis Helicobacter pylori kx2 mgep Helicobacter pylori kx1 npgec Escherichia coli Aggregatibacter actinomycetemcomitans* | 9.62E-002 | 0 | 1.00E+000 | 7.73E-001 | 5.00E-001 | 7.60E-001 | 4.91E-001 | 2.02E-001 | 1.11E-001 | 8.10E-001 | 4.04E-001 | 1.00E+000 | 1.96E-001 | 9.14E-001 |
| 942 | 9 | 6 | *Helicobacter pylori kx2 npgec Burkholderia pseudomallei Pseudomonas aeruginosa streptococcus pneumoniae d39 Helicobacter pylori kx1 npgec Pseudomonas aeruginosa fdr440 Mycobacterium tuberculosis Helicobacter pylori kx2 mgep Escherichia coli* | 9.62E-002 | 0 | 1.00E+000 | 2.27E-001 | 9.35E-001 | 9.55E-001 | 4.91E-001 | 2.02E-001 | 1.00E+000 | 5.09E-001 | 4.04E-001 | 1.63E-001 | 1.00E+000 | 9.14E-001 |
| 914 | 4 | 14 | *Pseudomonas aeruginosa fdr1234 Lactobacillus acidophilus Helicobacter pylori kx2 mgep Helicobacter pylori kx1 npgec* | 9.63E-001 | 7 | 2.18E-004 | 9.47E-001 | 3.03E-001 | 7.19E-001 | 6.80E-001 | 1.46E-001 | 2.65E-001 | 1.00E+000 | 4.51E-001 | 7.19E-001 | 1.00E+000 | 1.00E+000 |
| 908 | 4 | 14 | *Lactobacillus acidophilus Pseudomonas aeruginosa fdr440 Aeromonas cavia streptococcus pneumoniae d39* | 9.63E-001 | 2 | 2.83E-001 | 6.97E-001 | 6.97E-001 | 2.77E-001 | 9.37E-001 | 4.95E-001 | 2.65E-001 | 1.00E+000 | 8.43E-001 | 2.77E-001 | 1.00E+000 | 1.00E+000 |
| 884 | 4 | 14 | *Yersinia enterocolitica p60 bl6 Pseudomonas aeruginosa fdr1 Porphyromonas gingivalis Escherichia coli* | 9.63E-001 | 3 | 3.27E-001 | 3.03E-001 | 9.47E-001 | 1.00E+000 | 2.45E-001 | 4.95E-001 | 1.00E+000 | 3.20E-001 | 4.51E-001 | 7.19E-001 | 3.41E-001 | 1.00E+000 |
| 308 | 3 | 51 | *Ehrlichia chaffeensis arkansa Helicobacter pylori kx2 mgep Helicobacter pylori kx1 npgec* | 9.66E-001 | 49 | 3.08E-013 | 1.00E+000 | 1.16E-001 | 1.00E+000 | 3.54E-001 | 3.21E-001 | 1.00E+000 | 1.00E+000 | 2.87E-001 | 1.00E+000 | 1.00E+000 | 5.25E-001 |
| 310 | 3 | 51 | *Burkholderia pseudomallei Yersinia enterocolitica p60 bl6 Mycobacterium tuberculosis* | 9.66E-001 | 2 | 9.59E-001 | 5.00E-001 | 8.84E-001 | 1.00E+000 | 8.08E-001 | 1.00E+000 | 1.00E+000 | 1.92E-002 | 7.45E-001 | 6.08E-001 | 1.00E+000 | 5.25E-001 |
| 1472 | 5 | 5 | *Helicobacter pylori kx2 npgec Burkholderia pseudomallei Bacillus anthracis Bifidobacterium bifidum Helicobacter pylori kx1 npgec* | 9.67E-001 | 0 | 1.00E+000 | 8.28E-001 | 5.00E-001 | 3.93E-001 | 8.69E-001 | 6.41E-001 | 3.23E-001 | 4.46E-001 | 5.96E-001 | 1.00E+000 | 1.00E+000 | 2.88E-001 |
| 1466 | 5 | 5 | *Helicobacter pylori kx2 npgec Helicobacter pylori kx1 mgep Pseudomonas aeruginosa fdr1 Porphyromonas gingivalis Helicobacter pylori kx1 npgec* | 9.67E-001 | 8 | 7.78E-003 | 8.28E-001 | 5.00E-001 | 1.00E+000 | 1.68E-001 | 5.13E-003 | 1.00E+000 | 1.00E+000 | 2.35E-001 | 8.00E-001 | 4.10E-001 | 1.00E+000 |
| 1470 | 5 | 5 | *Helicobacter pylori kx2 npgec Helicobacter pylori kx1 mgep streptococcus pneumoniae d39 Helicobacter pylori kx1 npgec Ehrlichia chaffeensis liberty* | 9.67E-001 | 4 | 2.45E-001 | 9.76E-001 | 1.72E-001 | 8.00E-001 | 5.54E-001 | 6.08E-002 | 1.00E+000 | 1.00E+000 | 2.35E-001 | 8.00E-001 | 1.00E+000 | 7.21E-001 |
| 1476 | 5 | 5 | *Helicobacter pylori kx1 mgep Pseudomonas aeruginosa fdr440 Helicobacter pylori kx2 mgep Helicobacter pylori kx1 npgec Ehrlichia chaffeensis liberty* | 9.67E-001 | 8 | 1.25E-003 | 9.76E-001 | 1.72E-001 | 1.00E+000 | 1.68E-001 | 6.08E-002 | 1.00E+000 | 1.00E+000 | 2.35E-001 | 8.00E-001 | 1.00E+000 | 7.21E-001 |
| 1484 | 5 | 5 | *Burkholderia pseudomallei Pseudomonas aeruginosa fdr1 Lactobacillus acidophilus Pseudomonas aeruginosa fdr875 Aggregatibacter actinomycetemcomitans* | 9.67E-001 | 0 | 1.00E+000 | 1.72E-001 | 9.76E-001 | 8.00E-001 | 5.54E-001 | 2.75E-001 | 3.23E-001 | 8.32E-001 | 1.00E+000 | 3.93E-001 | 4.10E-001 | 7.21E-001 |
| 1468 | 5 | 5 | *Yersinia enterocolitica p60 bl6 Pseudomonas aeruginosa fdr440 Staphylococcus aureus streptococcus pneumoniae d39 Helicobacter pylori kx1 npgec* | 9.67E-001 | 1 | 7.47E-001 | 5.00E-001 | 8.28E-001 | 3.93E-001 | 8.69E-001 | 6.08E-002 | 1.00E+000 | 8.32E-001 | 5.96E-001 | 1.03E-001 | 1.00E+000 | 1.00E+000 |
| 1478 | 5 | 5 | *Helicobacter pylori kx2 npgec Pseudomonas aeruginosa fdr440 streptococcus pyogenes Helicobacter pylori kx1 npgec Escherichia coli* | 9.67E-001 | 7 | 3.43E-004 | 8.28E-001 | 5.00E-001 | 8.00E-001 | 5.54E-001 | 2.75E-001 | 1.00E+000 | 8.32E-001 | 2.35E-001 | 8.00E-001 | 1.00E+000 | 1.00E+000 |
| 1474 | 5 | 5 | *Yersinia enterocolitica wap bl6 Yersinia enterocolitica wap bc Yersinia enterocolitica p60 bl6 Yersinia enterocolitica p60 bc Pseudomonas aeruginosa* | 9.67E-001 | 0 | 1.00E+000 | 9.76E-001 | 1.72E-001 | 1.00E+000 | 1.68E-001 | 1.00E+000 | 1.00E+000 | 1.84E-002 | 4.69E-002 | 8.00E-001 | 1.00E+000 | 1.00E+000 |
| 1482 | 5 | 5 | *Helicobacter pylori kx2 npgec Pseudomonas aeruginosa fdr875 Mycobacterium tuberculosis Helicobacter pylori kx2 mgep Helicobacter pylori kx1 npgec* | 9.67E-001 | 5 | 3.60E-003 | 8.28E-001 | 5.00E-001 | 1.00E+000 | 5.54E-001 | 6.08E-002 | 1.00E+000 | 8.32E-001 | 2.35E-001 | 3.93E-001 | 1.00E+000 | 1.00E+000 |
| 1480 | 5 | 5 | *Helicobacter pylori kx2 npgec Ehrlichia chaffeensis wakulla Lactobacillus acidophilus Listeria monocytogenes Helicobacter pylori kx1 npgec* | 9.67E-001 | 0 | 1.00E+000 | 9.76E-001 | 1.72E-001 | 3.93E-001 | 8.69E-001 | 6.41E-001 | 3.31E-002 | 1.00E+000 | 2.35E-001 | 1.00E+000 | 1.00E+000 | 7.21E-001 |
| 324 | 3 | 50 | *Mycobacterium tuberculosis Helicobacter pylori kx1 npgec Ehrlichia chaffeensis liberty* | 9.67E-001 | 32 | 3.84E-005 | 8.84E-001 | 5.00E-001 | 1.00E+000 | 8.08E-001 | 7.74E-001 | 1.00E+000 | 6.46E-001 | 7.45E-001 | 6.08E-001 | 1.00E+000 | 5.25E-001 |
| 328 | 3 | 50 | *Fusobacterium nucleatum streptococcus pneumoniae d39 Helicobacter pylori kx1 npgec* | 9.67E-001 | 25 | 2.97E-001 | 5.00E-001 | 8.84E-001 | 6.08E-001 | 8.08E-001 | 4.88E-002 | 1.00E+000 | 1.00E+000 | 7.45E-001 | 6.08E-001 | 2.65E-001 | 1.00E+000 |
| 950 | 4 | 13 | *Yersinia enterocolitica p60 bl6 Pseudomonas aeruginosa fdr1 Porphyromonas gingivalis Helicobacter pylori kx2 mgep* | 9.67E-001 | 19 | 4.63E-006 | 6.97E-001 | 6.97E-001 | 1.00E+000 | 2.45E-001 | 1.46E-001 | 1.00E+000 | 7.55E-001 | 4.51E-001 | 7.19E-001 | 3.41E-001 | 1.00E+000 |
| 954 | 4 | 13 | *Yersinia enterocolitica wap bl6 Pseudomonas aeruginosa fdr440 Staphylococcus aureus Bifidobacterium bifidum* | 9.67E-001 | 2 | 6.40E-002 | 6.97E-001 | 6.97E-001 | 2.77E-001 | 9.37E-001 | 4.95E-001 | 2.65E-001 | 7.55E-001 | 8.43E-001 | 2.77E-001 | 1.00E+000 | 1.00E+000 |
| 952 | 4 | 13 | *Yersinia enterocolitica wap bl6 Ehrlichia chaffeensis wakulla Porphyromonas gingivalis streptococcus pneumoniae d39* | 9.67E-001 | 23 | 6.66E-005 | 6.97E-001 | 6.97E-001 | 7.19E-001 | 6.80E-001 | 4.95E-001 | 1.00E+000 | 7.55E-001 | 8.43E-001 | 7.19E-001 | 3.41E-001 | 6.34E-001 |
| 1588 | 7 | 3 | *Helicobacter pylori kx2 npgec Yersinia enterocolitica p60 bl6 Pseudomonas aeruginosa fdr440 Staphylococcus aureus Porphyromonas gingivalis Helicobacter pylori kx1 npgec Ehrlichia chaffeensis liberty* | 9.68E-001 | 0 | 1.00E+000 | 7.95E-001 | 5.00E-001 | 9.03E-001 | 3.40E-001 | 6.08E-002 | 1.00E+000 | 9.25E-001 | 4.88E-001 | 6.02E-001 | 5.32E-001 | 8.42E-001 |
| 1608 | 7 | 3 | *Helicobacter pylori kx2 npgec Burkholderia pseudomallei Pseudomonas aeruginosa fdr875 Porphyromonas gingivalis Aeromonas cavia Helicobacter pylori kx1 npgec Aggregatibacter actinomycetemcomitans* | 9.68E-001 | 0 | 1.00E+000 | 5.00E-001 | 7.95E-001 | 1.00E+000 | 7.55E-002 | 6.08E-002 | 1.00E+000 | 9.25E-001 | 4.88E-001 | 9.03E-001 | 1.23E-001 | 8.42E-001 |
| 1604 | 7 | 3 | *Burkholderia pseudomallei Pseudomonas aeruginosa fdr1 Lactobacillus acidophilus Pseudomonas aeruginosa fdr875 Pseudomonas aeruginosa fdr440 Staphylococcus aureus Helicobacter pylori kx2 mgep* | 9.68E-001 | 0 | 1.00E+000 | 2.05E-001 | 9.53E-001 | 6.02E-001 | 6.88E-001 | 6.08E-002 | 4.30E-001 | 9.25E-001 | 9.67E-001 | 6.35E-002 | 1.00E+000 | 8.42E-001 |
| 1582 | 7 | 3 | *Helicobacter pylori kx2 npgec Helicobacter pylori kx1 mgep Burkholderia pseudomallei Yersinia enterocolitica p60 bc Porphyromonas gingivalis Helicobacter pylori kx2 mgep Helicobacter pylori kx1 npgec* | 9.68E-001 | 0 | 1.00E+000 | 9.53E-001 | 2.05E-001 | 1.00E+000 | 7.55E-002 | 6.08E-002 | 1.00E+000 | 6.60E-001 | 4.43E-002 | 1.00E+000 | 5.32E-001 | 8.42E-001 |
| 1594 | 7 | 3 | *Yersinia enterocolitica wap bl6 Yersinia enterocolitica p60 bl6 Yersinia enterocolitica p60 bc Ehrlichia chaffeensis wakulla Porphyromonas gingivalis Ehrlichia chaffeensis liberty Aggregatibacter actinomycetemcomitans* | 9.68E-001 | 4 | 5.13E-003 | 9.53E-001 | 2.05E-001 | 1.00E+000 | 7.55E-002 | 8.39E-001 | 1.00E+000 | 3.12E-001 | 4.88E-001 | 1.00E+000 | 1.23E-001 | 4.72E-001 |
| 1606 | 7 | 3 | *Yersinia enterocolitica wap bl6 Helicobacter pylori kx2 npgec Ehrlichia chaffeensis arkansa Yersinia enterocolitica p60 bc streptococcus pneumoniae d39 Helicobacter pylori kx1 npgec Ehrlichia chaffeensis liberty* | 9.68E-001 | 0 | 1.00E+000 | 9.96E-001 | 4.65E-002 | 9.03E-001 | 3.40E-001 | 5.46E-001 | 1.00E+000 | 6.60E-001 | 1.92E-001 | 9.03E-001 | 1.00E+000 | 4.72E-001 |
| 1600 | 7 | 3 | *Helicobacter pylori kx2 npgec Helicobacter pylori kx1 mgep Porphyromonas gingivalis Helicobacter pylori kx2 mgep streptococcus pneumoniae d39 Helicobacter pylori kx1 npgec Aggregatibacter actinomycetemcomitans* | 9.68E-001 | 3 | 1.38E-001 | 7.95E-001 | 5.00E-001 | 9.03E-001 | 3.40E-001 | 4.24E-004 | 1.00E+000 | 1.00E+000 | 1.92E-001 | 9.03E-001 | 1.23E-001 | 1.00E+000 |
| 1584 | 7 | 3 | *Yersinia enterocolitica wap bl6 Helicobacter pylori kx2 npgec Ehrlichia chaffeensis wakulla Helicobacter pylori kx2 mgep Helicobacter pylori kx1 npgec Escherichia coli Ehrlichia chaffeensis liberty* | 9.68E-001 | 3 | 9.86E-002 | 9.96E-001 | 4.65E-002 | 1.00E+000 | 7.55E-002 | 5.46E-001 | 1.00E+000 | 6.60E-001 | 4.43E-002 | 1.00E+000 | 1.00E+000 | 4.72E-001 |
| 1602 | 7 | 3 | *Helicobacter pylori kx2 npgec Ehrlichia chaffeensis arkansa Burkholderia pseudomallei streptococcus gordonii Ehrlichia chaffeensis wakulla streptococcus pneumoniae d39 Helicobacter pylori kx1 npgec* | 9.68E-001 | 0 | 1.00E+000 | 7.95E-001 | 5.00E-001 | 6.02E-001 | 6.88E-001 | 2.36E-001 | 1.00E+000 | 9.25E-001 | 8.03E-001 | 9.03E-001 | 5.32E-001 | 1.55E-001 |
| 1610 | 7 | 3 | *Helicobacter pylori kx2 npgec streptococcus gordonii Pseudomonas aeruginosa fdr440 Fusobacterium nucleatum Aeromonas cavia streptococcus pneumoniae d39 Helicobacter pylori kx1 npgec* | 9.68E-001 | 0 | 1.00E+000 | 5.00E-001 | 7.95E-001 | 6.02E-001 | 6.88E-001 | 8.14E-003 | 1.00E+000 | 1.00E+000 | 4.88E-001 | 6.02E-001 | 1.23E-001 | 1.00E+000 |
| 1596 | 7 | 3 | *Helicobacter pylori kx2 npgec Ehrlichia chaffeensis arkansa Pseudomonas aeruginosa Helicobacter pylori kx2 mgep streptococcus pneumoniae d39 Helicobacter pylori kx1 npgec Ehrlichia chaffeensis liberty* | 9.68E-001 | 0 | 1.00E+000 | 9.53E-001 | 2.05E-001 | 9.03E-001 | 3.40E-001 | 2.36E-001 | 1.00E+000 | 1.00E+000 | 4.88E-001 | 6.02E-001 | 1.00E+000 | 4.72E-001 |
| 340 | 3 | 49 | *Pseudomonas aeruginosa fdr1 Pseudomonas aeruginosa fdr440 Aggregatibacter actinomycetemcomitans* | 9.69E-001 | 13 | 4.54E-001 | 1.16E-001 | 1.00E+000 | 1.00E+000 | 3.54E-001 | 4.88E-002 | 1.00E+000 | 1.00E+000 | 1.00E+000 | 1.63E-001 | 2.65E-001 | 1.00E+000 |
| 350 | 3 | 48 | *Burkholderia pseudomallei Mycobacterium tuberculosis Helicobacter pylori kx2 mgep* | 9.69E-001 | 6 | 1.84E-002 | 5.00E-001 | 8.84E-001 | 1.00E+000 | 8.08E-001 | 7.74E-001 | 1.00E+000 | 1.92E-001 | 7.45E-001 | 6.08E-001 | 1.00E+000 | 5.25E-001 |
| 372 | 3 | 46 | *Helicobacter pylori kx2 npgec streptococcus pyogenes Helicobacter pylori kx1 npgec* | 9.70E-001 | 109 | < 1.67E-026 | 1.00E+000 | 1.16E-001 | 6.08E-001 | 8.08E-001 | 3.21E-001 | 1.00E+000 | 1.00E+000 | 2.87E-001 | 1.00E+000 | 1.00E+000 | 1.00E+000 |
| 1048 | 4 | 12 | *streptococcus gordonii Porphyromonas gingivalis Mycobacterium tuberculosis Aggregatibacter actinomycetemcomitans* | 9.71E-001 | 1 | 9.77E-001 | 5.35E-002 | 1.00E+000 | 7.19E-001 | 9.37E-001 | 1.46E-001 | 1.00E+000 | 7.55E-001 | 1.00E+000 | 7.19E-001 | 1.37E-003 | 1.00E+000 |
| 388 | 3 | 43 | *Burkholderia pseudomallei Pseudomonas aeruginosa fdr1 Mycobacterium tuberculosis* | 9.73E-001 | 11 | 1.98E-002 | 1.16E-001 | 1.00E+000 | 1.00E+000 | 8.08E-001 | 7.74E-001 | 1.00E+000 | 1.92E-001 | 1.00E+000 | 1.63E-001 | 1.00E+000 | 5.25E-001 |
| 408 | 3 | 42 | *Helicobacter pylori kx2 npgec Pseudomonas aeruginosa fdr1234 Helicobacter pylori kx1 npgec* | 9.74E-001 | 40 | 1.73E-008 | 8.84E-001 | 5.00E-001 | 1.00E+000 | 3.54E-001 | 4.88E-002 | 1.00E+000 | 1.00E+000 | 2.87E-001 | 6.08E-001 | 1.00E+000 | 1.00E+000 |
| 1074 | 4 | 11 | *Ehrlichia chaffeensis arkansa Pseudomonas aeruginosa fdr440 Pseudomonas aeruginosa Ehrlichia chaffeensis liberty* | 9.76E-001 | 10 | 5.36E-005 | 6.97E-001 | 6.97E-001 | 1.00E+000 | 2.45E-001 | 8.66E-001 | 1.00E+000 | 1.00E+000 | 1.00E+000 | 2.77E-001 | 1.00E+000 | 1.96E-001 |
| 422 | 3 | 40 | *Yersinia enterocolitica p60 bl6 Helicobacter pylori kx2 mgep Helicobacter pylori kx1 npgec* | 9.77E-001 | 65 | 1.82E-012 | 1.00E+000 | 1.16E-001 | 1.00E+000 | 3.54E-001 | 3.21E-001 | 1.00E+000 | 6.46E-001 | 3.96E-002 | 1.00E+000 | 1.00E+000 | 1.00E+000 |
| 446 | 3 | 39 | *Pseudomonas aeruginosa fdr1 Helicobacter pylori kx1 npgec Ehrlichia chaffeensis liberty* | 9.79E-001 | 58 | < 1.67E-026 | 8.84E-001 | 5.00E-001 | 1.00E+000 | 3.54E-001 | 3.21E-001 | 1.00E+000 | 1.00E+000 | 7.45E-001 | 6.08E-001 | 1.00E+000 | 5.25E-001 |
| 448 | 3 | 39 | *Yersinia enterocolitica wap bl6 Pseudomonas aeruginosa Helicobacter pylori kx2 mgep* | 9.79E-001 | 18 | 8.01E-004 | 8.84E-001 | 5.00E-001 | 1.00E+000 | 3.54E-001 | 7.74E-001 | 1.00E+000 | 6.46E-001 | 2.87E-001 | 6.08E-001 | 1.00E+000 | 1.00E+000 |
| 450 | 3 | 39 | *Helicobacter pylori kx2 npgec Ehrlichia chaffeensis wakulla Helicobacter pylori kx2 mgep* | 9.79E-001 | 70 | 1.47E-012 | 1.00E+000 | 1.16E-001 | 1.00E+000 | 3.54E-001 | 3.21E-001 | 1.00E+000 | 1.00E+000 | 2.87E-001 | 1.00E+000 | 1.00E+000 | 5.25E-001 |
| 1648 | 5 | 4 | *Helicobacter pylori kx2 npgec Ehrlichia chaffeensis arkansa Lactobacillus acidophilus Mycobacterium tuberculosis Helicobacter pylori kx2 mgep* | 9.80E-001 | 9 | 5.06E-008 | 9.76E-001 | 1.72E-001 | 8.00E-001 | 8.69E-001 | 6.41E-001 | 3.23E-001 | 8.32E-001 | 5.96E-001 | 8.00E-001 | 1.00E+000 | 7.21E-001 |
| 1638 | 5 | 4 | *Yersinia enterocolitica wap bl6 Ehrlichia chaffeensis arkansa Ehrlichia chaffeensis wakulla streptococcus pyogenes Aeromonas cavia* | 9.80E-001 | 8 | 1.29E-007 | 1.00E+000 | 2.39E-002 | 8.00E-001 | 5.54E-001 | 1.00E+000 | 1.00E+000 | 8.32E-001 | 5.96E-001 | 1.00E+000 | 1.00E+000 | 2.88E-001 |
| 1660 | 5 | 4 | *Helicobacter pylori kx2 npgec Pseudomonas aeruginosa fdr1 streptococcus pyogenes Helicobacter pylori kx2 mgep Helicobacter pylori kx1 npgec* | 9.80E-001 | 11 | 9.55E-005 | 9.76E-001 | 1.72E-001 | 8.00E-001 | 5.54E-001 | 6.08E-002 | 1.00E+000 | 1.00E+000 | 2.35E-001 | 8.00E-001 | 1.00E+000 | 1.00E+000 |
| 1628 | 5 | 4 | *Pseudomonas aeruginosa fdr1234 Listeria monocytogenes Bifidobacterium bifidum Porphyromonas gingivalis Mycobacterium tuberculosis* | 9.80E-001 | 0 | 1.00E+000 | 1.72E-001 | 9.76E-001 | 3.93E-001 | 9.82E-001 | 6.41E-001 | 3.31E-002 | 8.32E-001 | 9.05E-001 | 3.93E-001 | 4.10E-001 | 1.00E+000 |
| 1612 | 5 | 4 | *Ehrlichia chaffeensis arkansa Burkholderia pseudomallei streptococcus pneumoniae d39 Helicobacter pylori kx1 npgec Escherichia coli* | 9.80E-001 | 0 | 1.00E+000 | 5.00E-001 | 8.28E-001 | 8.00E-001 | 5.54E-001 | 6.41E-001 | 1.00E+000 | 4.46E-001 | 5.96E-001 | 8.00E-001 | 1.00E+000 | 2.88E-001 |
| 1650 | 5 | 4 | *Helicobacter pylori kx1 mgep Lactobacillus acidophilus Pseudomonas aeruginosa fdr440 Mycobacterium tuberculosis Helicobacter pylori kx2 mgep* | 9.80E-001 | 0 | 1.00E+000 | 8.28E-001 | 5.00E-001 | 8.00E-001 | 8.69E-001 | 2.75E-001 | 3.23E-001 | 8.32E-001 | 5.96E-001 | 3.93E-001 | 1.00E+000 | 1.00E+000 |
| 1642 | 5 | 4 | *Yersinia enterocolitica wap bc Lactobacillus acidophilus Bifidobacterium bifidum Pseudomonas aeruginosa Porphyromonas gingivalis* | 9.80E-001 | 1 | 1.67E-001 | 8.28E-001 | 5.00E-001 | 3.93E-001 | 8.69E-001 | 9.23E-001 | 3.31E-002 | 8.32E-001 | 9.05E-001 | 8.00E-001 | 4.10E-001 | 1.00E+000 |
| 1652 | 5 | 4 | *Helicobacter pylori kx2 npgec Ehrlichia chaffeensis wakulla Pseudomonas aeruginosa fdr440 Helicobacter pylori kx2 mgep Helicobacter pylori kx1 npgec* | 9.80E-001 | 0 | 1.00E+000 | 9.76E-001 | 1.72E-001 | 1.00E+000 | 1.68E-001 | 6.08E-002 | 1.00E+000 | 1.00E+000 | 2.35E-001 | 8.00E-001 | 1.00E+000 | 7.21E-001 |
| 1616 | 5 | 4 | *Burkholderia pseudomallei Lactobacillus acidophilus Staphylococcus aureus Listeria monocytogenes streptococcus pneumoniae d39* | 9.80E-001 | 0 | 1.00E+000 | 1.72E-001 | 9.76E-001 | 1.26E-002 | 9.99E-001 | 6.41E-001 | 3.31E-002 | 8.32E-001 | 9.05E-001 | 3.93E-001 | 1.00E+000 | 7.21E-001 |
| 460 | 3 | 38 | *Pseudomonas aeruginosa fdr1 Lactobacillus acidophilus Pseudomonas aeruginosa fdr440* | 9.80E-001 | 7 | 1.90E-001 | 5.00E-001 | 8.84E-001 | 6.08E-001 | 8.08E-001 | 3.21E-001 | 2.04E-001 | 1.00E+000 | 1.00E+000 | 1.63E-001 | 1.00E+000 | 1.00E+000 |
| 1162 | 4 | 10 | *Burkholderia pseudomallei Pseudomonas aeruginosa fdr1234 Porphyromonas gingivalis Helicobacter pylori kx2 mgep* | 9.80E-001 | 7 | 7.20E-003 | 3.03E-001 | 9.47E-001 | 1.00E+000 | 2.45E-001 | 1.46E-001 | 1.00E+000 | 7.55E-001 | 8.43E-001 | 7.19E-001 | 3.41E-001 | 6.34E-001 |
| 1160 | 4 | 10 | *Helicobacter pylori kx2 npgec Yersinia enterocolitica p60 bc streptococcus pyogenes Helicobacter pylori kx1 npgec* | 9.80E-001 | 46 | < 1.67E-026 | 1.00E+000 | 5.35E-002 | 7.19E-001 | 6.80E-001 | 4.95E-001 | 1.00E+000 | 7.55E-001 | 1.22E-001 | 1.00E+000 | 1.00E+000 | 1.00E+000 |
| 1146 | 4 | 10 | *Pseudomonas aeruginosa fdr1234 Lactobacillus acidophilus Bifidobacterium bifidum Pseudomonas aeruginosa* | 9.80E-001 | 8 | 1.21E-006 | 6.97E-001 | 6.97E-001 | 2.77E-001 | 9.37E-001 | 8.66E-001 | 2.02E-002 | 1.00E+000 | 1.00E+000 | 2.77E-001 | 1.00E+000 | 1.00E+000 |
| 1158 | 4 | 10 | *Yersinia enterocolitica p60 bc Pseudomonas aeruginosa fdr440 Mycobacterium tuberculosis Aggregatibacter actinomycetemcomitans* | 9.80E-001 | 1 | 9.36E-001 | 3.03E-001 | 9.47E-001 | 1.00E+000 | 6.80E-001 | 4.95E-001 | 1.00E+000 | 3.20E-001 | 8.43E-001 | 2.77E-001 | 3.41E-001 | 1.00E+000 |
| 476 | 3 | 37 | *Burkholderia pseudomallei streptococcus gordonii Mycobacterium tuberculosis* | 9.82E-001 | 1 | 9.77E-001 | 1.16E-001 | 1.00E+000 | 6.08E-001 | 9.81E-001 | 7.74E-001 | 1.00E+000 | 1.92E-001 | 1.00E+000 | 6.08E-001 | 2.65E-001 | 5.25E-001 |
| 1216 | 4 | 9 | *Pseudomonas aeruginosa fdr1234 Pseudomonas aeruginosa fdr440 Porphyromonas gingivalis Escherichia coli* | 9.84E-001 | 1 | 8.54E-001 | 5.35E-002 | 1.00E+000 | 1.00E+000 | 2.45E-001 | 1.46E-001 | 1.00E+000 | 7.55E-001 | 8.43E-001 | 2.77E-001 | 3.41E-001 | 1.00E+000 |
| 1180 | 4 | 9 | *Helicobacter pylori kx1 mgep Lactobacillus acidophilus Porphyromonas gingivalis Helicobacter pylori kx2 mgep* | 9.84E-001 | 13 | 1.04E-007 | 9.47E-001 | 3.03E-001 | 7.19E-001 | 6.80E-001 | 1.46E-001 | 2.65E-001 | 1.00E+000 | 4.51E-001 | 1.00E+000 | 3.41E-001 | 1.00E+000 |
| 1238 | 4 | 9 | *Helicobacter pylori kx2 npgec Pseudomonas aeruginosa fdr1234 Pseudomonas aeruginosa fdr440 Helicobacter pylori kx1 npgec* | 9.84E-001 | 2 | 6.10E-001 | 6.97E-001 | 6.97E-001 | 1.00E+000 | 2.45E-001 | 1.63E-002 | 1.00E+000 | 1.00E+000 | 4.51E-001 | 2.77E-001 | 1.00E+000 | 1.00E+000 |
| 1224 | 4 | 9 | *streptococcus gordonii Pseudomonas aeruginosa fdr440 Mycobacterium tuberculosis Escherichia coli* | 9.84E-001 | 0 | 1.00E+000 | 5.35E-002 | 1.00E+000 | 7.19E-001 | 9.37E-001 | 4.95E-001 | 1.00E+000 | 3.20E-001 | 8.43E-001 | 2.77E-001 | 3.41E-001 | 1.00E+000 |
| 1234 | 4 | 9 | *Helicobacter pylori kx1 mgep Burkholderia pseudomallei Pseudomonas aeruginosa fdr1 Aggregatibacter actinomycetemcomitans* | 9.84E-001 | 0 | 1.00E+000 | 3.03E-001 | 9.47E-001 | 1.00E+000 | 2.45E-001 | 1.46E-001 | 1.00E+000 | 7.55E-001 | 8.43E-001 | 7.19E-001 | 3.41E-001 | 6.34E-001 |
| 1188 | 4 | 9 | *Pseudomonas aeruginosa fdr1 Lactobacillus acidophilus Staphylococcus aureus Pseudomonas aeruginosa* | 9.84E-001 | 0 | 1.00E+000 | 3.03E-001 | 9.47E-001 | 2.77E-001 | 9.37E-001 | 4.95E-001 | 2.65E-001 | 1.00E+000 | 1.00E+000 | 4.86E-002 | 1.00E+000 | 1.00E+000 |
| 496 | 3 | 34 | *Yersinia enterocolitica p60 bc Lactobacillus acidophilus streptococcus pneumoniae d39* | 9.85E-001 | 36 | 2.01E-004 | 8.84E-001 | 5.00E-001 | 1.63E-001 | 9.81E-001 | 7.74E-001 | 2.04E-001 | 6.46E-001 | 7.45E-001 | 6.08E-001 | 1.00E+000 | 1.00E+000 |
| 520 | 3 | 33 | *Burkholderia pseudomallei Yersinia enterocolitica p60 bl6 streptococcus pneumoniae d39* | 9.86E-001 | 27 | 6.02E-002 | 5.00E-001 | 8.84E-001 | 6.08E-001 | 8.08E-001 | 7.74E-001 | 1.00E+000 | 1.92E-001 | 7.45E-001 | 6.08E-001 | 1.00E+000 | 5.25E-001 |
| 524 | 3 | 33 | *streptococcus gordonii Pseudomonas aeruginosa Fusobacterium nucleatum* | 9.86E-001 | 27 | 1.13E-001 | 1.16E-001 | 1.00E+000 | 6.08E-001 | 8.08E-001 | 3.21E-001 | 1.00E+000 | 1.00E+000 | 1.00E+000 | 6.08E-001 | 2.02E-002 | 1.00E+000 |
| 1312 | 4 | 8 | *Helicobacter pylori kx2 npgec Ehrlichia chaffeensis wakulla streptococcus pyogenes Pseudomonas aeruginosa* | 9.87E-001 | 11 | 1.17E-005 | 9.47E-001 | 3.03E-001 | 7.19E-001 | 6.80E-001 | 8.66E-001 | 1.00E+000 | 1.00E+000 | 8.43E-001 | 7.19E-001 | 1.00E+000 | 6.34E-001 |
| 1308 | 4 | 8 | *Helicobacter pylori kx1 mgep Pseudomonas aeruginosa streptococcus pneumoniae d39 Helicobacter pylori kx1 npgec* | 9.87E-001 | 8 | 7.38E-002 | 6.97E-001 | 6.97E-001 | 7.19E-001 | 6.80E-001 | 1.46E-001 | 1.00E+000 | 1.00E+000 | 4.51E-001 | 2.77E-001 | 1.00E+000 | 1.00E+000 |
| 1320 | 4 | 8 | *Burkholderia pseudomallei streptococcus gordonii Lactobacillus acidophilus Pseudomonas aeruginosa fdr440* | 9.87E-001 | 0 | 1.00E+000 | 3.03E-001 | 9.47E-001 | 2.77E-001 | 9.37E-001 | 4.95E-001 | 2.65E-001 | 7.55E-001 | 1.00E+000 | 7.19E-001 | 3.41E-001 | 6.34E-001 |
| 1294 | 4 | 8 | *Ehrlichia chaffeensis arkansa Porphyromonas gingivalis Helicobacter pylori kx1 npgec Escherichia coli* | 9.87E-001 | 0 | 1.00E+000 | 6.97E-001 | 6.97E-001 | 1.00E+000 | 2.45E-001 | 4.95E-001 | 1.00E+000 | 7.55E-001 | 4.51E-001 | 1.00E+000 | 3.41E-001 | 6.34E-001 |
| 1306 | 4 | 8 | *Pseudomonas aeruginosa fdr1234 Pseudomonas aeruginosa fdr440 Bifidobacterium bifidum Helicobacter pylori kx2 mgep* | 9.87E-001 | 0 | 1.00E+000 | 6.97E-001 | 6.97E-001 | 7.19E-001 | 6.80E-001 | 1.46E-001 | 2.65E-001 | 1.00E+000 | 8.43E-001 | 2.77E-001 | 1.00E+000 | 1.00E+000 |
| 1328 | 4 | 8 | *Helicobacter pylori kx1 mgep Mycobacterium tuberculosis Aeromonas cavia Helicobacter pylori kx1 npgec* | 9.87E-001 | 5 | 1.29E-002 | 9.47E-001 | 3.03E-001 | 1.00E+000 | 6.80E-001 | 4.95E-001 | 1.00E+000 | 7.55E-001 | 1.22E-001 | 7.19E-001 | 1.00E+000 | 1.00E+000 |
| 1304 | 4 | 8 | *Yersinia enterocolitica wap bl6 Yersinia enterocolitica p60 bl6 streptococcus gordonii Fusobacterium nucleatum* | 9.87E-001 | 15 | 3.97E-002 | 6.97E-001 | 6.97E-001 | 7.19E-001 | 6.80E-001 | 4.95E-001 | 1.00E+000 | 3.20E-001 | 4.51E-001 | 1.00E+000 | 3.91E-002 | 1.00E+000 |
| 1314 | 4 | 8 | *Pseudomonas aeruginosa fdr1234 Pseudomonas aeruginosa fdr440 Aeromonas cavia Ehrlichia chaffeensis liberty* | 9.87E-001 | 10 | 1.01E-005 | 6.97E-001 | 6.97E-001 | 1.00E+000 | 2.45E-001 | 4.95E-001 | 1.00E+000 | 1.00E+000 | 8.43E-001 | 2.77E-001 | 1.00E+000 | 6.34E-001 |
| 582 | 3 | 30 | *Ehrlichia chaffeensis arkansa streptococcus pneumoniae d39 Helicobacter pylori kx1 npgec* | 9.88E-001 | 30 | 1.44E-008 | 8.84E-001 | 5.00E-001 | 6.08E-001 | 8.08E-001 | 3.21E-001 | 1.00E+000 | 1.00E+000 | 7.45E-001 | 6.08E-001 | 1.00E+000 | 5.25E-001 |
| 1722 | 6 | 3 | *Helicobacter pylori kx2 npgec Helicobacter pylori kx1 mgep Burkholderia pseudomallei Yersinia enterocolitica p60 bl6 streptococcus pneumoniae d39 Helicobacter pylori kx1 npgec* | 9.88E-001 | 0 | 1.00E+000 | 9.08E-001 | 3.31E-001 | 8.60E-001 | 4.39E-001 | 1.36E-001 | 1.00E+000 | 5.61E-001 | 1.08E-001 | 8.60E-001 | 1.00E+000 | 7.89E-001 |
| 1692 | 6 | 3 | *Ehrlichia chaffeensis arkansa streptococcus gordonii Ehrlichia chaffeensis wakulla Staphylococcus aureus Mycobacterium tuberculosis Helicobacter pylori kx1 npgec* | 9.88E-001 | 0 | 1.00E+000 | 6.69E-001 | 6.69E-001 | 5.03E-001 | 9.54E-001 | 4.13E-001 | 1.00E+000 | 8.87E-001 | 9.44E-001 | 5.03E-001 | 4.74E-001 | 3.82E-001 |
| 1728 | 6 | 3 | *Helicobacter pylori kx2 npgec Burkholderia pseudomallei Lactobacillus acidophilus Pseudomonas aeruginosa fdr875 Bifidobacterium bifidum Aeromonas cavia* | 9.88E-001 | 0 | 1.00E+000 | 9.08E-001 | 3.31E-001 | 5.03E-001 | 7.84E-001 | 7.55E-001 | 4.88E-002 | 8.87E-001 | 7.13E-001 | 8.60E-001 | 1.00E+000 | 7.89E-001 |
| 1688 | 6 | 3 | *Helicobacter pylori kx1 mgep Pseudomonas aeruginosa fdr1 Pseudomonas aeruginosa fdr440 Porphyromonas gingivalis Mycobacterium tuberculosis Aggregatibacter actinomycetemcomitans* | 9.88E-001 | 0 | 1.00E+000 | 9.18E-002 | 9.90E-001 | 1.00E+000 | 4.39E-001 | 2.32E-002 | 1.00E+000 | 8.87E-001 | 9.44E-001 | 1.73E-001 | 9.10E-002 | 1.00E+000 |
| 1700 | 6 | 3 | *Yersinia enterocolitica wap bl6 Helicobacter pylori kx1 mgep Burkholderia pseudomallei Porphyromonas gingivalis Helicobacter pylori kx2 mgep Aggregatibacter actinomycetemcomitans* | 9.88E-001 | 0 | 1.00E+000 | 6.69E-001 | 6.69E-001 | 1.00E+000 | 1.13E-001 | 1.36E-001 | 1.00E+000 | 5.61E-001 | 3.61E-001 | 1.00E+000 | 9.10E-002 | 7.89E-001 |
| 1676 | 6 | 3 | *Burkholderia pseudomallei Ehrlichia chaffeensis wakulla Lactobacillus acidophilus Staphylococcus aureus Ehrlichia chaffeensis liberty Aggregatibacter actinomycetemcomitans* | 9.88E-001 | 0 | 1.00E+000 | 6.69E-001 | 6.69E-001 | 5.03E-001 | 7.84E-001 | 7.55E-001 | 3.78E-001 | 8.87E-001 | 1.00E+000 | 8.60E-001 | 4.74E-001 | 1.01E-001 |
| 1684 | 6 | 3 | *Helicobacter pylori kx2 npgec streptococcus gordonii Pseudomonas aeruginosa Helicobacter pylori kx2 mgep streptococcus pneumoniae d39 Helicobacter pylori kx1 npgec* | 9.88E-001 | 0 | 1.00E+000 | 6.69E-001 | 6.69E-001 | 5.03E-001 | 7.84E-001 | 2.32E-002 | 1.00E+000 | 1.00E+000 | 3.61E-001 | 5.03E-001 | 4.74E-001 | 1.00E+000 |
| 1682 | 6 | 3 | *Helicobacter pylori kx2 npgec streptococcus pyogenes Pseudomonas aeruginosa Aeromonas cavia streptococcus pneumoniae d39 Helicobacter pylori kx1 npgec* | 9.88E-001 | 0 | 1.00E+000 | 9.08E-001 | 3.31E-001 | 5.03E-001 | 7.84E-001 | 4.13E-001 | 1.00E+000 | 1.00E+000 | 3.61E-001 | 5.03E-001 | 1.00E+000 | 1.00E+000 |
| 1716 | 6 | 3 | *Helicobacter pylori kx2 npgec Yersinia enterocolitica p60 bl6 Helicobacter pylori kx2 mgep streptococcus pneumoniae d39 Helicobacter pylori kx1 npgec Escherichia coli* | 9.88E-001 | 4 | 2.86E-002 | 9.08E-001 | 3.31E-001 | 8.60E-001 | 4.39E-001 | 1.36E-001 | 1.00E+000 | 5.61E-001 | 1.64E-002 | 8.60E-001 | 1.00E+000 | 1.00E+000 |
| 1724 | 6 | 3 | *Helicobacter pylori kx2 npgec Helicobacter pylori kx1 mgep Burkholderia pseudomallei Ehrlichia chaffeensis wakulla Pseudomonas aeruginosa Helicobacter pylori kx1 npgec* | 9.88E-001 | 0 | 1.00E+000 | 9.08E-001 | 3.31E-001 | 1.00E+000 | 1.13E-001 | 4.13E-001 | 1.00E+000 | 8.87E-001 | 3.61E-001 | 8.60E-001 | 1.00E+000 | 3.82E-001 |
| 1706 | 6 | 3 | *Helicobacter pylori kx2 npgec Eubacterium rectale Lactobacillus acidophilus Bifidobacterium bifidum Helicobacter pylori kx2 mgep Helicobacter pylori kx1 npgec* | 9.88E-001 | 0 | 1.00E+000 | 1.00E+000 | 1.03E-002 | 1.73E-001 | 9.54E-001 | 4.13E-001 | 4.88E-002 | 1.00E+000 | 1.08E-001 | 1.00E+000 | 1.00E+000 | 1.00E+000 |
| 1694 | 6 | 3 | *Helicobacter pylori kx2 npgec Staphylococcus aureus streptococcus pyogenes Pseudomonas aeruginosa Helicobacter pylori kx2 mgep Helicobacter pylori kx1 npgec* | 9.88E-001 | 7 | 4.14E-005 | 9.08E-001 | 3.31E-001 | 5.03E-001 | 7.84E-001 | 1.36E-001 | 1.00E+000 | 1.00E+000 | 3.61E-001 | 5.03E-001 | 1.00E+000 | 1.00E+000 |
| 1680 | 6 | 3 | *Helicobacter pylori kx2 npgec Ehrlichia chaffeensis arkansa Burkholderia pseudomallei Pseudomonas aeruginosa Mycobacterium tuberculosis Helicobacter pylori kx1 npgec* | 9.88E-001 | 0 | 1.00E+000 | 6.69E-001 | 6.69E-001 | 1.00E+000 | 4.39E-001 | 7.55E-001 | 1.00E+000 | 5.61E-001 | 7.13E-001 | 5.03E-001 | 1.00E+000 | 3.82E-001 |
| 1704 | 6 | 3 | *Helicobacter pylori kx1 mgep Pseudomonas aeruginosa fdr1234 Lactobacillus acidophilus Bifidobacterium bifidum Helicobacter pylori kx2 mgep Helicobacter pylori kx1 npgec* | 9.88E-001 | 0 | 1.00E+000 | 9.90E-001 | 9.18E-002 | 5.03E-001 | 7.84E-001 | 1.36E-001 | 4.88E-002 | 1.00E+000 | 3.61E-001 | 8.60E-001 | 1.00E+000 | 1.00E+000 |
| 1726 | 6 | 3 | *Burkholderia pseudomallei Lactobacillus acidophilus Pseudomonas aeruginosa fdr875 Pseudomonas aeruginosa fdr440 Porphyromonas gingivalis Aeromonas cavia* | 9.88E-001 | 0 | 1.00E+000 | 3.31E-001 | 9.08E-001 | 8.60E-001 | 4.39E-001 | 4.13E-001 | 3.78E-001 | 8.87E-001 | 9.44E-001 | 5.03E-001 | 4.74E-001 | 7.89E-001 |
| 1710 | 6 | 3 | *Helicobacter pylori kx2 npgec Lactobacillus acidophilus Pseudomonas aeruginosa fdr875 Bifidobacterium bifidum Pseudomonas aeruginosa streptococcus pneumoniae d39* | 9.88E-001 | 0 | 1.00E+000 | 6.69E-001 | 6.69E-001 | 1.73E-001 | 9.54E-001 | 4.13E-001 | 4.88E-002 | 1.00E+000 | 9.44E-001 | 1.73E-001 | 1.00E+000 | 1.00E+000 |
| 1702 | 6 | 3 | *Helicobacter pylori kx2 npgec Ehrlichia chaffeensis arkansa Pseudomonas aeruginosa fdr440 Bifidobacterium bifidum streptococcus pyogenes Ehrlichia chaffeensis liberty* | 9.88E-001 | 0 | 1.00E+000 | 9.90E-001 | 9.18E-002 | 5.03E-001 | 7.84E-001 | 7.55E-001 | 3.78E-001 | 1.00E+000 | 9.44E-001 | 8.60E-001 | 1.00E+000 | 3.82E-001 |
| 644 | 3 | 27 | *Helicobacter pylori kx2 npgec Ehrlichia chaffeensis arkansa Yersinia enterocolitica p60 bc* | 9.90E-001 | 38 | 4.48E-013 | 1.00E+000 | 1.16E-001 | 1.00E+000 | 3.54E-001 | 7.74E-001 | 1.00E+000 | 6.46E-001 | 2.87E-001 | 1.00E+000 | 1.00E+000 | 5.25E-001 |
| 652 | 3 | 27 | *Helicobacter pylori kx2 npgec Pseudomonas aeruginosa fdr875 Helicobacter pylori kx1 npgec* | 9.90E-001 | 18 | 9.23E-007 | 8.84E-001 | 5.00E-001 | 1.00E+000 | 3.54E-001 | 4.88E-002 | 1.00E+000 | 1.00E+000 | 2.87E-001 | 6.08E-001 | 1.00E+000 | 1.00E+000 |
| 646 | 3 | 27 | *Yersinia enterocolitica wap bl6 Pseudomonas aeruginosa fdr1 Aggregatibacter actinomycetemcomitans* | 9.90E-001 | 25 | 1.00E-005 | 5.00E-001 | 8.84E-001 | 1.00E+000 | 3.54E-001 | 3.21E-001 | 1.00E+000 | 6.46E-001 | 7.45E-001 | 6.08E-001 | 2.65E-001 | 1.00E+000 |
| 642 | 3 | 27 | *Listeria monocytogenes Aeromonas cavia Helicobacter pylori kx1 npgec* | 9.90E-001 | 14 | 3.77E-007 | 8.84E-001 | 5.00E-001 | 6.08E-001 | 8.08E-001 | 7.74E-001 | 2.04E-001 | 1.00E+000 | 3.96E-002 | 1.00E+000 | 1.00E+000 | 1.00E+000 |
| 684 | 3 | 26 | *Burkholderia pseudomallei streptococcus gordonii Aggregatibacter actinomycetemcomitans* | 9.90E-001 | 16 | 1.25E-001 | 1.16E-001 | 1.00E+000 | 6.08E-001 | 8.08E-001 | 3.21E-001 | 1.00E+000 | 6.46E-001 | 1.00E+000 | 1.00E+000 | 2.02E-002 | 5.25E-001 |
| 678 | 3 | 26 | *Yersinia enterocolitica p60 bl6 Porphyromonas gingivalis Ehrlichia chaffeensis liberty* | 9.90E-001 | 48 | 5.02E-011 | 8.84E-001 | 5.00E-001 | 1.00E+000 | 3.54E-001 | 7.74E-001 | 1.00E+000 | 6.46E-001 | 7.45E-001 | 1.00E+000 | 2.65E-001 | 5.25E-001 |
| 1404 | 4 | 7 | *Burkholderia pseudomallei Anaplasma phagocytophilum Porphyromonas gingivalis Ehrlichia chaffeensis liberty* | 9.90E-001 | 10 | 1.13E-006 | 3.03E-001 | 9.47E-001 | 1.00E+000 | 2.45E-001 | 8.66E-001 | 1.00E+000 | 7.55E-001 | 1.00E+000 | 1.00E+000 | 3.41E-001 | 2.59E-002 |
| 1402 | 4 | 7 | *Helicobacter pylori kx1 mgep Burkholderia pseudomallei Yersinia enterocolitica p60 bl6 Yersinia enterocolitica p60 bc* | 9.90E-001 | 2 | 4.14E-001 | 9.47E-001 | 3.03E-001 | 1.00E+000 | 2.45E-001 | 8.66E-001 | 1.00E+000 | 6.34E-002 | 1.22E-001 | 1.00E+000 | 1.00E+000 | 6.34E-001 |
| 1416 | 4 | 7 | *Helicobacter pylori kx1 mgep Staphylococcus aureus Helicobacter pylori kx2 mgep Aggregatibacter actinomycetemcomitans* | 9.90E-001 | 12 | 1.22E-003 | 6.97E-001 | 6.97E-001 | 7.19E-001 | 6.80E-001 | 1.63E-002 | 1.00E+000 | 1.00E+000 | 4.51E-001 | 7.19E-001 | 3.41E-001 | 1.00E+000 |
| 1428 | 4 | 7 | *Ehrlichia chaffeensis arkansa Ehrlichia chaffeensis wakulla Lactobacillus acidophilus Mycobacterium tuberculosis* | 9.90E-001 | 2 | 7.65E-003 | 9.47E-001 | 3.03E-001 | 7.19E-001 | 9.37E-001 | 1.00E+000 | 2.65E-001 | 7.55E-001 | 1.00E+000 | 7.19E-001 | 1.00E+000 | 1.96E-001 |
| 1406 | 4 | 7 | *Yersinia enterocolitica p60 bc Pseudomonas aeruginosa fdr440 Porphyromonas gingivalis Helicobacter pylori kx2 mgep* | 9.90E-001 | 5 | 2.70E-001 | 6.97E-001 | 6.97E-001 | 1.00E+000 | 2.45E-001 | 1.46E-001 | 1.00E+000 | 7.55E-001 | 4.51E-001 | 7.19E-001 | 3.41E-001 | 1.00E+000 |
| 704 | 3 | 25 | *Ehrlichia chaffeensis arkansa Porphyromonas gingivalis Aeromonas cavia* | 9.91E-001 | 30 | < 1.67E-026 | 8.84E-001 | 5.00E-001 | 1.00E+000 | 3.54E-001 | 7.74E-001 | 1.00E+000 | 1.00E+000 | 7.45E-001 | 1.00E+000 | 2.65E-001 | 5.25E-001 |
| 718 | 3 | 24 | *Helicobacter pylori kx1 mgep Burkholderia pseudomallei Porphyromonas gingivalis* | 9.92E-001 | 0 | 1.00E+000 | 5.00E-001 | 8.84E-001 | 1.00E+000 | 3.54E-001 | 3.21E-001 | 1.00E+000 | 6.46E-001 | 7.45E-001 | 1.00E+000 | 2.65E-001 | 5.25E-001 |
| 780 | 3 | 22 | *Pseudomonas aeruginosa fdr1234 Aeromonas cavia Aggregatibacter actinomycetemcomitans* | 9.93E-001 | 16 | 4.07E-006 | 5.00E-001 | 8.84E-001 | 1.00E+000 | 3.54E-001 | 3.21E-001 | 1.00E+000 | 1.00E+000 | 7.45E-001 | 6.08E-001 | 2.65E-001 | 1.00E+000 |
| 768 | 3 | 22 | *Yersinia enterocolitica p60 bl6 Pseudomonas aeruginosa fdr1 Staphylococcus aureus* | 9.93E-001 | 36 | 5.74E-013 | 5.00E-001 | 8.84E-001 | 6.08E-001 | 8.08E-001 | 3.21E-001 | 1.00E+000 | 6.46E-001 | 7.45E-001 | 1.63E-001 | 1.00E+000 | 1.00E+000 |
| 1488 | 4 | 6 | *Helicobacter pylori kx2 npgec Anaplasma phagocytophilum Helicobacter pylori kx1 npgec Escherichia coli* | 9.93E-001 | 1 | 8.89E-001 | 6.97E-001 | 6.97E-001 | 1.00E+000 | 2.45E-001 | 4.95E-001 | 1.00E+000 | 7.55E-001 | 1.22E-001 | 1.00E+000 | 1.00E+000 | 6.34E-001 |
| 1528 | 4 | 6 | *Helicobacter pylori kx2 npgec Ehrlichia chaffeensis arkansa Fusobacterium nucleatum Helicobacter pylori kx1 npgec* | 9.93E-001 | 3 | 6.01E-001 | 9.47E-001 | 3.03E-001 | 1.00E+000 | 2.45E-001 | 1.46E-001 | 1.00E+000 | 1.00E+000 | 4.51E-001 | 1.00E+000 | 3.41E-001 | 6.34E-001 |
| 1490 | 4 | 6 | *Helicobacter pylori kx1 mgep Porphyromonas gingivalis Helicobacter pylori kx2 mgep Ehrlichia chaffeensis liberty* | 9.93E-001 | 19 | 3.24E-008 | 9.47E-001 | 3.03E-001 | 1.00E+000 | 2.45E-001 | 1.46E-001 | 1.00E+000 | 1.00E+000 | 4.51E-001 | 1.00E+000 | 3.41E-001 | 6.34E-001 |
| 1550 | 4 | 6 | *Yersinia enterocolitica p60 bl6 Lactobacillus acidophilus Pseudomonas aeruginosa fdr440 Helicobacter pylori kx2 mgep* | 9.93E-001 | 0 | 1.00E+000 | 9.47E-001 | 3.03E-001 | 7.19E-001 | 6.80E-001 | 4.95E-001 | 2.65E-001 | 7.55E-001 | 4.51E-001 | 7.19E-001 | 1.00E+000 | 1.00E+000 |
| 1558 | 4 | 6 | *Helicobacter pylori kx2 npgec Pseudomonas aeruginosa fdr440 streptococcus pyogenes Pseudomonas aeruginosa* | 9.93E-001 | 4 | 5.84E-005 | 6.97E-001 | 6.97E-001 | 7.19E-001 | 6.80E-001 | 4.95E-001 | 1.00E+000 | 1.00E+000 | 8.43E-001 | 2.77E-001 | 1.00E+000 | 1.00E+000 |
| 1494 | 4 | 6 | *Burkholderia pseudomallei Ehrlichia chaffeensis wakulla Helicobacter pylori kx2 mgep Aggregatibacter actinomycetemcomitans* | 9.93E-001 | 0 | 1.00E+000 | 6.97E-001 | 6.97E-001 | 1.00E+000 | 2.45E-001 | 4.95E-001 | 1.00E+000 | 7.55E-001 | 8.43E-001 | 1.00E+000 | 3.41E-001 | 1.96E-001 |
| 1492 | 4 | 6 | *Ehrlichia chaffeensis wakulla Staphylococcus aureus Porphyromonas gingivalis Escherichia coli* | 9.93E-001 | 3 | 2.77E-001 | 3.03E-001 | 9.47E-001 | 7.19E-001 | 6.80E-001 | 4.95E-001 | 1.00E+000 | 7.55E-001 | 8.43E-001 | 7.19E-001 | 3.41E-001 | 6.34E-001 |
| 824 | 3 | 21 | *Burkholderia pseudomallei Pseudomonas aeruginosa fdr1234 Staphylococcus aureus* | 9.93E-001 | 0 | 1.00E+000 | 1.16E-001 | 1.00E+000 | 6.08E-001 | 8.08E-001 | 3.21E-001 | 1.00E+000 | 6.46E-001 | 1.00E+000 | 1.63E-001 | 1.00E+000 | 5.25E-001 |
| 822 | 3 | 21 | *Yersinia enterocolitica wap bl6 Pseudomonas aeruginosa fdr440 Ehrlichia chaffeensis liberty* | 9.93E-001 | 32 | 1.35E-009 | 8.84E-001 | 5.00E-001 | 1.00E+000 | 3.54E-001 | 7.74E-001 | 1.00E+000 | 6.46E-001 | 7.45E-001 | 6.08E-001 | 1.00E+000 | 5.25E-001 |
| 882 | 3 | 19 | *streptococcus pyogenes Helicobacter pylori kx2 mgep Helicobacter pylori kx1 npgec* | 9.94E-001 | 57 | < 1.67E-026 | 1.00E+000 | 1.16E-001 | 6.08E-001 | 8.08E-001 | 3.21E-001 | 1.00E+000 | 1.00E+000 | 2.87E-001 | 1.00E+000 | 1.00E+000 | 1.00E+000 |
| 880 | 3 | 19 | *Pseudomonas aeruginosa fdr875 Pseudomonas aeruginosa fdr440 Helicobacter pylori kx2 mgep* | 9.94E-001 | 2 | 9.68E-001 | 5.00E-001 | 8.84E-001 | 1.00E+000 | 3.54E-001 | 4.88E-002 | 1.00E+000 | 1.00E+000 | 7.45E-001 | 1.63E-001 | 1.00E+000 | 1.00E+000 |
| 878 | 3 | 19 | *Ehrlichia chaffeensis arkansa Pseudomonas aeruginosa fdr440 Helicobacter pylori kx1 npgec* | 9.94E-001 | 10 | 1.13E-006 | 8.84E-001 | 5.00E-001 | 1.00E+000 | 3.54E-001 | 3.21E-001 | 1.00E+000 | 1.00E+000 | 7.45E-001 | 6.08E-001 | 1.00E+000 | 5.25E-001 |
| 844 | 3 | 20 | *Helicobacter pylori kx2 npgec Listeria monocytogenes Ehrlichia chaffeensis liberty* | 9.94E-001 | 24 | 3.61E-011 | 8.84E-001 | 5.00E-001 | 6.08E-001 | 8.08E-001 | 7.74E-001 | 2.04E-001 | 1.00E+000 | 2.87E-001 | 1.00E+000 | 1.00E+000 | 5.25E-001 |
| 854 | 3 | 20 | *streptococcus gordonii Lactobacillus acidophilus Staphylococcus aureus* | 9.94E-001 | 18 | 1.88E-001 | 5.00E-001 | 8.84E-001 | 1.44E-002 | 1.00E+000 | 3.21E-001 | 2.04E-001 | 1.00E+000 | 1.00E+000 | 6.08E-001 | 2.65E-001 | 1.00E+000 |
| 470 | 7 | 16 | *Helicobacter pylori kx2 npgec Burkholderia pseudomallei Bifidobacterium bifidum Pseudomonas aeruginosa Helicobacter pylori kx2 mgep streptococcus pneumoniae d39 Helicobacter pylori kx1 npgec* | 9.94E-002 | 3 | 8.09E-002 | 7.95E-001 | 5.00E-001 | 6.02E-001 | 6.88E-001 | 2.36E-001 | 4.30E-001 | 9.25E-001 | 4.88E-001 | 6.02E-001 | 1.00E+000 | 8.42E-001 |
| 1630 | 4 | 5 | *Yersinia enterocolitica wap bl6 Pseudomonas aeruginosa fdr1234 Staphylococcus aureus Pseudomonas aeruginosa* | 9.95E-001 | 3 | 1.79E-002 | 3.03E-001 | 9.47E-001 | 7.19E-001 | 6.80E-001 | 4.95E-001 | 1.00E+000 | 7.55E-001 | 8.43E-001 | 4.86E-002 | 1.00E+000 | 1.00E+000 |
| 1618 | 4 | 5 | *Helicobacter pylori kx1 mgep Ehrlichia chaffeensis arkansa Pseudomonas aeruginosa Helicobacter pylori kx1 npgec* | 9.95E-001 | 11 | 1.28E-004 | 9.47E-001 | 3.03E-001 | 1.00E+000 | 2.45E-001 | 4.95E-001 | 1.00E+000 | 1.00E+000 | 4.51E-001 | 7.19E-001 | 1.00E+000 | 6.34E-001 |
| 1646 | 4 | 5 | *Helicobacter pylori kx2 npgec Pseudomonas aeruginosa fdr1234 Helicobacter pylori kx1 npgec Escherichia coli* | 9.95E-001 | 2 | 6.10E-001 | 6.97E-001 | 6.97E-001 | 1.00E+000 | 2.45E-001 | 1.46E-001 | 1.00E+000 | 7.55E-001 | 1.22E-001 | 7.19E-001 | 1.00E+000 | 1.00E+000 |
| 1662 | 4 | 5 | *Ehrlichia chaffeensis wakulla Listeria monocytogenes Bifidobacterium bifidum Aeromonas cavia* | 9.95E-001 | 4 | 3.06E-003 | 9.47E-001 | 3.03E-001 | 2.77E-001 | 9.37E-001 | 1.00E+000 | 2.02E-002 | 1.00E+000 | 4.51E-001 | 1.00E+000 | 1.00E+000 | 6.34E-001 |
| 1626 | 4 | 5 | *Ehrlichia chaffeensis arkansa Burkholderia pseudomallei streptococcus pyogenes Porphyromonas gingivalis* | 9.95E-001 | 0 | 1.00E+000 | 6.97E-001 | 6.97E-001 | 7.19E-001 | 6.80E-001 | 8.66E-001 | 1.00E+000 | 7.55E-001 | 1.00E+000 | 1.00E+000 | 3.41E-001 | 1.96E-001 |
| 1634 | 4 | 5 | *streptococcus pyogenes Mycobacterium tuberculosis Aeromonas cavia Helicobacter pylori kx1 npgec* | 9.95E-001 | 4 | 3.64E-002 | 9.47E-001 | 3.03E-001 | 7.19E-001 | 9.37E-001 | 8.66E-001 | 1.00E+000 | 7.55E-001 | 4.51E-001 | 7.19E-001 | 1.00E+000 | 1.00E+000 |
| 1636 | 4 | 5 | *Helicobacter pylori kx2 npgec Ehrlichia chaffeensis arkansa Mycobacterium tuberculosis Aeromonas cavia* | 9.95E-001 | 0 | 1.00E+000 | 9.47E-001 | 3.03E-001 | 1.00E+000 | 6.80E-001 | 8.66E-001 | 1.00E+000 | 7.55E-001 | 4.51E-001 | 7.19E-001 | 1.00E+000 | 6.34E-001 |
| 1644 | 4 | 5 | *Ehrlichia chaffeensis arkansa Burkholderia pseudomallei Anaplasma phagocytophilum Helicobacter pylori kx1 npgec* | 9.95E-001 | 16 | 1.46E-013 | 6.97E-001 | 6.97E-001 | 1.00E+000 | 2.45E-001 | 8.66E-001 | 1.00E+000 | 7.55E-001 | 8.43E-001 | 1.00E+000 | 1.00E+000 | 2.59E-002 |
| 1640 | 4 | 5 | *Lactobacillus acidophilus streptococcus pyogenes Pseudomonas aeruginosa Aeromonas cavia* | 9.95E-001 | 0 | 1.00E+000 | 9.47E-001 | 3.03E-001 | 2.77E-001 | 9.37E-001 | 1.00E+000 | 2.65E-001 | 1.00E+000 | 8.43E-001 | 7.19E-001 | 1.00E+000 | 1.00E+000 |
| 940 | 3 | 18 | *Staphylococcus aureus streptococcus pyogenes Helicobacter pylori kx1 npgec* | 9.95E-001 | 48 | 1.06E-010 | 8.84E-001 | 5.00E-001 | 1.63E-001 | 9.81E-001 | 3.21E-001 | 1.00E+000 | 1.00E+000 | 7.45E-001 | 6.08E-001 | 1.00E+000 | 1.00E+000 |
| 944 | 3 | 18 | *Helicobacter pylori kx2 npgec Eubacterium rectale Helicobacter pylori kx1 npgec* | 9.95E-001 | 0 | 1.00E+000 | 1.00E+000 | 1.16E-001 | 6.08E-001 | 8.08E-001 | 3.21E-001 | 1.00E+000 | 1.00E+000 | 3.96E-002 | 1.00E+000 | 1.00E+000 | 1.00E+000 |
| 1802 | 5 | 3 | *Helicobacter pylori kx2 npgec Bacillus anthracis Staphylococcus aureus Porphyromonas gingivalis Helicobacter pylori kx1 npgec* | 9.95E-001 | 0 | 1.00E+000 | 5.00E-001 | 8.28E-001 | 3.93E-001 | 8.69E-001 | 6.08E-002 | 1.00E+000 | 8.32E-001 | 5.96E-001 | 8.00E-001 | 4.10E-001 | 7.21E-001 |
| 1776 | 5 | 3 | *Pseudomonas aeruginosa fdr1 Pseudomonas aeruginosa fdr1234 Lactobacillus acidophilus Pseudomonas aeruginosa fdr875 Bifidobacterium bifidum* | 9.95E-001 | 0 | 1.00E+000 | 5.00E-001 | 8.28E-001 | 3.93E-001 | 8.69E-001 | 2.75E-001 | 3.31E-002 | 1.00E+000 | 1.00E+000 | 1.03E-001 | 1.00E+000 | 1.00E+000 |
| 1790 | 5 | 3 | *Helicobacter pylori kx2 npgec Burkholderia pseudomallei streptococcus pyogenes Fusobacterium nucleatum Helicobacter pylori kx1 npgec* | 9.95E-001 | 0 | 1.00E+000 | 8.28E-001 | 5.00E-001 | 8.00E-001 | 5.54E-001 | 2.75E-001 | 1.00E+000 | 8.32E-001 | 5.96E-001 | 1.00E+000 | 4.10E-001 | 7.21E-001 |
| 1804 | 5 | 3 | *Helicobacter pylori kx2 npgec streptococcus gordonii Pseudomonas aeruginosa fdr1 streptococcus pneumoniae d39 Helicobacter pylori kx1 npgec* | 9.95E-001 | 1 | 4.73E-001 | 5.00E-001 | 8.28E-001 | 3.93E-001 | 8.69E-001 | 5.13E-003 | 1.00E+000 | 1.00E+000 | 5.96E-001 | 3.93E-001 | 4.10E-001 | 1.00E+000 |
| 1786 | 5 | 3 | *Burkholderia pseudomallei Yersinia enterocolitica p60 bl6 streptococcus pneumoniae d39 Escherichia coli Aggregatibacter actinomycetemcomitans* | 9.95E-001 | 0 | 1.00E+000 | 1.72E-001 | 9.76E-001 | 8.00E-001 | 5.54E-001 | 6.41E-001 | 1.00E+000 | 1.31E-001 | 5.96E-001 | 8.00E-001 | 4.10E-001 | 7.21E-001 |
| 1780 | 5 | 3 | *Ehrlichia chaffeensis arkansa Burkholderia pseudomallei Ehrlichia chaffeensis wakulla Escherichia coli Ehrlichia chaffeensis liberty* | 9.95E-001 | 6 | 4.44E-007 | 8.28E-001 | 5.00E-001 | 1.00E+000 | 1.68E-001 | 1.00E+000 | 1.00E+000 | 4.46E-001 | 9.05E-001 | 1.00E+000 | 1.00E+000 | 5.04E-003 |
| 1796 | 5 | 3 | *Burkholderia pseudomallei Ehrlichia chaffeensis wakulla Staphylococcus aureus Listeria monocytogenes Aeromonas cavia* | 9.95E-001 | 5 | 2.83E-005 | 5.00E-001 | 8.28E-001 | 3.93E-001 | 8.69E-001 | 9.23E-001 | 3.23E-001 | 8.32E-001 | 5.96E-001 | 8.00E-001 | 1.00E+000 | 2.88E-001 |
| 1800 | 5 | 3 | *Ehrlichia chaffeensis arkansa streptococcus gordonii Lactobacillus acidophilus Aeromonas cavia Helicobacter pylori kx1 npgec* | 9.95E-001 | 0 | 1.00E+000 | 9.76E-001 | 1.72E-001 | 3.93E-001 | 8.69E-001 | 6.41E-001 | 3.23E-001 | 1.00E+000 | 5.96E-001 | 1.00E+000 | 4.10E-001 | 7.21E-001 |
| 1788 | 5 | 3 | *Helicobacter pylori kx2 npgec Helicobacter pylori kx1 mgep Eubacterium rectale Porphyromonas gingivalis Helicobacter pylori kx2 mgep* | 9.95E-001 | 0 | 1.00E+000 | 9.76E-001 | 1.72E-001 | 8.00E-001 | 5.54E-001 | 6.08E-002 | 1.00E+000 | 1.00E+000 | 4.69E-002 | 1.00E+000 | 4.10E-001 | 1.00E+000 |
| 1798 | 5 | 3 | *Ehrlichia chaffeensis arkansa Burkholderia pseudomallei Lactobacillus acidophilus Pseudomonas aeruginosa fdr875 Ehrlichia chaffeensis liberty* | 9.95E-001 | 0 | 1.00E+000 | 8.28E-001 | 5.00E-001 | 8.00E-001 | 5.54E-001 | 9.23E-001 | 3.23E-001 | 8.32E-001 | 1.00E+000 | 8.00E-001 | 1.00E+000 | 5.72E-002 |
| 1794 | 5 | 3 | *Yersinia enterocolitica p60 bc Pseudomonas aeruginosa fdr1234 Lactobacillus acidophilus Porphyromonas gingivalis Helicobacter pylori kx2 mgep* | 9.95E-001 | 0 | 1.00E+000 | 8.28E-001 | 5.00E-001 | 8.00E-001 | 5.54E-001 | 2.75E-001 | 3.23E-001 | 8.32E-001 | 5.96E-001 | 8.00E-001 | 4.10E-001 | 1.00E+000 |
| 1784 | 5 | 3 | *Burkholderia pseudomallei Ehrlichia chaffeensis wakulla Lactobacillus acidophilus Pseudomonas aeruginosa fdr440 Bifidobacterium bifidum* | 9.95E-001 | 0 | 1.00E+000 | 8.28E-001 | 5.00E-001 | 3.93E-001 | 8.69E-001 | 9.23E-001 | 3.31E-002 | 8.32E-001 | 1.00E+000 | 8.00E-001 | 1.00E+000 | 2.88E-001 |
| 960 | 3 | 17 | *Pseudomonas aeruginosa fdr875 Porphyromonas gingivalis Aggregatibacter actinomycetemcomitans* | 9.96E-001 | 9 | 5.76E-001 | 1.16E-001 | 1.00E+000 | 1.00E+000 | 3.54E-001 | 4.88E-002 | 1.00E+000 | 1.00E+000 | 1.00E+000 | 6.08E-001 | 2.02E-002 | 1.00E+000 |
| 958 | 3 | 17 | *Helicobacter pylori kx1 mgep Ehrlichia chaffeensis arkansa Helicobacter pylori kx2 mgep* | 9.96E-001 | 18 | 5.60E-012 | 1.00E+000 | 1.16E-001 | 1.00E+000 | 3.54E-001 | 3.21E-001 | 1.00E+000 | 1.00E+000 | 2.87E-001 | 1.00E+000 | 1.00E+000 | 5.25E-001 |
| 1068 | 3 | 15 | *Ehrlichia chaffeensis arkansa Lactobacillus acidophilus streptococcus pyogenes* | 9.96E-001 | 33 | 3.82E-021 | 1.00E+000 | 1.16E-001 | 1.63E-001 | 9.81E-001 | 1.00E+000 | 2.04E-001 | 1.00E+000 | 1.00E+000 | 1.00E+000 | 1.00E+000 | 5.25E-001 |
| 1040 | 3 | 16 | *Lactobacillus acidophilus Pseudomonas aeruginosa fdr875 Bifidobacterium bifidum* | 9.97E-001 | 13 | 1.75E-009 | 8.84E-001 | 5.00E-001 | 1.63E-001 | 9.81E-001 | 7.74E-001 | 1.03E-002 | 1.00E+000 | 1.00E+000 | 6.08E-001 | 1.00E+000 | 1.00E+000 |
| 1026 | 3 | 16 | *Yersinia enterocolitica wap bl6 Ehrlichia chaffeensis wakulla Helicobacter pylori kx2 mgep* | 9.97E-001 | 34 | 3.05E-007 | 1.00E+000 | 1.16E-001 | 1.00E+000 | 3.54E-001 | 7.74E-001 | 1.00E+000 | 6.46E-001 | 2.87E-001 | 1.00E+000 | 1.00E+000 | 5.25E-001 |
| 1752 | 4 | 4 | *Helicobacter pylori kx1 mgep Pseudomonas aeruginosa fdr440 Staphylococcus aureus Helicobacter pylori kx2 mgep* | 9.97E-001 | 0 | 1.00E+000 | 6.97E-001 | 6.97E-001 | 7.19E-001 | 6.80E-001 | 1.63E-002 | 1.00E+000 | 1.00E+000 | 4.51E-001 | 2.77E-001 | 1.00E+000 | 1.00E+000 |
| 1754 | 4 | 4 | *Lactobacillus acidophilus Bifidobacterium bifidum streptococcus pyogenes Porphyromonas gingivalis* | 9.97E-001 | 17 | 1.64E-014 | 9.47E-001 | 3.03E-001 | 4.86E-002 | 9.96E-001 | 8.66E-001 | 2.02E-002 | 1.00E+000 | 1.00E+000 | 1.00E+000 | 3.41E-001 | 1.00E+000 |
| 1746 | 4 | 4 | *Pseudomonas aeruginosa fdr875 Staphylococcus aureus Porphyromonas gingivalis Mycobacterium tuberculosis* | 9.97E-001 | 3 | 2.05E-001 | 5.35E-002 | 1.00E+000 | 7.19E-001 | 9.37E-001 | 1.46E-001 | 1.00E+000 | 7.55E-001 | 1.00E+000 | 4.86E-002 | 3.41E-001 | 1.00E+000 |
| 1738 | 4 | 4 | *Helicobacter pylori kx2 npgec streptococcus gordonii Pseudomonas aeruginosa fdr875 Fusobacterium nucleatum* | 9.97E-001 | 0 | 1.00E+000 | 3.03E-001 | 9.47E-001 | 7.19E-001 | 6.80E-001 | 1.63E-002 | 1.00E+000 | 1.00E+000 | 8.43E-001 | 7.19E-001 | 3.91E-002 | 1.00E+000 |
| 1764 | 4 | 4 | *Pseudomonas aeruginosa fdr875 Staphylococcus aureus Bifidobacterium bifidum Helicobacter pylori kx1 npgec* | 9.97E-001 | 0 | 1.00E+000 | 6.97E-001 | 6.97E-001 | 2.77E-001 | 9.37E-001 | 1.46E-001 | 2.65E-001 | 1.00E+000 | 8.43E-001 | 2.77E-001 | 1.00E+000 | 1.00E+000 |
| 1750 | 4 | 4 | *Yersinia enterocolitica wap bl6 Ehrlichia chaffeensis arkansa Pseudomonas aeruginosa fdr440 Helicobacter pylori kx1 npgec* | 9.97E-001 | 7 | 4.14E-005 | 9.47E-001 | 3.03E-001 | 1.00E+000 | 2.45E-001 | 4.95E-001 | 1.00E+000 | 7.55E-001 | 4.51E-001 | 7.19E-001 | 1.00E+000 | 6.34E-001 |
| 876 | 2 | 29 | *Helicobacter pylori kx1 mgep Pseudomonas aeruginosa fdr440* | 9.97E-001 | 20 | 1.79E-003 | 7.56E-001 | 7.56E-001 | 1.00E+000 | 5.05E-001 | 1.39E-001 | 1.00E+000 | 1.00E+000 | 5.92E-001 | 4.60E-001 | 1.00E+000 | 1.00E+000 |
| 1088 | 3 | 14 | *Yersinia enterocolitica p60 bl6 streptococcus pneumoniae d39 Escherichia coli* | 9.97E-001 | 15 | 1.64E-003 | 5.00E-001 | 8.84E-001 | 6.08E-001 | 8.08E-001 | 7.74E-001 | 1.00E+000 | 1.92E-001 | 2.87E-001 | 6.08E-001 | 1.00E+000 | 1.00E+000 |
| 1172 | 3 | 13 | *Pseudomonas aeruginosa Mycobacterium tuberculosis Fusobacterium nucleatum* | 9.98E-001 | 3 | 5.39E-001 | 1.16E-001 | 1.00E+000 | 1.00E+000 | 8.08E-001 | 7.74E-001 | 1.00E+000 | 6.46E-001 | 1.00E+000 | 1.63E-001 | 2.65E-001 | 1.00E+000 |
| 1174 | 3 | 13 | *Ehrlichia chaffeensis arkansa Pseudomonas aeruginosa fdr1 Aeromonas cavia* | 9.98E-001 | 30 | < 1.67E-026 | 8.84E-001 | 5.00E-001 | 1.00E+000 | 3.54E-001 | 7.74E-001 | 1.00E+000 | 1.00E+000 | 7.45E-001 | 6.08E-001 | 1.00E+000 | 5.25E-001 |
| 1178 | 3 | 12 | *Helicobacter pylori kx1 mgep Pseudomonas aeruginosa fdr1 Helicobacter pylori kx2 mgep* | 9.98E-001 | 21 | 2.99E-004 | 8.84E-001 | 5.00E-001 | 1.00E+000 | 3.54E-001 | 4.88E-002 | 1.00E+000 | 1.00E+000 | 2.87E-001 | 6.08E-001 | 1.00E+000 | 1.00E+000 |
| 1214 | 3 | 12 | *Ehrlichia chaffeensis arkansa Pseudomonas aeruginosa fdr440 Aeromonas cavia* | 9.98E-001 | 12 | 5.43E-008 | 8.84E-001 | 5.00E-001 | 1.00E+000 | 3.54E-001 | 7.74E-001 | 1.00E+000 | 1.00E+000 | 7.45E-001 | 6.08E-001 | 1.00E+000 | 5.25E-001 |
| 1210 | 3 | 12 | *Helicobacter pylori kx2 npgec Brucella ovis Helicobacter pylori kx1 npgec* | 9.98E-001 | 9 | 1.56E-001 | 1.00E+000 | 1.16E-001 | 1.00E+000 | 3.54E-001 | 3.21E-001 | 1.00E+000 | 6.46E-001 | 2.87E-001 | 1.00E+000 | 1.00E+000 | 5.25E-001 |
| 1182 | 3 | 12 | *Yersinia enterocolitica p60 bl6 Pseudomonas aeruginosa fdr440 Porphyromonas gingivalis* | 9.98E-001 | 11 | 1.01E-001 | 5.00E-001 | 8.84E-001 | 1.00E+000 | 3.54E-001 | 3.21E-001 | 1.00E+000 | 6.46E-001 | 7.45E-001 | 6.08E-001 | 2.65E-001 | 1.00E+000 |
| 1204 | 3 | 12 | *Fusobacterium nucleatum Escherichia coli Aggregatibacter actinomycetemcomitans* | 9.98E-001 | 7 | 8.80E-001 | 1.16E-001 | 1.00E+000 | 1.00E+000 | 3.54E-001 | 3.21E-001 | 1.00E+000 | 6.46E-001 | 7.45E-001 | 1.00E+000 | 2.02E-002 | 1.00E+000 |
| 1230 | 3 | 12 | *Helicobacter pylori kx2 npgec streptococcus pyogenes Mycobacterium tuberculosis* | 9.98E-001 | 24 | 2.11E-012 | 8.84E-001 | 5.00E-001 | 6.08E-001 | 9.81E-001 | 7.74E-001 | 1.00E+000 | 6.46E-001 | 7.45E-001 | 6.08E-001 | 1.00E+000 | 1.00E+000 |
| 1854 | 4 | 3 | *Helicobacter pylori kx2 npgec Fusobacterium nucleatum Brucella ovis Helicobacter pylori kx1 npgec* | 9.98E-001 | 5 | 8.17E-002 | 9.47E-001 | 3.03E-001 | 1.00E+000 | 2.45E-001 | 1.46E-001 | 1.00E+000 | 7.55E-001 | 4.51E-001 | 1.00E+000 | 3.41E-001 | 6.34E-001 |
| 1832 | 4 | 3 | *Helicobacter pylori kx1 mgep Porphyromonas gingivalis Ehrlichia chaffeensis liberty Aggregatibacter actinomycetemcomitans* | 9.98E-001 | 5 | 7.15E-002 | 6.97E-001 | 6.97E-001 | 1.00E+000 | 2.45E-001 | 1.46E-001 | 1.00E+000 | 1.00E+000 | 8.43E-001 | 1.00E+000 | 3.91E-002 | 6.34E-001 |
| 1834 | 4 | 3 | *Helicobacter pylori kx2 npgec Staphylococcus aureus Brucella ovis Helicobacter pylori kx1 npgec* | 9.98E-001 | 5 | 1.30E-001 | 9.47E-001 | 3.03E-001 | 7.19E-001 | 6.80E-001 | 1.46E-001 | 1.00E+000 | 7.55E-001 | 4.51E-001 | 7.19E-001 | 1.00E+000 | 6.34E-001 |
| 1840 | 4 | 3 | *Eubacterium rectale streptococcus pneumoniae g54 Porphyromonas gingivalis Aggregatibacter actinomycetemcomitans* | 9.98E-001 | 0 | 1.00E+000 | 3.03E-001 | 9.47E-001 | 2.77E-001 | 9.37E-001 | 1.46E-001 | 1.00E+000 | 1.00E+000 | 8.43E-001 | 7.19E-001 | 3.91E-002 | 1.00E+000 |
| 1824 | 4 | 3 | *Yersinia enterocolitica p60 bc Lactobacillus acidophilus Pseudomonas aeruginosa fdr875 Porphyromonas gingivalis* | 9.98E-001 | 0 | 1.00E+000 | 6.97E-001 | 6.97E-001 | 7.19E-001 | 6.80E-001 | 4.95E-001 | 2.65E-001 | 7.55E-001 | 8.43E-001 | 7.19E-001 | 3.41E-001 | 1.00E+000 |
| 1818 | 4 | 3 | *Helicobacter pylori kx1 mgep Burkholderia pseudomallei Anaplasma phagocytophilum Helicobacter pylori kx2 mgep* | 9.98E-001 | 0 | 1.00E+000 | 6.97E-001 | 6.97E-001 | 1.00E+000 | 2.45E-001 | 4.95E-001 | 1.00E+000 | 7.55E-001 | 4.51E-001 | 1.00E+000 | 1.00E+000 | 1.96E-001 |
| 1838 | 4 | 3 | *Helicobacter pylori kx2 npgec Ehrlichia chaffeensis arkansa Pseudomonas aeruginosa fdr875 Helicobacter pylori kx1 npgec* | 9.98E-001 | 10 | 5.09E-008 | 9.47E-001 | 3.03E-001 | 1.00E+000 | 2.45E-001 | 1.46E-001 | 1.00E+000 | 1.00E+000 | 4.51E-001 | 7.19E-001 | 1.00E+000 | 6.34E-001 |
| 1862 | 4 | 3 | *Pseudomonas aeruginosa fdr875 Staphylococcus aureus Helicobacter pylori kx2 mgep Aeromonas cavia* | 9.98E-001 | 0 | 1.00E+000 | 6.97E-001 | 6.97E-001 | 7.19E-001 | 6.80E-001 | 1.46E-001 | 1.00E+000 | 1.00E+000 | 4.51E-001 | 2.77E-001 | 1.00E+000 | 1.00E+000 |
| 1852 | 4 | 3 | *Pseudomonas aeruginosa fdr1234 Pseudomonas aeruginosa fdr875 Staphylococcus aureus Porphyromonas gingivalis* | 9.98E-001 | 3 | 4.72E-001 | 5.35E-002 | 1.00E+000 | 7.19E-001 | 6.80E-001 | 1.63E-002 | 1.00E+000 | 1.00E+000 | 1.00E+000 | 4.86E-002 | 3.41E-001 | 1.00E+000 |
| 1828 | 4 | 3 | *Burkholderia pseudomallei Pseudomonas aeruginosa fdr440 streptococcus pyogenes Escherichia coli* | 9.98E-001 | 0 | 1.00E+000 | 3.03E-001 | 9.47E-001 | 7.19E-001 | 6.80E-001 | 8.66E-001 | 1.00E+000 | 3.20E-001 | 8.43E-001 | 7.19E-001 | 1.00E+000 | 6.34E-001 |
| 1288 | 3 | 11 | *Listeria monocytogenes Pseudomonas aeruginosa Aeromonas cavia* | 9.98E-001 | 4 | 2.86E-002 | 5.00E-001 | 8.84E-001 | 6.08E-001 | 8.08E-001 | 1.00E+000 | 2.04E-001 | 1.00E+000 | 2.87E-001 | 6.08E-001 | 1.00E+000 | 1.00E+000 |
| 1284 | 3 | 11 | *Pseudomonas aeruginosa fdr1234 Staphylococcus aureus Helicobacter pylori kx2 mgep* | 9.98E-001 | 5 | 4.30E-001 | 5.00E-001 | 8.84E-001 | 6.08E-001 | 8.08E-001 | 4.88E-002 | 1.00E+000 | 1.00E+000 | 7.45E-001 | 1.63E-001 | 1.00E+000 | 1.00E+000 |
| 1286 | 3 | 11 | *Helicobacter pylori kx1 mgep Pseudomonas aeruginosa Escherichia coli* | 9.98E-001 | 7 | 3.54E-003 | 5.00E-001 | 8.84E-001 | 1.00E+000 | 3.54E-001 | 7.74E-001 | 1.00E+000 | 6.46E-001 | 2.87E-001 | 6.08E-001 | 1.00E+000 | 1.00E+000 |
| 1276 | 3 | 11 | *Ehrlichia chaffeensis wakulla Aeromonas cavia Escherichia coli* | 9.98E-001 | 26 | 9.95E-010 | 8.84E-001 | 5.00E-001 | 1.00E+000 | 3.54E-001 | 1.00E+000 | 1.00E+000 | 6.46E-001 | 2.87E-001 | 1.00E+000 | 1.00E+000 | 5.25E-001 |
| 1364 | 3 | 10 | *streptococcus pyogenes Escherichia coli Ehrlichia chaffeensis liberty* | 9.99E-001 | 23 | 4.18E-012 | 8.84E-001 | 5.00E-001 | 6.08E-001 | 8.08E-001 | 1.00E+000 | 1.00E+000 | 6.46E-001 | 7.45E-001 | 1.00E+000 | 1.00E+000 | 5.25E-001 |
| 1342 | 3 | 10 | *Burkholderia pseudomallei Anaplasma phagocytophilum Escherichia coli* | 9.99E-001 | 0 | 1.00E+000 | 1.16E-001 | 1.00E+000 | 1.00E+000 | 3.54E-001 | 1.00E+000 | 1.00E+000 | 1.92E-001 | 7.45E-001 | 1.00E+000 | 1.00E+000 | 1.11E-001 |
| 1368 | 3 | 10 | *Ehrlichia chaffeensis arkansa Ehrlichia chaffeensis wakulla Pseudomonas aeruginosa fdr440* | 9.99E-001 | 17 | 4.58E-009 | 8.84E-001 | 5.00E-001 | 1.00E+000 | 3.54E-001 | 7.74E-001 | 1.00E+000 | 1.00E+000 | 1.00E+000 | 6.08E-001 | 1.00E+000 | 1.11E-001 |
| 1458 | 3 | 9 | *Yersinia enterocolitica wap bl6 Bifidobacterium bifidum streptococcus pyogenes* | 9.99E-001 | 44 | 3.57E-013 | 1.00E+000 | 1.16E-001 | 1.63E-001 | 9.81E-001 | 1.00E+000 | 2.04E-001 | 6.46E-001 | 7.45E-001 | 1.00E+000 | 1.00E+000 | 1.00E+000 |
| 1440 | 3 | 9 | *Anaplasma phagocytophilum Helicobacter pylori kx2 mgep Helicobacter pylori kx1 npgec* | 9.99E-001 | 41 | < 1.67E-026 | 8.84E-001 | 5.00E-001 | 1.00E+000 | 3.54E-001 | 3.21E-001 | 1.00E+000 | 1.00E+000 | 2.87E-001 | 1.00E+000 | 1.00E+000 | 5.25E-001 |
| 1556 | 3 | 8 | *Pseudomonas aeruginosa fdr1234 Helicobacter pylori kx1 npgec Ehrlichia chaffeensis liberty* | 9.99E-001 | 17 | 3.40E-006 | 8.84E-001 | 5.00E-001 | 1.00E+000 | 3.54E-001 | 3.21E-001 | 1.00E+000 | 1.00E+000 | 7.45E-001 | 6.08E-001 | 1.00E+000 | 5.25E-001 |
| 1552 | 3 | 8 | *Helicobacter pylori kx2 npgec Pseudomonas aeruginosa fdr1234 Mycobacterium tuberculosis* | 9.99E-001 | 13 | 8.31E-009 | 5.00E-001 | 8.84E-001 | 1.00E+000 | 8.08E-001 | 3.21E-001 | 1.00E+000 | 6.46E-001 | 7.45E-001 | 1.63E-001 | 1.00E+000 | 1.00E+000 |
| 1516 | 3 | 8 | *Pseudomonas aeruginosa fdr1234 Pseudomonas aeruginosa Aeromonas cavia* | 9.99E-001 | 7 | 3.67E-006 | 5.00E-001 | 8.84E-001 | 1.00E+000 | 3.54E-001 | 7.74E-001 | 1.00E+000 | 1.00E+000 | 7.45E-001 | 1.63E-001 | 1.00E+000 | 1.00E+000 |
| 1532 | 3 | 8 | *Helicobacter pylori kx1 mgep Pseudomonas aeruginosa fdr875 Aggregatibacter actinomycetemcomitans* | 9.99E-001 | 0 | 1.00E+000 | 5.00E-001 | 8.84E-001 | 1.00E+000 | 3.54E-001 | 4.88E-002 | 1.00E+000 | 1.00E+000 | 7.45E-001 | 6.08E-001 | 2.65E-001 | 1.00E+000 |
| 1580 | 3 | 7 | *Ehrlichia chaffeensis arkansa Escherichia coli Aggregatibacter actinomycetemcomitans* | 9.99E-001 | 3 | 6.40E-001 | 5.00E-001 | 8.84E-001 | 1.00E+000 | 3.54E-001 | 7.74E-001 | 1.00E+000 | 6.46E-001 | 7.45E-001 | 1.00E+000 | 2.65E-001 | 5.25E-001 |
| 1592 | 3 | 7 | *Yersinia enterocolitica p60 bl6 streptococcus gordonii streptococcus pneumoniae d39* | 9.99E-001 | 19 | 2.61E-002 | 5.00E-001 | 8.84E-001 | 1.63E-001 | 9.81E-001 | 3.21E-001 | 1.00E+000 | 6.46E-001 | 7.45E-001 | 6.08E-001 | 2.65E-001 | 1.00E+000 |
| 1598 | 3 | 7 | *Helicobacter pylori kx1 mgep Pseudomonas aeruginosa Aggregatibacter actinomycetemcomitans* | 9.99E-001 | 1 | 9.56E-001 | 5.00E-001 | 8.84E-001 | 1.00E+000 | 3.54E-001 | 3.21E-001 | 1.00E+000 | 1.00E+000 | 7.45E-001 | 6.08E-001 | 2.65E-001 | 1.00E+000 |
| 1590 | 3 | 7 | *Yersinia enterocolitica wap bl6 Listeria monocytogenes Pseudomonas aeruginosa* | 9.99E-001 | 3 | 2.28E-001 | 5.00E-001 | 8.84E-001 | 6.08E-001 | 8.08E-001 | 1.00E+000 | 2.04E-001 | 6.46E-001 | 2.87E-001 | 6.08E-001 | 1.00E+000 | 1.00E+000 |
| 1586 | 3 | 7 | *Yersinia enterocolitica wap bc Yersinia enterocolitica p60 bc Listeria monocytogenes* | 9.99E-001 | 0 | 1.00E+000 | 8.84E-001 | 5.00E-001 | 6.08E-001 | 8.08E-001 | 1.00E+000 | 2.04E-001 | 1.92E-001 | 3.96E-002 | 1.00E+000 | 1.00E+000 | 1.00E+000 |
| 1712 | 3 | 6 | *Pseudomonas aeruginosa fdr875 Bifidobacterium bifidum Mycobacterium tuberculosis* | 9.99E-001 | 13 | 7.68E-007 | 5.00E-001 | 8.84E-001 | 6.08E-001 | 9.81E-001 | 7.74E-001 | 2.04E-001 | 6.46E-001 | 1.00E+000 | 1.63E-001 | 1.00E+000 | 1.00E+000 |
| 1708 | 3 | 6 | *Helicobacter pylori kx2 npgec Pseudomonas aeruginosa fdr1234 streptococcus pneumoniae d39* | 9.99E-001 | 3 | 2.28E-001 | 5.00E-001 | 8.84E-001 | 6.08E-001 | 8.08E-001 | 4.88E-002 | 1.00E+000 | 1.00E+000 | 7.45E-001 | 1.63E-001 | 1.00E+000 | 1.00E+000 |
| 1696 | 3 | 6 | *Bifidobacterium bifidum streptococcus pyogenes Aggregatibacter actinomycetemcomitans* | 9.99E-001 | 16 | 3.03E-009 | 8.84E-001 | 5.00E-001 | 1.63E-001 | 9.81E-001 | 7.74E-001 | 2.04E-001 | 1.00E+000 | 1.00E+000 | 1.00E+000 | 2.65E-001 | 1.00E+000 |
| 1366 | 2 | 15 | *Eubacterium rectale Aeromonas cavia* | 9.99E-001 | 21 | 8.03E-005 | 1.00E+000 | 2.44E-001 | 4.60E-001 | 9.23E-001 | 1.00E+000 | 1.00E+000 | 1.00E+000 | 1.22E-001 | 1.00E+000 | 1.00E+000 | 1.00E+000 |
| 1792 | 3 | 5 | *Helicobacter pylori kx1 mgep Burkholderia pseudomallei Pseudomonas aeruginosa fdr875* | 1.00E+000 | 0 | 1.00E+000 | 5.00E-001 | 8.84E-001 | 1.00E+000 | 3.54E-001 | 3.21E-001 | 1.00E+000 | 6.46E-001 | 7.45E-001 | 6.08E-001 | 1.00E+000 | 5.25E-001 |
| 1778 | 3 | 5 | *streptococcus pyogenes Porphyromonas gingivalis Aeromonas cavia* | 1.00E+000 | 18 | 2.83E-005 | 8.84E-001 | 5.00E-001 | 6.08E-001 | 8.08E-001 | 7.74E-001 | 1.00E+000 | 1.00E+000 | 7.45E-001 | 1.00E+000 | 2.65E-001 | 1.00E+000 |
| 1782 | 3 | 5 | *Ehrlichia chaffeensis arkansa Pseudomonas aeruginosa fdr440 Porphyromonas gingivalis* | 1.00E+000 | 9 | 6.88E-006 | 5.00E-001 | 8.84E-001 | 1.00E+000 | 3.54E-001 | 3.21E-001 | 1.00E+000 | 1.00E+000 | 1.00E+000 | 6.08E-001 | 2.65E-001 | 5.25E-001 |
| 1774 | 3 | 5 | *Yersinia enterocolitica p60 bc streptococcus gordonii Pseudomonas aeruginosa* | 1.00E+000 | 2 | 3.50E-001 | 5.00E-001 | 8.84E-001 | 6.08E-001 | 8.08E-001 | 7.74E-001 | 1.00E+000 | 6.46E-001 | 7.45E-001 | 6.08E-001 | 2.65E-001 | 1.00E+000 |
| 1820 | 3 | 4 | *Fusobacterium nucleatum Helicobacter pylori kx2 mgep Aggregatibacter actinomycetemcomitans* | 1.00E+000 | 4 | 1.70E-001 | 5.00E-001 | 8.84E-001 | 1.00E+000 | 3.54E-001 | 4.88E-002 | 1.00E+000 | 1.00E+000 | 7.45E-001 | 1.00E+000 | 2.02E-002 | 1.00E+000 |
| 1860 | 3 | 4 | *Pseudomonas aeruginosa fdr875 Staphylococcus aureus Ehrlichia chaffeensis liberty* | 1.00E+000 | 3 | 3.76E-001 | 5.00E-001 | 8.84E-001 | 6.08E-001 | 8.08E-001 | 3.21E-001 | 1.00E+000 | 1.00E+000 | 1.00E+000 | 1.63E-001 | 1.00E+000 | 5.25E-001 |
| 1836 | 3 | 4 | *Helicobacter pylori kx2 npgec Brucella ovis Helicobacter pylori kx2 mgep* | 1.00E+000 | 0 | 1.00E+000 | 1.00E+000 | 1.16E-001 | 1.00E+000 | 3.54E-001 | 3.21E-001 | 1.00E+000 | 6.46E-001 | 2.87E-001 | 1.00E+000 | 1.00E+000 | 5.25E-001 |
| 1826 | 3 | 4 | *Helicobacter pylori kx1 mgep streptococcus gordonii Helicobacter pylori kx2 mgep* | 1.00E+000 | 1 | 3.07E-001 | 8.84E-001 | 5.00E-001 | 6.08E-001 | 8.08E-001 | 4.88E-002 | 1.00E+000 | 1.00E+000 | 2.87E-001 | 1.00E+000 | 2.65E-001 | 1.00E+000 |
| 1510 | 2 | 12 | *Eubacterium rectale Porphyromonas gingivalis* | 1.00E+000 | 0 | 1.00E+000 | 7.56E-001 | 7.56E-001 | 4.60E-001 | 9.23E-001 | 6.23E-001 | 1.00E+000 | 1.00E+000 | 5.92E-001 | 1.00E+000 | 1.84E-001 | 1.00E+000 |
| 1512 | 2 | 12 | *Eubacterium rectale Bifidobacterium bifidum* | 1.00E+000 | 1 | 8.89E-001 | 1.00E+000 | 2.44E-001 | 6.39E-002 | 1.00E+000 | 1.00E+000 | 1.39E-001 | 1.00E+000 | 5.92E-001 | 1.00E+000 | 1.00E+000 | 1.00E+000 |
| 1524 | 2 | 12 | *Eubacterium rectale Helicobacter pylori kx2 mgep* | 1.00E+000 | 20 | 4.50E-006 | 1.00E+000 | 2.44E-001 | 4.60E-001 | 9.23E-001 | 6.23E-001 | 1.00E+000 | 1.00E+000 | 1.22E-001 | 1.00E+000 | 1.00E+000 | 1.00E+000 |
| 1902 | 3 | 3 | *Yersinia enterocolitica wap bc Bifidobacterium bifidum Helicobacter pylori kx2 mgep* | 1.00E+000 | 15 | 8.01E-014 | 1.00E+000 | 1.16E-001 | 6.08E-001 | 8.08E-001 | 7.74E-001 | 2.04E-001 | 6.46E-001 | 2.87E-001 | 1.00E+000 | 1.00E+000 | 1.00E+000 |
| 1910 | 3 | 3 | *Pseudomonas aeruginosa fdr875 Mycobacterium tuberculosis Aeromonas cavia* | 1.00E+000 | 2 | 2.16E-001 | 5.00E-001 | 8.84E-001 | 1.00E+000 | 8.08E-001 | 7.74E-001 | 1.00E+000 | 6.46E-001 | 7.45E-001 | 1.63E-001 | 1.00E+000 | 1.00E+000 |
| 1908 | 3 | 3 | *Burkholderia pseudomallei Pseudomonas aeruginosa fdr1234 streptococcus pneumoniae d39* | 1.00E+000 | 0 | 1.00E+000 | 1.16E-001 | 1.00E+000 | 6.08E-001 | 8.08E-001 | 3.21E-001 | 1.00E+000 | 6.46E-001 | 1.00E+000 | 1.63E-001 | 1.00E+000 | 5.25E-001 |
| 1900 | 3 | 3 | *streptococcus gordonii Listeria monocytogenes Escherichia coli* | 1.00E+000 | 0 | 1.00E+000 | 1.16E-001 | 1.00E+000 | 1.63E-001 | 9.81E-001 | 7.74E-001 | 2.04E-001 | 6.46E-001 | 2.87E-001 | 1.00E+000 | 2.65E-001 | 1.00E+000 |
| 1906 | 3 | 3 | *Ehrlichia chaffeensis wakulla Pseudomonas aeruginosa fdr440 streptococcus pneumoniae d39* | 1.00E+000 | 0 | 1.00E+000 | 5.00E-001 | 8.84E-001 | 6.08E-001 | 8.08E-001 | 3.21E-001 | 1.00E+000 | 1.00E+000 | 1.00E+000 | 1.63E-001 | 1.00E+000 | 5.25E-001 |
| 1904 | 3 | 3 | *Helicobacter pylori kx1 mgep Yersinia enterocolitica p60 bl6 Pseudomonas aeruginosa fdr440* | 1.00E+000 | 6 | 1.22E-002 | 8.84E-001 | 5.00E-001 | 1.00E+000 | 3.54E-001 | 3.21E-001 | 1.00E+000 | 6.46E-001 | 2.87E-001 | 6.08E-001 | 1.00E+000 | 1.00E+000 |
| 1690 | 2 | 9 | *Yersinia enterocolitica wap bl6 Pseudomonas aeruginosa fdr875* | 1.00E+000 | 4 | 4.08E-001 | 7.56E-001 | 7.56E-001 | 1.00E+000 | 5.05E-001 | 6.23E-001 | 1.00E+000 | 4.95E-001 | 5.92E-001 | 4.60E-001 | 1.00E+000 | 1.00E+000 |
| 1686 | 2 | 9 | *Brucella ovis Escherichia coli* | 1.00E+000 | 15 | 2.17E-003 | 7.56E-001 | 7.56E-001 | 1.00E+000 | 5.05E-001 | 1.00E+000 | 1.00E+000 | 7.67E-002 | 5.92E-001 | 1.00E+000 | 1.00E+000 | 3.87E-001 |
| 1916 | 2 | 4 | *Eubacterium rectale Pseudomonas aeruginosa fdr440* | 1.00E+000 | 6 | 4.91E-002 | 7.56E-001 | 7.56E-001 | 4.60E-001 | 9.23E-001 | 6.23E-001 | 1.00E+000 | 1.00E+000 | 5.92E-001 | 4.60E-001 | 1.00E+000 | 1.00E+000 |
| 1920 | 2 | 4 | *streptococcus gordonii streptococcus pyogenes* | 1.00E+000 | 15 | 7.29E-003 | 7.56E-001 | 7.56E-001 | 6.39E-002 | 1.00E+000 | 6.23E-001 | 1.00E+000 | 1.00E+000 | 1.00E+000 | 1.00E+000 | 1.84E-001 | 1.00E+000 |
| 1912 | 2 | 4 | *Eubacterium rectale Mycobacterium tuberculosis* | 1.00E+000 | 0 | 1.00E+000 | 7.56E-001 | 7.56E-001 | 4.60E-001 | 1.00E+000 | 1.00E+000 | 1.00E+000 | 4.95E-001 | 5.92E-001 | 4.60E-001 | 1.00E+000 | 1.00E+000 |
| 1918 | 2 | 4 | *Anaplasma phagocytophilum streptococcus pyogenes* | 1.00E+000 | 64 | < 1.67E-026 | 7.56E-001 | 7.56E-001 | 4.60E-001 | 9.23E-001 | 1.00E+000 | 1.00E+000 | 1.00E+000 | 1.00E+000 | 1.00E+000 | 1.00E+000 | 3.87E-001 |
